# Fragment‐Based Design of Targeted Covalent Inhibitors: The Scope and Limitation of Linking Approaches

**DOI:** 10.1002/cmdc.202501108

**Published:** 2026-05-22

**Authors:** Levente Kollár, Levente M. Mihalovits, Dávid Bajusz, Damijan Knez, József Simon, Blake H. Balcomb, Daren Fearon, Stanislav Gobec, György M. Keserű

**Affiliations:** ^1^ Medicinal Chemistry Research Group and Drug Innovation Centre HUN‐REN Research Centre for Natural Sciences Budapest Hungary; ^2^ Department of Organic Chemistry and Technology Faculty of Chemical Technology and Biotechnology Budapest University of Technology and Economics Budapest Hungary; ^3^ Department of Medicinal Chemistry Faculty of Pharmacy University of Ljubljana Ljubljana Slovenia; ^4^ MS Metabolomics Research Group HUN‐REN Research Centre for Natural Sciences Budapest Hungary; ^5^ Diamond Light Source Harwell Science and Innovation Campus Didcot UK; ^6^ Research Complex at Harwell Harwell Science and Innovation Campus Didcot UK

**Keywords:** 3CL^pro^ inhibitors, covalent inhibitors, fragment linking, fragment‐based drug discovery, SARS‐CoV‐2

## Abstract

Linking of fragments in neighboring binding sites is one of the optimization strategies in fragment‐based drug discovery, where additive or even more substantial bioactivity improvements can be realized. However, such efforts present a considerable challenge when one fragment binds covalently to the target protein, as small modifications can influence the correct positioning of the covalent warhead toward the targeted nucleophilic residue. Here, we present a case study of fragment linking that yielded single‐digit micromolar, covalent inhibitors of the SARS‐CoV‐2 main protease, starting from fragments that were inactive in the biochemical assay. Using structural information from a recent, high‐throughput crystallographic fragment screen, we show that the success of fragment linking in the design of targeted covalent inhibitors is heavily impacted by several factors, including the warhead type, the labeling chemistry, and even subtle changes in the designed linker. Notably, we observe that induced fit effects might override the original fragment orientations in the linked molecule, highlighting the need for reliable structure verification, especially in consecutive rounds of fragment elaboration.

## Graphical abstract

This paper by Kollár et al. reports a covalent fragment linking study against the SARS‐CoV‐2 main protease (3CL^Pro^). Beginning with inactive fragment hits, the application of rational linker design enabled the generation of reversible covalent inhibitors exhibiting low micromolar potency. The study details some general limitations and best practices of the electrophile‐first approach of covalent fragment linking.

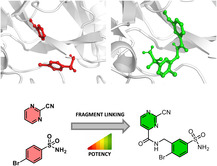

**Abbreviations**
3CL^pro^
: 3‐chymotrypsin‐like protease
DABCO: 1,4‐diazabicyclo[2.2.2]octane
DCC: N,N′‐dicyclohexylcarbodiimide
DCM: dichloromethane
DIPEA: N,N‐diisopropylethylamine
DMF: N,N‐dimethylformamide
DMSO: dimethylsulfoxide
FBDD: fragment‐based drug discovery
HATU: Hexafluorophosphate Azabenzotriazole Tetramethyl Uronium
IFD: induced fit docking
M^pro^
: main protease
MS: mass spectrometry
NHS: N‐hydroxysuccinimide
NMR: nuclear magnetic resonance
PDA: photodiode array
PDB: Protein Data Bank
RA: remaining activity
RMSD: Root Mean Square Deviation
SAR: structure-activity relationship
SPhos: dicyclohexyl(2′,6′‐dimethoxy[1,1′‐biphenyl]‐2‐yl)phosphane
TFA: trifluoroacetic acid
THF: tetrahydrofuran

## 1 Introduction

The first successes of fragment‐based drug discovery (FBDD) date back to the late 1990s [1, 2, 3]. The potential of FBDD was quickly recognized by the pharmaceutical industry and has been used widely as a tool for hit discovery over the past two decades [4]. Fragments are compounds with small molecular weight and relative chemical simplicity. To describe their general physicochemical parameters, Congreve and colleagues coined the Rule of 3 (Ro3), which includes the molecular weight (≤300 Da), the number of hydrogen bond donors and acceptors (≤3 for both separately), and the calculated logP (≤3) [5]. The concept has been widely adopted; however, subsequent fragment screening experiments revealed that additional molecular descriptors can be considered to increase the success rate of FBDD. Although compounds that match Ro3 are not necessarily useful fragments, Ro3 provides a preliminary tool to eliminate molecules with undesired properties [6, 7, 8]. It is worth noting that other, at times simpler definitions have also been used to describe fragment‐like chemical space (i.e., compounds between 10 and 16 heavy atoms) [9].

The first step in an FBDD campaign is to identify fragment‐sized hits. The small size of the molecules under investigation means that they typically have a weak affinity for the target due to the limited possibilities of protein‐ligand interactions and the limited complementarity with the binding site. Therefore, the screening methods used have been adapted accordingly to overcome this shortcoming. Techniques that are sufficiently sensitive and thus most commonly used for screening include nuclear magnetic resonance (NMR) [10], X‐ray crystallography [11], and surface plasmon resonance (SPR) [12]. Additionally, other approaches, such as differential scanning fluorimetry (DSF), isothermal titration calorimetry (ITC), and computational methods offer useful alternatives [13, 14]. The main advantage of the fragment‐based approach comes from the core idea behind the method: by screening molecules of limited size, we can increase the probability of a binding event and cover the chemical space more efficiently with a carefully assembled library [4, 13, 15, 16]. This — together with recent, highly developed screening techniques and a wealth of accumulated experience — significantly improves the success and efficiency of hit‐finding attempts. However, the maturation of hits into lead molecules is far more difficult and time‐consuming, as illustrated by the small (but growing) share of FBDD‐related lead optimization strategies in literature data [17].

**FIGURE 1:**
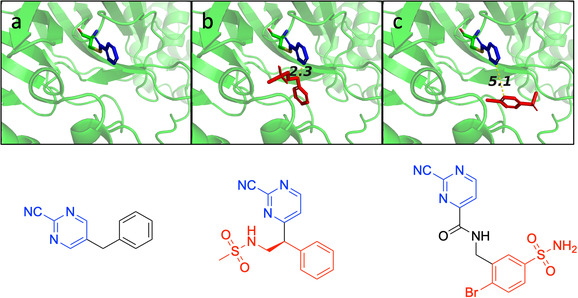
Strategies for fragment development: (a) growing, (b) merging, and (c) linking. The examples involve 3CL^pro^ versus fragment structures investigated in this study, with interatomic distances between the linkage points displayed in Angstroms.

There are three general strategies for the optimization process, namely fragment growing, fragment merging, and fragment linking (Figure 1) [4, 18]. Fragment growing is by far the most widely used technique and involves the optimization of a hit molecule, including the insertion and/or substitution of various chemical groups to improve affinity. Fragment merging can be applied when two identified hits have an overlapping binding site, while fragment linking addresses the case when two hits are in close proximity and have adjacent binding sites [14].

As of January 2020, 46 molecules have progressed to at least Phase I clinical trials, where a fragment‐based strategy played a key role in their discovery. Out of these, 41 are the result of growing, but only 4 of linking, and 1 of merging strategies [19]. It is also noteworthy that dozens of successful fragment‐to‐lead research projects have been published in recent years, and the number of FBDD programs in the clinical stage has reached 59 by 2024 [20], including 7 that already provided approved drugs. It has been applied to a wide range of targets such as kinases, proteases, G‐protein coupled receptors (GPCRs), protein–protein interactions (PPIs), and other targets [21, 22, 23, 24, 25, 26, 27, 28].

The concept of fragment linking is elegant and simple, but it is considered the most challenging strategy to implement. It holds the promise of a leap in affinity; however, designing an appropriate linker that does not disrupt the original binding modes of fragments is particularly challenging [18]. This difficulty is even greater when the distance between the two fragments is large (>3 Å) [19]. In addition, linking can lead to an altered binding mode, protonation/tautomeric state of the fragments, internal strain energy of the linked molecule, or unfavorable interactions between the target and the linker. Moreover, the linkage might decrease the relative configurational entropy of the fragments, which was assumed to be the main reason for the failure of most fragment linking strategies [29]. Despite all discussed difficulties, there are success stories, such as the discovery of venetoclax, a selective Bcl‐2 inhibitor, which was approved to treat chronic lymphocytic leukaemia [30, 31]. Besides that, a number of leads have been generated by fragment linking [19].

At the dawn of fragment‐based drug discovery, fragment libraries were specifically designed to exclude molecules that could potentially form covalent bonds with the target proteins [14]. However, around the same time, the potential of covalent inhibitors was recognized, which in turn has influenced fragment‐based drug discovery. Nowadays, covalent fragments are an integral part of the drug discovery toolbox and play an important role in target identification and validation, as well as hit identification [32, 33, 34]. Although rarely selective, they can possess relatively high affinity compared to non‐covalent fragments. During a covalent fragment‐to‐lead campaign, affinity and selectivity should be carefully optimized together. One of the greatest advantages of the application of covalent FBDD is that it provides hits for targets previously thought to be “undruggable”. An example of that is the discovery of sotorasib—a selective KRas^G12C^ inhibitor—and its preceding hit [35, 36, 37].

Covalent drug discovery projects can follow two different strategies, widely termed as “ligand‐first” and “electrophile‐first” approaches. The former means the purposeful addition of reactive functional groups to existing non‐covalent ligands, while the latter consists of screening covalent ligands and optimizing the non‐covalent core or even by replacing the warhead with another [34]. Both can be effective, as demonstrated by examples: the ligand‐first approach afforded epidermal growth factor receptor (EGFR) inhibitors (e.g., osimertinib, rociletinib) [38, 39] and the Bruton's tyrosine kinase (BTK) inhibitor ibrutinib [40, 41], while the electrophile‐first approach resulted in the aforementioned KRas^G12C^ inhibitor sotorasib [35, 36, 37] and a SARS‐CoV‐2 main protease (M^pro^ or 3CL^pro^) inhibitor, nirmatrelvir [42].

We note that, as in non‐covalent fragment‐to‐lead optimization, growing is the most common strategy for covalent fragment hit maturation, with merging sometimes being justified [43]. Examples of fragment linking are scarce; however, in a recent study, an electrophilic fragment screen of two hundred acrylate methyl esters resulted in a selective binder of BRD4‐BD2 that was linked via a PEG linker to a known non‐covalent BRD4‐BD1 inhibitor, which resulted in a covalent BRD4 inhibitor with cellular activity [44]. Considering that the linked motifs bind at different domains of the target and one of them is not a fragment at all, this study cannot be considered as a typical fragment linking experience.

Therefore, we were prompted to gain insight into covalent fragment linking based on a recent fragment screening campaign against the SARS‐CoV‐2 main protease, 3CL^pro^ [45], a primary target for small‐molecule drug discovery against SARS‐CoV‐2. The main protease cleaves its substrate at eleven sites to release non‐structural proteins (nsp4–16), which form the replicase complex responsible for replication and transcription of the viral genome [45, 46]. 3CL^pro^ is a highly conserved cysteine protease [47, 48, 49, 50], and contains a catalytic His41‐Cys145 dyad at the active site. Therefore, it holds great potential for the development of covalent ligands targeting Cys145 [51], as demonstrated by its only approved inhibitor, nirmatrelvir. It is important to add that multiple mutations have recently been identified in 3CL^pro^ from naturally occurring viruses that are resistant to nirmatrelvir [52]. For this reason alone, it is important to identify a new generation of 3CL^pro^ inhibitors that can help us defend against current and future coronavirus infections [53, 54]. 3CL^pro^ inhibitors will continue to attract significant interest, as evidenced by the clinical progress of leritrelvir [55], simnotrelvir [56], and ensitrelvir [57]. The XChem facility at Diamond Light source screened over 1250 fragments against the 3CL^pro^ enzyme of SARS‐CoV‐2 using a combination of mass spectrometry and X‐ray. As a result, 74 high‐value fragment hits were found, including 23 non‐covalent and 48 covalent hits in the active site, and 3 hits at the vital dimerization interface [45].

## 2 Results and Discussion

### 2.1 Design and Synthesis

**FIGURE 2:**
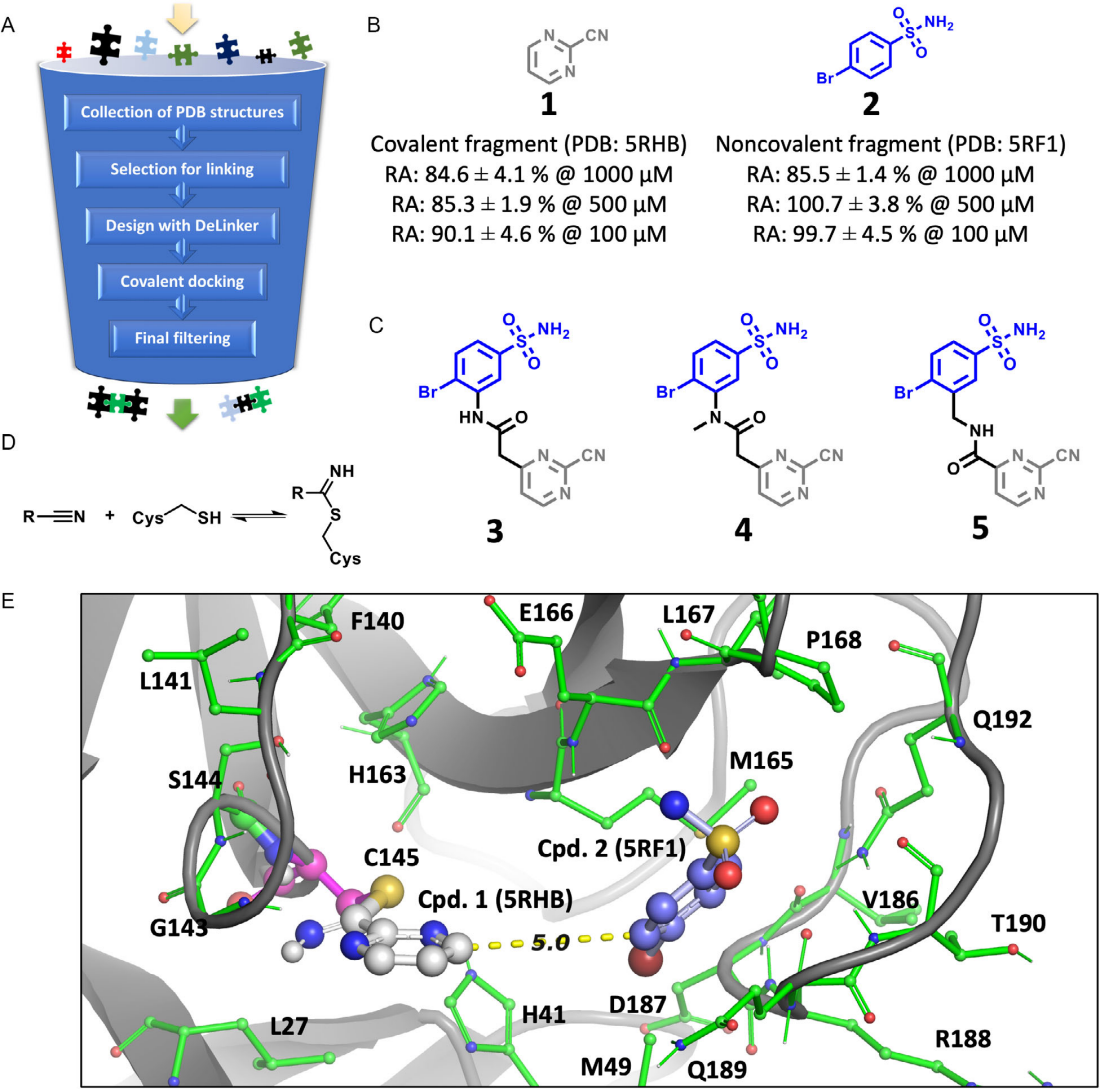
(A) Applied fragment‐based virtual screening workflow. (B) Residual activity (RA) values of the initial fragment hits, linked in the selected compounds. (C) Compounds selected for synthesis. (D) Reversible thioimidate formation in the reaction of a nitrile and a cysteine residue. (E) Structural overlay of the fragments found in PDB structures 5RHB [45] and 5RF1 [45] (protein structure included from 5RHB).

The XChem screening campaign included our Covalent MiniFrag [58] library of five‐ and six‐membered nitrogen heterocycles with various substituents (nitrile, ethynyl, and vinyl groups and halogens) [59]. Two hits were found, pyrimidine‐2‐carbonitrile and imidazole‐2‐carbonitrile (PDB codes: 5RHB and 5RHC, respectively) [45], both of which were able to bind to Cys145 by forming a thioimidate (Figure 2D). With the structural information provided by the X‐ray diffraction study in hand, we started a fragment‐based drug design effort to investigate whether these covalent fragments could be linked to other, non‐covalent fragment hits that were identified.

First, we extracted the non‐covalent fragments from their respective X‐ray structures after structural alignment. Hits that occupy feasible positions for linking with the covalent heterocycles were selected based on various criteria and by visual inspection (Figure S1 and 2). To design linkers between the selected covalent and non‐covalent fragments, DeLinker — a graph‐based deep generative method — was utilized [60]. The main advantage of DeLinker is that it incorporates 3D structural information directly into the design process; therefore, the structural alignment of the linked molecular parts is conserved, or at least the linking is forced to fulfill the positional constraints defined by the X‐ray structures. Also, applying a dedicated tool for linker design ensures an optimal exploration of the available linker space in a more time‐efficient and reproducible way than designing linkers by hand. Next, covalent docking was performed for the linked molecules, and compounds with the best docking scores and RMSD values calculated for the non‐covalent fragment parts were selected for visual inspection and synthetic feasibility assessment (Figure S3). Note that the RMSD of the non‐covalent fragment was selected as the primary evaluation metric, as correct positioning of the non‐covalent core plays an essential role in the appropriate orientation of the warhead toward its binding partner. Therefore, suboptimal binding poses of the non‐covalent core result in unproductive warhead orientations. After exploring the linker space and filtering by fragment pose conservation, candidates for synthesis were selected by an assessment of synthetic feasibility. As such, at the end of the fragment‐based design workflow (Figure 2A), three compounds were chosen for synthesis (Figure 2C). Note that all three compounds consisted of the fragments extracted from the PDB entries 5RHB and 5RF1 (Figure 2E) [45].

Before synthesizing new molecules, the activities of the separate fragment molecules, pyrimidine‐2‐carbonitrile (**1**) and 4‐bromobenzenesulfonamide (**2**), were determined against 3CL^pro^ in vitro at concentrations of 500 and 100 µM with 30 min of pre‐incubation. Both were found practically inactive under the assay conditions (Figure 2B).

**SCHEME 1:**
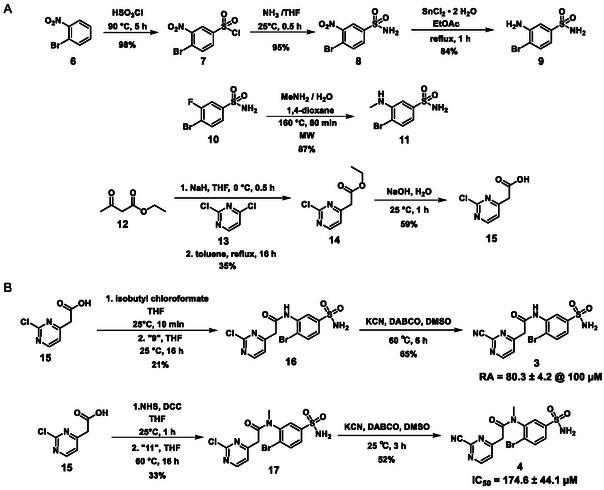
Synthesis of **3** and **4**.

To obtain **3** and **4**, the appropriate building blocks were synthesized first (Scheme 1A). 1‐Bromo‐2‐nitrobenzene (**6**) was treated with chlorosulfonic acid at 90°C for 5 h [61], and the **7** obtained was reacted with ammonia (0.4 M solution in THF) to furnish the sulfonamide **8**. To reduce the nitro group, tin(II) chloride dihydrate was utilized, and the amine (**9**) was obtained in a good yield. To get the *N*‐methylated analog of the latter, a different approach was used. 3‐Fluoro‐4‐bromobenzenesulfonamide (**10**) was reacted with methylamine in a solvent mixture of water and 1,4‐dioxane. The reaction was conducted at 160°C in a microwave reactor to get **11**. To construct the other part of the target molecules, ethyl acetoacetate (**12**) was deprotonated with NaH, and then an aromatic nucleophilic substitution reaction was carried out with 2,4‐dichloropyrimidine (**13**) [62]. Alkaline hydrolysis of the product (**14**) led to the appropriate acid (**15**).

To acylate **9**, carboxylic acid **15** was activated with isobutyl chloroformate, and the reaction resulted in the formation of **16** with a low yield. In the last step, a nucleophilic substitution was done at 60°C with KCN and DABCO was used as a base to get one of the target molecules (**3**). For the *N*‐acylation of **11**, compound **15** was pre‐activated as an NHS‐ester, and the acylation reaction was done at 60°C overnight, yielding the amide (**17**). The last step was again a substitution with KCN to get **4** (Scheme 1B).

**SCHEME 2:**
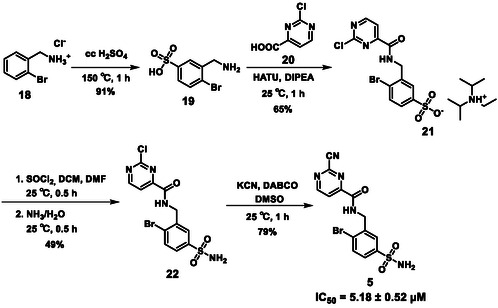
Synthesis of **5**.

The third target molecule was prepared on a different synthetic route (Scheme 2). 2‐Bromobenzylamine hydrochloride (**18**) was sulfonated at 150°C in 96% sulfuric acid to obtain compound **19**, which was purified by crystallization from 1,4‐dioxane, and then reacted with 2‐chloropyrimidine‐4‐carboxylic acid (**20**) using HATU as the coupling agent. Following chromatography, the resulting amide was obtained as a diisopropyl‐ethylammonium salt (**21**). It was first converted to the appropriate sulfonyl chloride, which was not isolated, it was reacted with ammonia(aq) to furnish the respective sulfonamide (**22**). It was then converted to the desired compound (**5**) with a nucleophilic substitution reaction with KCN; the reaction was much faster compared to the preparation of **3** and **4**.

The activity of the linked molecules is highlighted in Schemes 1 and 2, and the results are summarized in Table 1. Compound **3** was found to be inactive, while **4** showed very weak activity. Compound **5** was the most potent analog with an IC_50_ value of 5.18 ± 0.52 µM. Interestingly, linking of **11** with another non‐covalent fragment hit (**23,** 96.0 ± 4.6% residual activity at 100 µM) using the same linker also resulted in an inactive compound (**24,** 89.3 ± 0.5% residual activity at 100 µM, Table 2, Supplementary Note 1).

**TABLE 1:** Summary of the measured biological activities against 3CL^pro^, intact protein MS, and jump dilution assay results.

Compound	3CL^pro^ RA, % or IC_50_, µM	Intact protein MS	Jump dilution assay
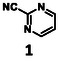	90.1 ± 4.6% @ 100 µM	labeling confirmed	n. d.
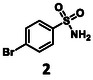	99.7 ± 4.5% @ 100 µM	n. d.	n. d.
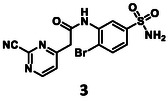	80.3 ± 4.2% @ 100 µM	n. d.	n. d.
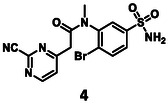	174.6 ± 44.1 µM	n. d.	reversible inhibitor
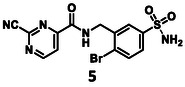	**5.18 ± 0.52 µM**	**labeling confirmed**	**reversible inhibitor**

n. d.: not determined.

**TABLE 2:** Summary of the measured biological activities of compounds obtained in the frame of “classic” SAR exploration.

Compound	3CL^pro^ RA, % or IC_50_, µM	Compound	3CL^pro^ RA, % or IC_50_, µM
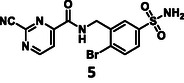 “Core compound“	5.18 ± 0.52 µM	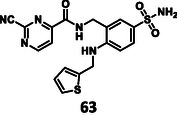	75.9 ± 7.6 μM
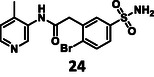	89.3 ± 0.5%@ 100 µM	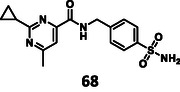	97.9 ± 1.9%@ 100 µM
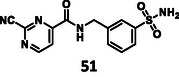	226.3 ± 36.5 μM	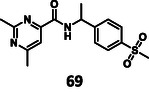	89.3 ± 0.5%@ 100 µM
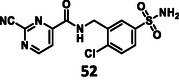	4.26 ± 0.22 μM	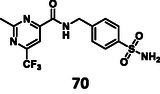	90.4 ± 0.6%@ 100 µM
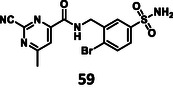	114.2 ± 11.2 μM	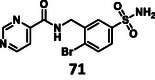	93.0 ± 6.1%@ 100 µM

Although non‐peptidic covalent 3CL^pro^ inhibitors with double‐digit nanomolar IC_50_ are known [63], it is important to note that the optimization leading to these inhibitors has typically been based on a hit of similar or lower potency than **5**. It should be highlighted that the original fragment hits (**1** and **2**) were completely inactive. The compounds with IC_50_s were evaluated in a jump dilution assay, and both of them were found to be reversible inhibitors, just as expected of the pyrimidine‐2‐carbonitrile warhead [64]. When incubated together with 3CL^pro^, **5** covalently labelled the protein, **1** was used as a control (Figures S4 and 5). The results are summarized in Table 1.

### 2.2 Crystal Structure of the **5**‐3CL^Pro^ Complex

**FIGURE 3:**
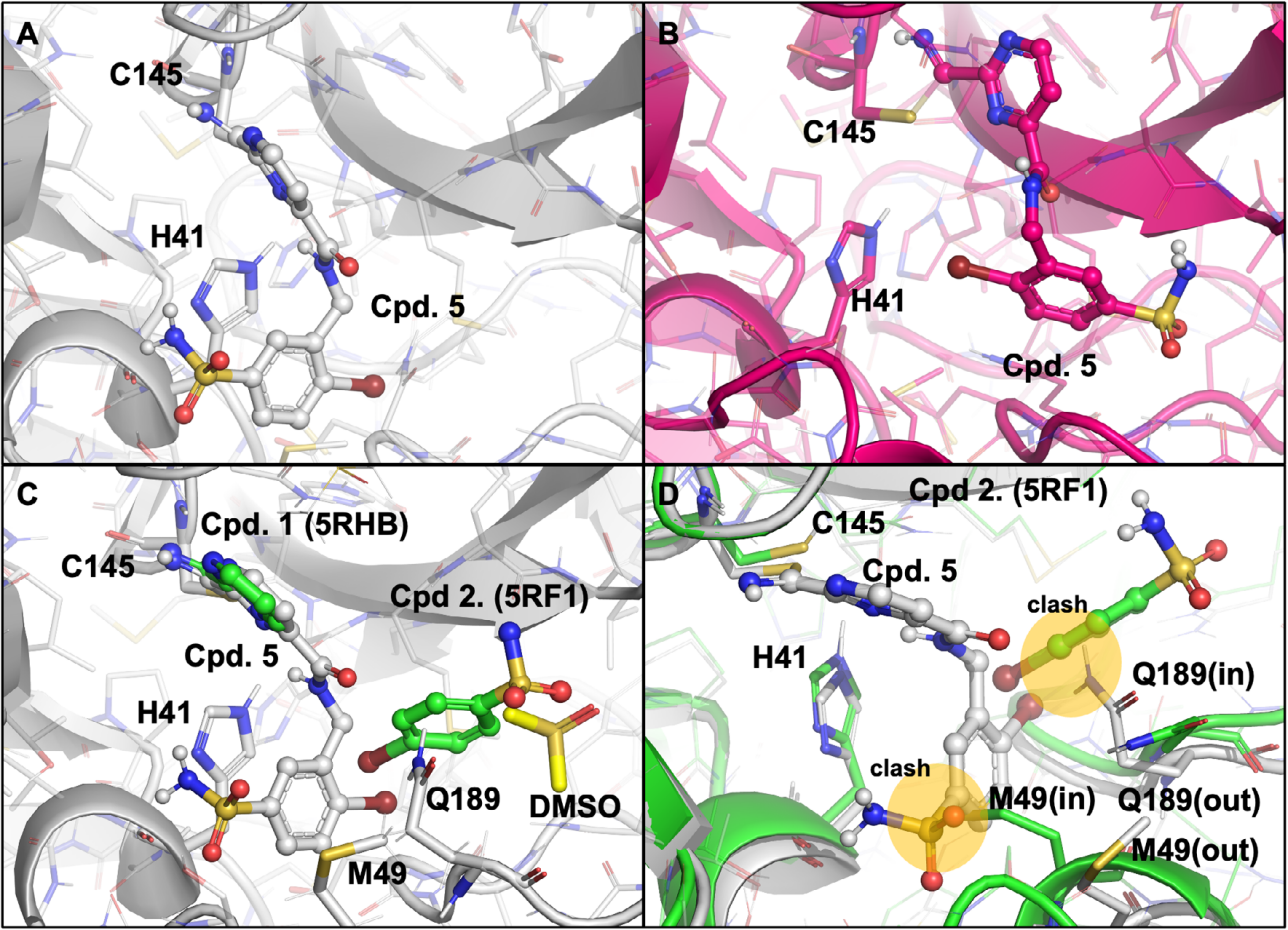
(A) Crystal structure of the **5**‐3CL^pro^ complex. (B) Modeled covalent docking pose of **5.** (C) Superposition of the crystal structure of the **5**(gray)‐3CL^pro^ complex and the two fragments extracted from PDB: 5RHB and 5RF1 (green). (D) Superposition of the **5**‐3CL^pro^ (gray) complex and PDB: 5RF1 (green) structures highlighting the alternative positions of M49 and Q189 (clashes between alternative ligand poses and enzyme environments are highlighted with orange). Throughout the figure, colors correspond to experimental fragment structures (green), experimental structure of cpd. **5** (white) and modeled structures (pink).

With **5** in hand, we received a reversible covalent inhibitor with notable activity compared to the fragment hits. As a follow‐up, we aimed to improve the activity of the core compound by subsequent design attempts. First, the crystal structure of the **5**‐3CL^pro^ complex was acquired (Figure 3A). Interestingly, the moiety adopted from the fragment of 5RF1 (**2**) adopts a “flipped” position compared to its initial pose at the 3CL^pro^ binding site and the previously modeled covalent docking pose of **5** (Figure 3B), i.e., linking the covalent and non‐covalent fragments did not result in a compound adopting the same orientation as its building blocks. However, the initial position of the sulfonamide moiety of the non‐covalent fragment is occupied by a DMSO molecule in the experimental crystal structure, suggesting that the placement of a sulfoxide group is, in fact, still feasible in that position (Figure 3C).

Visual inspection of the linked molecules and fragments’ crystal structure revealed the position of two residues, namely M49 and Q189, as important factors on the relative position of the non‐covalent moiety. The position of the sidechains pointing towards the binding pocket is marked as “in”, the ones that point outside the pocket are denoted as “out” (Figure 3D). With this nomenclature, the M49(in)‐Q189(out) position aids the orientation of the initial 5RF1 fragment and clashes with the orientation found for the crystallographic structure of the non‐covalent moiety of **5**; whereas the M49(out)‐Q189(in) promotes the latter orientation and clashes with the fragment of 5RF1. To test this hypothesis, we have obtained a more recent X‐ray (PDB: 7RNK) [65] structure of 3CL^pro^ from the Fragalysis library [66] that captures the M49(out)‐Q189(in) orientation, and we docked **5** into that particular structure covalently (notably, this sidechain orientation was not captured in any structures of the original fragment screen). The experimental binding pose of the linked molecule was reproduced with an RMSD of 1.74 Å by docking into a M49(out)‐Q189(in) structure (Figure S6) instead of the crystal structure of the covalent fragment, which initially resulted in an RMSD of 7.95 Å. This corroborates our observation that there is a considerable induced‐fit effect during ligand binding that involves sidechain movements of M49 and Q189.

### 2.3 Structure‐Activity Exploration Around the Core Compound **5**

After the exhaustive structural analysis of the experimental crystal structure, we aimed to improve our core compound based on the abovementioned observations and further X‐ray structures extracted from the Fragalysis 3CL^pro^ library. We wondered what kind of modification this structure could tolerate without a loss of activity, and whether we could improve its activity by making substitutions in the mapped regions. According to the crystal structure of **5**, two possible sites of modification were revealed: at the aromatic bromine substituent and the region of the sulfonamide group.

First, we have performed a "classic" SAR exploration of the identified regions; the measured activitIes of derivatives are summarized in Table 2. The compound tolerates the substitution of bromine to chlorine (**52**), but not the removal of the bromine atom (**51**) (Supplementary Note 2). Installing a larger apolar moiety (**63**) or supplementing the pyrimidine ring with a methyl group (**59**) (to accommodate a small cavity of the binding site) was also not tolerated (Supplementary Note 3). Commercial compounds selected and purchased (**68**‐**70**) based on shape similarity to **5** did not yield any further hits, either (Supplementary Note 4).

Next, we investigated the effect of warhead modification. Here, we found that the removal of the warhead (**71**) resulted in a complete loss of activity, highlighting that the secondary interactions between the protein and ligand are not strong enough to achieve measurable potency, but are crucial for warhead positioning during covalent inhibition (Supplementary Note 5). For warhead substitution, we investigated the easily accessible 2‐chloropyrimidine warhead. Since 2‐chloropyrimidine derivatives were key intermediates in all syntheses of the compounds described so far, we investigated whether they react as irreversible covalent warheads [67]. We hypothesized that the electron‐deficient aromatic ring activates the halogen for aromatic nucleophilic substitution that provides a covalent warhead with differences in reactivity, positioning, and labeling mechanism. Here, we identified two derivatives as irreversible covalent inhibitors, but their activities were two orders of magnitude weaker than **5**, due to the suboptimal orientation of the warhead (Supplementary Note 6).

**FIGURE 4:**
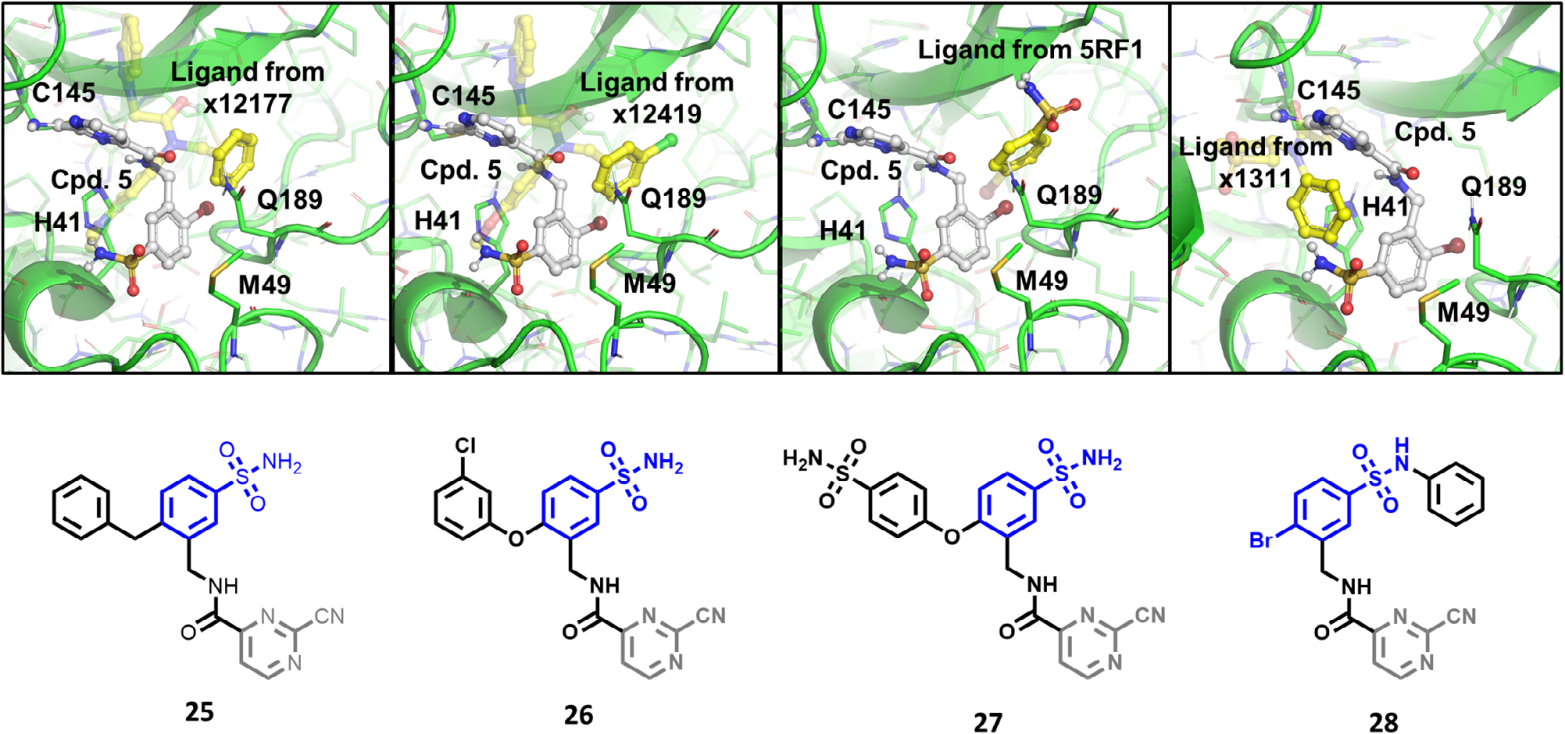
Structure of designed analogs of **5** and the basis of the respective designs where the additional substituents are highlighted in full yellow ball and stick representation (labels refer to PDB IDs, where available, or Fragalysis IDs – see MPro among “Legacy targets” at https://fragalysis.diamond.ac.uk/viewer/react/landing).

Turning once again to our structure‐based design protocol (this time for fragment merging, rather than linking), suitable substituents were selected by clustering, constrained docking and visual inspection of the collected X‐ray ligands. Here, instead of the complete molecules, only those moieties that can be reasonably attached to the core structure were taken into account, in a manner analogous to fragment merging. Three of the newly designed compounds were selected for synthesis (Figure 4).

**SCHEME 3:**
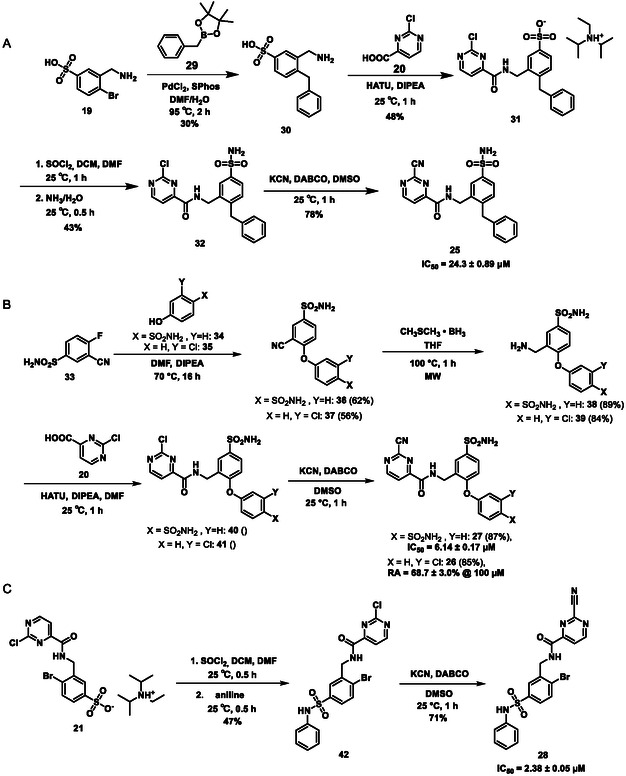
Synthesis of **25** (A), **27**, **26** (B), and **28** (C).

To get **25** (Scheme 3A), the key step was the cross‐coupling of **19** with benzyl boronic acid pinacol ester (**29**), using PdCl_2_ as a catalyst and SPhos as a ligand, which afforded the desired reaction product (**30**). Cross‐coupling was followed by acylation with 2‐chloropyrimidine carboxylic acid (**20**), sulfonamide formation, and nucleophilic substitution with KCN to obtain **25**, similarly to the synthesis of **5**.

A new route was developed for the synthesis of **27** and **26** (Scheme 3B). In the first step, 3‐cyano‐4‐fluorobenzensulfonamide (**33**) was reacted with the appropriate phenol derivatives (**34** and **35**). The S_*N*_Ar reaction needed 70°C and 16 h to get a high conversion. After chromatography, **36** and **37** were obtained with medium yields. Their nitrile groups were reduced using borane dimethyl sulfide complex under MW conditions; the resulting benzylamine derivatives (**38** and **39**) were used immediately in the next reaction. The last two steps were straightforward—acylation with 2‐chloropyrimidine carboxylic acid (**20**) to produce **40** and **41**, and finally nucleophilic substitution with KCN to give the desired nitrile **26** and **27**.

In the synthesis of **28** (Scheme 3C), the previously described intermediate **21** was utilized. Again, it was converted to the sulfonyl chloride using SOCl_2_, then aniline was acylated to get sulfonamide **42**. At last, it was converted with a nucleophilic substitution using KCN to the desired compound (**28**).

The results of the biochemical evaluation are summarized in Table 3. Regarding the designed compounds: if the bromine substituent of **5** (IC_50_ = 5.18 ± 0.52 µM) was replaced with a benzyl group (**25**, IC_50_ = 24.3 ± 0.89 μM), the activity has dropped by an order of magnitude. The incorporation of the *m*‐chlorophenol moiety (**26**, RA = 68.7 ± 3.0% @ 100 µM) led to a significant deterioration in biochemical activity. If a *p*‐H_2_NSO_2_Ph unit was present at this position (**27**, IC_50_ = 6.14 ± 0.17 µM), a bit less active inhibitor was received comparing to the parent compound. Jump dilution assay confirmed its reversible covalent mode of action. However, when the sulfonamide moiety of **5** was modified by the incorporation of a phenyl substituent (**28**, IC_50_ = 2.38 ± 0.05 µM), an improvement in activity was observed. It was also characterized as a reversible covalent inhibitor. Interestingly, if we also consider this molecule as the result of two linked fragments, we can see that they are inactive on their own [**1** (90.1 ± 4.6% @ 100 µM) and **43** (96.7 ± 0.6% @ 100 µM)] while a simple linker between them yields an inhibitor with a low micromolar IC_50_ value in the single digits.

**TABLE 3:** Second round design: summary of the measured biological activities against 3CL^pro^ and jump dilution assay results.

Compound	3CL^pro^ RA, % or IC_50_, µM	Jump dilution assay
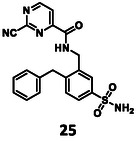	24.3 ± 0.89 μM	n. d.
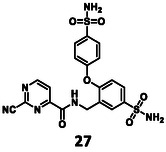	**6.14 ± 0.17 µM**	**reversible inhibitor**
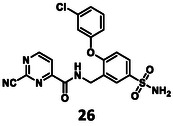	68.7 ± 3.0% @ 100 µM^a^	n. d.
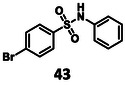	96.7 ± 0.6% @ 100 µM	n. d.
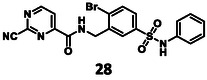	**2.38 ± 0.05 µM**	**reversible inhibitor**

n. d.: not determined.

a : poor‐dose response, IC_50_ could not be determined.

## 3 Conclusion

In this study, we aimed to investigate the feasibility of fragment linking in the optimization of covalent fragment hits obtained with the electrophile‐first strategy. We used 3CL^pro^ as a model system, and subjected fragments previously identified by X‐ray crystallography to fragment linking. We started with fragments **1** and **2,** which are themselves inactive but have been shown to bind to the protein. After designing appropriate linkers, three molecules were synthesized, one of which had a low micromolar activity (**5**) and a reversible, covalent mode of action. However, the linking effort resulted in an unexpected binding mode of this compound, where the non‐covalent fragment adapted a flipped binding pose to accommodate the alternative orientation of two side chains in the binding site. This highlights the necessity of robust structural verification, especially when multiple consecutive rounds of fragment elaboration are performed. It is also interesting to note that the designed molecules (**3**, **4,** and **5**) show a remarkable similarity in structure, with only the linker showing some differences; however, there is a major difference in their biological activities. We attribute this to the general sensitivity of the resulting binding modes to even minor structural changes, especially when one of the fragments is covalent and needs correct warhead positioning. Another factor could be the suboptimal performance of the computational linker design approach (DeLinker), but this was not investigated in detail and could not be reliably concluded without sampling and evaluating a more robust and diverse selection of linkers.

We wanted to assess the robustness of fragment linking, therefore the crystal structure of the **5**‐3CL^pro^ complex was determined and further compounds were designed and synthesized based on the modification of the core compound (**5**) and its experimental binding position. The results highlighted the above‐mentioned vulnerability of the correct binding pose during fragment linking efforts. Maturation of hit compounds derived from covalent fragment linking faces multiple difficulties; structural modifications alter not only the overall binding pose but also the positioning of the warhead towards the targeted sidechain. This, in turn, can exert a significant effect on the energy profile of the covalent reaction. This finding was reinforced during our warhead replacement efforts: while we have managed to identify two 2‐chloropyrimidine derivatives as irreversible covalent inhibitors, their activities were two orders of magnitude weaker than **5**, due to the suboptimal orientation of the warhead.

At the same time, the covalent mode of action was found to be crucial: without a warhead, the molecule (**71**) lost its activity completely. It is important to underline that using the “ligand‐first” approach would not have led to our most active molecule (**5**), as in the absence of activity, we would not have identified the warhead‐free core as a hit.

Finally, we have performed a round of fragment merging based on further fragment positions in the neighboring areas. These have further confirmed the general sensitivity of fragment merging/linking approaches to small structural modifications (cf. significant activity loss for two closely related analogs), but also yielded comparably active 3CL^Pro^ inhibitors, providing further examples for the utility of the electrophile‐first approach, as detailed above.

In summary, we have conducted a fragment‐linking study using the “electrophile‐first” approach for targeted covalent inhibitors. Most importantly, our effort resulted in an inhibitor (**5**) with an IC_50_ value of 5.2 µM by linking practically inactive fragments, which was further optimized using a merging approach to get an inhibitor (**28**) with an IC_50_ value of 2.38 µM. Crucially, early detection of the unexpected binding pose of **5** was necessary to guide further elaboration. Furthermore, our results show the importance of maintaining the correct binding pose for the compound to act as a covalent inhibitor. This is very fragile during fragment linking and optimization, but high‐quality structural information significantly increases the chances of success. Finally, we should note that the in vivo utility of the compounds resulting from fragment‐based approaches (or any other drug discovery effort) is influenced by a number of orthogonal factors (permeability, cytotoxicity, etc.) that were not addressed in detail, in light of the methodological nature of the present study.

## 4 Experimental Section

### 4.1 General Chemistry Methods

Reagents and solvents were purchased from commercial sources (Sigma Aldrich, Enamine, Combi‐Blocks, Fluorochem) and used as received. Flash column chromatography was performed using a CombiFlash Rf 200 instrument (Teledyne ISCO, Lincoln, NE, USA). RediSep Rf reversed‐phase C18 columns (26 g, 43 g, 86 g) were used for reversed‐phase chromatography. Normal‐phase flash column chromatography was performed on Merck Silica Gel 60 (particle size 0.040–0.063 mm; Merck, Germany). Melting points were determined using a Reichelt hot stage apparatus and are uncorrected. ^1^H and ^13^C NMR spectra were recorded at 295 K on a Varian System 500 NMR spectrometer (Varian, Palo Alto, CA, USA) or Varian System 300 NMR spectrometer operating at frequencies for ^1^H NMR at 500 MHz or 300 MHz, and for ^13^C NMR at 126 or 75 MHz, respectively. Chemical shifts (δ) are in parts per million (ppm) and are referenced to the deuterated solvent used. The coupling constants (*J*) are given in Hz, and the splitting patterns are designated as follows: s, singlet; br s, broad singlet; d, doublet; app d, apparent doublet; dd, doublet of doublets; ddd, doublet of doublets of doublets; t, triplet; dt, doublet of triplets; td, triplet of doublets; m, multiplet. All ^13^C NMR spectra were proton decoupled. HPLC‐MS measurements were performed using a Shimadzu LC‐MS‐2020 instrument (Shimadzu Corporation, Kyoto, Japan) equipped with a Reprospher 100 C18 (5 mm, 100 × 3 mm) column and a positive–negative double ion source (DUIS±) with a quadrupole mass spectrometer in the range of 50–1000 m/z. Samples were eluted by gradient elution using eluent A (0.1% HCOOH in H_2_O) and eluent B (0.1% HCOOH in MeCN). The flow rate was set to 1.5 mL/min. The initial condition was 0% B eluent, followed by a linear gradient to 100% B eluent in 2 min, from 2 to 3.75 min, 100% B eluent was maintained, and from 3.75 to 4.5 min, the initial condition was restored and maintained until 5 min. The column temperature was maintained at 30°C, and the injection volume was 1 µL. For purity determination, the PDA detection wavelength was set at 254 nm, unless stated otherwise.

### 4.2 Cloning, Expression, and Purification of Recombinant 3CL^pro^

Codon optimized gene encoding the SARS–CoV‐2 3CL^pro^ gene (Integrated DNA Technologies, USA) was cloned into the pET‐28c(+) plasmid, and used to transform *E. coli* NiCo21(DE3) (New England Biolabs, USA). Transformed bacteria were grown at 37°C and 250 rpm in LB broth supplemented with kanamycin (50 μg/mL) to an OD_600_ ∼1.8. Cultures were cooled on ice for 10 min and expression was induced with isopropyl β‐D‐1‐thiogalactopyranoside – IPTG (200 µM). Expression was continued at 16°C, and 250 rpm for 24 h. Cells were harvested by centrifugation (2 × 10 min, 3000 × *g* at 4°C). Cell pellets were resuspended in buffer A (20 mM Tris, pH 7.5, 0.05 mM EDTA, 2.5 mM DTT, 10% glycerol) and lysed on ice by sonication. Cell debris was removed by centrifugation (2 × 30 min, 16,000 × *g* at 4°C). Clear lysate was filtered through a 100‐kDa MWCO unit (Amicon Ultra‐15 centrifugal filter units; Merck, Germany). (NH_4_)_2_SO_4_ (0.5 M final concentration) was slowly added to the filtrate, which was then loaded onto a 1 mL HiTrap Phenyl HP column (Cytiva, USA) pre‐equilibrated with buffer B (50 mM Tris, pH 7.5, 0.5 M (NH_4_)_2_SO_4_, 0.05 mM EDTA, 2.5 mM DTT, 10% glycerol). The column was washed with 20 volumes of buffer B and the 3CL^pro^ was eluted with a linear gradient into buffer A. The eluted protein was concentrated with a 30‐kDa MWCO unit (Ultra‐4 centrifugal filter units; Amicon), snap‐frozen in liquid nitrogen, and stored at −80°C. Protein concentration was determined by measuring absorbance at 280 nm and considering the extinction coefficient of 34,380 M^−1^ cm^−1^. Purity was determined by SDS‐PAGE.

### 4.3 Enzyme Activity Assay

The enzymatic activity of 3CL^pro^ was measured by kinetic assay using FRET fluorogenic substrate HilyteFluor488‐ESATLQSGLRKAKQXL520 (HilyteFluor488, HPLC peptide purity 93%, Product #: AS‐65 599, Anaspec, USA). Experiments were performed in assay buffer 50 mM Tris‐HCl, pH 7.3, 1 mM EDTA, 0.05% Triton X‐114. For the screening, compounds were pre‐incubated at a concentration of 500 µM and 100 µM with 3CL^pro^ for 30 min at 30°C. The reaction was started by adding substrate, and the increase in fluorescence intensity was measured using Synergy H4 microplate reader (BioTek Instruments, Inc., USA) at λ_ex_ = 485/20 and λ_em_ = 528/20 nm. The final concentrations were as follows: substrate, HilyteFluor488 (2 µM); 3CL^pro^, 50 nM; DMSO, 10% [v/v]. In control experiments, the compound was replaced by DMSO. For the determination of *b* (blank), the enzyme was replaced with Tris‐HCl buffer solution. Initial velocities (*v*) were calculated from the linear trends obtained, with each measurement performed in duplicate. Inhibitory potencies were expressed as residual activities (RA = (*v*_i_ – *b*)/(*v*_*o*_ – *b*), where *v*_i_ is the velocity in the presence of the test compound, and *v*_0_ is the control velocity in the presence of DMSO. At least seven different concentrations were used for IC_50_ determinations for each compound. The IC_50_ values were calculated from experimental data fitted to a four‐parameter logistic function (GraphPad Prism 9.4).

### 4.4 Jump Dilution Assay

For 100‐fold jump dilution assay, compounds were preincubated with 3CL^pro^ (5 µM) for 30 min at the concentration equal to 10× IC_50_ value, and then diluted 100‐fold into buffer containing DABCYL‐EDANS substrate (DABCYL‐EDANS, HPLC peptide purity 97%, Product #: COVD‐001, CPC Scientific, CA, USA, 20 µM). The fluorescence increase was followed using Synergy H4 microplate reader (BioTek Instruments, Inc., USA) at λ_ex_ = 360/40 and λ_em_ = 460/40 nm. Control experiment was carried out in the same manner, and the inhibitor solution was replaced by DMSO, and GC376 was used as a positive control.

### 4.5 Crystallization and Data Collection of 3CL^pro^‐Small Molecule Complexes

Purified protein at a concentration of 5 mg/mL in 10 mM HEPES pH 7.5, 500 mM NaCl, 5% glycerol buffer was used in performing crystallization using sitting‐drop vapor diffusion method using Swissci 3 lens crystallisation plates. The reservoir solution (35 μL added to each reservoir well) used in crystallization contained 11% (w/v) PEG 4000, 5% DMSO, 0.1 M MES, pH 6.7. Crystals of 3CL^pro^ orthorhombic crystal form (P2_1_2_1_2_1_) were grown with drop ratios of 0.15 μL protein, 0.15 μL reservoir solution, and 0.05 μL seeds prepared from previously produced crystals of the same crystal form [68]. Plates were incubated in a Formulatrix Rock Imager (20°C) with the first images taken after 12 h and the imaging schedule following a Fibonacci sequence of days for further collections. Crystal formation was observed in 95% of drops within 24 h and reached their maximum size after 48 h. Further details can be found here [68, 69].

For 3CL^pro^‐small molecule complexes, all experiments were performed at Diamond Light Source XChem facility [70]. Compound was dispensed into crystal drops by an ECHO 550 Liquid Handler (Beckman Coulter) with 17.64 (v/v) % DMSO, with final concentration of 17.64 mM compound soaked into each crystal drop. Thereafter, crystallisation plates were incubated for 1 h at 20°C. Crystals were then harvested using Crystal Shifter [70] (Oxford Lab Technologies) and flash frozen in liquid nitrogen. Diffraction data were collected at Diamond Light Source on the beamline I04−1 at 100 K and processed with the fully automated pipelines at Diamond, including XDS27, Autoproc28, Xia2 29, and DIALS30 [71, 72, 73, 74, 75, 76]. All further analysis was performed using XChemExplorer [77]. Electron density map were generated by Dimple [78] and ligand restrains were generated with GRADE [79]. Model building and refinement were carried out in COOT [80] using Buster [81] via the XChemExplorer platform.

### 4.6 Intact Protein LC‐MS Measurements

The protein stock solution (10.0 µL, 37.6 µM) was diluted with buffer solution (10.0 µL; pH 7.3; 50 mM Tris×HCl, 1 mM EDTA, 0.05% Triton X‐114), then the DMSO stock solution of the inhibitor (0.66 µL, 50 mM; 87‐fold reagent excess) was added. The sample was incubated at 37°C for 90 min, and then subjected to protein LC‐MS measurements. The molecular weights of the conjugates of 3CL^pro^ were identified using a Triple TOF 5600+ hybrid Quadrupole‐TOF LC/MS/MS system (Sciex, Singapore, Woodlands) equipped with a DuoSpray IonSource coupled with a Shimadzu Prominence LC20 UFLC (Shimadzu, Japan) system consisting of binary pump, an autosampler, and a thermostated column compartment. Data acquisition and processing were performed using Analyst TF software version 1.7.1 (AB Sciex Instruments, CA, USA). Chromatographic separation was achieved on a Thermo Beta Basic C8 (50 mm × 3 mm, 3 µm, 150 Å) HPLC column. The sample was eluted in gradient elution mode using solvent A (0.1% formic acid in water) and solvent B (0.1% formic acid in ACN). The initial condition was 15% B for 1 min, followed by a linear gradient to 55% B by 12 min, from 12 to 15 min to 95%, from 15 to 17.5 min B was retained; and from 17.5 to 18 min back to initial condition with 10% eluent B and retained from 18 to 20 min. The flow rate was set to 0.5 ml/min. The column temperature was 40°C, and the injection volume was 10 µl. Nitrogen was used as the nebulizer gas (GS1), heater gas (GS2), and curtain gas with the optimum values set at 40, 40, and 45 (arbitrary units), respectively. Data were acquired in positive electrospray mode in the mass range of m/*z* = 250 to 2500, with 1 s accumulation time. The source temperature was 350°C, and the spray voltage was set to 5000 V. Declustering potential value was set to 80 V. Peak View Software V.2.2 (version 2.2, Sciex, Redwood City, CA, USA) was used for deconvoluting the raw electrospray data to obtain the neutral molecular masses.

### 4.7 Computational Methods

#### 4.7.1 Structure Alignment, Fragment Selection, and Fragment Linking

First, the respective PDB structures, containing non‐covalent and covalent fragments, were collected using Schrödinger's Maestro [82], and protein structures were prepared by the Protein Preparation Wizard [83]. Then, protein structure alignment was carried out based on the amino acid sequence of SARS‐CoV‐2 3CL^pro^. Non‐covalent ligands were extracted, and molecules that overlap with the covalent warheads or bind to another part of the protein were filtered out. The remaining structures were visually inspected, and non‐covalent fragments that occupy a position that is feasible for linking were kept for the next phase. At the end of the selection phase, two covalent and 17 non‐covalent fragments were selected for linking. Based on the feasibility of linking (i.e. suitable distance and orientation), 17 covalent – non‐covalent fragment pairs were created (see Supporting Information) and were linked using DeLinker [60]. Additionally, fragment pairs that contain atoms within bounding distance were merged based on visual inspection. This approach resulted in 20 further directly merged compounds. The design process resulted in a total of 2780 compounds.

#### 4.7.2 Covalent Docking and Compound Filtering

The designed compounds were prepared by LigPrep [84] and docked covalently into the catalytic cysteine of SARS‐CoV‐2 3CL^pro^ using Maestro's Covdock [85]. The previously prepared PDB structure of 5RHB [45] was used for the docking, with the covalent ligand set as the center of the grid and Cys145 selected as the targeted sidechain. The volume of the inner grid was set to 10 × 10 × 10 Å, while the outer box was set to 30 × 30  ×  30 Å. The reaction type was set to “Nucleophilic Addition to a Triple Bond” and the virtual screening precision was used to dock the compounds. Based on the docking score the best 1000 compounds and docking poses were written out. The RMSD of the molecular parts derived from the non‐covalent fragments was evaluated for the docked molecules compared to the original pose of the non‐covalent fragments. Based on the docking scores, RMSD values, visual inspection, and synthetic feasibility, three compounds were selected to be synthesized and tested.

Covalent docking of **5** into the crystal structure of PDB 7RNK [65] possessing M41(out) Q189(in) orientation was carried out similarly as described previously. The protein was pre‐processed with the Protein Preparation Wizard. The crystallographic ligand was selected as the center of the grid, Cys145 was selected as the targeted residue, while the reaction type was set to “Nucleophilic Addition to a Triple Bond” with pose prediction (PP) precision.

#### 4.7.3 Linking of Non‐Covalent Fragments

For the linking of non‐covalent fragments, the previously collected and prepared PDB structures were used. After visual inspection, four fragments were found to be feasible to be linked. Based on their crystallographic positions, four possible fragment pairs were created and linked using DeLinker [60]. Finally, 481 new non‐covalent inhibitors were designed. The molecules created by DeLinker were prepared with LigPrep [84] and were docked with Glide [86] using single precision (SP). The protein structure of 5RHB [45] was used as a docking structure in which the covalent bond was deleted between the ligand and Cys145, Cys145 was present in its neutral form. The restored nitrile derivative was selected as the center of the grid; further parameters were set to their default values. The designed compounds were evaluated by docking scores, RMSD values to their original fragment positions, and synthetic feasibility.

## Author Contributions

**Levente Kollár** synthesized and characterized the compounds, performed protein labeling experiments; **Levente M. Mihalovits** performed the computational work; **József Simon** performed analytical characterization of the synthesized compounds; **Damijan Knez** and **Stanislav Gobec** performed bioactivity measurements; **Blake H. Balcomb** and **Daren Fearon** performed crystallization and X‐ray diffraction measurements; **Dávid Bajusz** and **György M. Keserű** supervised the project. All authors contributed to writing the manuscript.

## Funding

This study was supported by Nemzeti Kutatási Fejlesztési és Innovációs Hivatal (Grants FK146063, SNN135335, 2020‐1.1.2‐PIACI‐KFI‐2020‐00039 and RRF‐2.3.1‐21‐2022‐00015), Hungarian Academy of Sciences (NAP3.0) and Slovenian Research and Innovation Agency ‐ ARIS (grants BI‐HU/21‐22‐003 and P1‐0208).

## Conflicts of Interest

The authors declare no conflicts of interest.

## Supporting information

Supplementary Material

## Data Availability

The crystallographic data referenced in this paper are openly available at the RCSB Protein Data Bank under entries 5RHB, 5RHC, 5RF1, 7RNK and 29OK (for the structure of compound **5** vs. 3CLPro), or at the Fragalysis service of the Diamond Light Source, UK, under Legacy targets/MPro, entries ×1311, ×12177, ×12419. The crystallographic data table of the structure of compound **5** vs. 3CLPro is included in the Supplementary Information (Table S2).
