# Supplementary material for: Fragment‐Based Design of Targeted Covalent Inhibitors: The Scope and Limitation of Linking Approaches
Source: ChemMedChem. 2026 May 22;21(10):e202501108. doi: 10.1002/cmdc.202501108 (PMC13206456; doi:10.1002/cmdc.202501108)
Supplement: Supplementary file 1 — Supplementary Material [file CMDC-21-e202501108-s001.pdf]

SUPPORTING INFORMATION

Fragment-Based Design of Targeted Covalent Inhibitors: The Scope and Limitation of Linking Approaches

Levente Kollár,[1,2,‡] Levente M. Mihalovits,[1,‡] Dávid Bajusz,[1] Damijan Knez,[3] József Simon,[1,4] Blake H. Balcomb,[5,6] Daren Fearon,[5] Stanislav Gobec,[3] György M. Keserű[1,2,*]

[1] *Medicinal Chemistry Research Group and Drug Innovation Centre, Research Centre for Natural Sciences, Magyar tudósok krt. 2, 1117 Budapest, Hungary*

[2] *Department of Organic Chemistry and Technology, Faculty of Chemical Technology and Biotechnology, Budapest University of Technology and Economics, Műegyetem rkp. 3., H-1111 Budapest, Hungary*

[3] *Department of Medicinal Chemistry, Faculty of Pharmacy, University of Ljubljana, Aškerčeva cesta 7, SI-1000, Ljubljana, Slovenia*

[4] *MS Metabolomics Research Group, Research Centre for Natural Sciences, Magyar tudósok krt. 2, 1117 Budapest, Hungary*

[5] *Diamond Light Source, Harwell Science and Innovation Campus, Fermi Ave, Didcot OX11 0DE, UK*

[6] *Research Complex at Harwell, Harwell Science and Innovation Campus, Fermi Ave, Didcot, OX11 0FA, UK*

‡ These authors have contributed equally.

* Corresponding author e-mail: keseru.gyorgy@ttk.hu

Contents of Supporting Information

1. Supplementary Note 1 2

2. Supplementary Note 2 3

3. Supplementary Note 3 4

4. Supplementary Note 4 5

5. Supplementary Note 5 6

6. Supplementary Note 6 6

7. Supplementary Note 7 8

8. Synthetic procedures and characterization of the compounds 10

9. LC-MS chromatograms and spectra, ^{1}H NMR, and ^{13}C NMR spectra of the compounds .. 42

10. Supplementary Figures 141

References 148

1. Supplementary Note 1

In an attempt to perform the linking of two non-covalent fragments in an analogous manner, we linked **11** with another non-covalent fragment hit (**44**) using the same linker, resulting in compound **24**. At the beginning of the synthesis, 3-fluoro-4-bromobenzenesulfonamide (**10**) was equipped with two *p*-methoxyphenyl protecting groups (**45**). Diethyl malonate (**47**) was deprotonated using NaH in 1,4-dioxane, then **45** was added, and the nucleophilic substitution reaction was carried out at 160 ºC in a sealed tube. Next, the *p*-methoxyphenyl protecting groups were cleaved using TFA and anisole in DCM solvent, then the volatiles were evaporated and redissolved in the mixture of acetic acid and cc HCl solution. It was heated to 100 ºC for 1 h, phenylacetic acid derivative (**49**) was received this way. At last, **49** was activated using HATU and utilized to acylate 3-amino-4-methylpyridine (**50**), target compound **24** was obtained (**Scheme S1**). Interestingly, this strategy resulted in an inactive compound (RA = 89.3 ± 0.5% @ 100 µM).

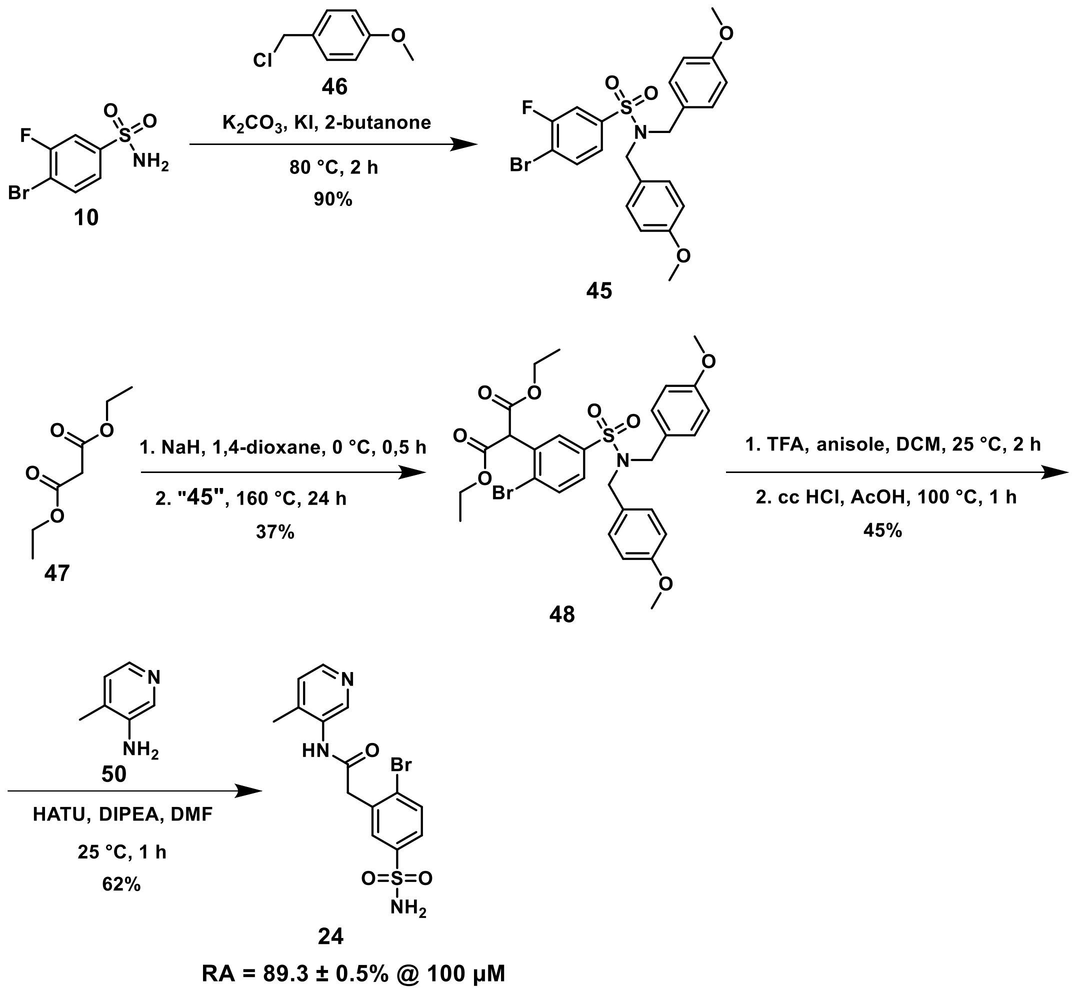

Scheme S1: Synthesis of **24**

2. Supplementary Note 2

To examine the role of the bromine substituent of the core compound **5**, we replaced the bromine atom with hydrogen (**51**) and chlorine (**52**). To synthesize the former, 3-(aminomethyl)benzenesulfonamide hydrochloride (**53**) was acylated with **20**, and the obtained product (**54**) was transformed into **51** using potassium cyanide. The synthetic route that led to **52** was identical to the one that was described for **5**, only the starting material was different (**55**). The activities of these compounds were determined (**Scheme S2**). Compound **52** performed similarly to **5** in protein labelling (**Figure S7**).

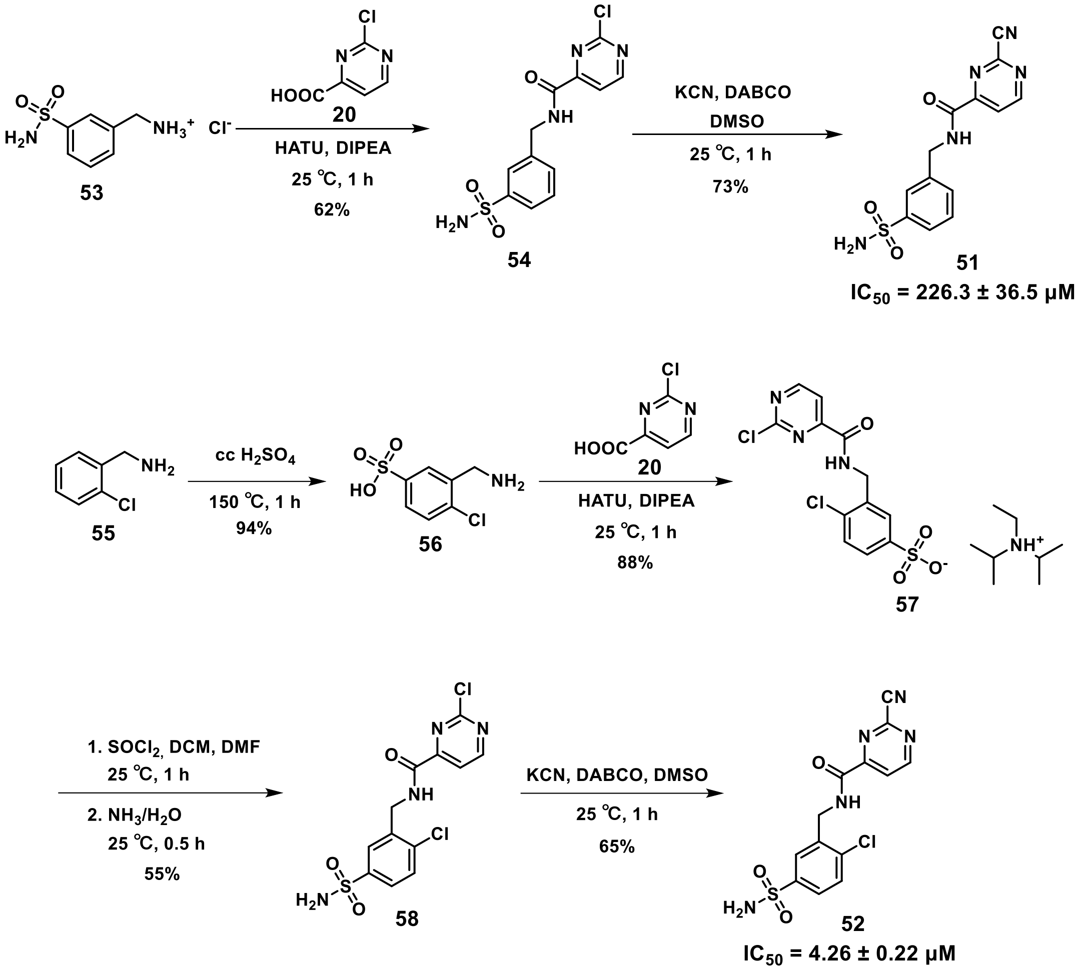

Scheme S2. Synthesis of **51** and **52**

The chlorine analogue (**52**) showed almost the same inhibition of $3CL^{pro}$ as **5**; however, a two orders of magnitude drop was found in the IC_{50} of the non-halogenated compound (**51**). These results suggest that the growing of the molecule is less feasible at the halogen site, in fact, a halogen atom is required in that specific position to retain proper potency.

3. Supplementary Note 3

We have proposed two compounds for further SAR exploration of the bromine position and the pyrimidine ring, syntheses are shown below. **59** is a methyl-derivative of **5**, therefore the synthesis was analogous to the synthesis of **5** – 6-methyl-2-chloropyrimidine-4-carboxylic acid (**60**) was used in the acylation step (**Scheme S3A**). For the synthesis of **63**, 3-cyano-4-fluorobenzensulfonamide (**33**), was reacted with 2-aminomethyltiophene (**64**) to get **65** in an excellent yield. The nitrile group was reduced through catalytic hydrogenation in the presence of Raney nickel in a mixture of methanol and water containing ammonia to yield **66**. The last two steps were straightforward – acylation with 2-chloropyrimidine carboxylic acid (**20**) to

produce **67** and nucleophilic substitution with KCN at last to give the desired nitrile **63** (**Scheme S3B**).

The derivatives of **5** showed no improvement in activity, in fact, all compounds were less potent inhibitors of 3CL^{pro}. Though the binding site was found to be able to accommodate the inserted molecular groups (based on ligand docking), experimental measurements suggest that the initial binding pose is altered for the designed molecules, which in turn results in lower activities.

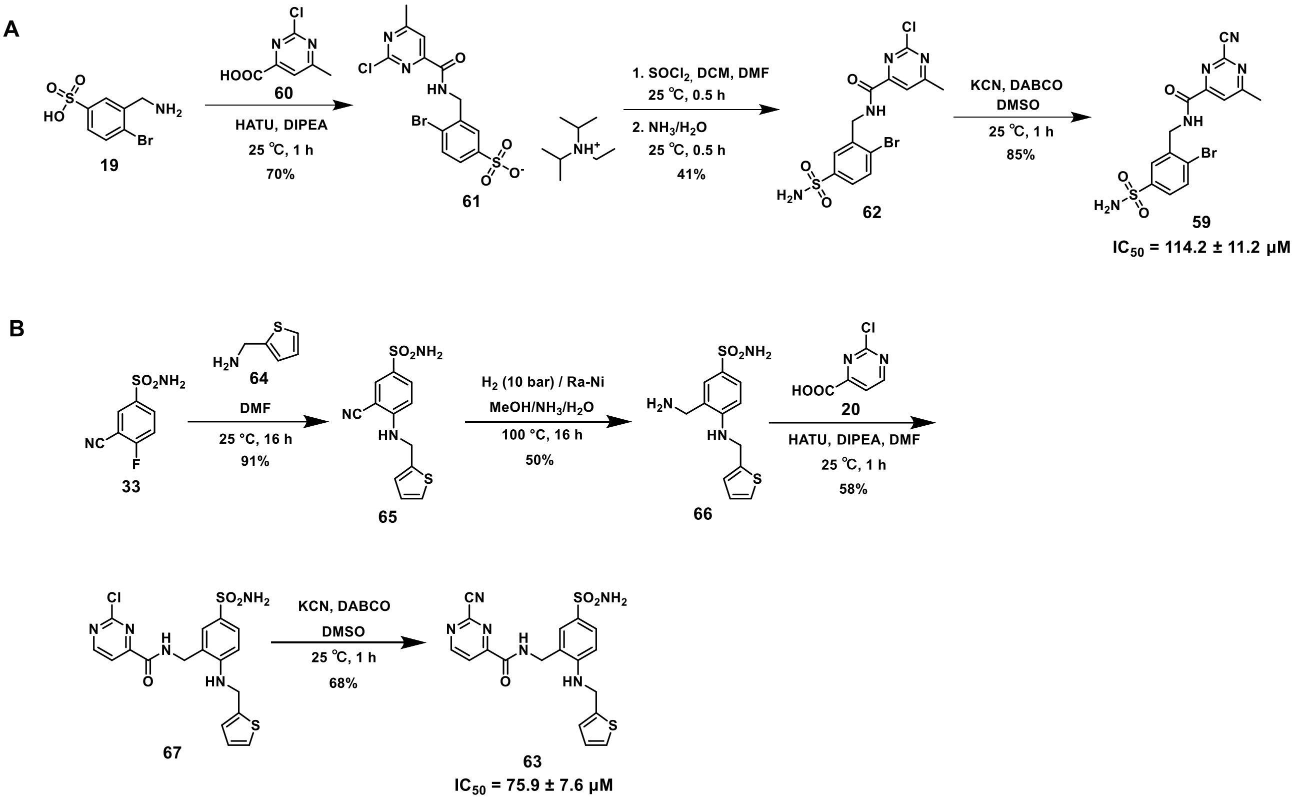

Scheme S3. Synthesis of **59** and **63**

4. Supplementary Note 4

We performed shape complementarity analysis in commercial libraries using the core compound **5** as query. After visual inspection of the available derivatives, we agreed that at least the two aromatic rings (pyrimidine and benzene) and the linker in the right position to connect them should be included in the compounds. Three suitable compounds were purchased and tested against 3CL^{pro}, yet all were inactive (**Scheme S4**). Overall, shape complementarity seems to be insufficient for the inhibition of 3CL^{pro} that further underlines the importance of covalent labelling for this set of inhibitors.

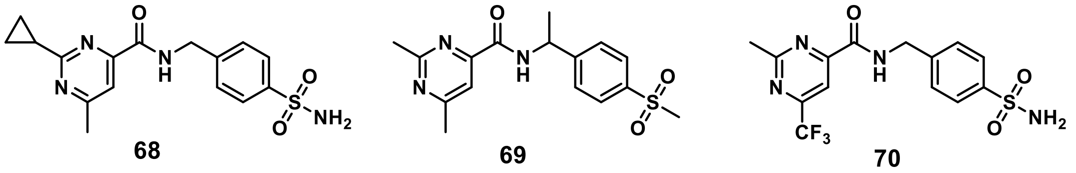

RA = 97.9 ± 1.9% @ 100 μM RA = 89.3 ± 0.5% @ 100 μM RA = 90.4 ± 0.6% @ 100 μM

Scheme S4. Structure and activity of purchased analogues

5. Supplementary Note 5

We aimed to synthesize and assay the non-covalent counterpart of the core compound **5** devoid of the nitrile warhead – **71**. Pyrimidine-4-carboxylic acid (**72**) was utilised to acylate **19**, then the product (**73**) was converted into sulfonamide **71** (**Scheme S5**). Removing the warhead of **5** resulted in a complete loss of activity, highlighting that the secondary interactions between the protein and ligand are not strong enough to achieve measurable potency, but are crucial for warhead positioning during covalent inhibition.

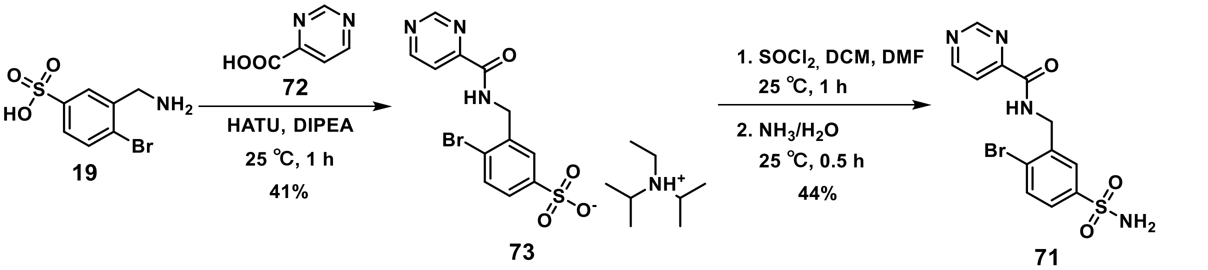

RA = 93.0 ± 6.1% @ 100 μM

Scheme S5. Synthesis of **71**

6. Supplementary Note 6

For warhead substitution, we investigated the easily accessible 2-chloropyrimidine warhead (**Table S1**). All relevant derivatives were evaluated against 3CL^{pro}, and the results are summarised in the table below. Most of them were inactive, but the chloro-derivatives (**22** and **58**) of the most active nitriles (**5** and **52**, respectively) showed some activity. Both were found to be irreversible covalent inhibitors as confirmed by the jump dilution assay results and mass spectrometry (**Figures S8–9**).

Table S1. Summary of the measured biological activities against 3CL^{pro}, intact protein MS, and jump dilution assay results for 2-chloropyrimidine warhead-containing compounds.

Compound	3CL^{pro}	Intact protein MS	Jump dilution assay

	RA [%] or IC_{50} [µM]		
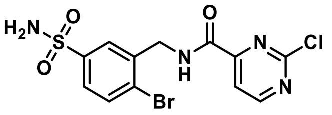 **22**	239.2 ± 34.5 µM	successful labelling	irreversible covalent inhibitor
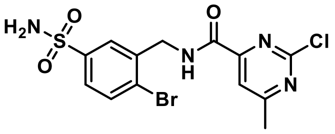 **62**	98.6 ± 0.6% @ 100 µM	n. d.	n. d.
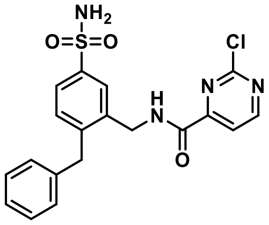 **32**	92.5 ± 3.3% @ 100 µM	n. d.	n. d.
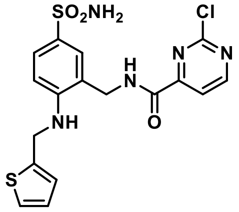 **67**	87.4 ± 5.6 @ 100 µM	n. d.	n. d.
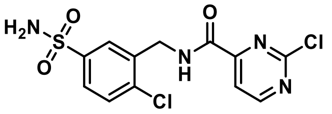 **58**	150.3 ± 24.3 µM	successful labelling	irreversible covalent inhibitor
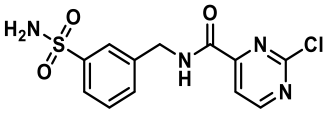 **54**	90.6 ± 3.9 @ 100 µM	n. d.	n. d.
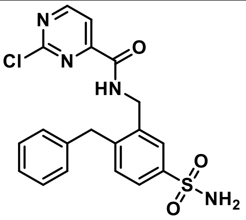 **32**	92.5 ± 3.3% @ 100 µM	n. d.	n. d.
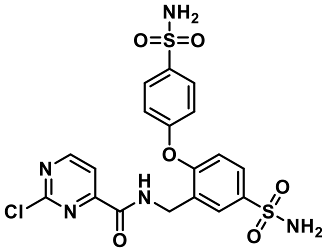 **40**	100.8 ± 1.5% @ 100 µM	n. d.	n. d.

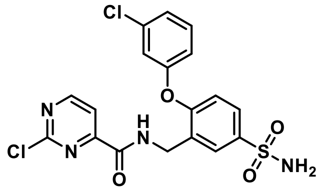 **41**	101.9 ± 0.6% @ 100 µM	n. d.	n. d.
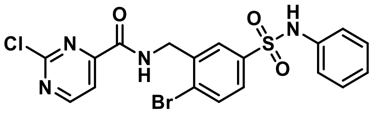 **42**	79.9 ± 1.1% @ 100 µM	n. d.	n. d.

n. d.: not determined

These data suggest that the position of the warhead was suboptimal for the aromatic nucleophilic substitution mechanism of the labelling reaction. This is also underscored by QM/MM umbrella sampling calculations, which revealed a higher barrier for the rate determining step for the nucleophilic substitution reaction of **22** ($\Delta G^{\ddagger}$: 34.8 kcal/mol) than that of the Michael-addition reaction of **5** ($\Delta G^{\ddagger}$: 11.9 kcal/mol) (Supplementary Note 7).

7. Supplementary Note 7

To evaluate the free energy profile of the reaction between Cys145 and **5** and **22** we applied QM/MM based steered molecular dynamics simulations (SMD)[1] coupled with umbrella sampling (US).[2] The experimental crystal structure of the **5**-3CLpro was used to construct the non-covalent complex by deleting the covalent bond between the enzyme and ligand and by transferring the remaining proton from the ligand back to His41. The initial pose of **22** was created by manually modifying the nitrile group to a simple chlorine. Simulation boxes were created with tLeap using the constructed non-covalent complexes with cross-protonated Cys145-His41. The enzyme-ligand complexes were immersed to an octahedral box of TIP3P[3] waters with a spacing of 12 Å and 1.0 closeness parameter. The constructed systems were subjected to a multiple step relaxation protocol applying 500-500 steps of steepest descent and conjugate gradient constrained (applied for the heavy atoms of the protein) minimization, 500-500 steps of unconstrained steepest descent and conjugate gradient minimization, 100 ps of NVT heating to 300 K, 2 ns of NPT equilibration and finally 100 ps of NVT DFTB3[4]/FF14SB[5] QM/MM equilibration. The latter three step were consisted of a positional constraint for the targeted cysteine, histidine, and for the ligands. The relaxation protocol was followed by back-and-forth NVT steered QM/MM MD (SMD) simulations, 50 ps long each. Snapshots from the backwards SMDs were used as starting structures for the subsequent umbrella sampling simulations. Each simulation window was consisted of 40 ps NVT

simulations, the spacing between neighbouring windows was 0.1 Å along the reaction coordinate. The reaction coordinate values during the simulations were collected and used to create the potential of mean force curves applying the weighted histogram analysis method (WHAM).[6,7] Biases during the SMD and US simulations were handled with the PLUMED[8] extension for AMBER applying a 300 kcal/mol force constant to the biasing potential. Temperature and pressure regulation were carried out using the Langevin thermostat and Berendsen barostat, respectively. The applied reaction coordinates, the modelled reactions, highlighted states (reactants, transition state, product) and constructed PMFs are shown on **Figure S10**.

8. Synthetic procedures and characterization of the compounds

Pyrimidine-2-carbonitrile (1)

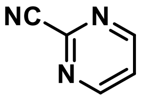

Pyrimidine-2-carbonitrile (**1**) is prone to form 2,4,6-tris(2-pyrimidyl)-1,3,5-triazine during storage. Before biochemical evaluation, it was purified by reversed-phase flash column chromatography using eluents A (0.1% HCOOH in MeCN) and B (0.1% HCOOH in H_2O) (gradient from 5:95 to 30:70). The product pyrimidine-2-carbonitrile (**1**) was obtained as a white solid. Analytical data was identical to those reported previously.[9] Mp.: 38–40 °C; ^{1}H NMR (500 MHz, DMSO-*d*$_6$) δ 9.02 (d, J = 5.0 Hz, 2H), 7.87 (t, J = 5.0 Hz, 1H); ^{13}C NMR (126 MHz, DMSO-*d*$_6$) δ 158.77, 144.00, 124.81, 115.97; HRMS (ESI$^+$) *m/z* [M+H]$^+$, calcd. for $C_5H_4N_3$: 106.0405, found: 106.0410; Purity by HPLC: 100%.

4-Bromobenzene-1-sulfonamide (2)

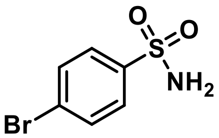

4-Bromobenzene-1-sulfonamide (**2**) was purchased from a commercial source, and the purity of the compound was checked before biochemical evaluation. Analytical data was identical to those reported previously.[10] Mp.: 159–160 °C; ^{1}H NMR (500 MHz, DMSO-*d*$_6$) δ 7.81 – 7.74 (m, 4H), 7.45 (s, 2H); ^{13}C NMR (126 MHz, DMSO-*d*$_6$) δ 143.39, 131.97, 127.70, 125.38; HRMS (ESI$^-$) *m/z* [M–H]$^-$, calcd. for $C_6H_5NO_2SBr$: 233.9224, found: 233.9220; Purity by HPLC: 100%.

N-(2-Bromo-5-sulfamoylphenyl)-2-(2-cyanopyrimidin-4-yl)acetamide (3)

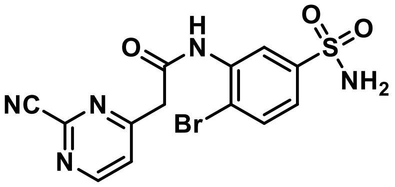

To a solution of *N*-(2-bromo-5-sulfamoylphenyl)-2-(2-chloropyrimidin-4-yl)acetamide (**16**, 16 mg, 0.04 mmol, 1.0 equiv.) in DMSO (1.4 mL), DABCO (26 mg, 0.24 mmol, 6.0 equiv.) and KCN (7 mg, 0.112 mmol, 2.8 equiv.) were added and the mixture was allowed to stir at 60 °C for 6 h. The solution was subjected to reversed-phase flash column chromatography using

eluents A (0.1% HCOOH in MeCN) and B (0.1% HCOOH in H_2O) (gradient from 10:90 to 50:50). The product *N*-(2-bromo-5-sulfamoylphenyl)-2-(2-cyanopyrimidin-4-yl)acetamide (**3**) was obtained as a white solid (10 mg, 65% yield). Mp.: 151–153 °C; ^{1}H NMR (300 MHz, CD_3CN) δ 9.06 (s, 1H), 8.84 (d, *J* = 5.2 Hz, 1H), 8.61 (d, *J* = 2.1 Hz, 1H), 8.29 (s, 1H), 7.82 – 7.77 (m, 1H), 7.74 (d, *J* = 5.2 Hz, 1H), 7.55 – 7.49 (m, 1H), 4.08 (s, 2H); ^{13}C NMR (75 MHz, CD_3CN) δ 167.26, 166.59, 159.62, 145.23, 144.28, 137.57, 134.45, 125.79, 124.01, 121.49, 119.49, 116.90, 45.24; HRMS (ESI$^-$) *m/z* [M–H]$^-$, calcd. for $C_{13}H_9N_5O_3SBr$: 393.9609, found: 393.9615; Purity by HPLC: 98.7%.

***N*-(2-Bromo-5-sulfamoylphenyl)-2-(2-cyanopyrimidin-4-yl)-*N*-methylacetamide (4)**

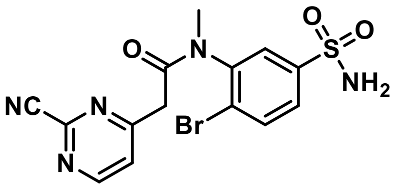

To a solution of *N*-(2-bromo-5-sulfamoylphenyl)-2-(2-chloropyrimidin-4-yl)-*N*-methylacetamide (**17**, 18 mg, 0.04 mmol, 1.0 equiv.) in DMSO (1.6 mL), DABCO (26 mg, 0.24 mmol, 6.0 equiv.) and KCN (7 mg, 0.112 mmol, 2.8 equiv.) were added and the mixture was allowed to stir at 25 °C for 3 h. The solution was subjected to reversed-phase flash column chromatography using eluents A (0.1% HCOOH in MeCN) and B (0.1% HCOOH in H_2O) (gradient from 10:90 to 50:50). The product *N*-(2-bromo-5-sulfamoylphenyl)-2-(2-cyanopyrimidin-4-yl)-*N*-methylacetamide (**4**) was obtained as a white solid (9 mg, 52% yield). Mp.: 174–175 °C; ^{1}H NMR (500 MHz, DMSO-d_6) δ 8.88 (s, 1H), 8.03 (d, J = 6.8 Hz, 1H), 7.98 (s, 1H), 7.80 (s, 1H), 7.65 (s, 1H), 7.53 (s, 2H), 3.70 (d, J = 17.4 Hz, 1H), 3.52 (d, J = 16.2 Hz, 1H), 3.13 (d, J = 1.8 Hz, 3H); ^{13}C NMR (126 MHz, DMSO-d_6) δ 166.98, 165.93, 158.37, 145.18, 143.52, 141.67, 134.72, 127.75, 127.53, 126.84, 125.26, 115.90, 42.57, 35.68;
HRMS (ESI$^-$) *m/z* [M–H]$^-$, calcd. for $C_{14}H_{11}N_5O_3SBr$: 407.9765, found: 407.9780; Purity by HPLC: 99.8%.

N-[(2-Bromo-5-sulfamoylphenyl)methyl]-2-cyanopyrimidine-4-carboxamide (5)

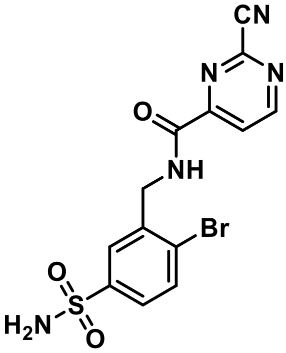

To a solution of *N*-[(2-bromo-5-sulfamoylphenyl)methyl]-2-chloropyrimidine-4-carboxamide (**22**, 16 mg, 0.04 mmol, 1.0 equiv.) in DMSO (1.0 mL), DABCO (13 mg, 0.12 mmol, 3.0 equiv.) and KCN (4 mg, 0.056 mmol, 1.4 equiv.) were added and the mixture was allowed to stir at 25 °C for 1 h. The solution was subjected to reversed-phase flash column chromatography using eluents A (0.1% HCOOH in MeCN) and B (0.1% HCOOH in H_2O) (gradient from 10:90 to 80:20). The product *N*-[(2-bromo-5-sulfamoylphenyl)methyl]-2-cyanopyrimidine-4-carboxamide (**5**) was obtained as a white solid (12 mg, 79% yield). Mp.: 197–198 °C; ^{1}H NMR (500 MHz, DMSO-d_6) δ 9.88 (t, J = 6.0 Hz, 1H), 9.28 (d, J = 5.1 Hz, 1H), 8.32 (d, J = 5.1 Hz, 1H), 7.86 (d, J = 8.3 Hz, 1H), 7.74 (s, 1H), 7.65 (d, J = 8.2 Hz, 1H), 7.44 (s, 2H), 4.60 (d, J = 6.0 Hz, 2H); ^{13}C NMR (126 MHz, DMSO-d_6) δ 161.67, 161.40, 157.46, 143.69, 143.21, 138.25, 133.11, 125.95, 125.66, 125.23, 121.75, 115.78, 43.16; HRMS (ESI$^-$) *m/z* [M–H]$^-$, calcd. for $C_{13}H_9N_5O_3SBr$: 393.9609, found: 393.9594; Purity by HPLC: 99.4%.

4-Bromo-3-nitrobenzene-1-sulfonyl chloride (7)

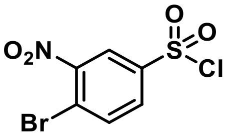

4-Bromo-3-nitrobenzene-1-sulfonyl chloride (**7**) was prepared based on a literature method.[11] A round bottom flask containing chloro sulfonic acid (10 mL) was cooled to 0 °C to which l-bromo-2-nitrobenzene (**6**, 2020 mg, 10.0 mmol) was added in portions. The reaction mixture was then heated to 90 °C for 5 h and poured slowly into crushed ice. The precipitate was filtered, washed with distilled water (10 × 30 mL), and allowed to air dry. The product 4-bromo-3-nitrobenzene-1-sulfonyl chloride (**7**) was obtained as a brown solid (2930 mg, 98% yield). Mp.: 52–54 °C; ^{1}H NMR (500 MHz, DMSO-d_6) δ 8.07 (d, J = 1.9 Hz, 1H), 7.88 (d, J = 8.2 Hz, 1H), 7.75 (dd, J = 8.3, 2.0 Hz, 1H); ^{13}C NMR (126 MHz, DMSO-d_6) δ 149.18, 149.09, 134.87,

130.78, 122.54, 113.38; HRMS (ESI$^-$) *m/z* [M–H]$^-$, calcd. for $C_6H_3NO_5SBr$: 279.8915, found: 279.8906 (detected as the corresponding sulfonic acid derivative); Purity by HPLC: 100%.

4-Bromo-3-nitrobenzene-1-sulfonamide (8)

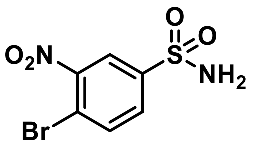

A round bottom flask was charged with ammonia solution (40 mL, 0.4 M in THF), 4-bromo-3-nitrobenzene-1-sulfonyl chloride (**7**, 2705 mg) was added in portions. The reaction was allowed to proceed at 25 °C for 0.5 h. The mixture was concentrated; the residue was redissolved in EtOAc (40 mL) and extracted with water (20 mL) and brine (20 mL). The organic layer was dried with Na_2SO_4, filtered, and the volatiles were removed under reduced pressure. The product 4-bromo-3-nitrobenzene-1-sulfonamide (**8**) was obtained as a brown solid (2662 mg, 95% yield). Mp.: 158–160 °C; ^{1}H NMR (500 MHz, DMSO-*d*$_6$) δ 8.40 (d, J = 2.1 Hz, 1H), 8.19 – 8.14 (m, 1H), 7.98 (dd, J = 8.4, 2.1 Hz, 1H), 7.73 (s, 2H); ^{13}C NMR (126 MHz, DMSO-*d*$_6$) δ 149.37, 144.70, 136.00, 130.42, 122.77, 117.08; HRMS (ESI$^-$) *m/z* [M–H]$^-$, calcd. for $C_6H_4N_2O_4SBr$: 278.9075, found: 278.9065; Purity by HPLC: 98.7%.

3-Amino-4-bromobenzene-1-sulfonamide (9)

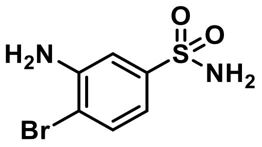

To a solution of 4-bromo-3-nitrobenzene-1-sulfonamide (**8**, 562 mg, 2.00 mmol, 1.0 equiv.) in EtOAc (40 mL), $SnCl_2 \times 2\ H_2O$ (2708 mg, 12.00 mmol, 6.0 equiv.) was added and the reaction was allowed to reflux for 1 h. It was then diluted with EtOAc (120 mL) and washed with saturated aqueous solution of $NaHCO_3$ (2 × 120 mL) and brine (60 mL). The organic layer was dried with Na_2SO_4, filtered, and the volatiles were removed under reduced pressure. The product 3-amino-4-bromobenzene-1-sulfonamide (**9**) was obtained as a light brown solid (423 mg, 84% yield). Mp.: 137– 138 °C; ^{1}H NMR (300 MHz, DMSO-*d*$_6$) δ 7.52 (d, J = 8.3 Hz, 1H), 7.26 (d, J = 5.1 Hz, 2H), 7.24 (d, J = 2.2 Hz, 1H), 6.88 (dd, J = 8.3, 2.2 Hz, 1H), 5.76 (s, 2H); ^{13}C NMR (75 MHz, DMSO-*d*$_6$) δ 146.09, 144.14, 132.70, 113.82, 111.84, 110.12; HRMS (ESI$^-$) *m/z* [M–H]$^-$, calcd. for $C_6H_6N_2O_2SBr$: 248.9333, found: 248.9326; Purity by HPLC: 99.8%.

4-Bromo-3-(methylamino)benzene-1-sulfonamide (11)

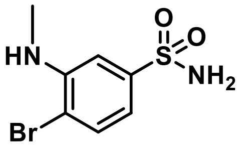

To a solution of 3-fluoro-4-bromobenzenesulfonamide (**10**, 356 mg, 1.40 mmol) in 1,4-dioxane (3.2 mL), methylamine solution (40% in H_2O, 6.0 mL) was added. The mixture was allowed to stir at 160 °C for 80 min in a microwave reactor. The volatiles were removed under reduced pressure, the residue was triturated with water (10 ml) and it was filtered. The solid was washed with H_2O (2 × 15 mL) and allowed to air dry. The product 4-bromo-3-(methylamino)benzene-1-sulfonamide (**11**) was obtained as a beige coloured solid (322 mg, 87% yield). Mp.: 150–151 °C; ^{1}H NMR (500 MHz, DMSO-*d$_6$*) δ 7.58 (d, J = 8.2 Hz, 1H), 7.29 (s, 2H), 7.00 (d, J = 2.1 Hz, 1H), 6.95 – 6.92 (m, 1H), 5.75 (d, J = 4.8 Hz, 1H), 2.80 (d, J = 4.8 Hz, 3H); ^{13}C NMR (126 MHz, DMSO-*d$_6$*) δ 146.33, 144.57, 132.36, 113.35, 111.24, 107.01, 29.99; HRMS (ESI$^-$) *m/z* [M–H]$^-$, calcd. for $C_7H_8N_2O_2SBr$: 262.9489, found: 262.9481; Purity by HPLC: 97.6%.

Ethyl 2-(2-chloropyrimidin-4-yl)acetate (14)

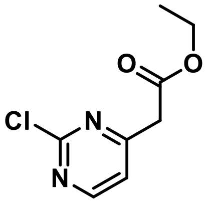

Ethyl 2-(2-chloropyrimidin-4-yl)acetate (**14**) was prepared based on a literature method.[12] A round bottom flask containing THF (100 mL) was cooled to 0 °C, then NaH (60% suspension in paraffin oil, 864 mg, 36.0 mmol, 1.80 equiv.) was added. Ethyl acetoacetate (**12**, 4.56 mL, 36.0 mmol, 1.80 equiv.) was added dropwise and the mixture was allowed to stir at 0 °C for 0.5 h. The volatiles were removed under reduced pressure; the residue was suspended in toluene (120 mL). 2,4-Dichloropyrimidine (**13**, 2979 mg, 20.0 mmol, 1.0 equiv.) was added and the mixture was allowed to reflux for 16 h. The volatiles were removed under reduced pressure, the residue was redissolved in EtOAc (200 mL) and extracted with water (100 mL) and brine (50 mL). The organic layer was concentrated, and the crude residue was purified by reversed-phase flash column chromatography using eluents A (0.1% HCOOH in MeCN) and B (0.1% HCOOH in H_2O) (gradient from 10:90 to 80:20). The product ethyl 2-(2-chloropyrimidin-4-yl)acetate (**14**) was obtained as an orange coloured oil (1416 mg, 35% yield). ^{1}H NMR (500 MHz, DMSO-*d$_6$*) δ 8.74 (d, J = 5.1 Hz, 1H), 7.59 (d, J = 5.1 Hz, 1H), 4.12 (q, J = 7.1 Hz, 2H), 3.93 (s, 2H), 1.18 (t, J = 7.2 Hz, 3H); ^{13}C NMR (126 MHz, DMSO-*d$_6$*) δ 168.68, 166.94, 160.50,

159.76, 120.97, 60.83, 42.08, 13.93; HRMS (ESI$^+$) *m/z* [M+H]$^+$, calcd. for $C_8H_{10}N_2O_2Cl$: 201.0430, found: 201.0436; Purity by HPLC: 98.2%.

2-(2-Chloropyrimidin-4-yl)acetic acid (15)

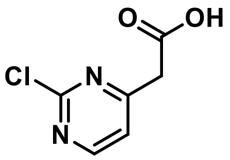

To a suspension of ethyl 2-(2-chloropyrimidin-4-yl)acetate (**14**, 1091 mg, 5.44 mmol, 1.0 equiv.) in H_2O (10 mL), NaOH (218 mg, 5.44 mmol, 1.0 equiv.) was added. The reaction was allowed to proceed at 25 °C for 1 h. A yellow solution was obtained. The pH of the solution was modified to 1-2 with 5% HCl. It was extracted with EtOAc (6 × 50 mL). The merged organic layers were dried with Na_2SO_4, filtered, and the volatiles were removed under reduced pressure. The product 2-(2-chloropyrimidin-4-yl)acetic acid (**15**) was obtained as a yellow solid (554 mg, 59% yield). Mp.: 31–32 °C; ^{1}H NMR (300 MHz, DMSO-*d$_6$*) δ 8.61 (d, J = 5.0 Hz, 1H), 7.46 (d, J = 5.0 Hz, 1H), 2.48 (s, 2H); ^{13}C NMR (75 MHz, DMSO-*d$_6$*) δ 171.11, 159.82, 159.76, 159.25, 120.29, 23.39; HRMS (ESI$^+$) *m/z* [M+H]$^+$, calcd. for $C_6H_6N_2O_2Cl$: 173.0117, found: 173.0118; Purity by HPLC: 90.1%.

***N*-(2-Bromo-5-sulfamoylphenyl)-2-(2-chloropyrimidin-4-yl)acetamide (16)**

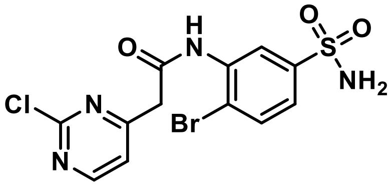

To a solution of 2-(2-chloropyrimidin-4-yl)acetic acid (**15**, 69 mg, 0.40 mmol, 1.0 equiv.) in THF (4.0 mL), TEA (56 µL, 0.40 mmol, 1.0 equiv.) and isobutyl chloroformate (52 µL, 0.40 mmol, 1.0 equiv.) were added. The mixture was allowed to stir for 10 min, then 3-amino-4-bromobenzene-1-sulfonamide (**9**, 100 mg, 0.40 mmol, 1.0 equiv.) was added and the mixture was stirred at 25 °C for 16 h. The solution was purified by reversed-phase flash column chromatography using eluents A (0.1% HCOOH in MeCN) and B (0.1% HCOOH in H_2O) (gradient from 10:90 to 60:40). The product *N*-(2-bromo-5-sulfamoylphenyl)-2-(2-chloropyrimidin-4-yl)acetamide (**16**) was obtained as a light yellow solid (34 mg, 21% yield). Mp.: 158–159 °C; ^{1}H NMR (300 MHz, DMSO-*d$_6$*) δ 10.10 (s, 1H), 8.75 (d, J = 4.4 Hz, 1H), 8.12 (s, 1H), 7.89 (d, J = 8.4 Hz, 1H), 7.64 (d, J = 4.4 Hz, 1H), 7.50 (s, 2H), 7.25 (d, J = 9.9 Hz, 1H), 4.06 (s, 2H); ^{13}C NMR (126 MHz, DMSO-*d$_6$*) δ 166.84, 160.30, 146.05, 143.88, 133.45,

132.65, 123.87, 123.45, 121.01, 113.81, 111.82, 44.02; HRMS (ESI$^-$) *m/z* [M–H]$^-$, calcd. for $C_{12}H_9N_4O_3SClBr$: 402.9267, found: 402.9285; Purity by HPLC: 97.5%.

***N*-(2-Bromo-5-sulfamoylphenyl)-2-(2-chloropyrimidin-4-yl)-*N*-methylacetamide (17)**

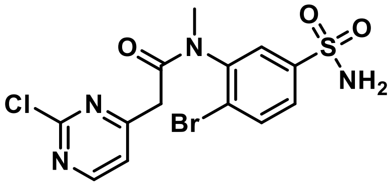

To a solution of 2-(2-chloropyrimidin-4-yl)acetic acid (**15**, 173 mg, 1.00 mmol, 2.0 equiv.) in THF (10.0 mL), NHS (123 mg, 1.00 mmol, 2.0 equiv.) and DCC (227 mg, 2.00 mmol, 1.0 equiv.) were added and the mixture was allowed to stir at 25 °C for 1 h. It was filtered, and washed with THF (5.0 mL). To the solution containing the appropriate NHS ester, 4-bromo-3-(methylamino)benzene-1-sulfonamide (**11**, 133 mg, 0.50 mmol, 1.0 equiv.) was added and the mixture was allowed to stir at 60 °C for 16 h. The solution was subjected to reversed-phase flash column chromatography using eluents A (0.1% HCOOH in MeCN) and B (0.1% HCOOH in H_2O) (gradient from 10:90 to 50:50). The product *N*-(2-bromo-5-sulfamoylphenyl)-2-(2-chloropyrimidin-4-yl)-*N*-methylacetamide (**17**) was obtained as a white solid (69 mg, 33% yield). Mp.: 141–142 °C; ^{1}H NMR (500 MHz, DMSO-*d*$_6$) δ 8.65 (d, J = 5.0 Hz, 1H), 8.03 (d, J = 8.4 Hz, 1H), 7.99 (d, J = 2.0 Hz, 1H), 7.79 (dd, J = 8.4, 2.0 Hz, 1H), 7.53 (s, 2H), 7.37 (d, J = 5.0 Hz, 1H), 3.62 (d, J = 15.9 Hz, 1H), 3.44 (d, J = 15.9 Hz, 1H), 3.13 (s, 3H); ^{13}C NMR (126 MHz, DMSO-*d*$_6$) δ 167.51, 167.04, 159.92, 159.59, 145.14, 141.70, 134.61, 127.65, 127.43, 126.77, 121.06, 42.46, 35.61; HRMS (ESI$^-$) *m/z* [M–H]$^-$, calcd. for $C_{13}H_{11}N_4O_3SClBr$: 416.9423, found: 416.9422; Purity by HPLC: 98.2%.

3-(Aminomethyl)-4-bromobenzene-1-sulfonic acid (19)

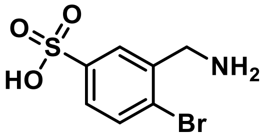

2-Bromobenzylamine hydrochloride (**18**, 1001 mg, 4.50 mmol) was added portion-wise to concentrated sulfuric acid (96%, 6.0 mL). The solution was heated to 150 °C and the reaction was allowed to proceed for 1 h. It was poured into 1,4-dioxane (60 mL). After 1 h, the precipitated solid was filtered and it was washed with 1,4-dioxane (2 × 50 mL), and allowed to air dry. The product 3-(aminomethyl)-4-bromobenzene-1-sulfonic acid (**19**) was obtained as a white solid (1085 mg, 91% yield). Mp.: 155–156 °C; ^{1}H NMR (500 MHz, DMSO-*d*$_6$ + D_2O) δ

7.77 (s, 1H), 7.71 (d, J = 8.3 Hz, 1H), 7.57 (dd, J = 8.2, 1.5 Hz, 1H), 4.13 (s, 2H); ^{13}C NMR (126 MHz, DMSO-d_6 + D_2O) δ 146.76, 134.05, 133.49, 128.89, 128.41, 125.99, 43.12; HRMS (ESI$^-$) *m/z* [M–H]$^-$, calcd. for $C_7H_7NO_3SBr$: 263.9330, found: 263.9329; Purity by HPLC: 100% (integration at wavelength 190 nm).

Ethylbis(propan-2-yl)azanium 4-bromo-3-[[(2-chloropyrimidin-4yl)formamido]methyl]benzene-1-sulfonate (21)

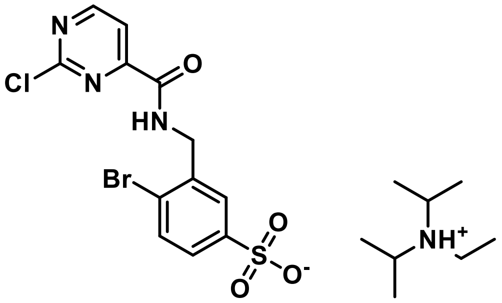

To a stirred solution of 2-chloropyrimidine-4-carboxylic acid (**20**, 38 mg, 0.24 mmol, 1.2 equiv.), HATU (91 mg, 0.24 mmol, 1.2 equiv.) and DIPEA (111 µL, 0.64 mmol, 3.2 equiv.) in DMF (3.0 mL), 3-(aminomethyl)-4-bromobenzene-1-sulfonic acid (**19**, 53 mg, 0.20 mmol, 1.0 equiv.) was added and the mixture was allowed to stir at 25 °C for 1 h. The mixture was purified by reversed-phase flash column chromatography using eluents MeCN and H_2O (gradient from 5:95 to 50:50). The product ethylbis(propan-2-yl)azanium 4-bromo-3-[[(2-chloropyrimidin-4yl)formamido]methyl]benzene-1-sulfonate (**21**) was obtained as a light yellow oil (70 mg, 65% yield). ^1H NMR (500 MHz, DMSO-d_6) δ 9.66 (t, J = 6.2 Hz, 1H), 9.05 (d, J = 4.9 Hz, 1H), 8.24 (s, 1H), 8.06 (d, J = 4.9 Hz, 1H), 7.59 (d, J = 8.2 Hz, 1H), 7.57 (d, J = 1.8 Hz, 1H), 7.44 (dd, J = 8.2, 2.0 Hz, 1H), 4.54 (d, J = 6.2 Hz, 2H), 3.62 – 3.56 (m, 2H), 3.11 (qd, J = 7.3, 4.2 Hz, 2H), 1.23 (dd, J = 14.9, 7.7 Hz, 15H); ^{13}C NMR (126 MHz, DMSO-d_6) δ 163.70, 162.35, 160.32, 160.16, 148.37, 137.30, 132.41, 126.72, 125.99, 122.73, 118.57, 54.25, 43.64, 42.49, 18.66, 17.31, 13.01; HRMS (ESI$^-$) *m/z* [M–H]$^-$, calcd. for $C_{12}H_8N_3O_4SClBr$: 403.9107, found: 403.9079; Purity by HPLC: 100%.

***N*-[(2-Bromo-5-sulfamoylphenyl)methyl]-2-chloropyrimidine-4-carboxamide (22)**

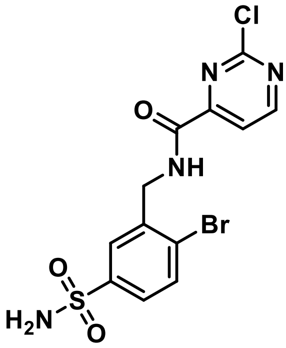

To a solution of ethylbis(propan-2-yl)azanium 4-bromo-3-[[(2-chloropyrimidin-4yl)formamido]methyl]benzene-1-sulfonate (**21**, 70 mg, 0.13 mmol, 1.0 equiv.) in DCM (3 mL) and DMF (0.1 mL), $SOCl_2$ was added (463 µL, 6.5 mmol, 50.0 equiv.) and the mixture was stirred at 25 °C for 0.5 h. The volatiles were removed under reduced pressure. Ammonia solution (25% in H_2O, 10 mL) was added, and white precipitation appeared immediately. The mixture was allowed to stir at 25 °C for 0.5 h, then it was filtered and washed with H_2O (2 × 5 mL). The residue was purified by reversed-phase flash column chromatography using eluents A (0.1% HCOOH in MeCN) and B (0.1% HCOOH in H_2O) (gradient from 10:90 to 80:20). The product *N*-[(2-bromo-5-sulfamoylphenyl)methyl]-2-chloropyrimidine-4-carboxamide (**22**) was obtained as a white solid (26 mg, 49% yield). Mp.: 166–167 °C; ^{1}H NMR (500 MHz, DMSO-*d*$_6$) δ 9.69 (t, J = 6.1 Hz, 1H), 9.05 (d, J = 4.9 Hz, 1H), 8.06 (d, J = 4.9 Hz, 1H), 7.85 (d, J = 8.3 Hz, 1H), 7.73 (d, J = 1.9 Hz, 1H), 7.65 (dd, J = 8.3, 2.2 Hz, 1H), 7.46 (s, 2H), 4.58 (d, J = 6.1 Hz, 2H); ^{13}C NMR (126 MHz, DMSO-*d*$_6$) δ 163.05, 161.93, 159.66, 159.51, 143.70, 138.37, 133.10, 125.95, 125.65, 125.18, 117.97, 43.15; HRMS (ESI$^-$) *m/z* [M–H]$^-$, calcd. for $C_{12}H_9N_4O_3SClBr$: 402.9267, found: 402.9255; Purity by HPLC: 97.9%.

2-(2-Bromo-5-sulfamoylphenyl)-*N*-(4-methylpyridin-3-yl)acetamide (24)

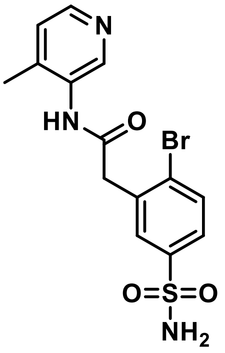

To a stirred solution of 2-(2-bromo-5-sulfamoylphenyl)acetic acid (**49**, 18 mg, 0.06 mmol, 1.2 equiv.), HATU (23 mg, 0.06 mmol, 1.2 equiv.) and DIPEA (19 µL, 1.10 mmol, 2.2 equiv.) in

DMF (1.0 mL), 3-amino-4-methylpyridine (**50**, 5 mg, 0.05 mmol, 1.0 equiv.) was added and the mixture was allowed to stir at 25 °C for 1 h. The mixture was purified by reversed-phase flash column chromatography using eluents A (0.1% HCOOH in MeCN) and B (0.1% HCOOH in H_2O) (gradient from 5:95 to 60:40). The product 2-(2-bromo-5-sulfamoylphenyl)-*N*-(4-methylpyridin-3-yl)acetamide (**24**) was obtained as a white solid (12 mg, 62% yield). Mp.: 148–150 °C; ^{1}H NMR (500 MHz, DMSO-d_6) δ 9.88 (s, 1H), 8.51 (s, 1H), 8.25 (d, J = 4.7 Hz, 1H), 7.89 (d, J = 1.9 Hz, 1H), 7.85 (d, J = 8.4 Hz, 1H), 7.65 (dd, J = 8.3, 2.2 Hz, 1H), 7.46 (s, 2H), 7.27 (d, J = 4.7 Hz, 1H), 4.01 (s, 2H), 2.26 (s, 3H); ^{13}C NMR (126 MHz, DMSO-d_6) δ 168.28, 146.40, 146.36, 143.61, 141.89, 136.96, 133.53, 133.36, 129.27, 128.63, 126.25, 125.76, 42.73, 17.60; HRMS (ESI$^-$) *m/z* [M–H]$^-$, calcd. for $C_{14}H_{13}N_3O_3SBr$: 381.9860, found: 381.9869; Purity by HPLC: 98.8%.

***N*-[(2-Benzyl-5-sulfamoylphenyl)methyl]-2-cyanopyrimidine-4-carboxamide (25)**

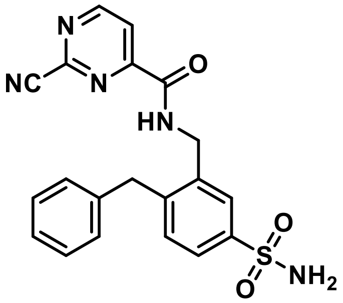

To a solution of *N*-[(2-benzyl-5-sulfamoylphenyl)methyl]-2-chloropyrimidine-4-carboxamide (**32**, 15 mg, 0.035 mmol, 1.0 equiv.) in DMSO (1.0 mL), DABCO (12 mg, 0.105 mmol, 3.0 equiv.) and KCN (3 mg, 0.049 mmol, 1.4 equiv.) were added and the mixture was allowed to stir at 25 °C for 1 h. The solution was subjected to reversed-phase flash column chromatography using eluents A (0.1% HCOOH in MeCN) and B (0.1% HCOOH in H_2O) (gradient from 10:90 to 80:20). The product *N*-[(2-Benzyl-5-sulfamoylphenyl)methyl]-2-cyanopyrimidine-4-carboxamide (**25**) was obtained as a white solid (11 mg, 78% yield).

Mp.: 205–206 °C; ^{1}H NMR (500 MHz, DMSO-d_6) δ 9.68 (t, J = 6.0 Hz, 1H), 9.23 (d, J = 5.1 Hz, 1H), 8.26 (d, J = 5.1 Hz, 1H), 7.79 (s, 1H), 7.69 (d, J = 8.0 Hz, 1H), 7.37 (d, J = 8.0 Hz, 1H), 7.30 – 7.23 (m, 4H), 7.16 (t, J = 7.2 Hz, 3H), 4.55 (d, J = 6.1 Hz, 2H), 4.19 (s, 2H); ^{13}C NMR (126 MHz, DMSO-d_6) δ 161.91, 161.87, 158.25, 143.73, 143.06, 142.89, 140.25, 138.09, 131.37, 129.16, 129.07, 126.70, 125.90, 125.11, 122.31, 116.40, 41.07, 38.14; HRMS (ESI$^-$) *m/z* [M–H]$^-$, calcd. for $C_{20}H_{16}N_5O_3S$: 406.0973, found: 406.0966; Purity by HPLC: 99.6%.

N-{[2-(3-Chlorophenoxy)-5-sulfamoylphenyl]methyl}-2-cyanopyrimidine-4-carboxamide (26)

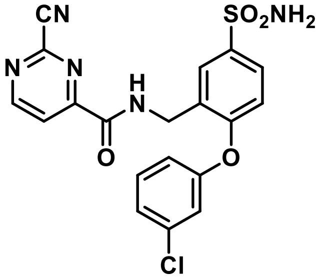

To a solution of 2-chloro-*N*-{[2-(3-chlorophenoxy)-5-sulfamoylphenyl]methyl}pyrimidine-4-carboxamide (**41**, 43 mg, 0.095 mmol, 1.0 equiv.) in DMSO (2.0 mL), DABCO (32 mg, 0.29 mmol, 3.0 equiv.) and KCN (9 mg, 0.13 mmol, 1.4 equiv.) were added and the mixture was allowed to stir at 25 °C for 1 h. The solution was subjected to reversed-phase flash column chromatography using eluents A (0.1% HCOOH in MeCN) and B (0.1% HCOOH in H_2O) (gradient from 10:90 to 80:20). The product *N*-{[2-(3-chlorophenoxy)-5-sulfamoylphenyl]methyl}-2-cyanopyrimidine-4-carboxamide (**26**) was obtained as a white solid (36 mg, 85% yield). Mp.: 176–177 °C; ^{1}H NMR (500 MHz, DMSO-d_6) δ 9.73 (t, J = 6.0 Hz, 1H), 9.24 (d, J = 5.1 Hz, 1H), 8.25 (d, J = 5.1 Hz, 1H), 7.87 (d, J = 1.8 Hz, 1H), 7.76 (dd, J = 8.5, 2.0 Hz, 1H), 7.41 (t, J = 8.2 Hz, 1H), 7.36 (s, 2H), 7.19 (d, J = 8.0 Hz, 1H), 7.11 – 7.06 (m, 2H), 7.03 (dd, J = 8.2, 1.8 Hz, 1H), 4.62 (d, J = 6.0 Hz, 2H); ^{13}C NMR (126 MHz, DMSO-d_6) δ 161.30, 161.22, 157.55, 157.09, 155.81, 143.09, 139.76, 133.99, 131.45, 130.25, 126.83, 126.67, 123.79, 121.60, 118.98, 118.49, 117.26, 115.72, 37.89; HRMS (ESI$^+$) *m/z* $[M+NH_4]^+$, calcd. for $C_{19}H_{18}N_6O_4SCl$: 461.0793, found: 461.0773; Purity by HPLC: 99.4%.

2-Cyano-*N*-{[5-sulfamoyl-2-(4-sulfamoylphenoxy)phenyl]methyl}pyrimidine-4-carboxamide (27)

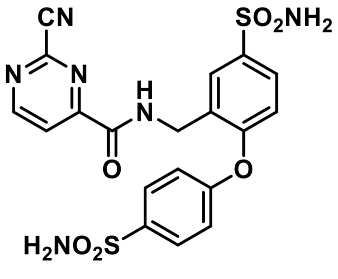

To a solution of 2-chloro-*N*-{[5-sulfamoyl-2-(4-sulfamoylphenoxy)phenyl]methyl}pyrimidine-4-carboxamide (**40**, 43 mg, 0.092 mmol, 1.0 equiv.) in DMSO (2.0 mL), DABCO (31 mg, 0.28 mmol, 3.0 equiv.) and KCN (9 mg, 0.13 mmol, 1.4 equiv.) were added and the mixture was allowed to stir at 25 °C for 1 h. The solution

was subjected to reversed-phase flash column chromatography using eluents A (0.1% HCOOH in MeCN) and B (0.1% HCOOH in H_2O) (gradient from 10:90 to 80:20). The product 2-cyano-*N*-{[5-sulfamoyl-2-(4-sulfamoylphenoxy)phenyl]methyl}pyrimidine-4-carboxamide (**27**) was obtained as a white solid (39 mg, 87% yield). Mp.: 275–276 °C; ^{1}H NMR (500 MHz, DMSO-*d*$_6$) δ 9.79 (t, *J* = 6.0 Hz, 1H), 9.24 (d, *J* = 5.1 Hz, 1H), 8.26 (d, *J* = 5.1 Hz, 1H), 7.90 – 7.81 (m, 3H), 7.78 (dd, *J* = 8.5, 2.0 Hz, 1H), 7.36 (br s, 4H), 7.19 (d, *J* = 8.7 Hz, 2H), 7.13 (d, *J* = 8.5 Hz, 1H), 4.60 (d, *J* = 5.9 Hz, 2H); ^{13}C NMR (126 MHz, DMSO-*d*$_6$) δ 161.45, 161.29, 158.68, 157.56, 155.26, 143.15, 140.15, 139.29, 130.60, 128.11, 126.67, 126.62, 121.63, 119.44, 118.14, 115.77, 37.67; HRMS (ESI$^+$) *m/z* [M+H]$^+$, calcd. for $C_{19}H_{17}N_6O_6S_2$: 489.0651, found: 489.0638; Purity by HPLC: 100%.

***N*-{[2-Bromo-5-(phenylsulfamoyl)phenyl]methyl}-2-cyanopyrimidine-4-carboxamide (28)**

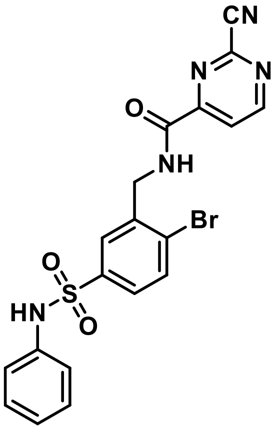

To a solution of *N*-{[2-bromo-5-(phenylsulfamoyl)phenyl]methyl}-2-chloropyrimidine-4-carboxamide (**42**, 72 mg, 0.15 mmol, 1.0 equiv.) in DMSO (2.0 mL), DABCO (50 mg, 0.45 mmol, 3.0 equiv.) and KCN (14 mg, 0.21 mmol, 1.4 equiv.) were added and the mixture was allowed to stir at 25 °C for 1 h. The solution was subjected to reversed-phase flash column chromatography using eluents A (0.1% HCOOH in MeCN) and B (0.1% HCOOH in H_2O) (gradient from 10:90 to 80:20). The product *N*-{[2-bromo-5-(phenylsulfamoyl)phenyl]methyl}-2-cyanopyrimidine-4-carboxamide (**28**) was obtained as a white solid (50 mg, 71% yield). Mp.: 162–164 °C; ^{1}H NMR (500 MHz, DMSO-*d*$_6$) 10.34 (br s, 1H), 9.83 (t, *J* = 6.0 Hz, 1H), 9.31 (d, *J* = 5.1 Hz, 1H), 8.32 (d, *J* = 5.1 Hz, 1H), 7.82 (d, *J* = 8.3 Hz, 1H), 7.66 (d, *J* = 1.7 Hz, 1H), 7.56 (dd, *J* = 8.3, 2.0 Hz, 1H), 7.13 (t, *J* = 7.8 Hz, 2H), 7.01 (d, *J* = 7.8 Hz, 2H), 6.93 (t, *J* = 7.3 Hz, 1H), 4.53 (d, *J* = 6.1 Hz, 2H); ^{13}C NMR (126 MHz, DMSO-*d*$_6$) δ 161.55, 161.42, 157.40, 143.18, 139.32, 138.70, 137.30, 133.40, 129.00, 126.91,

126.76, 125.96, 124.14, 121.78, 120.41, 115.78, 43.01; HRMS (ESI$^+$) *m/z* $[M+H]^+$, calcd. for $C_{19}H_{15}N_5O_3Br$: 472.0073, found: 472.0052; Purity by HPLC: 99.7%.

3-(Aminomethyl)-4-benzylbenzene-1-sulfonic acid (30)

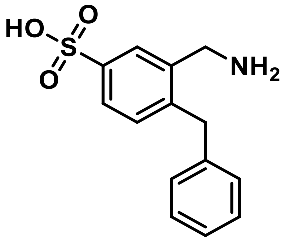

To a solution of 3-(aminomethyl)-4-bromobenzene-1-sulfonic acid (**19**, 479 mg, 1.80 mmol, 1.0 equiv.) in H_2O (5 mL) and DMF (4 mL), K_2CO_3 (746 mg, 5.4 mmol, 3.0 equiv.) and benzyl boronic acid pinacol ester (**29**, 641 µL, 2.88 mmol, 1.6 equiv.) were added. Next, SPhos (111 mg, 0.27 mmol, 0.15 equiv.) and $PdCl_2$ (16 mg, 0.09 mmol, 0.05 equiv.) were added and the mixture was allowed to stir at 95 °C for 2 h. A dark brown solution was obtained. It was subjected to reversed-phase flash column chromatography using eluents MeCN and H_2O (gradient from 5:95 to 40:60). The product 3-(aminomethyl)-4-benzylbenzene-1-sulfonic acid (**30**) was obtained as a white solid (150 mg, 30% yield). Mp.: 295–297 °C; ^{1}H NMR (500 MHz, DMSO-*d6*) δ 7.76 (s, 1H), 7.55 (dd, J = 7.9, 1.5 Hz, 1H), 7.30 (t, J = 7.5 Hz, 2H), 7.20 (t, J = 7.4 Hz, 1H), 7.16 (d, J = 7.9 Hz, 1H), 7.12 (d, J = 7.3 Hz, 2H), 4.06 (s, 2H), 3.97 (s, 2H); ^{13}C NMR (126 MHz, DMSO-*d6*) δ 146.94, 139.74, 139.21, 131.89, 129.79, 128.52, 128.50, 126.15, 125.84, 125.62, 39.39, 37.44; HRMS (ESI$^-$) *m/z* $[M-H]^-$, calcd. for $C_{14}H_{14}NO_3S$: 276.0694, found: 276.0684; Purity by HPLC: 98.8%.

Ethylbis(propan-2-yl)azanium 4-benzyl-3-[[(2-chloropyrimidin-4-yl)formamido]methyl]benzene-1-sulfonate (31)

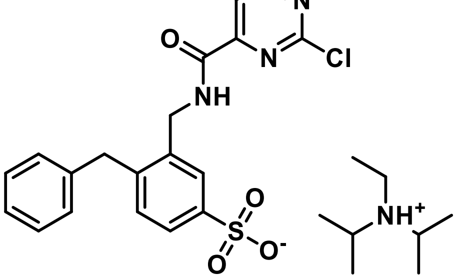

To a stirred solution of 2-chloropyrimidine-4-carboxylic acid (**20**, 72 mg, 0.46 mmol, 1.2 equiv.), HATU (173 mg, 0.46 mmol, 1.2 equiv.) and DIPEA (173 µL, 1.22 mmol, 3.2 equiv.) in DMF (3.0 mL), 3-(aminomethyl)-4-benzylbenzene-1-sulfonic acid (**30**, 105 mg, 0.38 mmol,

1.0 equiv.) was added and the mixture was allowed to stir at 25 °C for 1 h. The mixture was purified by reversed-phase flash column chromatography using eluents MeCN and H_2O (gradient from 5:95 to 50:50). The product ethylbis(propan-2-yl)azanium 4-benzyl-3-[[(2-chloropyrimidin-4-yl)formamido]methyl]benzene-1-sulfonate (**31**) was obtained as a colourless oil (76 mg, 48% yield). ^{1}H NMR (500 MHz, DMSO-*d*$_6$) δ 9.43 (t, J = 6.1 Hz, 1H), 9.00 (d, J = 4.9 Hz, 1H), 8.00 (d, J = 4.9 Hz, 1H), 7.61 (d, J = 1.2 Hz, 1H), 7.46 (dd, J = 7.8, 1.5 Hz, 1H), 7.29 – 7.20 (m, 2H), 7.14 (dd, J = 6.8, 3.6 Hz, 4H), 4.46 (d, J = 6.2 Hz, 2H), 4.11 (s, 2H), 3.68 – 3.51 (m, 2H), 3.12 (qd, J = 7.3, 4.3 Hz, 2H), 1.23 (dt, J = 11.2, 5.7 Hz, 15H); ^{13}C NMR (126 MHz, DMSO-*d*$_6$) δ 162.88, 161.27, 159.76, 159.54, 146.45, 140.36, 138.61, 136.04, 129.62, 128.45, 128.33, 125.86, 125.38, 124.38, 117.85, 53.61, 41.86, 40.30, 37.57, 18.05, 16.70, 12.43; HRMS (ESI$^-$) *m/z* [M–H]$^-$, calcd. for $C_{19}H_{15}N_3O_4SCl$: 416.0471, found: 416.0452; Purity by HPLC: 98.9%.

***N*-[(2-Benzyl-5-sulfamoylphenyl)methyl]-2-chloropyrimidine-4-carboxamide (32)**

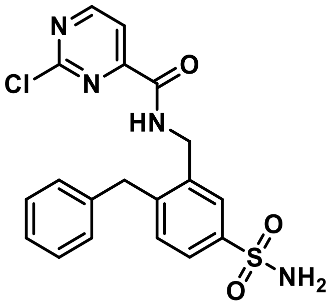

To a solution of ethylbis(propan-2-yl)azanium 4-benzyl-3-[[(2-chloropyrimidin-4-yl)formamido]methyl]benzene-1-sulfonate (**31**, 82 mg, 0.15 mmol, 1.0 equiv.) in DCM (5 mL) and DMF (0.15 mL), $SOCl_2$ was added (640 µL, 9.0 mmol, 60.0 equiv.) and the mixture was stirred at 25 °C for 1 h. The volatiles were removed under reduced pressure. Ammonia solution (25% in H_2O, 10 mL) was added, and white precipitation appeared immediately. The mixture was allowed to stir at 25 °C for 0.5 h, then it was filtered and washed with H_2O (2 × 5 mL). The residue was purified by reversed-phase flash column chromatography using eluents A (0.1% HCOOH in MeCN) and B (0.1% HCOOH in H_2O) (gradient from 10:90 to 80:20). The product *N*-[(2-benzyl-5-sulfamoylphenyl)methyl]-2-chloropyrimidine-4-carboxamide (**32**) was obtained as a white solid (28 mg, 43% yield). Mp.: 109–110 °C; ^{1}H NMR (500 MHz, DMSO-*d*$_6$) δ 9.51 (t, J = 6.0 Hz, 1H), 9.01 (d, J = 4.9 Hz, 1H), 8.01 (d, J = 5.0 Hz, 1H), 7.78 (s, 1H), 7.69 (dd, J = 8.0, 1.6 Hz, 1H), 7.37 (d, J = 8.0 Hz, 1H), 7.30 – 7.25 (m, 4H), 7.17 (t, J = 7.1 Hz, 3H), 4.54 (d, J = 6.1 Hz, 2H), 4.18 (s, 2H); ^{13}C NMR (126 MHz, DMSO-*d*$_6$) δ 162.84, 161.51, 159.67, 159.51, 142.39, 142.23, 139.57, 137.53, 130.65, 128.52, 128.42, 126.06, 125.08, 124.41,

117.84, 40.31, 37.49; HRMS (ESI+) *m/z* $[M+H]^+$, calcd. for $C_{19}H_{18}N_4O_3SCl$: 417.0788, found: 417.0804; Purity by HPLC: 99.8%.

3-Cyano-4-(4-sulfamoylphenoxy)benzene-1-sulfonamide (36)

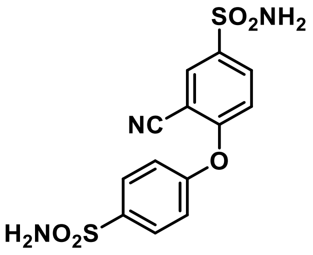

To a solution of 3-cyano-4-fluorobenzensulfonamide (**33**, 400 mg, 2.00 mmol, 1.0 equiv.) in DMF (4.0 mL) and DIPEA (418 µL, 2.40 mmol, 1.2 equiv.), 4-hydroxybenzenesulfonamide (**34**, 416 mg, 2.40 mmol, 1.2 equiv.) was added and the mixture was allowed to stir at 70 °C for 16 h. The solution was subjected to reversed-phase flash column chromatography using eluents A (0.1% HCOOH in MeCN) and B (0.1% HCOOH in H_2O) (gradient from 10:90 to 80:20). The product 3-cyano-4-(4-sulfamoylphenoxy)benzene-1-sulfonamide (**36**) was obtained as a white solid (439 mg, 62% yield). Mp.: 204–205 °C; ^{1}H NMR (500 MHz, DMSO-*d*6) δ 8.30 (d, *J* = 2.1 Hz, 1H), 8.09 (dd, *J* = 8.9, 2.1 Hz, 1H), 7.94 (d, *J* = 8.7 Hz, 2H), 7.47 (br s, 4H), 7.43 (d, *J* = 8.7 Hz, 2H), 7.23 (d, *J* = 8.9 Hz, 1H); ^{13}C NMR (126 MHz, DMSO-*d*6) δ 159.97, 156.62, 141.18, 139.88, 132.82, 131.85, 128.42, 120.03, 118.37, 114.61, 103.46; HRMS (ESI+) *m/z* $[M+NH_4]^+$, calcd. for $C_{13}H_{14}N_4O_5S_2$: 371.0478, found: 371.0460; Purity by HPLC: 99.7%.

4-(3-Chlorophenoxy)-3-cyanobenzene-1-sulfonamide (37)

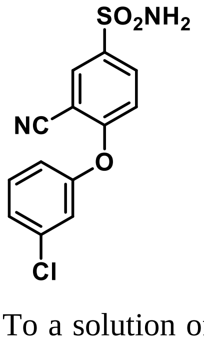

To a solution of 3-cyano-4-fluorobenzensulfonamide (**33**, 400 mg, 2.00 mmol, 1.0 equiv.) in DMF (4.0 mL) and DIPEA (418 µL, 2.40 mmol, 1.2 equiv.), 3-chlorophenol (**35**, 309 mg, 2.40 mmol, 1.2 equiv.) was added and the mixture was allowed to stir at 70 °C for 16 h. The solution was subjected to reversed-phase flash column chromatography using eluents A (0.1% HCOOH in MeCN) and B (0.1% HCOOH in H_2O) (gradient from 10:90 to 80:20). The product 4-(3-chlorophenoxy)-3-cyanobenzene-1-sulfonamide (**37**) was obtained as a white solid (343 mg,

56% yield). Mp.: 140–41 °C; ^{1}H NMR (500 MHz, DMSO-d_6) δ 8.27 (d, J = 2.1 Hz, 1H), 8.05 (dd, J = 8.9, 2.1 Hz, 1H), 7.61 – 7.48 (m, 3H), 7.45 (s, 1H), 7.41 (d, J = 8.0 Hz, 1H), 7.26 (dd, J = 8.2, 1.6 Hz, 1H), 7.15 (d, J = 8.9 Hz, 1H); ^{13}C NMR (126 MHz, DMSO-d_6) δ 160.61, 154.71, 139.36, 134.43, 132.73, 131.95, 131.74, 126.02, 120.60, 119.07, 117.39, 114.70, 102.79; HRMS (ESI$^-$) m/z [M–H]$^-$, calcd. for $C_{13}H_8N_2O_3SCl$: 306.9944, found: 306.9951; Purity by HPLC: 100%.

3-(Aminomethyl)-4-(4-sulfamoylphenoxy)benzene-1-sulfonamide (38)

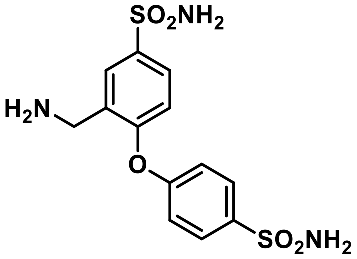

3-Cyano-4-(4-sulfamoylphenoxy)benzene-1-sulfonamide (**36**, 346 mg, 0.98 mmol, 1.0 equiv.) was dissolved in THF (3.0 mL), then borane dimethyl sulfide complex (2 M solution in THF, 2.88 mL, 6.0 equiv.) was added and the mixture was allowed to stir at 100 °C for 1 h in a microwave reactor. It was cooled to 25 °C, then MeOH (4.0 mL) was added dropwise. The volatiles were evaporated, and crude product 3-(aminomethyl)-4-(4-sulfamoylphenoxy)benzene-1-sulfonamide (**38**) was obtained as a white solid (310 mg, 89% yield), and it was used immediately in the next step. HRMS (ESI$^+$) m/z [M+H]$^+$, calcd. for $C_{13}H_{16}N_3O_5S_2$: 358.0525, found: 358.0509; Purity by HPLC: 98.3%.

3-(Aminomethyl)-4-(3-chlorophenoxy)benzene-1-sulfonamide (39)

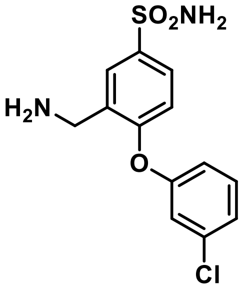

4-(3-Chlorophenoxy)-3-cyanobenzene-1-sulfonamide (**37**, 250 mg, 0.81 mmol, 1.0 equiv.) was dissolved in THF (3.0 mL), then borane dimethyl sulfide complex (2 M solution in THF, 2.43 mL, 6.0 equiv.) was added and the mixture was allowed to stir at 100 °C for 1 h in a microwave reactor. It was cooled to 25 °C, then MeOH (3.0 mL) was added dropwise. The volatiles were evaporated, and crude product 3-(aminomethyl)-4-(3-chlorophenoxy)benzene-1-sulfonamide

(**39**) was obtained as a white solid (213 mg, 84% yield), and it was used immediately in the next step. HRMS (ESI$^+$) *m/z* [M+H]$^+$, calcd. for $C_{13}H_{14}N_2O_3SCl$: 313.0408, found: 313.0395; Purity by HPLC: 98.8%.

2-Chloro-*N*-{[5-sulfamoyl-2-(4-sulfamoylphenoxy)phenyl]methyl}pyrimidine-4-carboxamide (40)

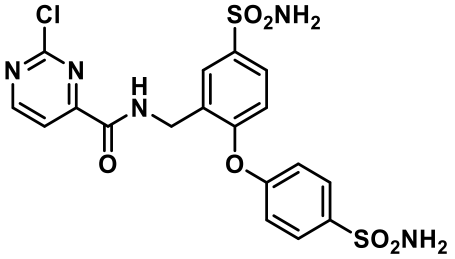

To a stirred solution of 2-chloropyrimidine-4-carboxylic acid (**20**, 152 mg, 0.96 mmol, 1.2 equiv.), HATU (365 mg, 0.96 mmol, 1.2 equiv.) and DIPEA (307 µL, 1.76 mmol, 2.2 equiv.) in DMF (5.0 mL), 3-(aminomethyl)-4-(4-sulfamoylphenoxy)benzene-1-sulfonamide (**38**, 286 mg, 0.80 mmol, 1.0 equiv.) was added and the mixture was allowed to stir at 25 °C for 1 h. The mixture was purified by reversed-phase flash column chromatography using eluents A (0.1% HCOOH in MeCN) and B (0.1% HCOOH in H_2O) (gradient from 10:90 to 80:20). The product 2-chloro-*N*-{[5-sulfamoyl-2-(4-sulfamoylphenoxy)phenyl]methyl}pyrimidine-4-carboxamide (**40**) was obtained as a white solid (199 mg, 50% yield). Mp.: 263–265 °C; ^{1}H NMR (500 MHz, DMSO-*d*$_6$) δ 9.60 (t, *J* = 6.1 Hz, 1H), 9.02 (d, *J* = 4.9 Hz, 1H), 8.01 (d, *J* = 4.9 Hz, 1H), 7.86 (d, *J* = 8.7 Hz, 3H), 7.77 (dd, *J* = 8.5, 2.0 Hz, 1H), 7.36 (br s, 4H), 7.19 (d, *J* = 8.7 Hz, 2H), 7.12 (d, *J* = 8.5 Hz, 1H), 4.57 (d, *J* = 6.1 Hz, 2H); ^{13}C NMR (126 MHz, DMSO-*d*$_6$) δ 162.92, 161.70, 159.62, 159.57, 158.65, 155.26, 140.12, 139.33, 130.68, 128.12, 126.60, 126.45, 119.34, 118.18, 117.83, 37.58; HRMS (ESI$^+$) *m/z* [M+H]$^+$, calcd. for $C_{18}H_{17}N_5O_6S_2Cl$: 498.0308, found: 498.0289; Purity by HPLC: 99.9%.

2-Chloro-*N*-{[2-(3-chlorophenoxy)-5-sulfamoylphenyl]methyl}pyrimidine-4-carboxamide (41)

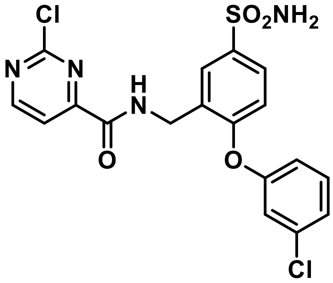

To a stirred solution of 2-chloropyrimidine-4-carboxylic acid (**20**, 95 mg, 0.60 mmol, 1.2 equiv.), HATU (228 mg, 0.60 mmol, 1.2 equiv.) and DIPEA (192 µL, 1.10 mmol, 2.2 equiv.) in DMF (3.0 mL), 3-(aminomethyl)-4-(3-chlorophenoxy)benzene-1-sulfonamide (**39**, 156 mg, 0.50 mmol, 1.0 equiv.) was added and the mixture was allowed to stir at 25 °C for 1 h. The mixture was purified by reversed-phase flash column chromatography using eluents A (0.1% HCOOH in MeCN) and B (0.1% HCOOH in H_2O) (gradient from 10:90 to 80:20). The product 2-chloro-*N*-{[2-(3-chlorophenoxy)-5-sulfamoylphenyl]methyl}pyrimidine-4-carboxamide (**41**) was obtained as a white solid (120 mg, 53% yield). Mp.: 168–69 °C; ^{1}H NMR (500 MHz, DMSO-d_6) δ 9.54 (t, *J* = 6.0 Hz, 1H), 9.02 (d, *J* = 4.9 Hz, 1H), 8.00 (d, *J* = 4.9 Hz, 1H), 7.85 (d, *J* = 1.9 Hz, 1H), 7.75 (dd, *J* = 8.5, 2.2 Hz, 1H), 7.42 (t, *J* = 8.2 Hz, 1H), 7.35 (s, 2H), 7.21 (dd, *J* = 8.0, 1.1 Hz, 1H), 7.10 (t, *J* = 2.0 Hz, 1H), 7.07 (d, *J* = 8.6 Hz, 1H), 7.03 (dd, *J* = 8.2, 1.9 Hz, 1H), 4.60 (d, *J* = 6.1 Hz, 2H); ^{13}C NMR (126 MHz, DMSO-d_6) δ 162.85, 161.57, 159.59, 159.54, 157.02, 155.81, 139.71, 134.01, 131.47, 130.29, 126.64, 126.59, 123.87, 118.82, 118.58, 117.79, 117.30, 37.78; HRMS (ESI$^+$) *m/z* $[M+H]^+$, calcd. for $C_{18}H_{15}N_4O_4SCl_2$: 453.0191, found: 453.0185; Purity by HPLC: 98.9%.

***N*-{[2-Bromo-5-(phenylsulfamoyl)phenyl]methyl}-2-chloropyrimidine-4-carboxamide (42)**

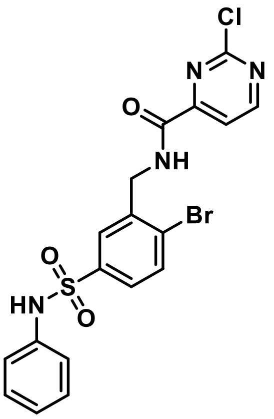

To a solution of ethylbis(propan-2-yl)azanium 4-bromo-3-[[(2-chloropyrimidin-4yl)formamido]methyl]benzene-1-sulfonate (**21**, 216 mg, 0.53 mmol, 1.0 equiv.) in DCM (4 mL) and DMF (0.1 mL), $SOCl_2$ was added (1.93 mL, 26.5 mmol, 50.0 equiv.) and the mixture was stirred at 25 °C for 0.5 h. The volatiles were removed under reduced pressure and the residue was dissolved in pyridine (2.0 mL). Aniline (58 µL, 0.64 mmol, 1.2 equiv.) was added, and the mixture was allowed to stir at 25 °C for 0.5 h, then it was purified by reversed-phase flash column chromatography using eluents A (0.1% HCOOH in MeCN) and B (0.1% HCOOH in H_2O) (gradient from 10:90 to 80:20). The product *N*-{[2-bromo-5-

(phenylsulfamoyl)phenyl]methyl}-2-chloropyrimidine-4-carboxamide (**42**) was obtained as a yellow solid (119 mg, 47% yield). Mp.: 148–149 °C; ^{1}H NMR (500 MHz, DMSO-d_6) 10.33 (s, 1H), 9.65 (t, *J* = 6.0 Hz, 1H), 9.09 (d, *J* = 4.9 Hz, 1H), 8.07 (d, *J* = 4.9 Hz, 1H), 7.82 (d, *J* = 8.3 Hz, 1H), 7.65 (d, *J* = 1.7 Hz, 1H), 7.56 (dd, *J* = 8.3, 2.0 Hz, 1H), 7.13 (t, *J* = 7.8 Hz, 2H), 7.02 (d, *J* = 7.8 Hz, 2H), 6.93 (t, *J* = 7.3 Hz, 1H), 4.52 (d, *J* = 6.1 Hz, 2H); ^{13}C NMR (126 MHz, DMSO-d_6) δ 163.02, 161.79, 159.64, 159.42, 139.22, 138.83, 137.18, 133.37, 129.00, 126.90, 126.74, 125.87, 124.23, 120.44, 117.95, 42.97; HRMS (ESI$^-$) *m/z* [M–H]$^-$, calcd. for $C_{18}H_{13}N_4O_3SClBr$: 478.9580, found: 478.9590; Purity by HPLC: 98.1%.

4-Bromo-*N*-phenylbenzene-1-sulfonamide (43)

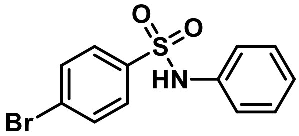

Aniline (110 µL, 1.2 mmol, 1.0 equiv.) was dissolved in pyridine (2.0 mL), and 4-bromobenzenesulfonyl chloride (307 mg, 1.2 mmol, 1.0 equiv.) was added in one portion. The solution was allowed to stir for 1 h, then it was subjected to reversed-phase flash column chromatography using eluents A (0.1% HCOOH in MeCN) and B (0.1% HCOOH in H_2O) (gradient from 10:90 to 80:20). The product 4-bromo-*N*-phenylbenzene-1-sulfonamide (**43**) was obtained as a yellow solid (297 mg, 79% yield). Mp.: 105–106 °C; ^{1}H NMR (500 MHz, DMSO-d_6) δ 10.33 (s, 1H), 7.75 (d, *J* = 8.6 Hz, 2H), 7.66 (d, *J* = 8.6 Hz, 2H), 7.24 (t, *J* = 7.9 Hz, 2H), 7.09 (d, *J* = 7.7 Hz, 2H), 7.04 (t, *J* = 7.4 Hz, 1H); ^{13}C NMR (126 MHz, DMSO-d_6) δ 138.74, 137.29, 132.28, 129.17, 128.60, 126.69, 124.35, 120.35; HRMS (ESI$^-$) *m/z* [M–H]$^-$, calcd. for $C_{12}H_9NO_2SBr$: 309.9537, found: 309.9550; Purity by HPLC: 99.1%.

***N*-(4-Methylpyridin-3-yl)acetamide (44)**

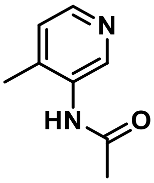

N-(4-Methylpyridin-3-yl)acetamide (**44**) was purchased from a commercial source, and the purity of the compound was checked before biochemical evaluation. Mp.: 67–68 °C; ^{1}H NMR (500 MHz, DMSO-d_6) δ 9.50 (s, 1H), 8.50 (s, 1H), 8.22 (d, J = 4.9 Hz, 1H), 7.24 (d, J = 4.9 Hz, 1H), 2.21 (s, 3H), 2.08 (s, 3H); ^{13}C NMR (126 MHz, DMSO-d_6) δ 168.56, 146.17, 145.74,

140.81, 133.49, 125.19, 23.03, 17.21; HRMS (ESI$^-$) *m/z* [M–H]$^-$, calcd. for $C_8H_9N_2O$: 149.0714, found: 149.0723; Purity by HPLC: 100%.

4-Bromo-3-fluoro-*N*,*N*-bis[(4-methoxyphenyl)methyl]benzene-1-sulfonamide (45)

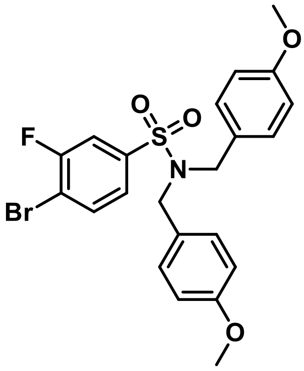

To a solution of 3-fluoro-4-bromobenzenesulfonamide (**10**, 508 mg, 2.00 mmol, 1.0 equiv.) in 2-butanone (5.0 mL), K_2CO_3 (829 mg, 6.00 mmol, 3.0 equiv.) and KI (30 mg, 0.20 mmol, 0.1 equiv.) were added. A solution of 4-methoxybenzyl chloride (**46**, 872 µL, 6.00 mmol, 3.0 equiv.) in 2-butanone (5.0 mL) was added dropwise. The mixture was heated to 80 °C and it was stirred for 2 h. After that, it was cooled to room temperature then filtered through a pad of Celite and washed with DCM (2 × 30 mL). It was extracted with brine (30 mL), and then the organic layer was concentrated. The residue was subjected to reversed-phase flash column chromatography using eluents A (0.1% HCOOH in MeCN) and B (0.1% HCOOH in H_2O) (gradient from 10:90 to 100:0). The product 4-bromo-3-fluoro-*N*,*N*-bis[(4-methoxyphenyl)methyl]benzene-1-sulfonamide (**45**) was obtained as a white solid (892 mg, 90% yield). Mp.: 124–125 °C; ^{1}H NMR (500 MHz, DMSO-*d*$_6$) δ 7.91 (dd, J = 8.3, 6.9 Hz, 1H), 7.68 (dd, J = 8.3, 2.0 Hz, 1H), 7.58 (dd, J = 8.3, 2.1 Hz, 1H), 7.03 (t, J = 5.7 Hz, 4H), 6.81 – 6.77 (m, 4H), 4.26 (s, 4H), 3.71 (s, 6H); ^{13}C NMR (126 MHz, DMSO-*d*$_6$) δ 159.03, 158.66, 157.04, 141.29, 141.24, 134.55, 129.65, 127.64, 124.33, 124.30, 115.24, 115.04, 113.63, 113.39, 113.23, 55.03, 50.42; HRMS (ESI$^-$) *m/z* [M–H]$^-$, calcd. for $C_{22}H_{20}NO_4FSBr$: 492.0280, found: 492.0273; Purity by HPLC: 98.2%.

1,3-Diethyl 2-(5-[bis[(4-methoxyphenyl)methyl]sulfamoyl]-2-bromophenyl)propanedioate (48)

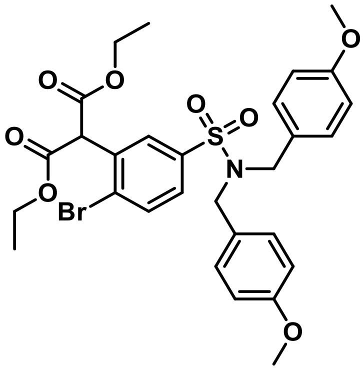

A sealed tube containing 1,4-dioxane (10 mL) was cooled to 0 °C, then NaH (60% suspension in paraffin oil, 400 mg, 10.0 mmol, 25.0 equiv.) was added. Diethyl malonate (**47**, 1525 µL, 10.0 mmol, 25.0 equiv.) was added dropwise and the mixture was allowed to stir at 0 °C for 0.5 h. A solution of 4-bromo-3-fluoro-*N*,*N*-bis[(4-methoxyphenyl)methyl]benzene-1-sulfonamide (**45**, 198 mg, 0.40 mmol, 1.0 equiv.) in 1,4-dioxane (5.0 mL) was added dropwise, the tube was closed, and the mixture was allowed to stir at 160 °C for 16 h. After that, the mixture was cooled to room temperature, and the reaction was quenched with the addition of H_2O (40 mL). The pH of the solution was adjusted to 1 with the addition of 1M HCl. It was extracted with DCM (3 × 60 mL). The residue was subjected to normal-phase flash column chromatography using eluents EtOAc and hexane (gradient from 0:100 to 25:75). The product 1,3-diethyl 2-(5-[bis[(4-methoxyphenyl)methyl]sulfamoyl]-2-bromophenyl)propanedioate (**48**) was obtained as a colourless oil (94 mg, 37% yield). ^{1}H NMR (300 MHz, DMSO-d_6) δ 7.91 (d, J = 7.6 Hz, 2H), 7.72 (d, J = 7.9 Hz, 1H), 6.98 (d, J = 7.8 Hz, 4H), 6.79 (d, J = 7.7 Hz, 4H), 5.31 (s, 1H), 4.31 – 4.09 (m, 8H), 3.71 (s, 6H), 1.19 (t, J = 6.5 Hz, 6H); ^{13}C NMR (75 MHz, DMSO-d_6) δ 166.41, 158.66, 139.48, 134.15, 134.04, 129.58, 129.27, 128.73, 128.11, 127.43, 113.70, 62.10, 56.68, 55.03, 49.93, 13.79; HRMS (ESI$^-$) *m/z* [M–H]$^-$, calcd. for $C_{29}H_{31}NO_8SBr$: 632.0953, found: 632.0934; Purity by HPLC: 98.3%.

2-(2-Bromo-5-sulfamoylphenyl)acetic acid (49)

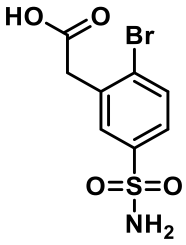

To a solution of 1,3-diethyl 2-(5-[bis[(4-methoxyphenyl)methyl]sulfamoyl]-2-bromophenyl)propanedioate (**48**, 152 mg, 0.24 mmol, 1.0 equiv.) in DCM (5.0 mL), TFA (1838 µL, 24 mmol, 100.0 equiv.) and anisole (156 mg, 1.44 mmol, 6.0 equiv.) were added and the

mixture was allowed to stir at 25 °C for 2 h. The volatiles were evaporated, and the residue was redissolved in cc HCl (2.0 mL) and AcOH (2.0 mL). The mixture was heated to 100 °C and it was allowed to stir for 1 h. It was cooled to room temperature then it was diluted with H_2O (30 mL) and extracted with EtOAc (30 mL). The organic layer was extracted with 0,5 M NaOH (30 ml), and then the pH of the aqueous layer was adjusted to 1 with 20% HCl solution. It was extracted with EtOAc (3 × 60 mL). The merged organic layers were concentrated and the residue was subjected to reversed-phase flash column chromatography using eluents A (0.1% HCOOH in MeCN) and B (0.1% HCOOH in H_2O) (gradient from 10:90 to 60:40). The product 2-(2-bromo-5-sulfamoylphenyl)acetic acid (**49**) was obtained as a white solid (32 mg, 45% yield). Mp.: 249–250 °C; ^{1}H NMR (500 MHz, DMSO-*d*$_6$) δ 7.82 (d, J = 8.3 Hz, 2H), 7.63 (dd, J = 8.4, 2.1 Hz, 1H), 7.45 (s, 2H), 3.81 (s, 2H); ^{13}C NMR (126 MHz, DMSO-*d*$_6$) δ 170.95, 143.49, 136.39, 132.97, 128.88, 128.31, 125.91, 41.38; HRMS (ESI$^-$) *m/z* [M–H]$^-$, calcd. for $C_8H_7NO_4SBr$: 291.9279, found: 291.9267; Purity by HPLC: 97.0%.

2-Cyano-*N*-[(3-sulfamoylphenyl)methyl]pyrimidine-4-carboxamide (51)

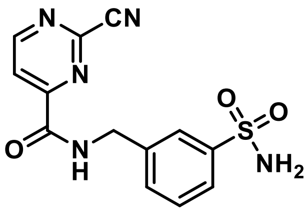

To a solution of 2-chloro-*N*-[(3-sulfamoylphenyl)methyl]pyrimidine-4-carboxamide (**54**, 65 mg, 0.20 mmol, 1.0 equiv.) in DMSO (2.0 mL), DABCO (67 mg, 0.60 mmol, 3.0 equiv.) and KCN (18 mg, 0.28 mmol, 1.4 equiv.) were added and the mixture was allowed to stir at 25 °C for 1 h. The solution was subjected to reversed-phase flash column chromatography using eluents A (0.1% HCOOH in MeCN) and B (0.1% HCOOH in H_2O) (gradient from 10:90 to 80:20). The product 2-cyano-*N*-[(3-sulfamoylphenyl)methyl]pyrimidine-4-carboxamide (**51**) was obtained as a white solid (46 mg, 73% yield). Mp.: 140–142 °C; ^{1}H NMR (500 MHz, DMSO-*d*$_6$) δ 9.86 (s, 1H), 9.24 (d, J = 3.3 Hz, 1H), 8.29 (d, J = 3.3 Hz, 1H), 7.81 (s, 1H), 7.72 (d, J = 7.1 Hz, 1H), 7.59 – 7.49 (m, 2H), 7.34 (s, 2H), 4.57 (d, J = 5.8 Hz, 2H); ^{13}C NMR (126 MHz, DMSO-*d*$_6$) δ 162.00, 161.93, 158.34, 144.85, 143.81, 140.42, 131.60, 129.57, 125.11, 124.97, 122.35, 116.43, 43.14; HRMS (ESI$^-$) *m/z* [M–H]$^-$, calcd. for $C_{13}H_{10}N_5O_3S$: 316.0504, found: 316.0498; Purity by HPLC: 99.8%.

N-[(2-Chloro-5-sulfamoylphenyl)methyl]-2-cyanopyrimidine-4-carboxamide (52)

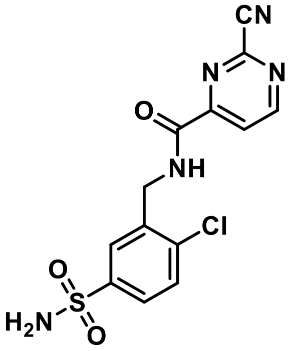

To a solution of 2-chloro-*N*-[(2-chloro-5-sulfamoylphenyl)methyl]pyrimidine-4-carboxamide (**58**, 72 mg, 0.20 mmol, 1.0 equiv.) in DMSO (3.0 mL), DABCO (67 mg, 0.60 mmol, 3.0 equiv.) and KCN (18 mg, 0.28 mmol, 1.4 equiv.) were added and the mixture was allowed to stir at 25 °C for 1 h. The solution was subjected to reversed-phase flash column chromatography using eluents A (0.1% HCOOH in MeCN) and B (0.1% HCOOH in H_2O) (gradient from 10:90 to 80:20). The product *N*-[(2-chloro-5-sulfamoylphenyl)methyl]-2-cyanopyrimidine-4-carboxamide (**52**) was obtained as a white solid (46 mg, 65% yield). Mp.: 179–181 °C; ^{1}H NMR (500 MHz, DMSO-*d*$_6$) δ 9.87 (t, J = 6.1 Hz, 1H), 9.28 (d, J = 5.1 Hz, 1H), 8.32 (d, J = 5.1 Hz, 1H), 7.80 (d, J = 1.9 Hz, 1H), 7.74 (dd, J = 8.4, 2.2 Hz, 1H), 7.69 (d, J = 8.4 Hz, 1H), 7.44 (s, 2H), 4.64 (d, J = 6.1 Hz, 2H); ^{13}C NMR (126 MHz, DMSO-*d*$_6$) δ 161.65, 161.38, 157.48, 143.21, 143.11, 136.69, 135.26, 129.82, 125.88, 125.57, 121.75, 115.78, 40.62; HRMS (ESI$^-$) *m/z* [M–H]$^-$, calcd. for $C_{13}H_9N_5O_3SCl$: 350.0114, found: 350.0103; Purity by HPLC: 98.7%.

2-Chloro-N-[(3-sulfamoylphenyl)methyl]pyrimidine-4-carboxamide (54)

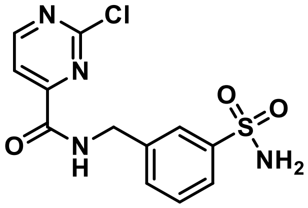

To a stirred solution of 2-chloropyrimidine-4-carboxylic acid (**20**, 133 mg, 0.84 mmol, 1.2 equiv.), HATU (319 mg, 0.84 mmol, 1.2 equiv.) and DIPEA (268 µL, 1.54 mmol, 2.2 equiv.) in DMF (4.0 mL), 3-(aminomethyl)benzenesulfonamide hydrochloride (**53**, 156 mg, 0.70 mmol, 1.0 equiv.) was added and the mixture was allowed to stir at 25 °C for 1 h. The solution was subjected to reversed-phase flash column chromatography using eluents A (0.1% HCOOH in MeCN) and B (0.1% HCOOH in H_2O) (gradient from 10:90 to 80:20). The product 2-chloro-*N*-[(3-sulfamoylphenyl)methyl]pyrimidine-4-carboxamide (**54**) was obtained as a light yellow solid (142 mg, 64% yield). Mp.: 204–206 °C; ^{1}H NMR (500 MHz, DMSO-*d*$_6$) δ 9.68 (t, J = 6.2

Hz, 1H), 9.02 (d, J = 4.9 Hz, 1H), 8.04 (d, J = 4.9 Hz, 1H), 7.81 (s, 1H), 7.72 (d, J = 7.5 Hz, 1H), 7.58 – 7.50 (m, 2H), 7.34 (s, 2H), 4.56 (d, J = 6.3 Hz, 2H); ^{13}C NMR (126 MHz, DMSO-d_6) δ 162.90, 161.60, 159.78, 159.58, 144.20, 139.88, 130.92, 128.91, 124.42, 124.28, 117.90, 42.44; HRMS (ESI$^-$) *m/z* [M–H]$^-$, calcd. for $C_{12}H_{10}N_4O_3SCl$: 325.0162, found: 325.0151; Purity by HPLC: 99.5%.

3-(Aminomethyl)-4-chlorobenzene-1-sulfonic acid (56)

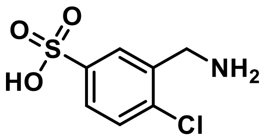

2-Chlorobenzylamine (**55**, 966 µL, 8.00 mmol) was added portion-wise to concentrated sulfuric acid (96%, 8.0 mL). The solution was heated to 150 °C and the reaction was allowed to proceed for 1 h. It was poured into 1,4-dioxane (80 mL). After 1 h, the precipitated solid was filtered and it was washed with 1,4-dioxane (2 × 80 mL) and allowed to air dry. The product 3-(aminomethyl)-4-chlorobenzene-1-sulfonic acid (**56**) was obtained as a white solid (1663 mg, 94% yield). Mp.: 297–299 °C; ^{1}H NMR (500 MHz, DMSO-d_6) δ 8.29 (s, 2H), 7.89 (d, J = 1.6 Hz, 1H), 7.63 (dd, J = 8.2, 1.8 Hz, 1H), 7.49 (d, J = 8.2 Hz, 1H), 4.15 (q, J = 5.5 Hz, 2H); ^{13}C NMR (126 MHz, DMSO-d_6) δ 147.67, 132.72, 130.84, 128.91, 127.55, 127.47, 39.61; HRMS (ESI$^-$) *m/z* [M–H]$^-$, calcd. for $C_7H_7NO_3SCl$: 219.9835, found: 219.9836; Purity by HPLC: 100% (integration at wavelength 190 nm).

Ethylbis(propan-2-yl)azanium 4-chloro-3-[[(2-chloropyrimidin-4-yl)formamido]methyl]benzene-1-sulfonate (57)

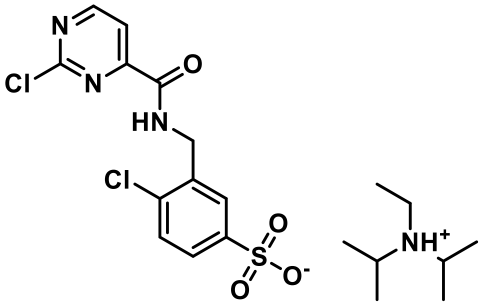

To a stirred solution of 2-chloropyrimidine-4-carboxylic acid (**20**, 190 mg, 1.20 mmol, 1.2 equiv.), HATU (456 mg, 1.20 mmol, 1.2 equiv.) and DIPEA (559 µL, 3.2 mmol, 3.2 equiv.) in DMF (5.0 mL), 3-(aminomethyl)-4-chlorobenzene-1-sulfonic acid (**56**, 222 mg, 1.00 mmol, 1.0 equiv.) was added and the mixture was allowed to stir at 25 °C for 1 h. The mixture was purified by reversed-phase flash column chromatography using eluents MeCN and H_2O (gradient from

5:95 to 50:50). The product ethylbis(propan-2-yl)azanium 4-chloro-3-[[(2-chloropyrimidin-4-yl)formamido]methyl]benzene-1-sulfonate (**57**) was obtained as a light yellow oil (433 mg, 88% yield). ^{1}H NMR (500 MHz, DMSO-*d*$_6$) δ 9.65 (t, J = 6.1 Hz, 1H), 9.04 (d, J = 4.9 Hz, 1H), 8.36 (s, 1H), 8.05 (d, J = 4.9 Hz, 1H), 7.60 (d, J = 1.8 Hz, 1H), 7.51 (dd, J = 8.2, 2.0 Hz, 1H), 7.41 (d, J = 8.2 Hz, 1H), 4.57 (d, J = 6.2 Hz, 2H), 3.59 (dq, J = 13.1, 6.5 Hz, 2H), 3.11 (q, J = 7.3 Hz, 2H), 1.26 – 1.22 (m, 15H); ^{13}C NMR (126 MHz, DMSO-*d*$_6$) δ 163.02, 161.67, 159.65, 159.55, 147.23, 135.09, 131.75, 128.45, 125.80, 125.49, 117.91, 53.52, 41.77, 40.44, 12.37; HRMS (ESI$^-$) *m/z* [M–H]$^-$, calcd. for $C_{12}H_8N_3O_4SCl_2$: 359.9612, found: 359.9600; Purity by HPLC: 98.5%.

2-Chloro-*N*-[(2-chloro-5-sulfamoylphenyl)methyl]pyrimidine-4-carboxamide (58)

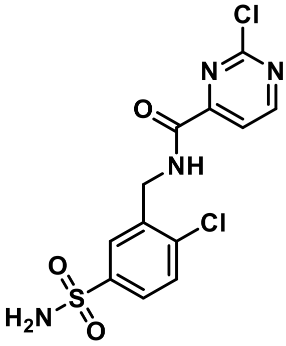

To a solution of ethylbis(propan-2-yl)azanium 4-chloro-3-[[(2-chloropyrimidin-4-yl)formamido]methyl]benzene-1-sulfonate (**57**, 433 mg, 0.88 mmol, 1.0 equiv.) in DCM (6 mL) and DMF (0.2 mL), $SOCl_2$ was added (3.13 mL, 44.0 mmol, 50.0 equiv.) and the mixture was stirred at 25 °C for 1 h. The volatiles were removed under reduced pressure. Ammonia solution (25% in H_2O, 18 mL) was added, and white precipitation appeared immediately. The mixture was allowed to stir at 25 °C for 0.5 h, then it was filtered and washed with H_2O (2 × 10 mL). The residue was purified by reversed-phase flash column chromatography using eluents A (0.1% HCOOH in MeCN) and B (0.1% HCOOH in H_2O) (gradient from 10:90 to 80:20). The product 2-chloro-*N*-[(2-chloro-5-sulfamoylphenyl)methyl]pyrimidine-4-carboxamide (**58**) was obtained as a white solid (174 mg, 55% yield). Mp.: 120–121 °C; ^{1}H NMR (300 MHz, DMSO-*d*$_6$) δ 9.70 (s, 1H), 9.05 (d, J = 4.6 Hz, 1H), 8.06 (d, J = 4.3 Hz, 1H), 7.81 – 7.66 (m, 3H), 7.46 (s, 2H), 4.61 (d, J = 5.6 Hz, 2H); ^{13}C NMR (126 MHz, DMSO-*d*$_6$) δ 163.01, 161.88, 159.63, 159.51, 143.11, 136.79, 135.20, 129.78, 125.83, 125.45, 117.94, 40.57; HRMS (ESI$^-$) *m/z* [M–H]$^-$, calcd. for $C_{12}H_9N_4O_3SCl_2$: 358.9772, found: 358.9759; Purity by HPLC: 97.3%.

***N*-[(2-Bromo-5-sulfamoylphenyl)methyl]-2-cyano-6-methylpyrimidine-4-carboxamide (59)**

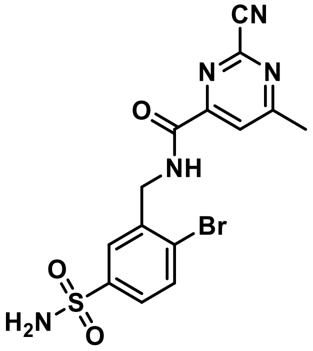

To a solution of *N*-[(2-bromo-5-sulfamoylphenyl)methyl]-2-chloro-6-methylpyrimidine-4-carboxamide (**62**, 8 mg, 0.02 mmol, 1.0 equiv.) in DMSO (0.8 mL), DABCO (7 mg, 0.06 mmol, 3.0 equiv.) and KCN (2 mg, 0.024 mmol, 1.4 equiv.) were added and the mixture was allowed to stir at 25 °C for 1 h. The solution was subjected to reversed-phase flash column chromatography using eluents A (0.1% HCOOH in MeCN) and B (0.1% HCOOH in H_2O) (gradient from 10:90 to 80:20). The product *N*-[(2-Bromo-5-sulfamoylphenyl)methyl]-2-cyano-6-methylpyrimidine-4-carboxamide (**59**) was obtained as a white solid (7 mg, 85% yield). Mp.: 195–196 °C; ^{1}H NMR (500 MHz, DMSO-*d$_6$*) δ 9.81 (t, J = 6.0 Hz, 1H), 8.24 (s, 1H), 7.86 (d, J = 8.3 Hz, 1H), 7.72 (s, 1H), 7.65 (d, J = 8.4 Hz, 1H), 7.44 (s, 2H), 4.58 (d, J = 6.0 Hz, 2H), 2.68 (s, 3H); ^{13}C NMR (126 MHz, DMSO-*d$_6$*) δ 172.24, 161.86, 156.90, 143.70, 142.84, 138.30, 133.12, 125.96, 125.66, 125.17, 121.46, 115.81, 43.15, 23.76; HRMS (ESI$^-$) *m/z* [M–H]$^-$, calcd. for $C_{14}H_{11}N_5O_3SBr$: 407.9765, found: 407.9748; Purity by HPLC: 99.7%.

Ethylbis(propan-2-yl)azanium 4-bromo-3[[(2-chloro-6-methylpyrimidin-4-yl)formamido]methyl]benzene-1- sulfonate (61)

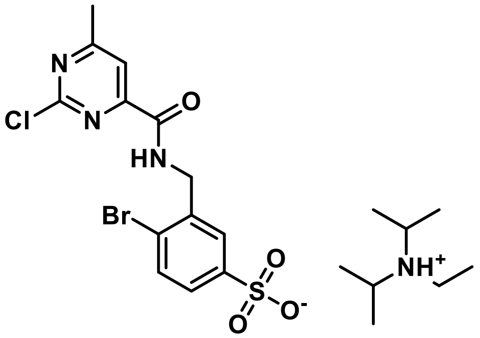

To a stirred solution of 6-methyl-2-chloropyrimidine-4-carboxylic acid (**60**, 56 mg, 0.32 mmol, 1.2 equiv.), HATU (123 mg, 0.32 mmol, 1.2 equiv.) and DIPEA (150 µL, 0.86 mmol, 3.2 equiv.) in DMF (3.0 mL), 3-(aminomethyl)-4-bromobenzene-1-sulfonic acid (**19**, 72 mg, 0.27

mmol, 1.0 equiv.) was added and the mixture was allowed to stir at 25 °C for 1 h. The mixture was purified by reversed-phase flash column chromatography using eluents MeCN and H_2O (gradient from 5:95 to 50:50). The product ethylbis(propan-2-yl)azanium 4-bromo-3[[(2-chloro-6-methylpyrimidin-4-yl)formamido]methyl]benzene-1- sulfonate (**61**) was obtained as a light yellow oil (79 mg, 70% yield). ^{1}H NMR (500 MHz, DMSO-d_6) δ 9.59 (t, J = 6.2 Hz, 1H), 8.27 – 8.12 (m, 1H), 7.97 (s, 1H), 7.57 (d, J = 8.2 Hz, 1H), 7.53 (d, J = 1.9 Hz, 1H), 7.42 (dd, J = 8.2, 2.0 Hz, 1H), 4.52 (d, J = 6.2 Hz, 2H), 3.64 – 3.57 (m, 2H), 3.13 (qd, J = 7.3, 4.3 Hz, 2H), 2.60 (s, 3H), 1.27 – 1.22 (m, 15H); ^{13}C NMR (126 MHz, DMSO-d_6) δ 173.89, 161.84, 159.14, 158.91, 147.89, 136.61, 131.67, 126.02, 125.24, 121.91, 117.55, 53.59, 42.94, 41.83, 23.74, 18.04, 16.69, 12.39; HRMS (ESI$^-$) *m/z* [M–H]$^-$, calcd. for $C_{13}H_{10}N_3O_4SClBr$: 417.9263, found: 417.9231; Purity by HPLC: 98.3%.

_N_-[(2-Bromo-5-sulfamoylphenyl)methyl]-2-chloro-6-methylpyrimidine-4-carboxamide (62)

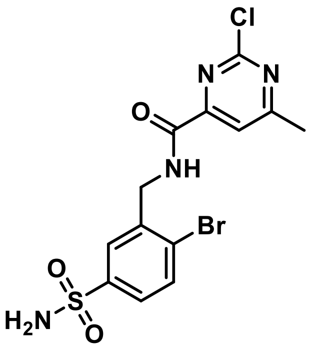

To a solution of ethylbis(propan-2-yl)azanium 4-bromo-3[[(2-chloro-6-methylpyrimidin-4-yl)formamido]methyl]benzene-1- sulfonate (**61**, 93 mg, 0.17 mmol, 1.0 equiv.) in DCM (4 mL) and DMF (0.15 mL), $SOCl_2$ was added (605 µL, 8.5 mmol, 50.0 equiv.) and the mixture was stirred at 25 °C for 0.5 h. The volatiles were removed under reduced pressure. Ammonia solution (25% in H_2O, 10 mL) was added, and white precipitation appeared immediately. The mixture was allowed to stir at 25 °C for 0.5 h, then it was filtered and washed with H_2O (2 × 5 mL). The residue was purified by reversed-phase flash column chromatography using eluents A (0.1% HCOOH in MeCN) and B (0.1% HCOOH in H_2O) (gradient from 10:90 to 80:20). The product *N*-[(2-bromo-5-sulfamoylphenyl)methyl]-2-chloro-6-methylpyrimidine-4-carboxamide (**62**) was obtained as a white solid (30 mg, 41% yield). Mp.: 115–116 °C; ^{1}H NMR (500 MHz, DMSO-d_6) δ 9.62 (t, J = 6.1 Hz, 1H), 7.98 (s, 1H), 7.85 (d, J = 8.3 Hz, 1H), 7.71 (d, J = 1.9 Hz, 1H), 7.65 (dd, J = 8.3, 2.1 Hz, 1H), 7.45 (s, 2H), 4.57 (d, J = 6.1 Hz, 2H), 2.61 (s, 3H); ^{13}C NMR (126 MHz, DMSO-d_6) δ 173.87, 162.07, 159.15, 158.91, 143.69, 138.40,

133.08, 125.92, 125.61, 125.08, 117.52, 43.11, 23.75; HRMS (ESI$^-$) *m/z* [M–H]$^-$, calcd. for $C_{13}H_{11}N_4O_3SClBr$: 416.9423, found: 416.9410; Purity by HPLC: 97.4%.

2-Cyano-*N*-[(5-sulfamoyl-2-[[(thiophen-2-yl)methyl]amino]phenyl)methyl]pyrimidine-4-carboxamide (63)

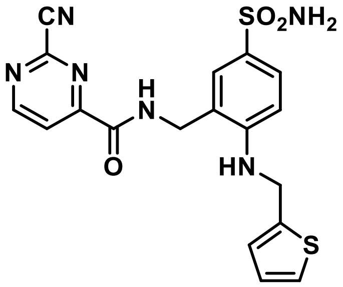

To a solution of 2-chloro-*N*-[(5-sulfamoyl-2-[[(thiophen-2-yl)methyl]amino]phenyl)methyl]pyrimidine-4-carboxamide (**67**, 33 mg, 0.08 mmol, 1.0 equiv.) in DMSO (1.8 mL), DABCO (26 mg, 0.24 mmol, 3.0 equiv.) and KCN (3 mg, 0.112 mmol, 1.4 equiv.) were added and the mixture was allowed to stir at 25 °C for 1 h. The solution was subjected to reversed-phase flash column chromatography using eluents A (0.1% HCOOH in MeCN) and B (0.1% HCOOH in H_2O) (gradient from 10:90 to 80:20). The product 2-cyano-*N*-[(5-sulfamoyl-2-[[(thiophen-2-yl)methyl]amino]phenyl)methyl]pyrimidine-4- carboxamide (**63**) was obtained as a light yellow solid (22 mg, 68% yield). Mp.: 108–110 °C; ^{1}H NMR (500 MHz, DMSO-*d*$_6$) δ 9.77 (t, J = 6.3 Hz, 1H), 9.26 (d, J = 5.1 Hz, 1H), 8.30 (d, J = 5.1 Hz, 1H), 7.61 (d, J = 2.2 Hz, 1H), 7.49 (dd, J = 8.6, 2.2 Hz, 1H), 7.32 (dd, J = 5.1, 1.2 Hz, 1H), 7.08 – 7.05 (m, 1H), 6.94 (t, J = 4.2 Hz, 3H), 6.75 (dd, J = 13.7, 7.3 Hz, 2H), 4.64 (d, J = 5.6 Hz, 2H), 4.44 (d, J = 6.3 Hz, 2H); ^{13}C NMR (126 MHz, DMSO-*d*$_6$) δ 161.55, 161.35, 157.49, 147.86, 143.27, 143.14, 130.73, 127.16, 126.73, 126.51, 125.04, 124.59, 121.67, 121.60, 115.77, 109.64, 41.48, 39.78; HRMS (ESI$^+$) *m/z* [M+H]$^+$, calcd. for $C_{18}H_{17}N_6O_3S_2$: 429.0803, found: 429.0809; Purity by HPLC: 98.4%.

3-Cyano-4-[[(thiophen-2-yl)methyl]amino]benzene-1-sulfonamide (65)

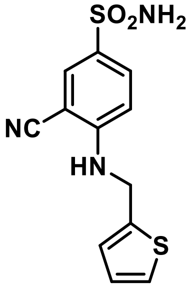

To a solution of 3-cyano-4-fluorobenzensulfonamide (**33**, 400 mg, 2.00 mmol, 1.0 equiv.) in DMF (4.0 mL) and DIPEA (418 µL, 2.40 mmol, 1.2 equiv.), 2-aminomethyltiophene (**64**, 410 µL, 4.00 mmol, 2.0 equiv.) was added and the mixture was allowed to stir at 25 °C for 16 h. The solution was subjected to reversed-phase flash column chromatography using eluents A (0.1% HCOOH in MeCN) and B (0.1% HCOOH in H_2O) (gradient from 10:90 to 80:20). The product 3-cyano-4-[[(thiophen-2-yl)methyl]amino]benzene-1-sulfonamide **(65**) was obtained as a white solid (532 mg, 91% yield). Mp.: 152–153 °C; ^{1}H NMR (500 MHz, DMSO-*d₆*) δ 7.85 (d, J = 2.1 Hz, 1H), 7.73 (dd, J = 9.0, 2.0 Hz, 1H), 7.58 (t, J = 6.0 Hz, 1H), 7.40 – 7.36 (m, 1H), 7.15 (s, 2H), 7.09 (d, J = 2.8 Hz, 1H), 6.97 (dd, J = 4.9, 3.6 Hz, 1H), 6.94 (d, J = 9.1 Hz, 1H), 4.68 (d, J = 6.0 Hz, 2H); ^{13}C NMR (126 MHz, DMSO-d_6) δ 151.68, 141.99, 131.59, 131.47, 131.37, 126.81, 125.51, 124.98, 116.75, 111.60, 93.90, 41.16; HRMS (ESI$^-$) *m/z* [M–H]$^-$, calcd. for $C_{12}H_{10}N_3O_2S_2$: 292.0214, found: 292.0204; Purity by HPLC: 99.7%.

3-(Aminomethyl)-4-[[(thiophen-2-yl)methyl]amino]benzene-1-sulfonamide (66)

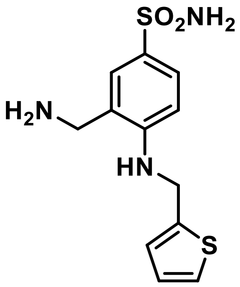

3-Cyano-4-[[(thiophen-2-yl)methyl]amino]benzene-1-sulfonamide **(65**, 370 mg, 1.26 mmol) was dissolved in a mixture of ammonia solution (4 M in MeOH, 30 mL) of H_2O (6 mL). Raney nickel (W. R. Grace and Co. Raney® 2800 slurry, in H_2O, active catalyst; 400 mg) was added and the reactor was filled with H_2 (10 bar). The mixture was heated to 100 °C and it was stirred at this temperature for 16 h. It was then filtered through a pad of Celite, washed with MeOH (3 × 20 mL) and water (2 × 10 mL) then the volatiles evaporated. The solution was subjected to reversed-phase flash column chromatography using eluents A (0.1% HCOOH in MeCN) and B (0.1% HCOOH in H_2O) (gradient from 10:90 to 60:40). The product 3-(aminomethyl)-4-[[(thiophen-2-yl)methyl]amino]benzene-1-sulfonamide (**66**) was obtained as a white solid (188 mg, 50% yield). Mp.: 105–107 °C; ^{1}H NMR (500 MHz, DMSO-d_6) δ 7.56 (d, J = 2.1 Hz, 1H), 7.49 (dd, J = 8.5, 2.2 Hz, 1H), 7.36 (d, J = 5.0 Hz, 1H), 7.19 (d, J = 20.4 Hz, 1H), 7.07 (d, J = 3.3 Hz, 1H), 6.97 (dd, J = 5.0, 3.5 Hz, 1H), 6.68 (dd, J = 8.6, 2.9 Hz, 1H), 4.61 (s, 2H), 3.79 (s, 2H); ^{13}C NMR (126 MHz, DMSO-d_6) δ 149.12, 143.35, 130.62, 126.81, 125.99, 125.93,

125.52, 124.95, 124.57, 109.08, 43.32, 41.53; HRMS (ESI$^-$) *m/z* [M–H]$^-$, calcd. for $C_{12}H_4N_3O_2S_2$: 296.0527, found: 296.0520; Purity by HPLC: 90.1%.

2-Chloro-*N*-[(5-sulfamoyl-2-[[(thiophen-2-yl)methyl]amino]phenyl)methyl]pyrimidine-4-carboxamide (67)

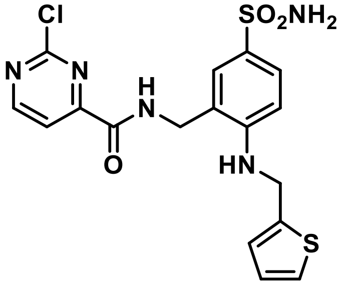

To a stirred solution of 2-chloropyrimidine-4-carboxylic acid (**20**, 42 mg, 0.264 mmol, 1.2 equiv.), HATU (100 mg, 0.264 mmol, 1.2 equiv.) and DIPEA (84 µL, 0.484 mmol, 2.2 equiv.) in DMF (3.0 mL), 3-(aminomethyl)-4-[[(thiophen-2-yl)methyl]amino]benzene-1-sulfonamide (**66**, 65 mg, 0.22 mmol, 1.0 equiv.) was added and the mixture was allowed to stir at 25 °C for 1 h. The mixture was purified by reversed-phase flash column chromatography using eluents A (0.1% HCOOH in MeCN) and B (0.1% HCOOH in H_2O) (gradient from 10:90 to 80:20). The product 2-chloro-*N*-[(5-sulfamoyl-2-[[(thiophen-2-yl)methyl]amino]phenyl)methyl]pyrimidine-4- carboxamide (**67**) was obtained as a light yellow solid (56 mg, 58% yield). Mp.: 88–90 °C; ^{1}H NMR (500 MHz, DMSO-*d*$_6$) δ 9.62 (t, J = 6.0 Hz, 1H), 9.03 (d, J = 4.9 Hz, 1H), 8.05 (d, J = 4.9 Hz, 1H), 7.62 (d, J = 1.8 Hz, 1H), 7.55 – 7.47 (m, 1H), 7.32 (d, J = 4.7 Hz, 1H), 7.07 (d, J = 2.8 Hz, 1H), 7.00 – 6.91 (m, 3H), 6.83 – 6.71 (m, 2H), 4.64 (d, J = 5.4 Hz, 2H), 4.43 (d, J = 6.2 Hz, 2H); ^{13}C NMR (126 MHz, DMSO-*d*$_6$) δ 163.03, 161.84, 159.61, 159.57, 147.89, 143.34, 130.72, 127.17, 126.77, 126.53, 125.07, 124.62, 121.72, 117.91, 109.65, 41.49; HRMS (ESI$^+$) *m/z* [M+H]$^+$, calcd. for $C_{17}H_{17}N_5O_3S_2Cl$: 438.0461, found: 438.0480; Purity by HPLC: 100%.

2-Cyclopropyl-6-methyl-*N*-[(4-sulfamoylphenyl)methyl]pyrimidine-4-carboxamide (68)

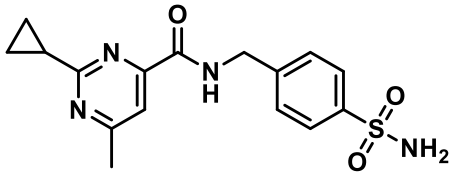

The compound was purchased from MolPort (MolPort-046-942-260), and the purity of it was checked before biochemical evaluation. Purity by HPLC: 98.5%.

***N*-[1-(4-Methanesulfonylphenyl)ethyl]-2,6-dimethylpyrimidine-4-carboxamide (69)**

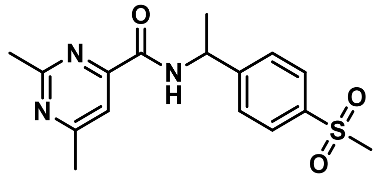

The compound was purchased from MolPort (MolPort-045-938-133), and the purity of it was checked before biochemical evaluation. Purity by HPLC: 99.2%.

2-Methyl-*N*-[(4-sulfamoylphenyl)methyl]-6-(trifluoromethyl)pyrimidine-4-carboxamide (70)

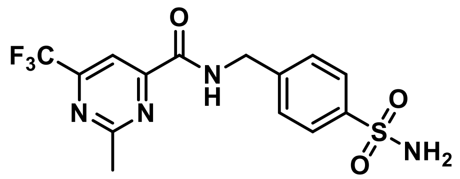

The compound was purchased from MolPort (MolPort-046-929-025), and the purity of it was checked before biochemical evaluation. Purity by HPLC: 98.6%.

Ethylbis(propan-2-yl)azanium 4-bromo-3-[[(pyrimidin-4-yl)formamido]methyl]benzene-1-sulfonate (73)

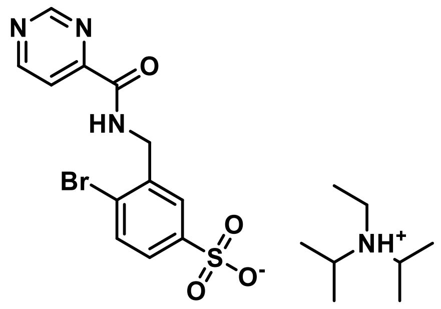

To a stirred solution of pyrimidine-4-carboxylic acid (**72**, 137 mg, 1.10 mmol, 1.2 equiv.), HATU (420 mg, 1.10 mmol, 1.2 equiv.) and DIPEA (513 µL, 2.94 mmol, 3.2 equiv.) in DMF (5.0 mL), 3-(aminomethyl)-4-chlorobenzene-1-sulfonic acid (**19**, 245 mg, 0.92 mmol, 1.0 equiv.) was added and the mixture was allowed to stir at 25 °C for 1 h. The mixture was purified by reversed-phase flash column chromatography using eluents MeCN and H_2O (gradient from 5:95 to 50:50). The product ethylbis(propan-2-yl)azanium 4-bromo-3-[[(pyrimidin-4-yl)formamido]methyl]benzene-1-sulfonate (**73**) was obtained as a yellow oil (141 mg, 41% yield). ^1H NMR (500 MHz, DMSO-*d*$_6$) δ 9.69 (t, J = 5.9 Hz, 1H), 9.39 (s, 1H), 9.10 (d, J = 5.0 Hz, 1H), 8.06 (d, J = 4.7 Hz, 1H), 7.61 – 7.53 (m, 2H), 7.42 (d, J = 8.1 Hz, 1H), 4.54 (d, J = 6.1

Hz, 2H), 3.59 (dt, J = 13.1, 6.5 Hz, 2H), 3.11 (q, J = 7.2 Hz, 2H), 1.25 – 1.19 (m, 15H); ^{13}C NMR (126 MHz, DMSO-d_6) δ 163.00, 159.86, 158.06, 156.12, 147.80, 136.86, 131.81, 126.09, 125.24, 122.07, 118.72, 53.58, 42.92, 41.85, 12.51; HRMS (ESI$^-$) *m/z* [M–H]$^-$, calcd. for $C_{12}H_9N_3O_4SBr$: 369.9497, found: 369.9480; Purity by HPLC: 100%.

9. LC-MS chromatograms and spectra, ^{1}H NMR, and ^{13}C NMR spectra of the compounds

Pyrimidine-2-carbonitrile (1)

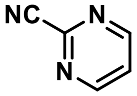

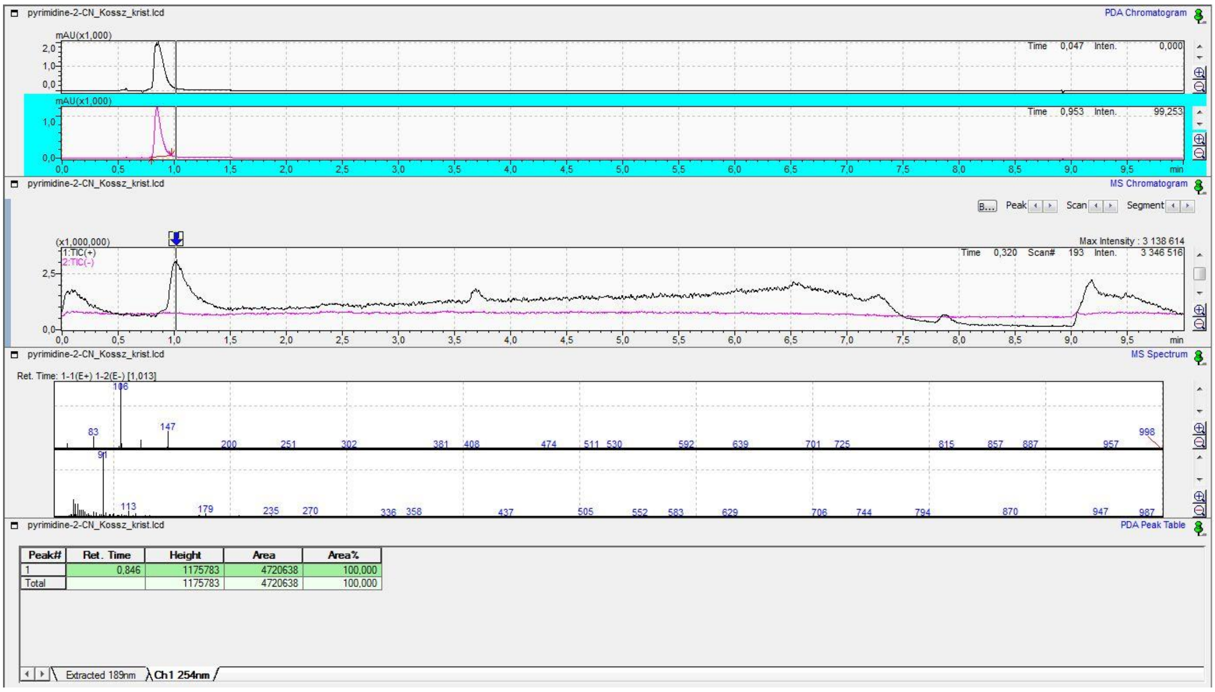

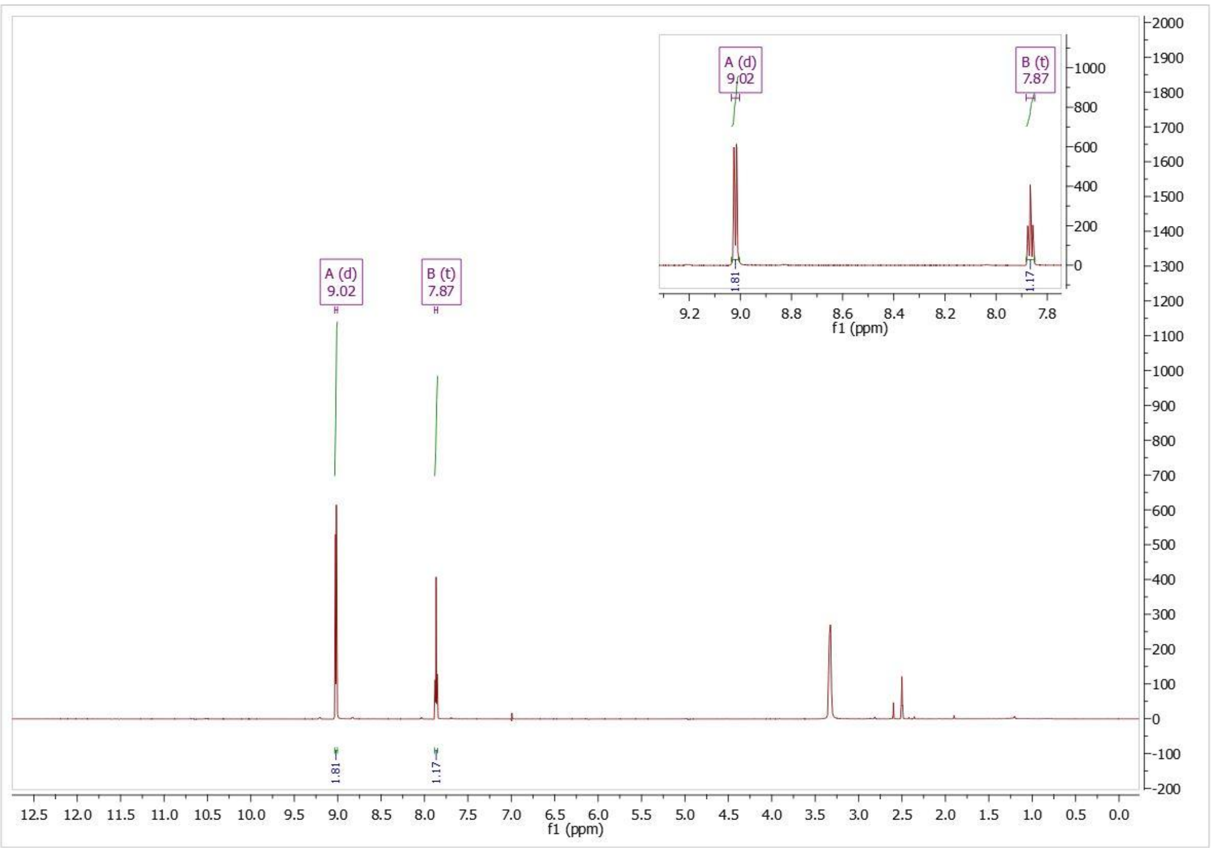
A (d)
9.02
B (t)
7.87
1.81
1.17
f1 (ppm)

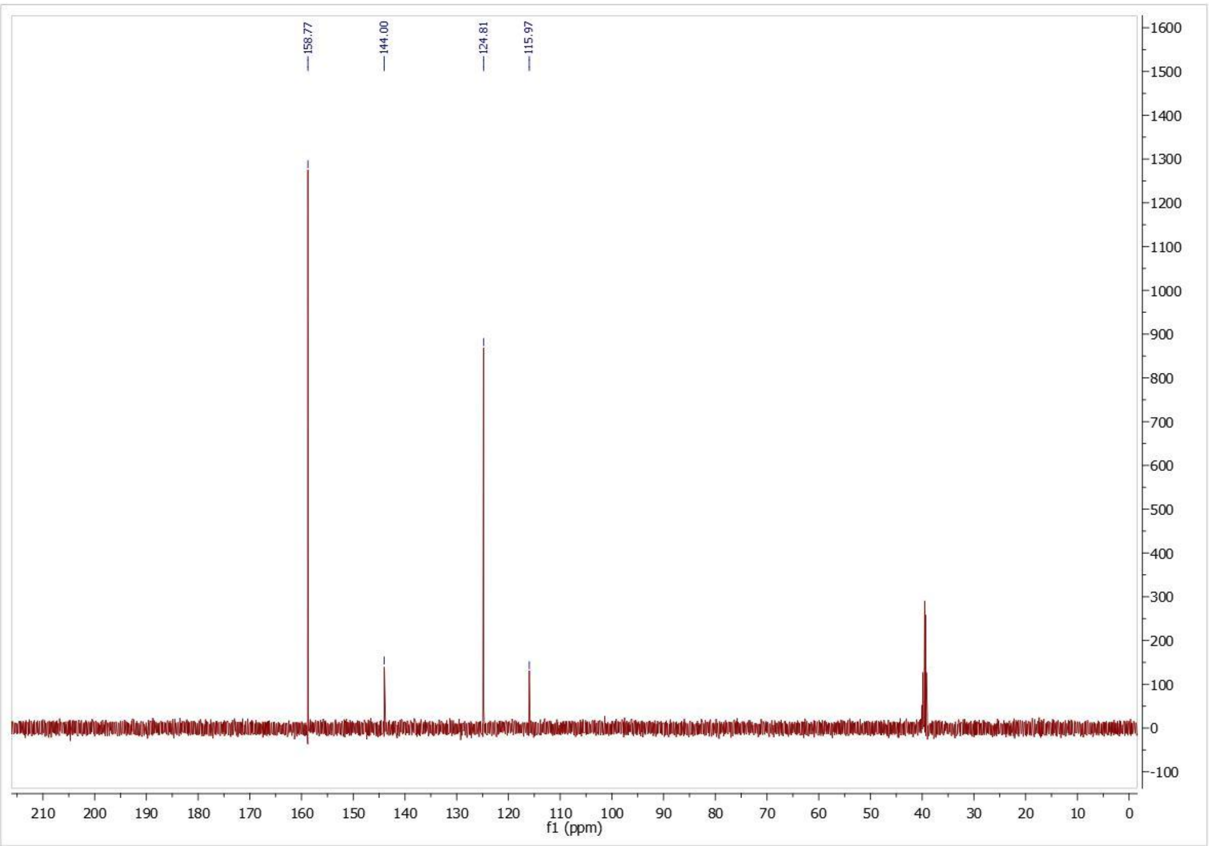
158.77
144.00
124.81
115.97
f1 (ppm)

4-Bromobenzene-1-sulfonamide (2)

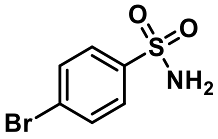

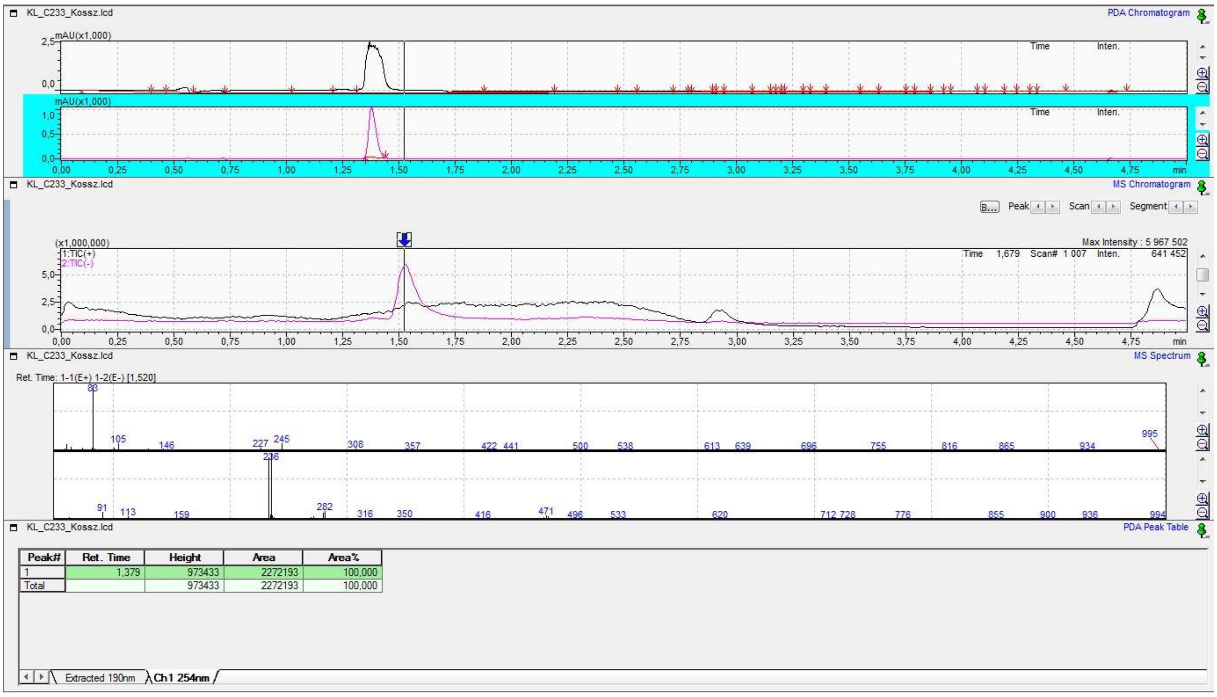

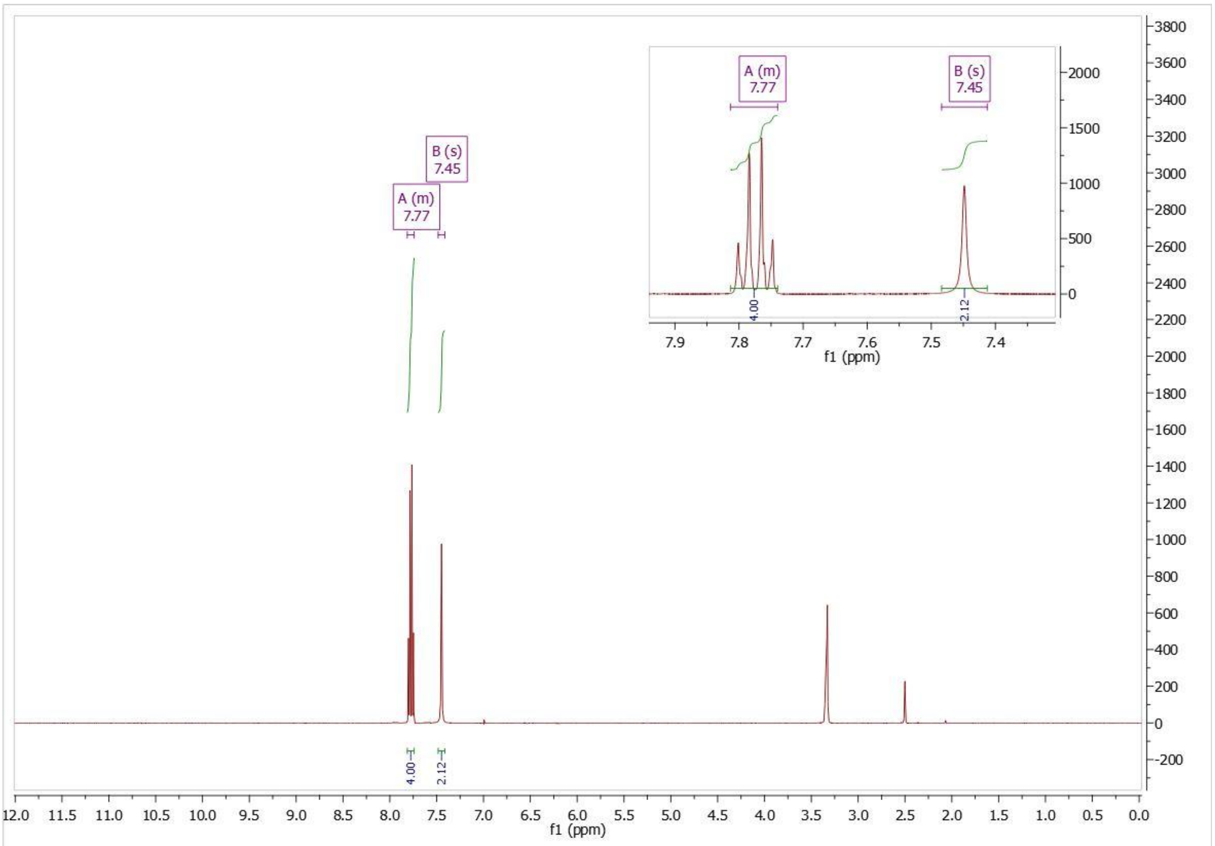

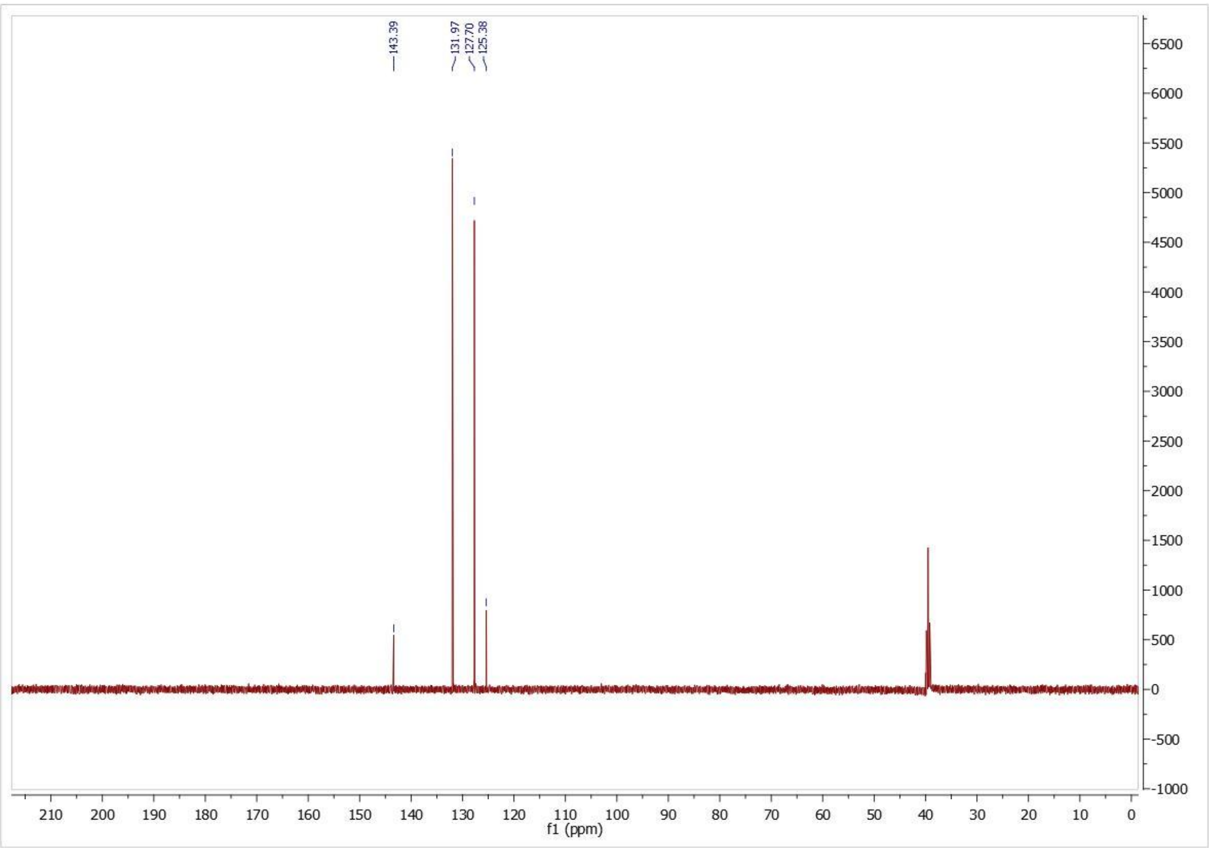

143.39
131.97
127.70
125.38
6500
6000
5500
5000
4500
4000
3500
3000
2500
2000
1500
1000
500
0
-500
-1000
210 200 190 180 170 160 150 140 130 120 110 100 90 80 70 60 50 40 30 20 10 0
f1 (ppm)

N-(2-bromo-5-sulfamoylphenyl)-2-(2-cyanopyrimidin-4-yl)acetamide (3)

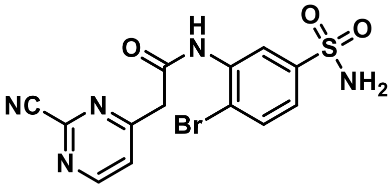

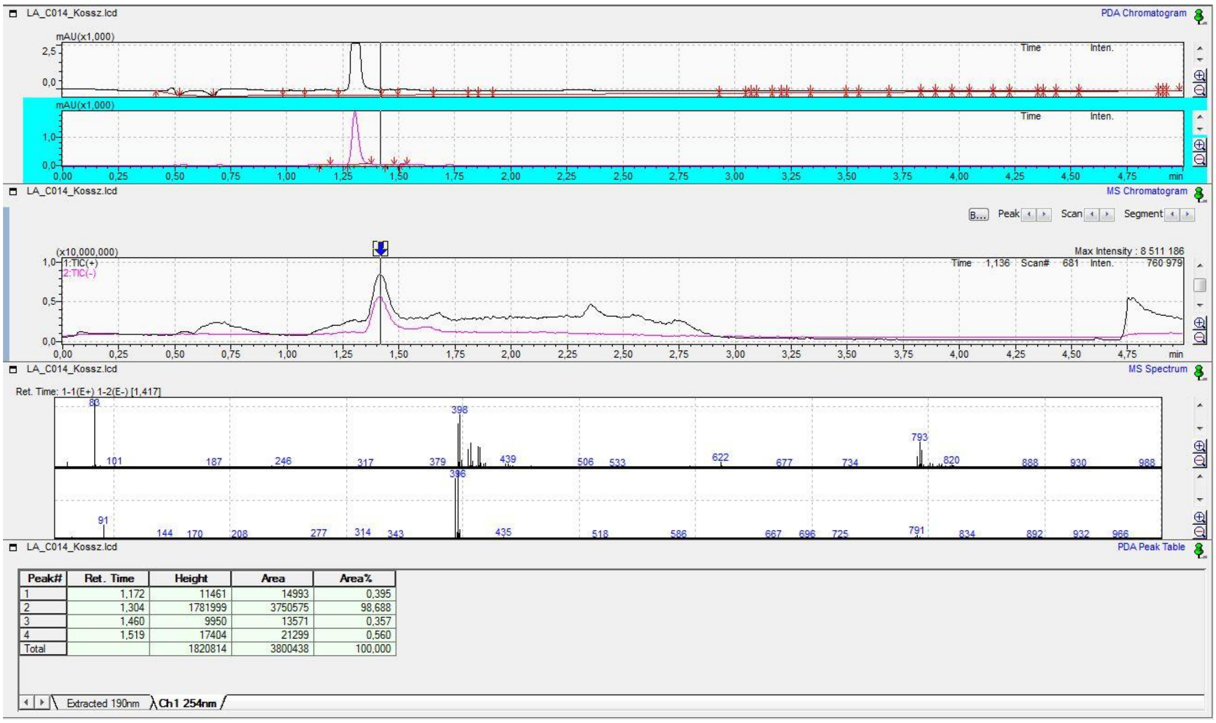

Peak#	Ret. Time	Height	Area	Area%
1	1,172	11461	14993	0,395
2	1,304	1781999	3750575	98,688
3	1,460	9950	13571	0,357
4	1,519	17404	21299	0,560
Total		1820814	3800438	100,000

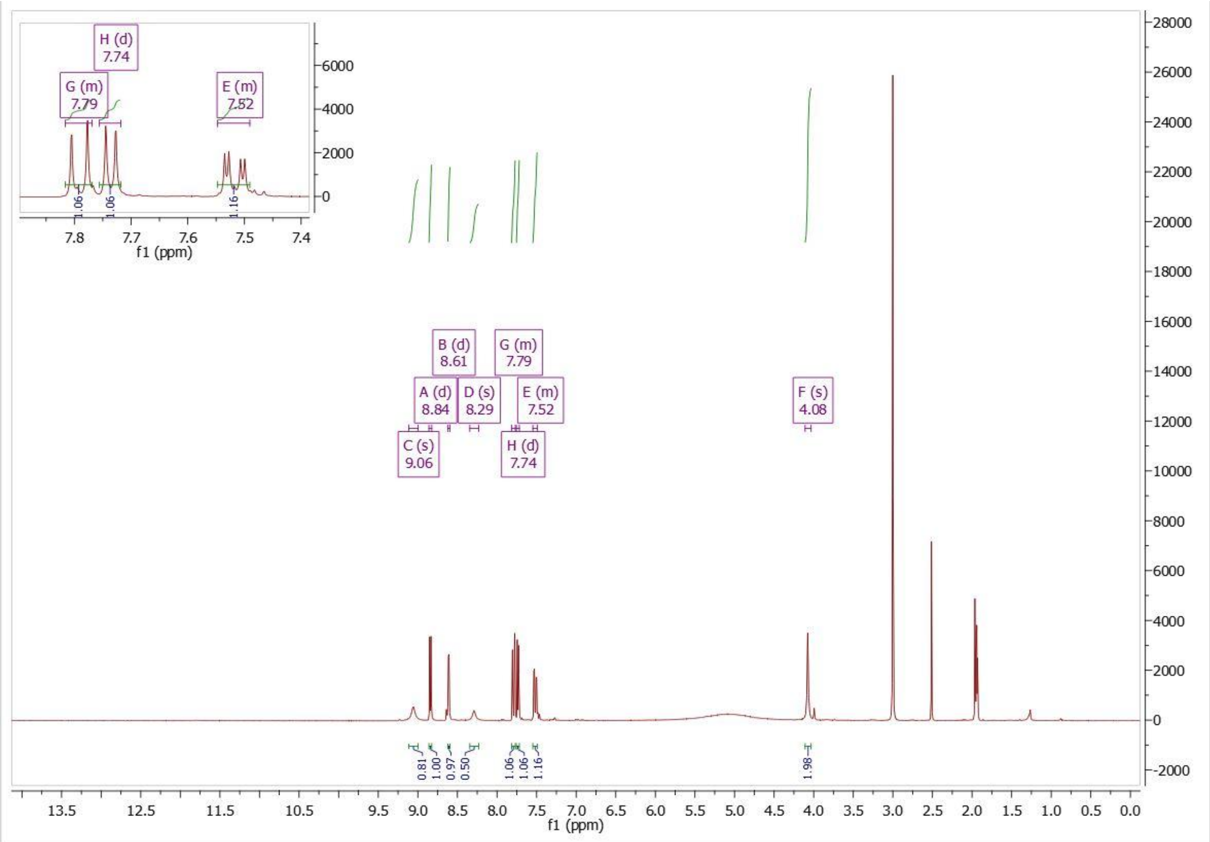

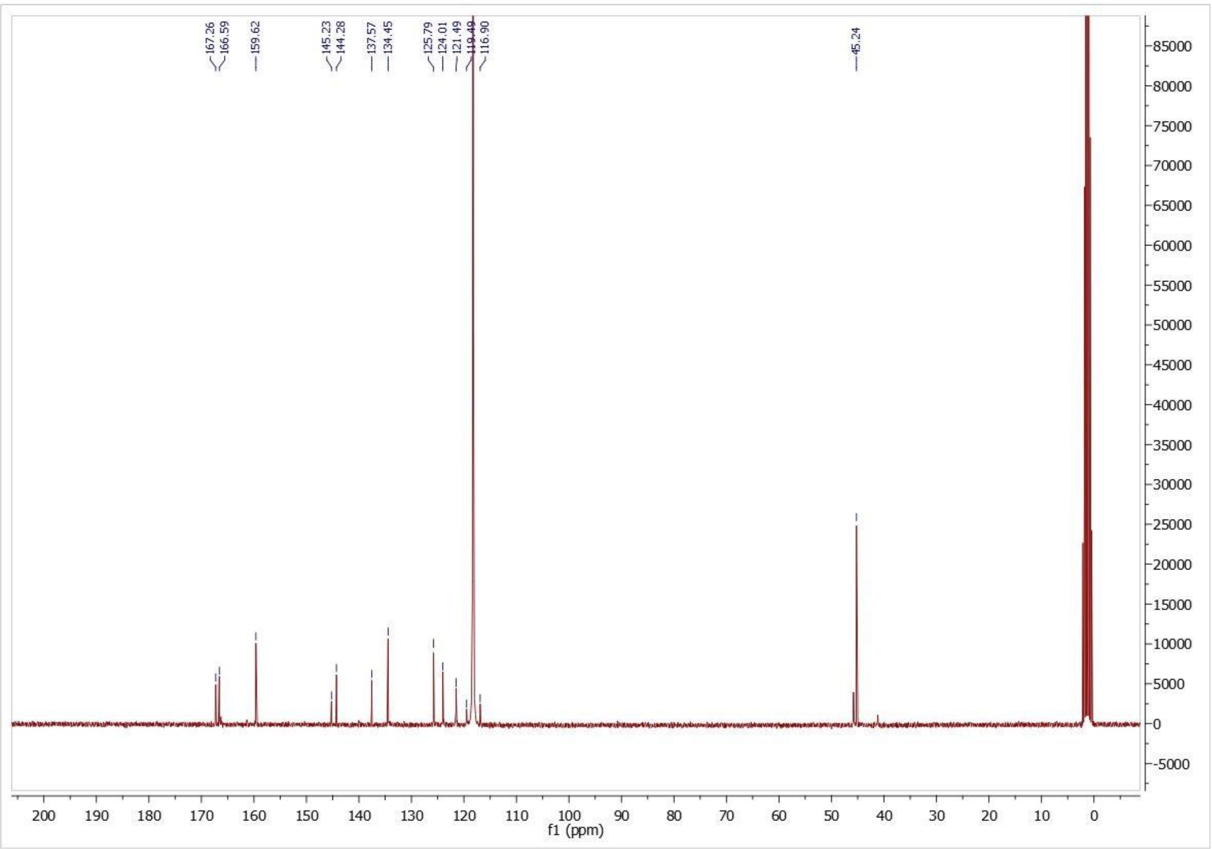

***N*-(2-bromo-5-sulfamoylphenyl)-2-(2-cyanopyrimidin-4-yl)-*N*-methylacetamide (4)**

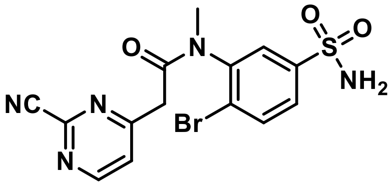

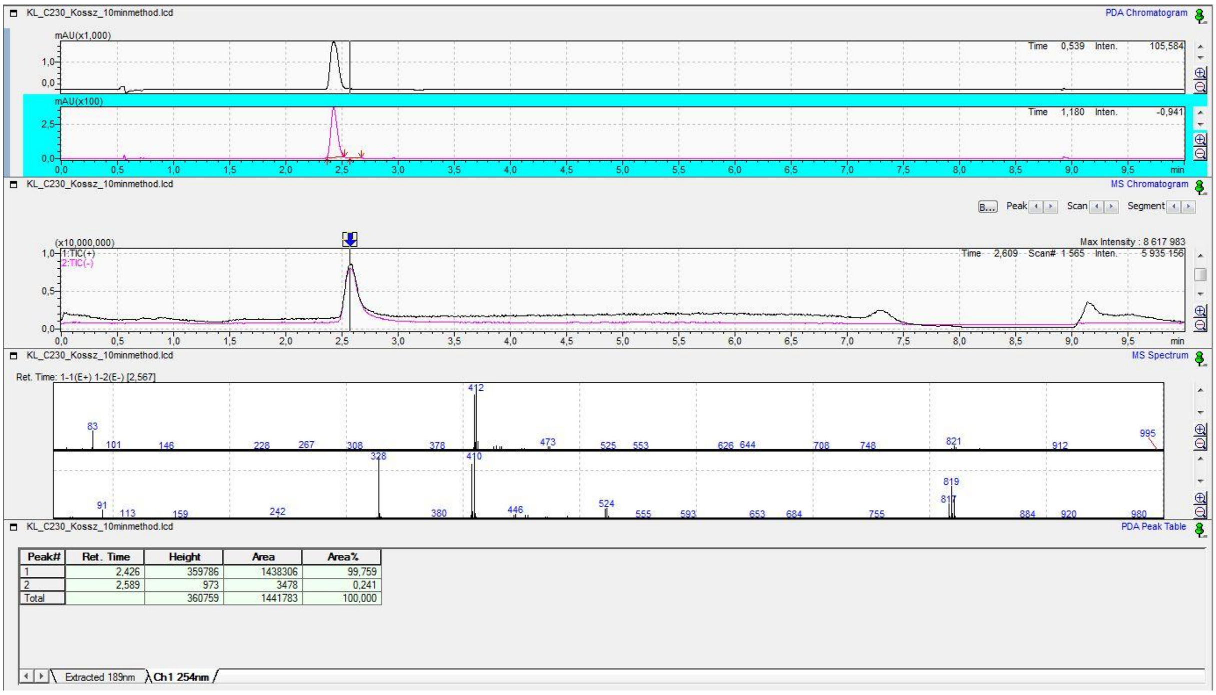

Peak#	Ret. Time	Height	Area	Area%
1	2,426	359786	1438306	99,759
2	2,589	973	3478	0,241
Total		360759	1441783	100,000

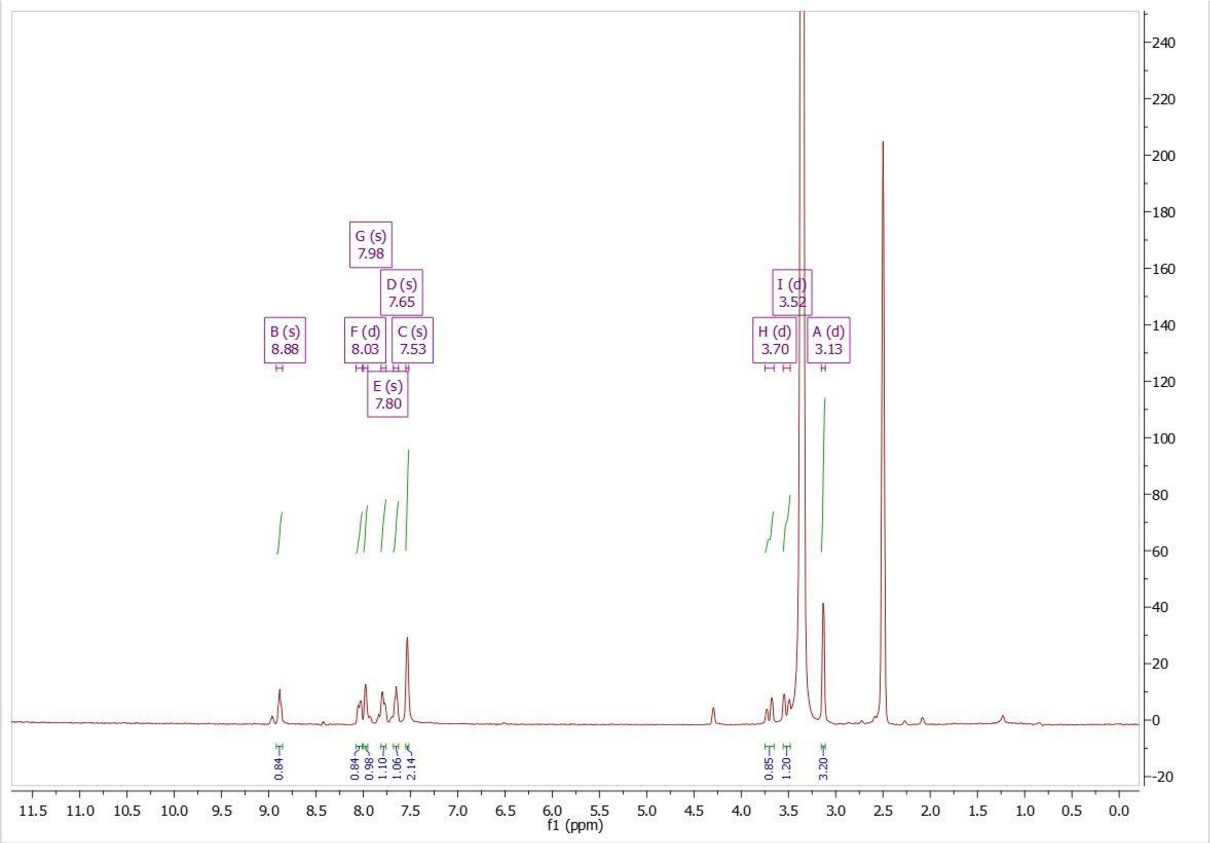

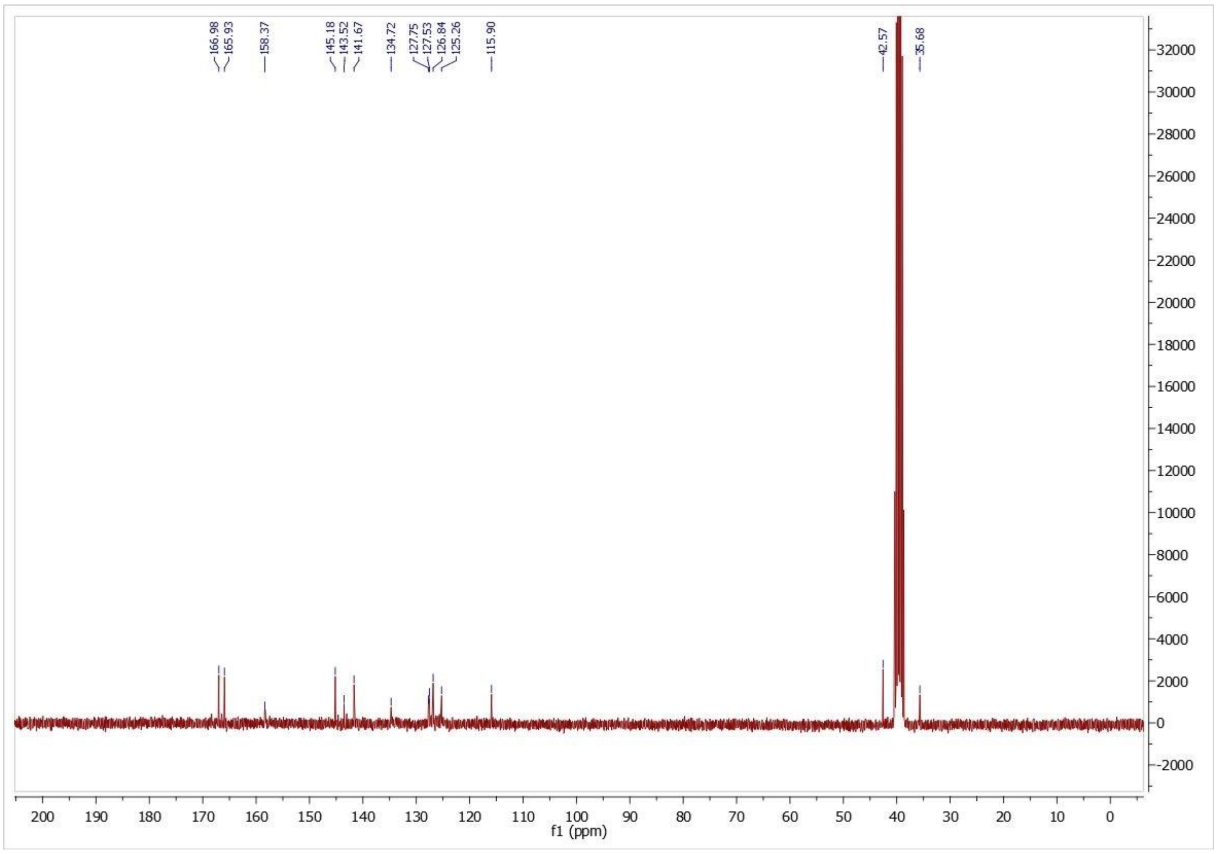

N-[(2-bromo-5-sulfamoylphenyl)methyl]-2-cyanopyrimidine-4-carboxamide (5)

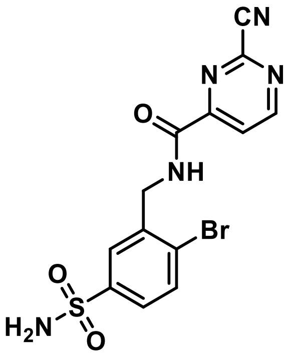

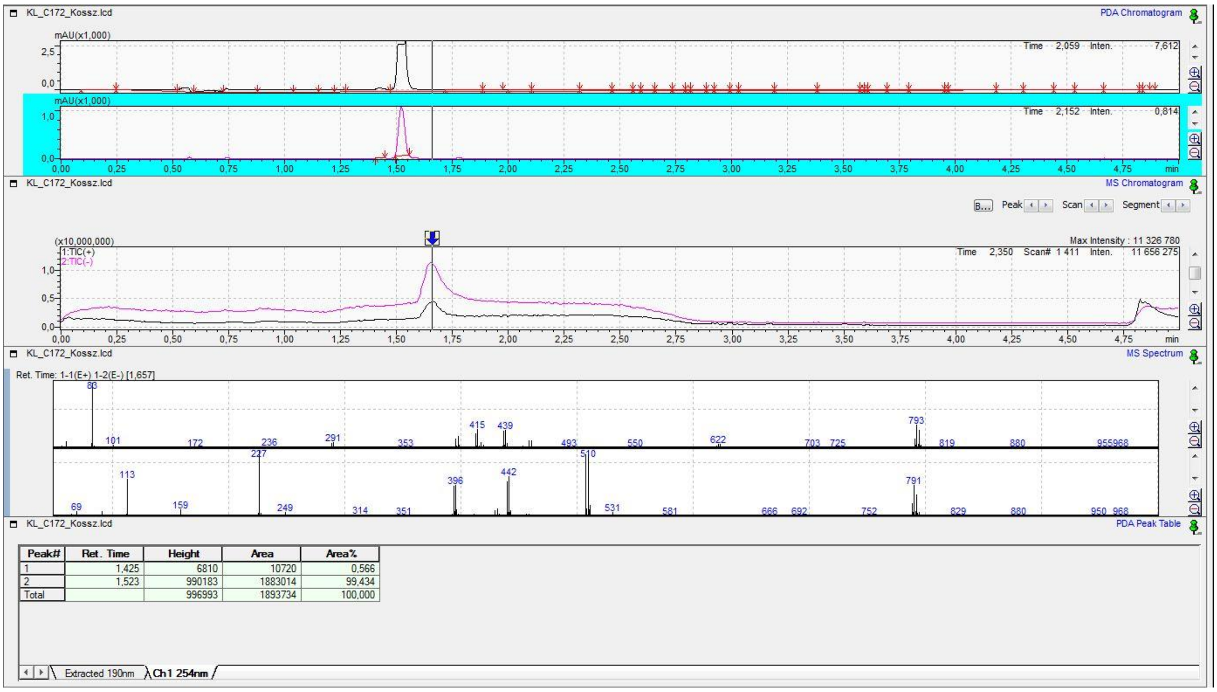
KL_C172_Kossz.lcd
PDA Chromatogram
mAU(x1,000)
MS Chromatogram
MS Spectrum
Ret. Time: 1-1(E+) 1-2(E-) [1,657]
PDA Peak Table
Peak# Ret. Time Height Area Area%
1 1,425 6810 10720 0,566
2 1,523 990183 1883014 99,434
Total 996993 1893734 100,000
Extracted 190nm
Ch1 254nm

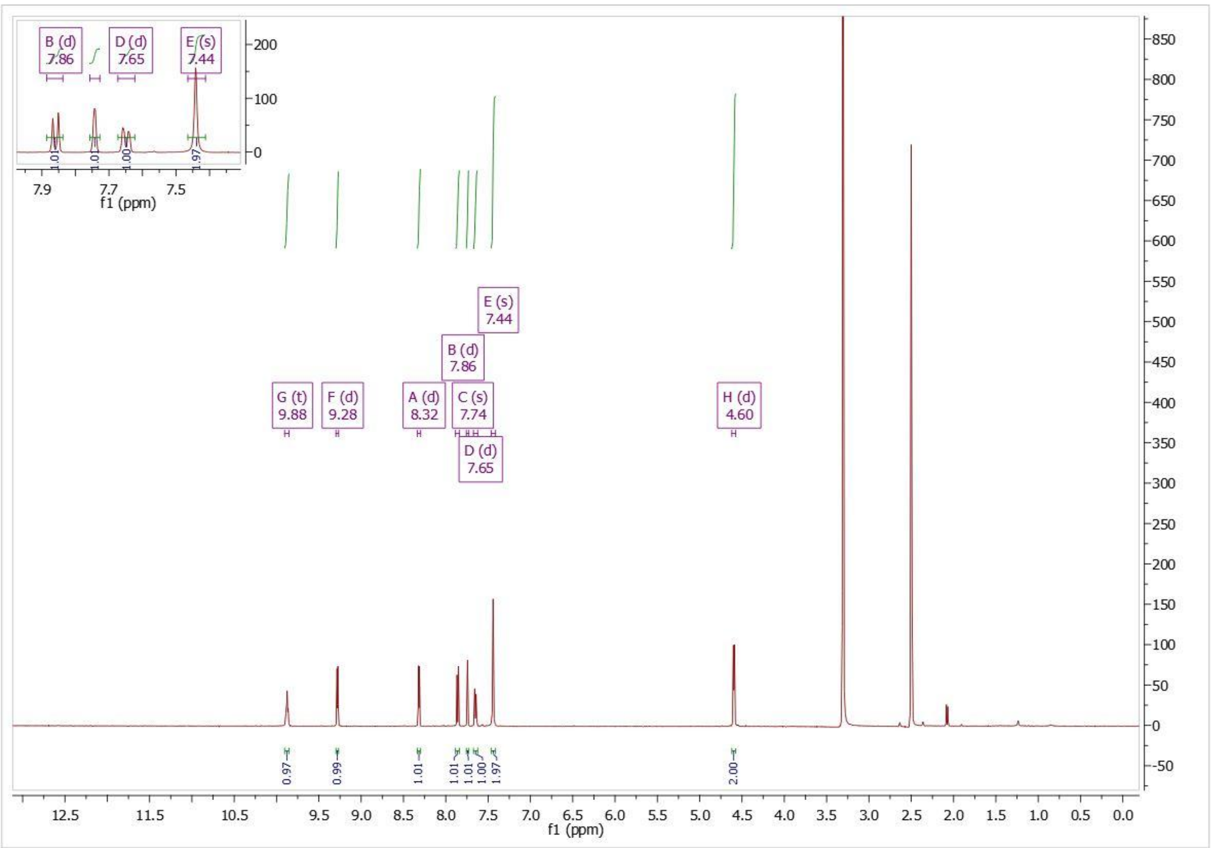
B (d) 7.86
D (d) 7.65
E (s) 7.44
G (t) 9.88
F (d) 9.28
A (d) 8.32
C (s) 7.74
H (d) 4.60
f1 (ppm)

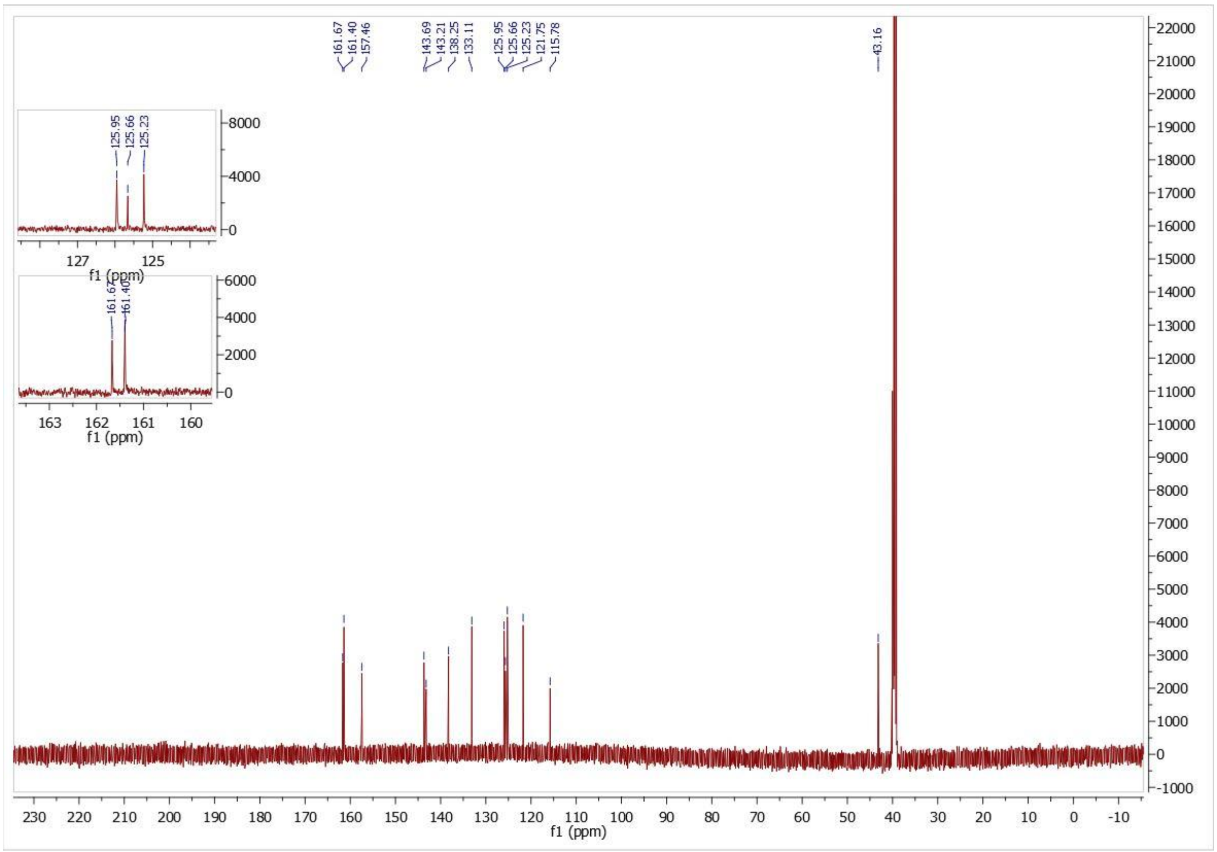

4-Bromo-3-nitrobenzene-1-sulfonyl chloride (7)

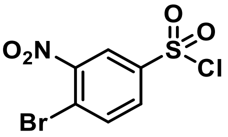

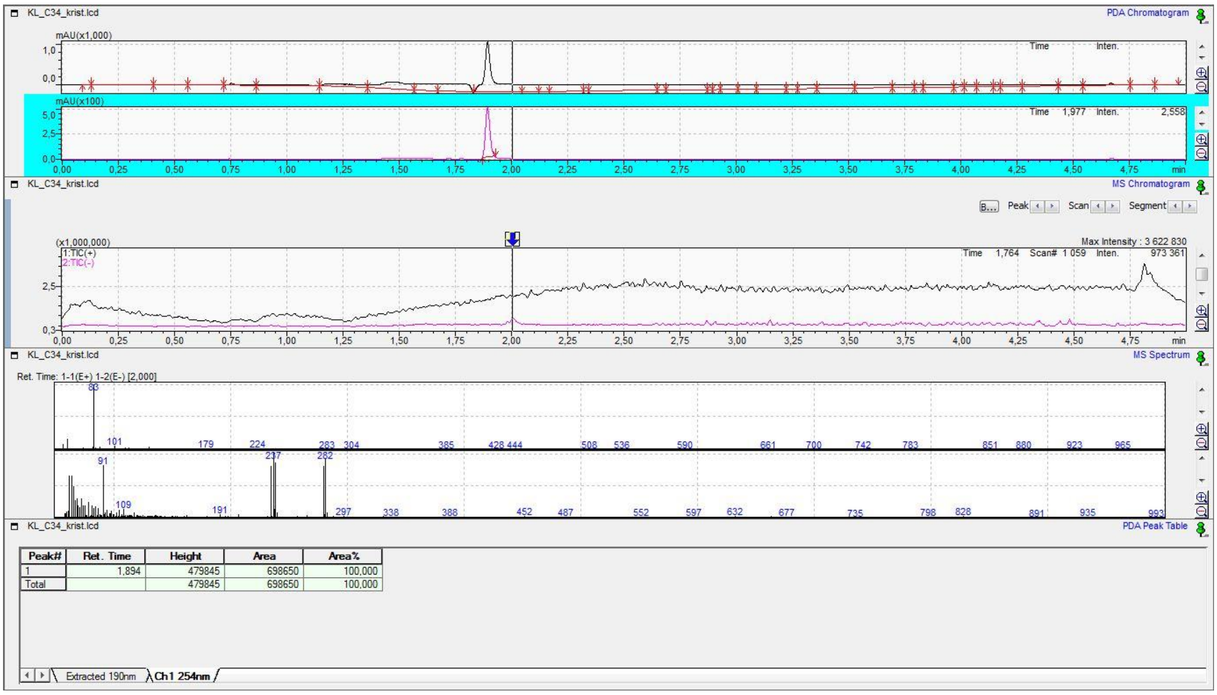

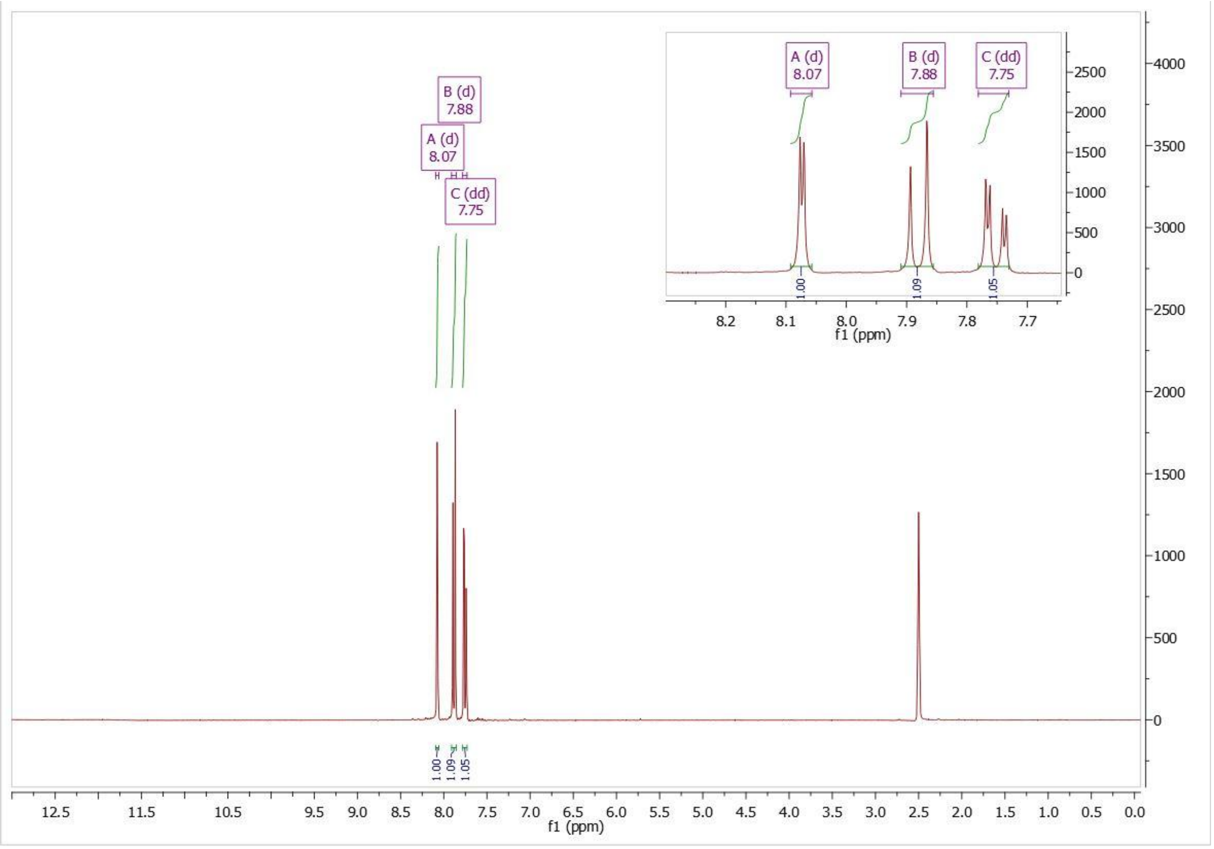

B (d)
7.88
A (d)
8.07
C (dd)
7.75
1.00
1.09
1.05
f1 (ppm)

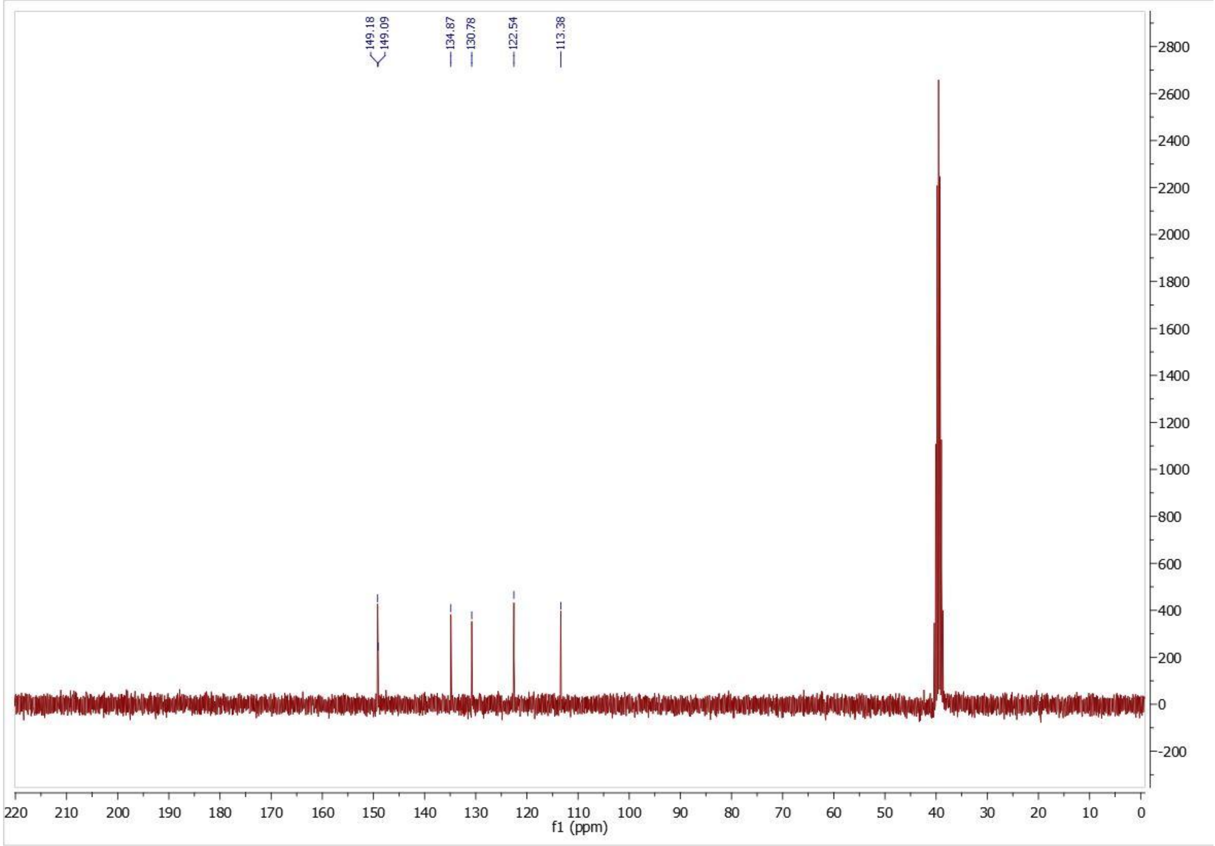

149.18
149.09
134.87
130.78
122.54
113.38
f1 (ppm)

4-Bromo-3-nitrobenzene-1-sulfonamide (8)

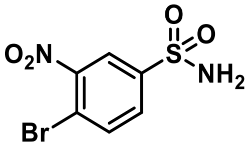

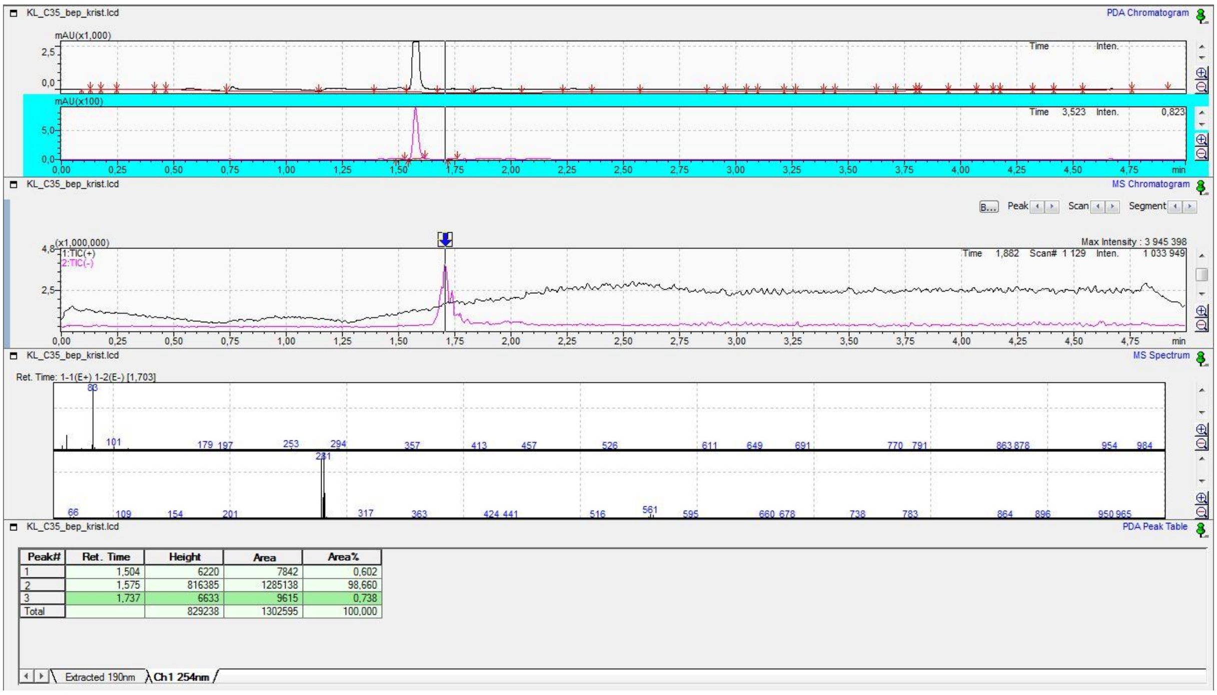

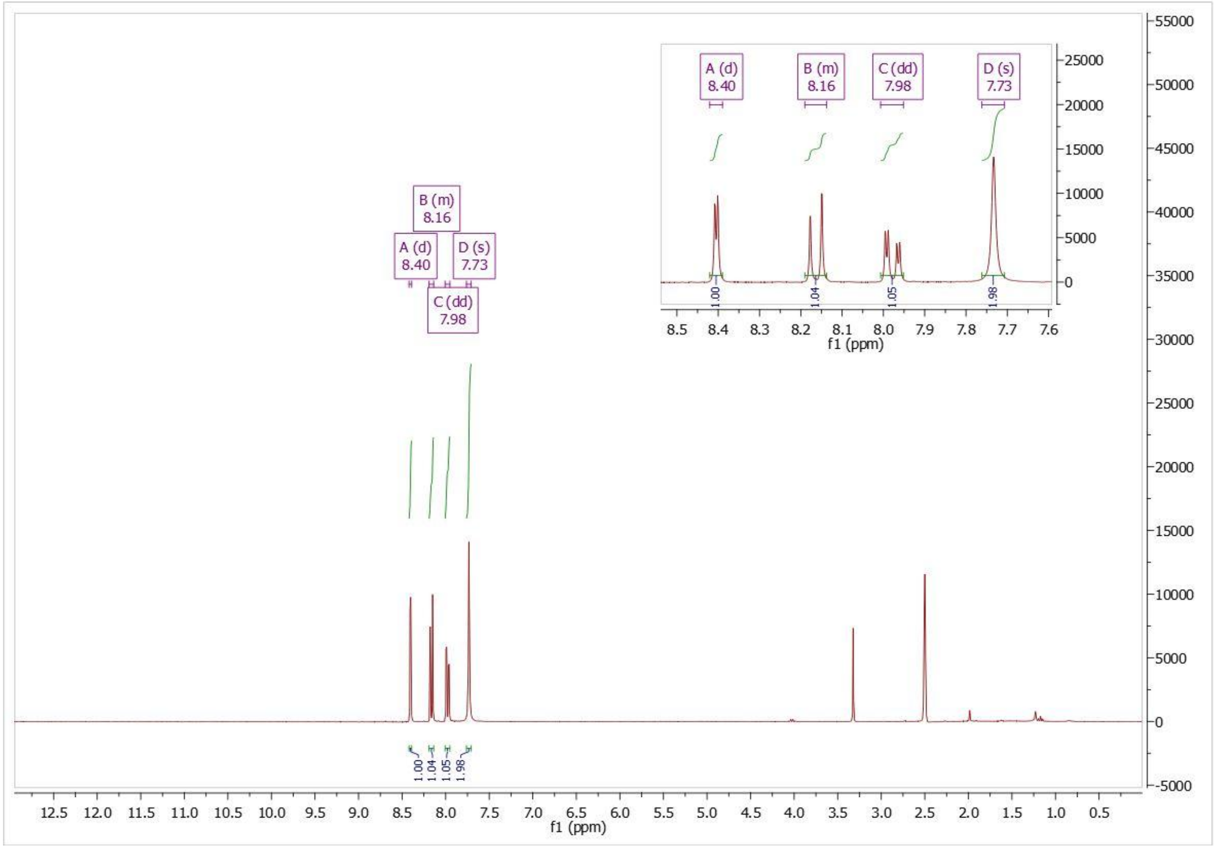

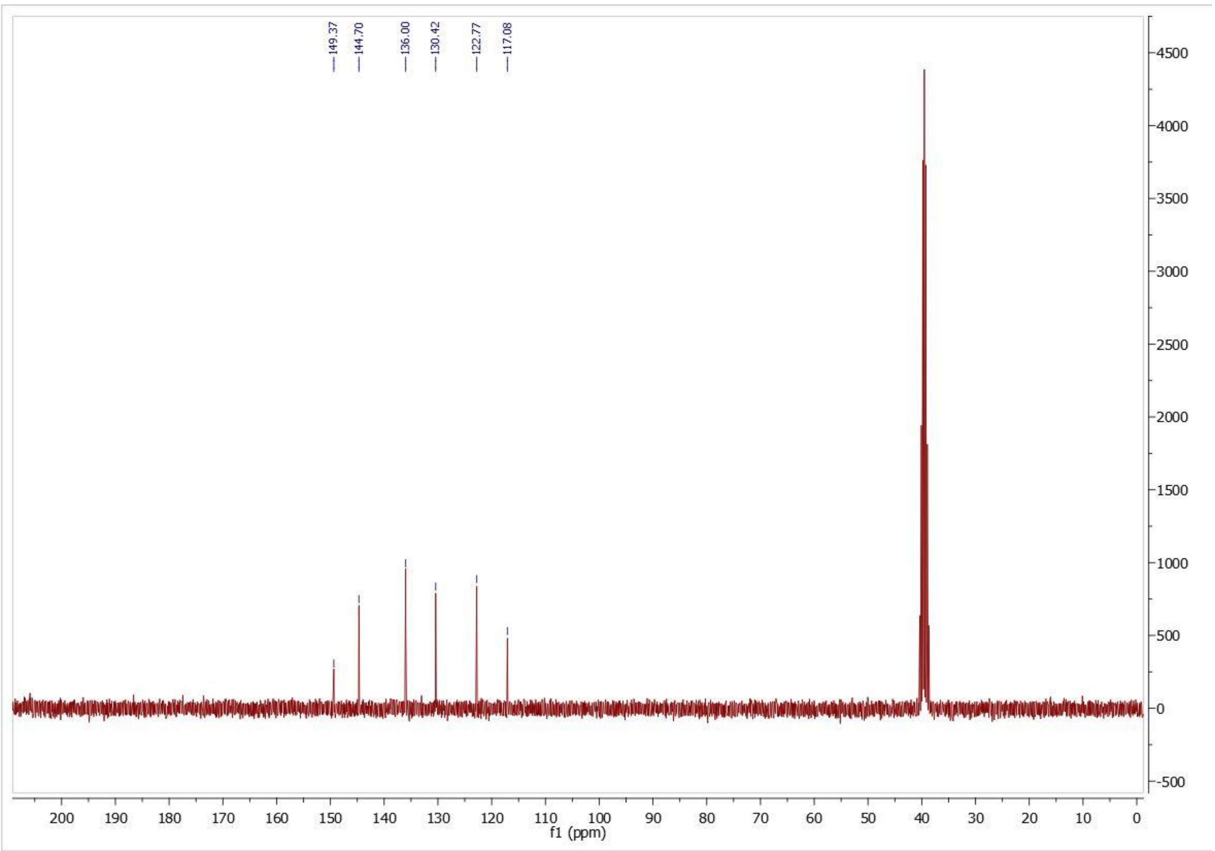

3-Amino-4-bromobenzene-1-sulfonamide (9)

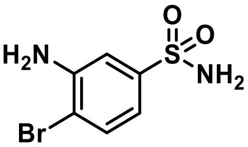

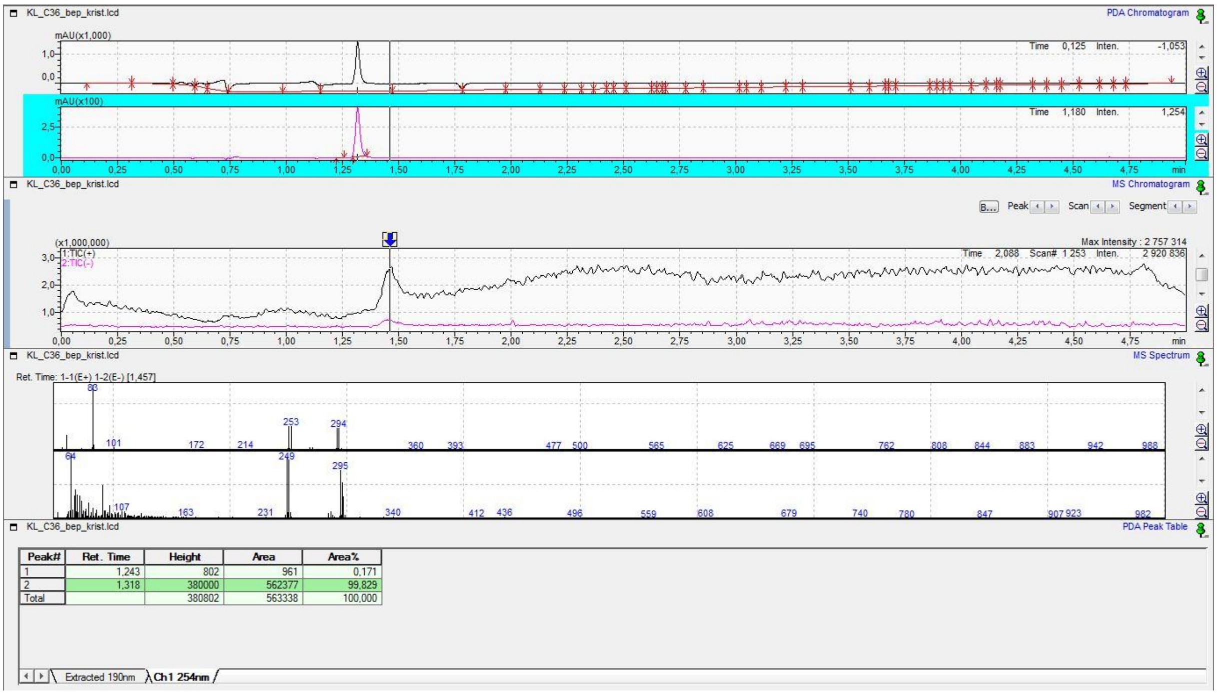

Peak#	Ret. Time	Height	Area	Area%
1	1,243	802	961	0,171
2	1,318	380000	562377	99,829
Total		380802	563338	100,000

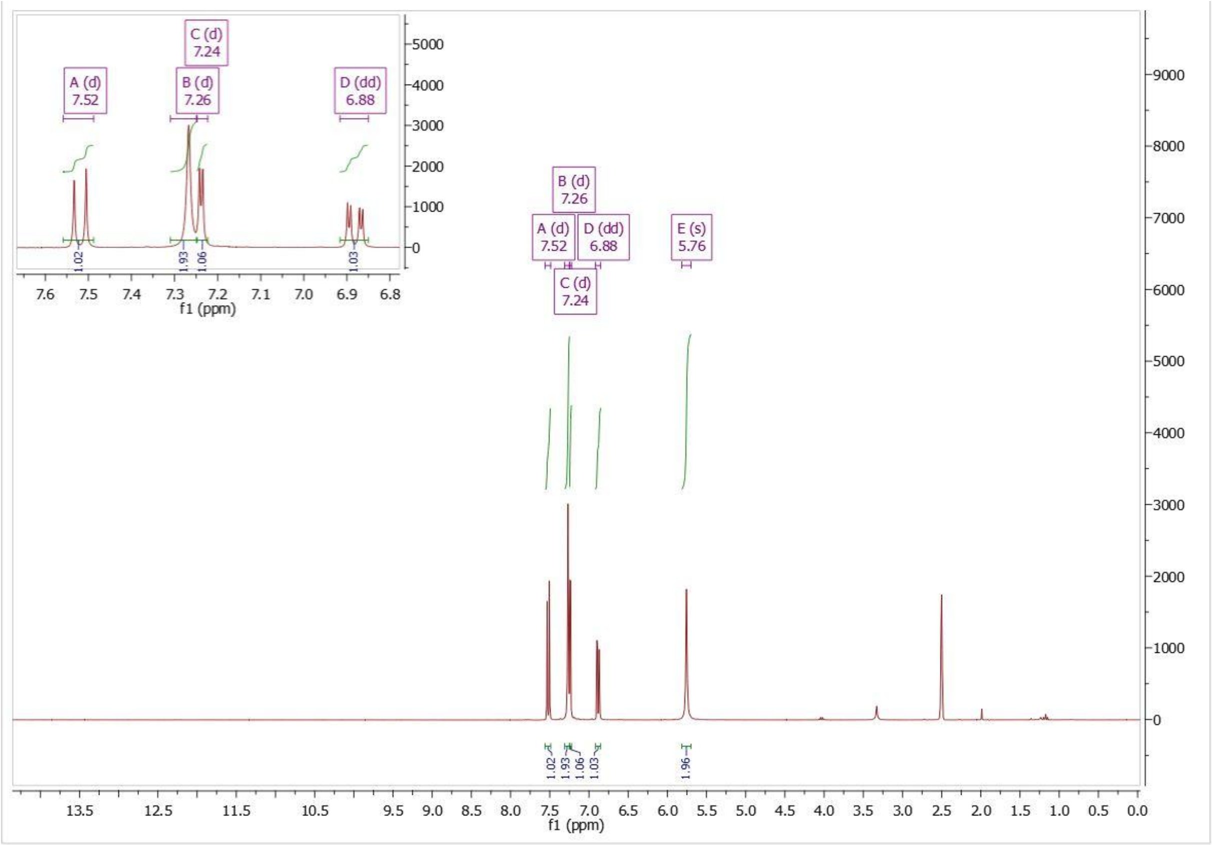

A (d)
7.52
B (d)
7.26
C (d)
7.24
D (dd)
6.88
E (s)
5.76
1.02
1.93
1.06
1.03
1.96
f1 (ppm)

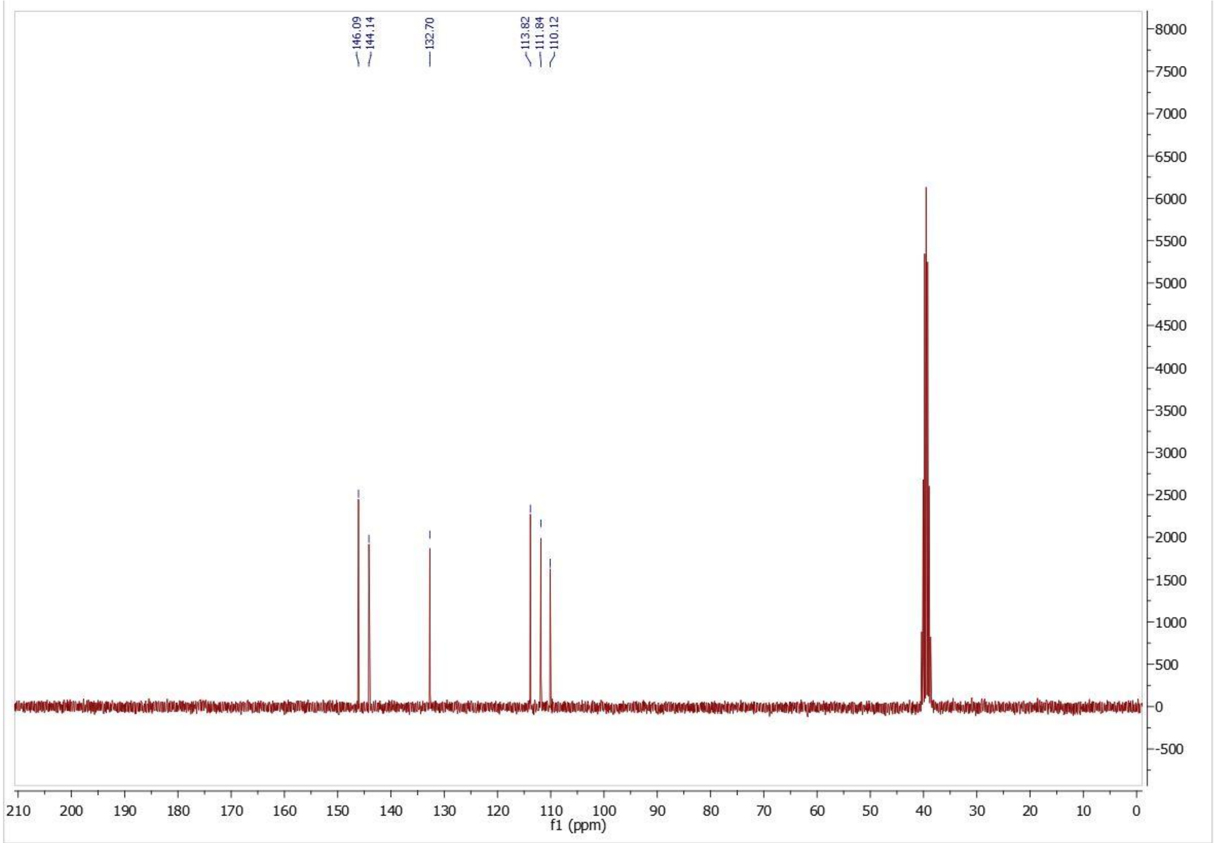

146.09
144.14
132.70
113.82
111.84
110.12
f1 (ppm)

4-Bromo-3-(methylamino)benzene-1-sulfonamide (11)

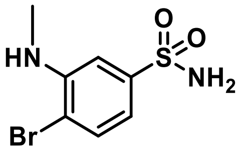

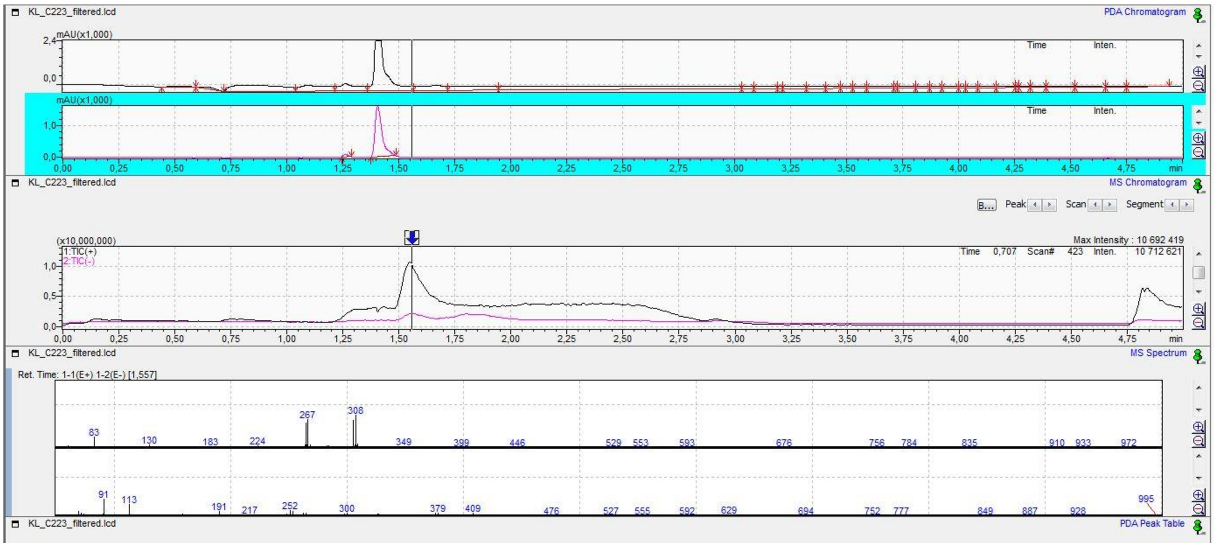

Peak#	Ret. Time	Height	Area	Area%
1	1,263	64656	87984	2,445
2	1,407	1544987	3511102	97,555
Total		1609643	3599086	100,000

Extracted 190nm | Ch1 254nm

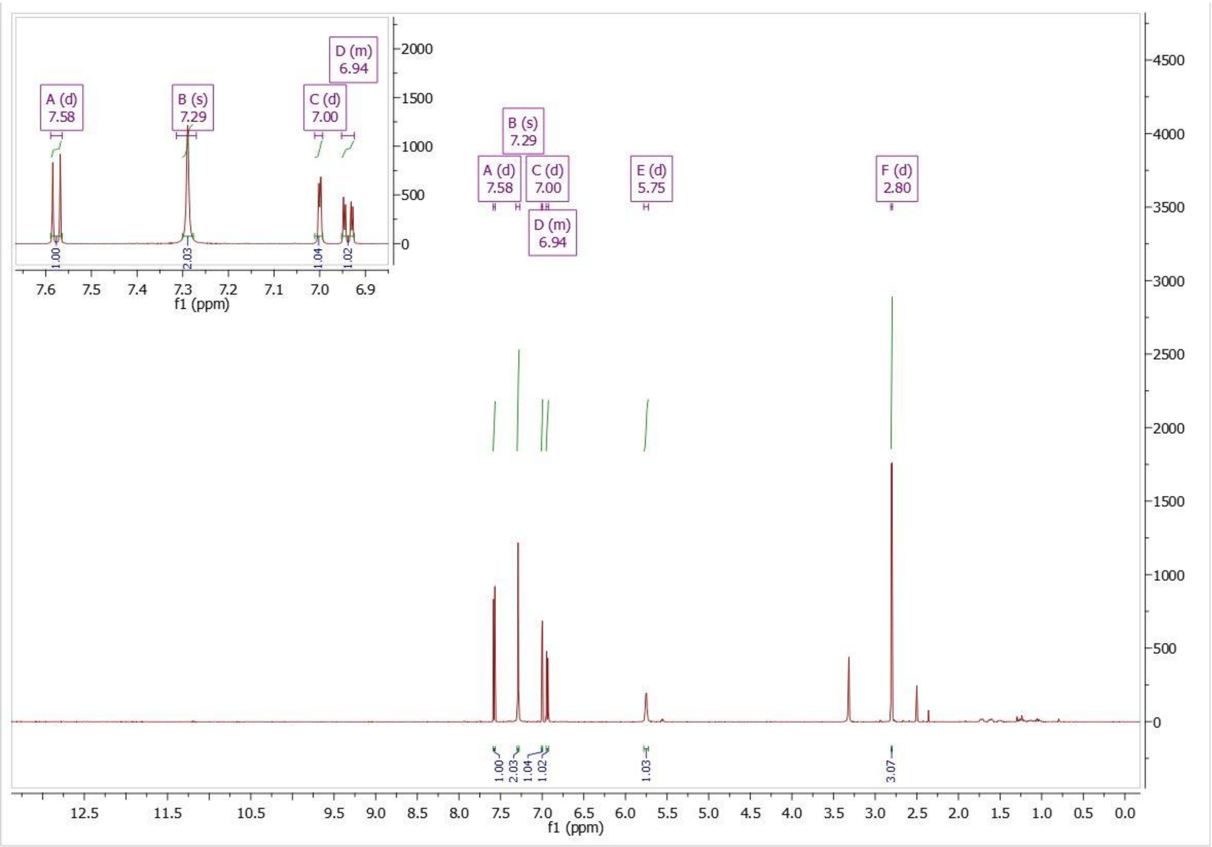

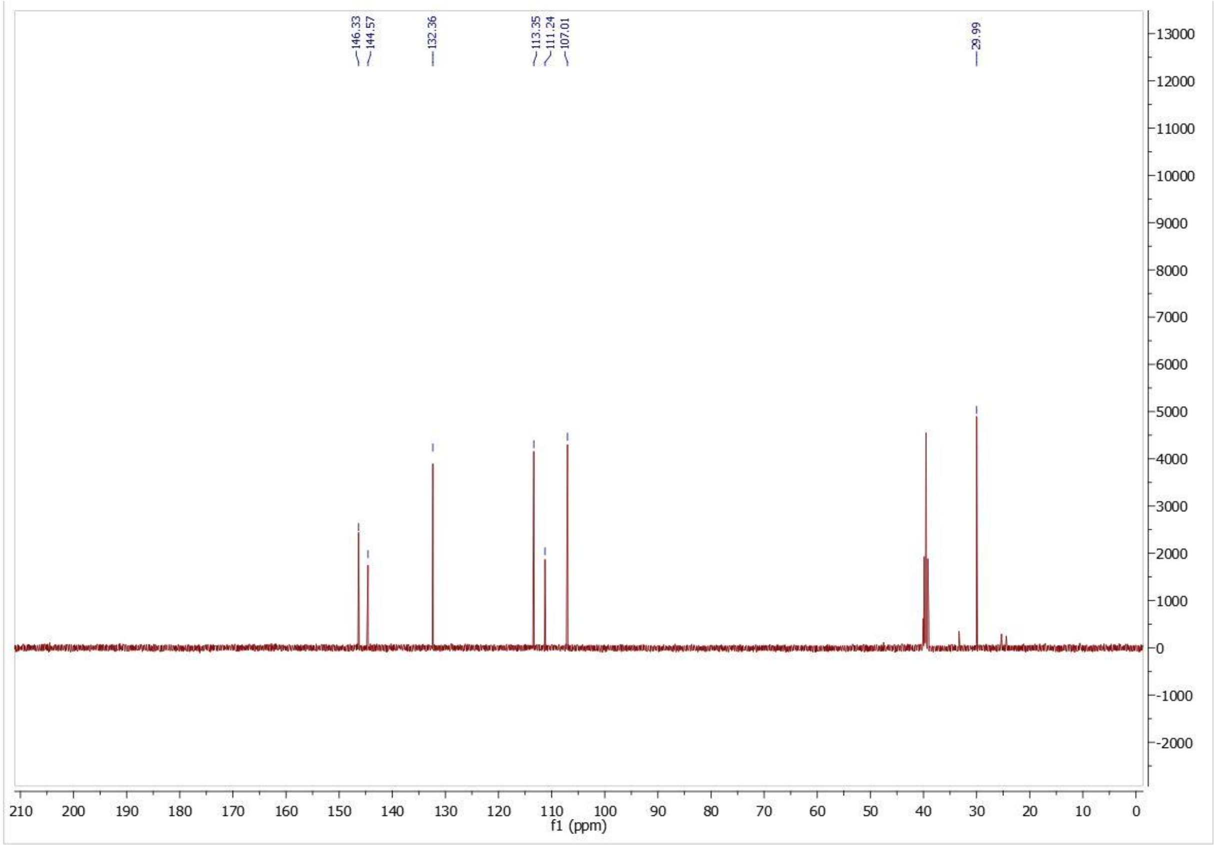
146.33
144.57
132.36
113.35
111.24
107.01
29.99
f1 (ppm)

Ethyl 2-(2-chloropyrimidin-4-yl)acetate (14)

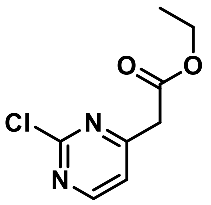

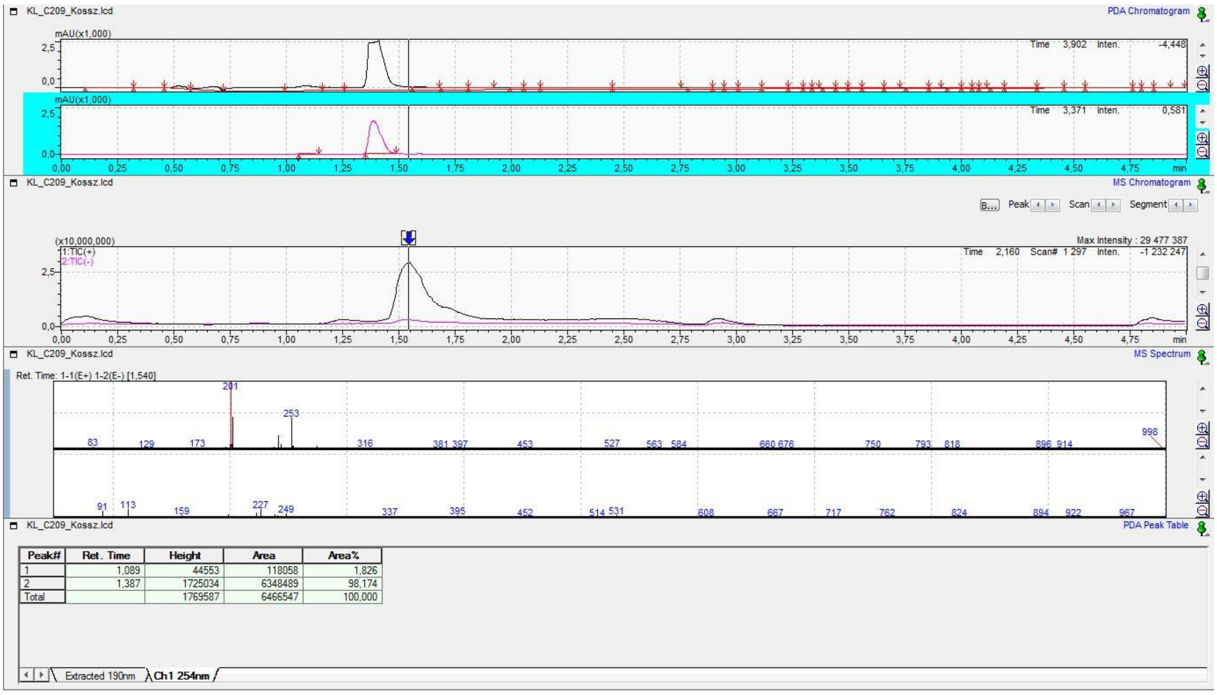

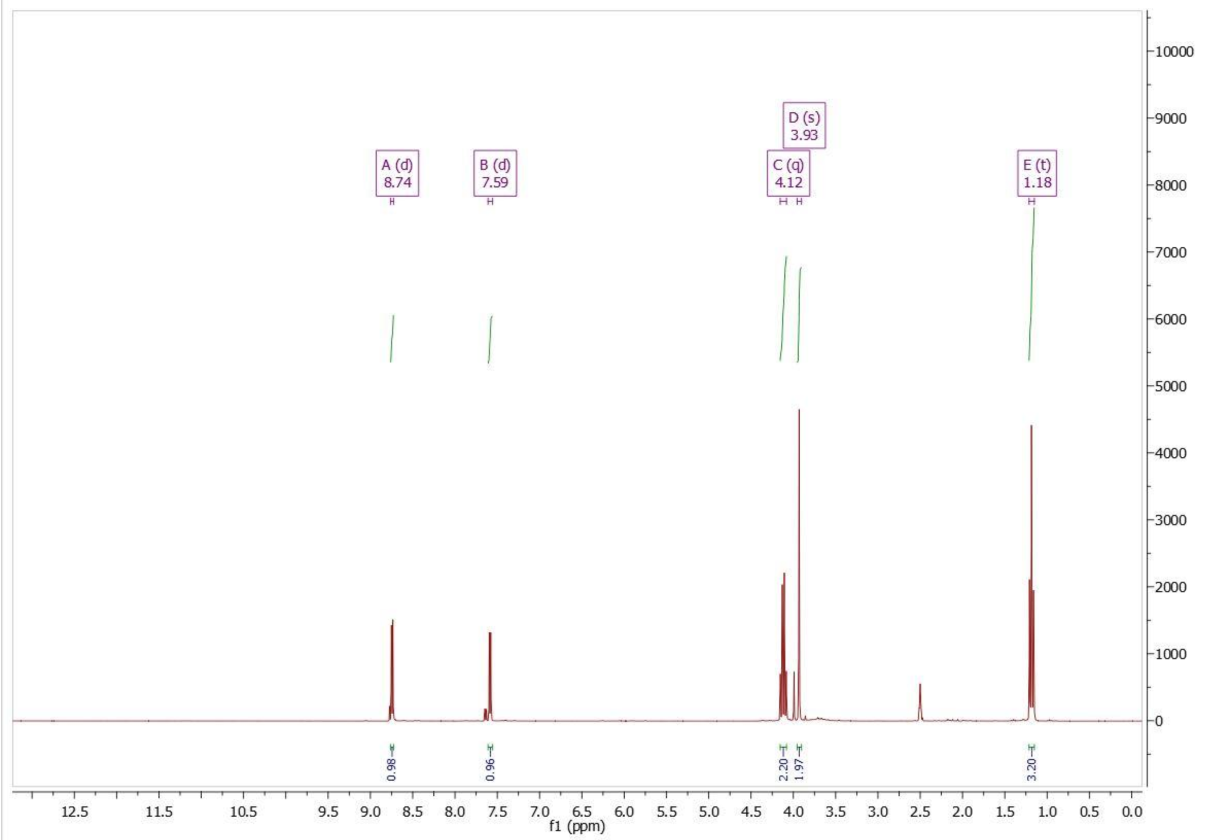

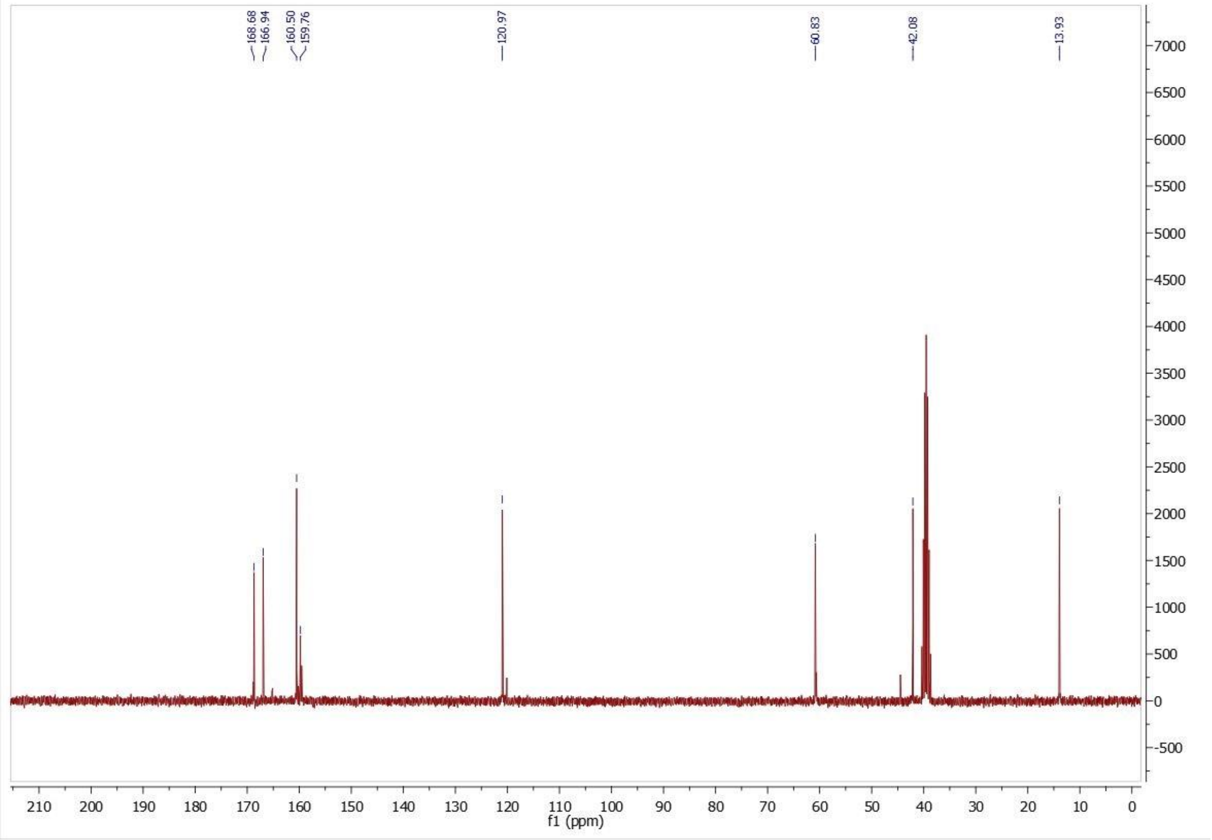

2-(2-Chloropyrimidin-4-yl)acetic acid (15)

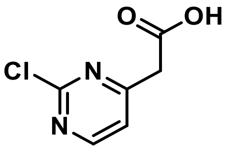

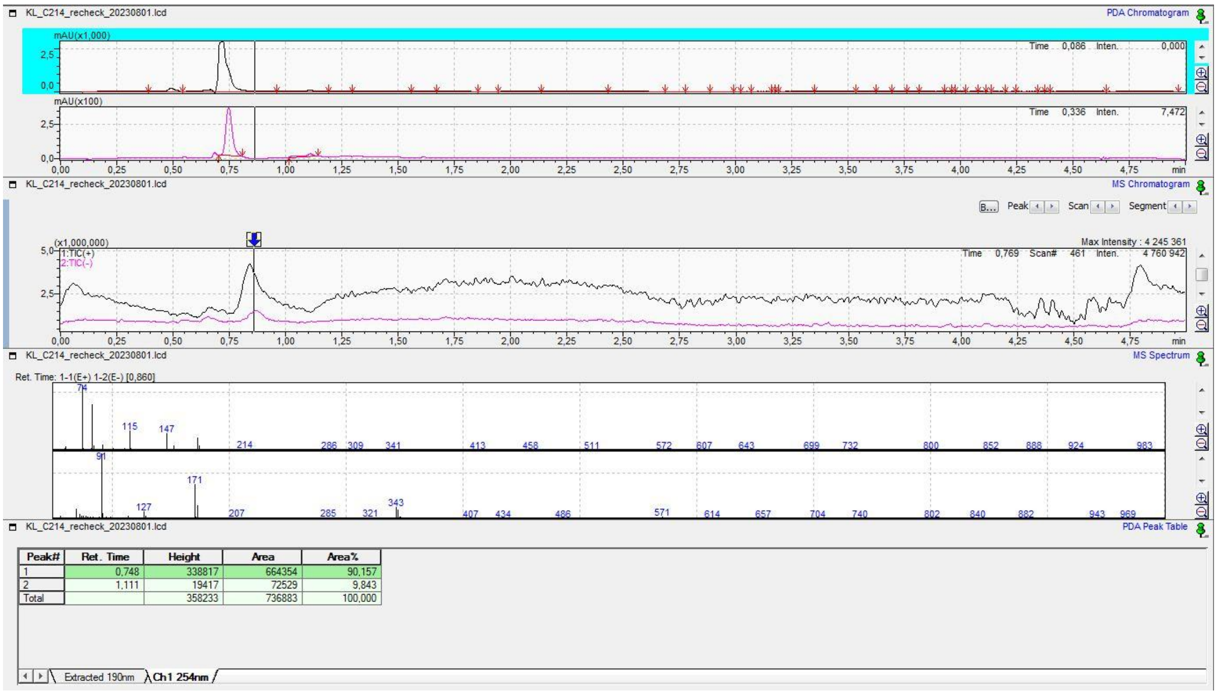

Peak#	Ret. Time	Height	Area	Area%
1	0,748	338817	664354	90,157
2	1,111	19417	72529	9,843
Total		358233	736883	100,000

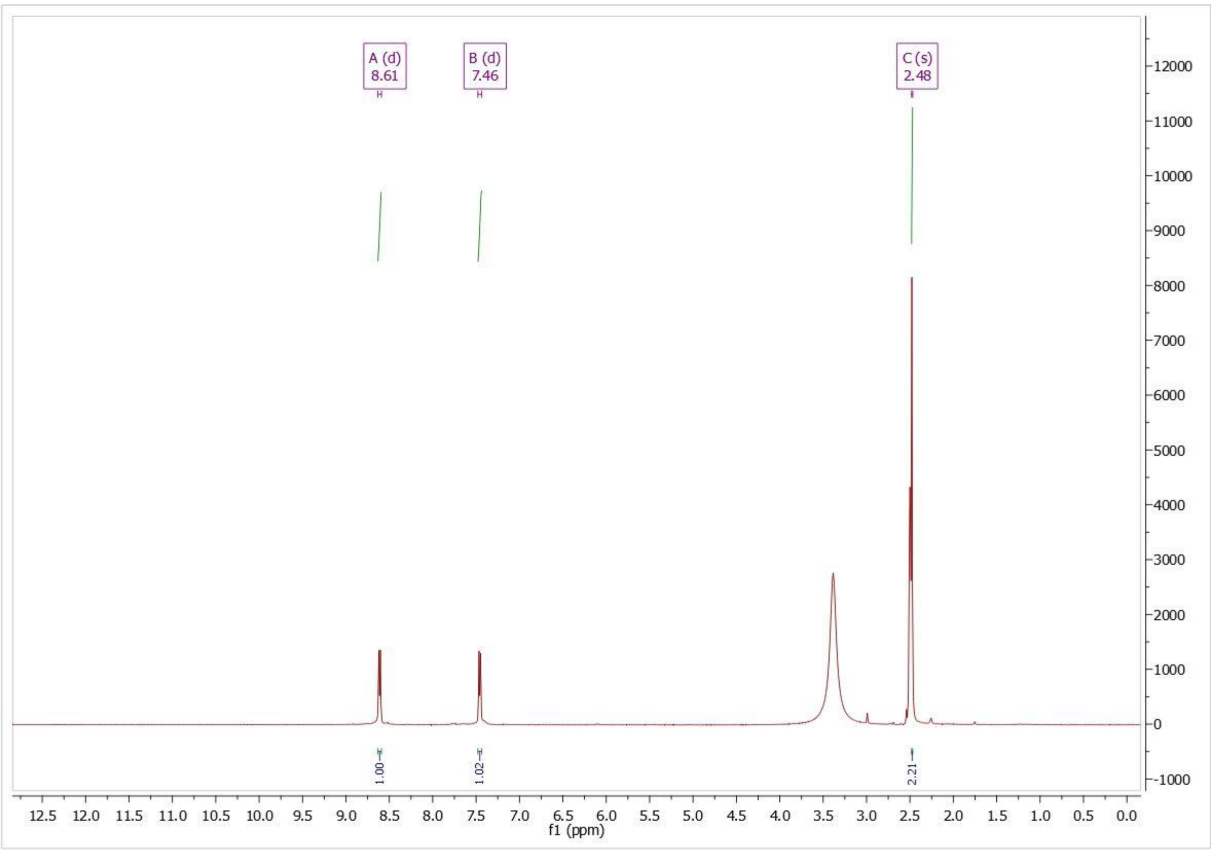
A (d)
8.61
B (d)
7.46
C (s)
2.48
1.00
1.02
2.21
f1 (ppm)

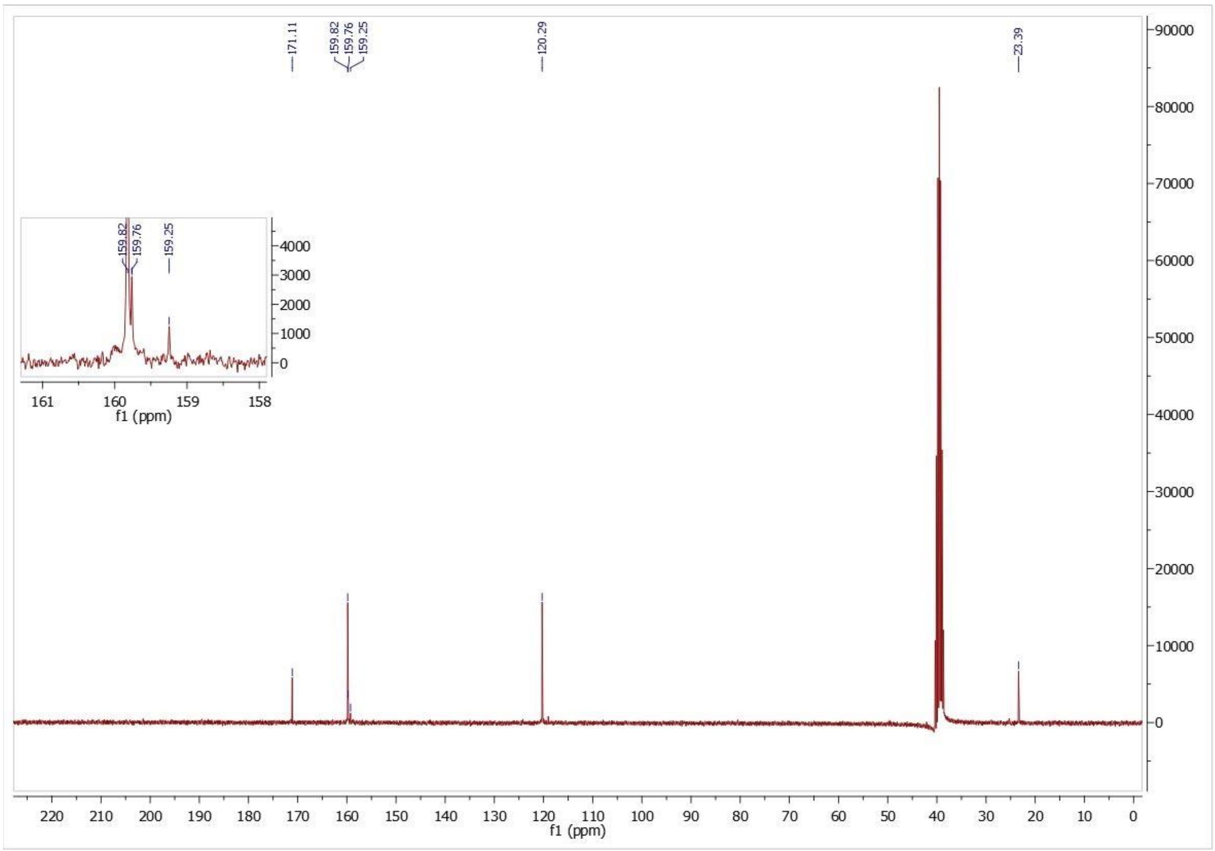
171.11
159.82
159.76
159.25
120.29
23.39
f1 (ppm)

N-(2-bromo-5-sulfamoylphenyl)-2-(2-chloropyrimidin-4-yl)acetamide (16)

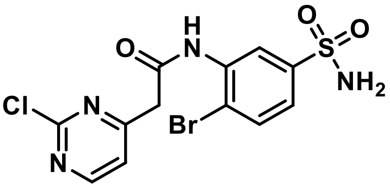

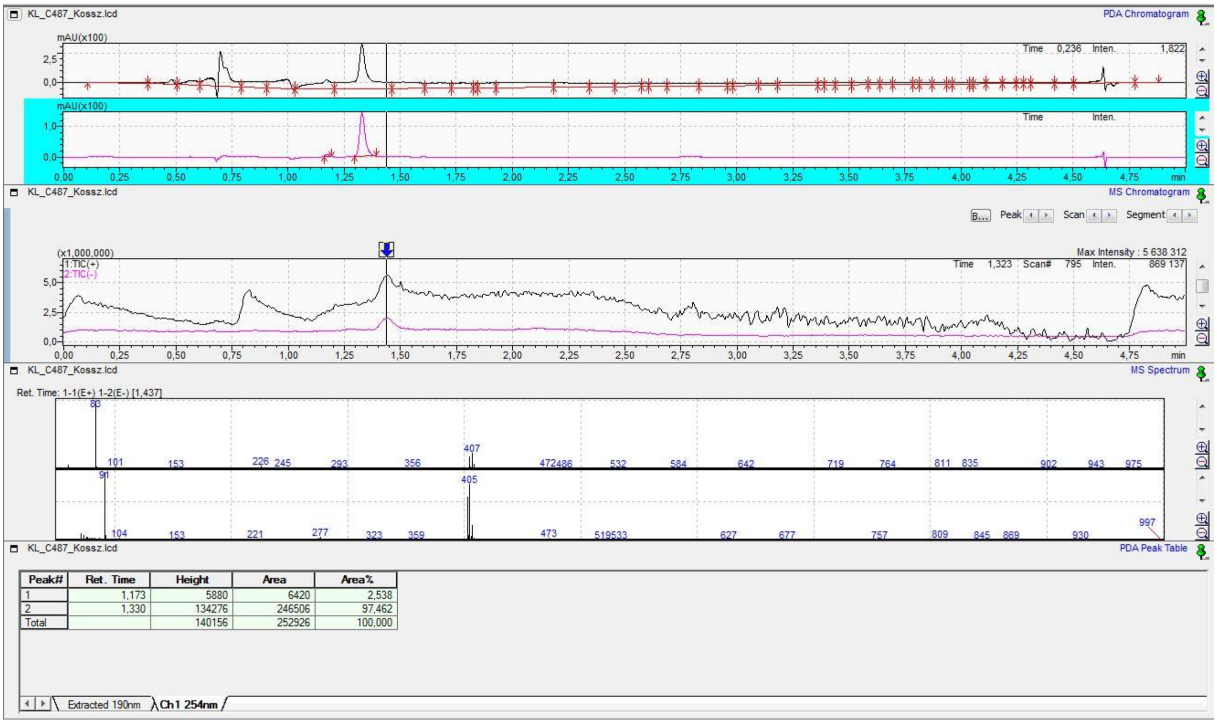

Peak#	Ret. Time	Height	Area	Area%
1	1,173	5880	6420	2,538
2	1,330	134276	246506	97,462
Total		140156	252926	100,000

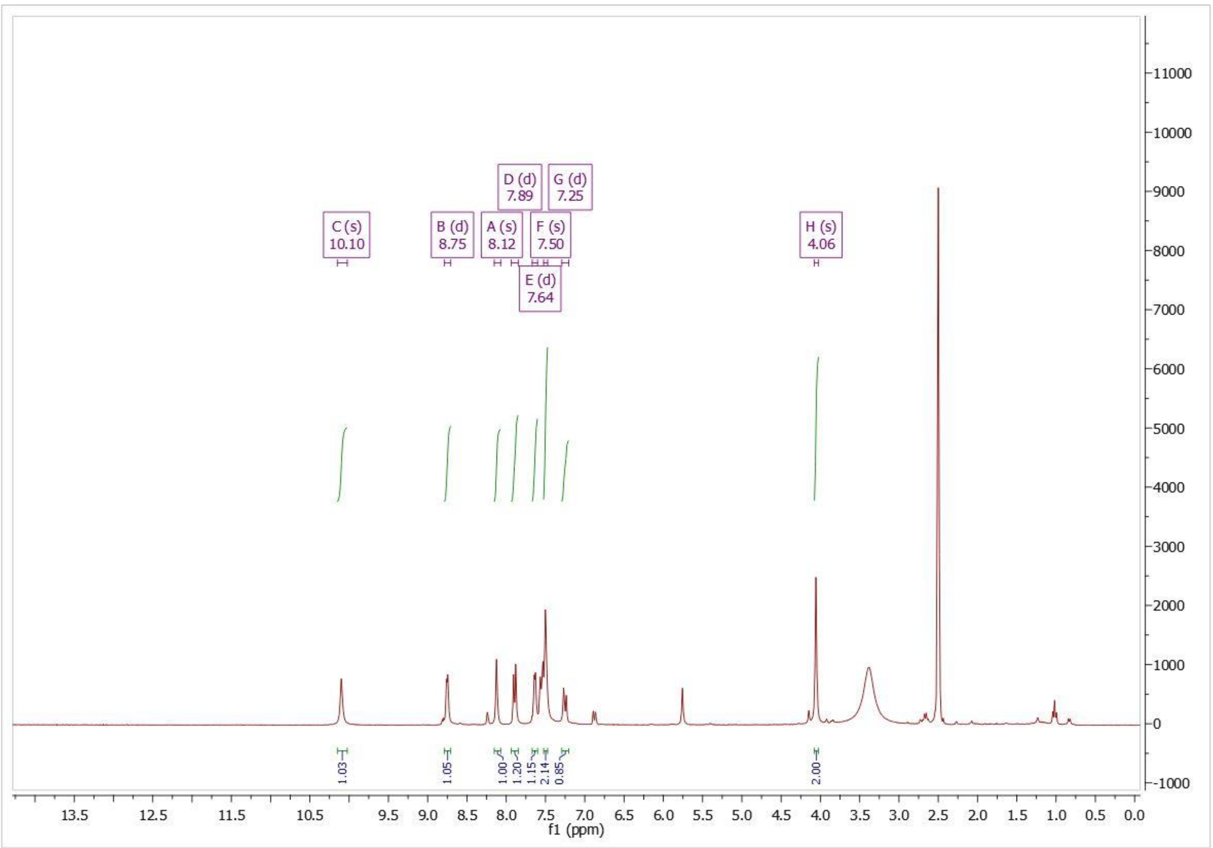

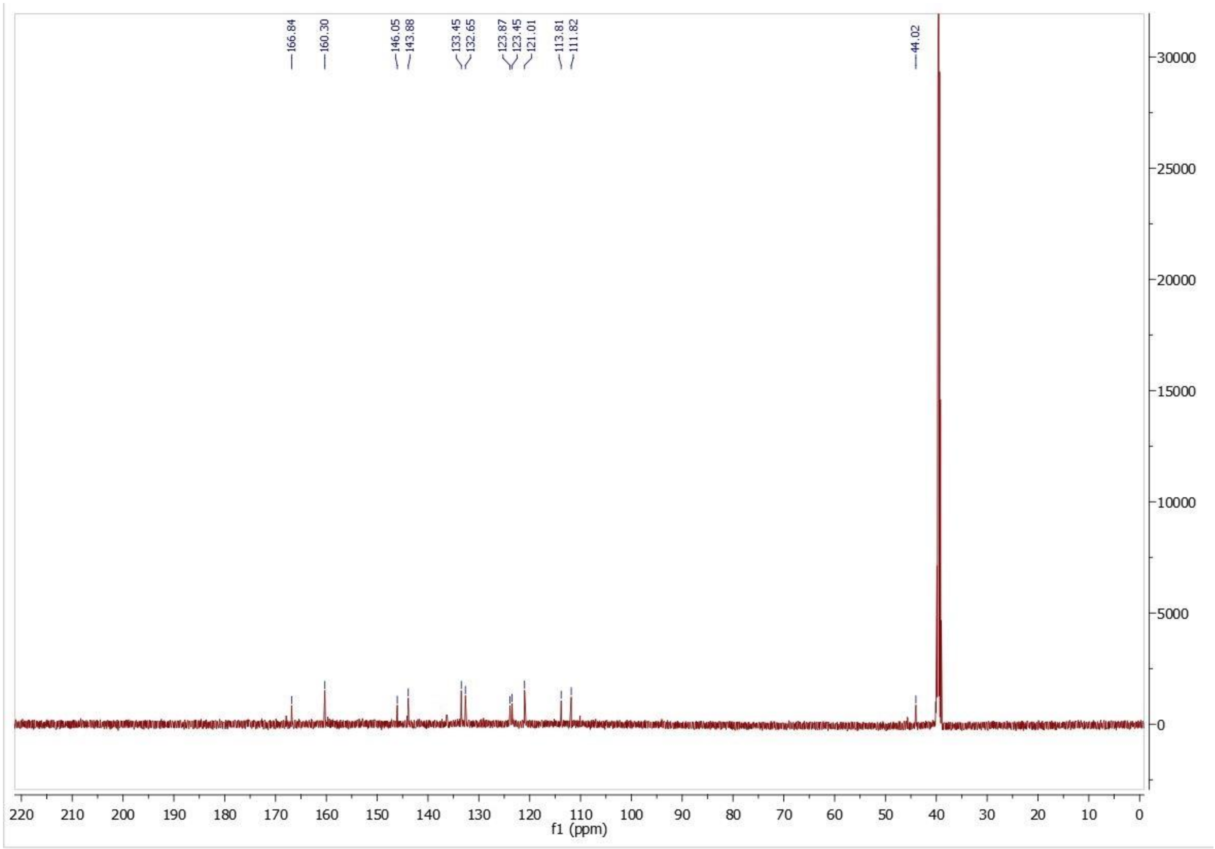

166.84
160.30
146.05
143.88
133.45
132.65
123.87
123.45
121.01
113.81
111.82
44.02
30000
25000
20000
15000
10000
5000
0
220 210 200 190 180 170 160 150 140 130 120 110 100 90 80 70 60 50 40 30 20 10 0
f1 (ppm)

N-(2-bromo-5-sulfamoylphenyl)-2-(2-chloropyrimidin-4-yl)-*N*-methylacetamide (17)

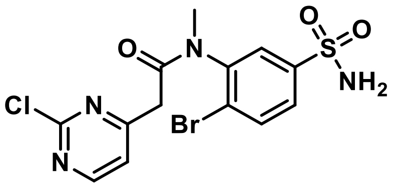

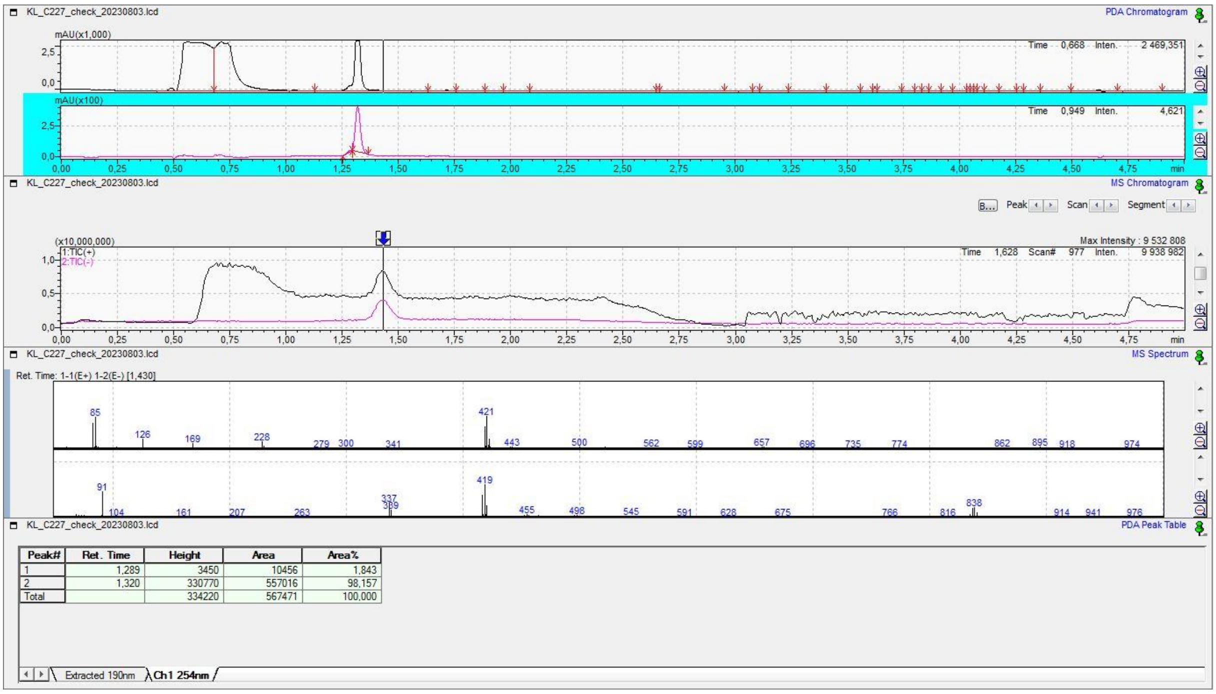

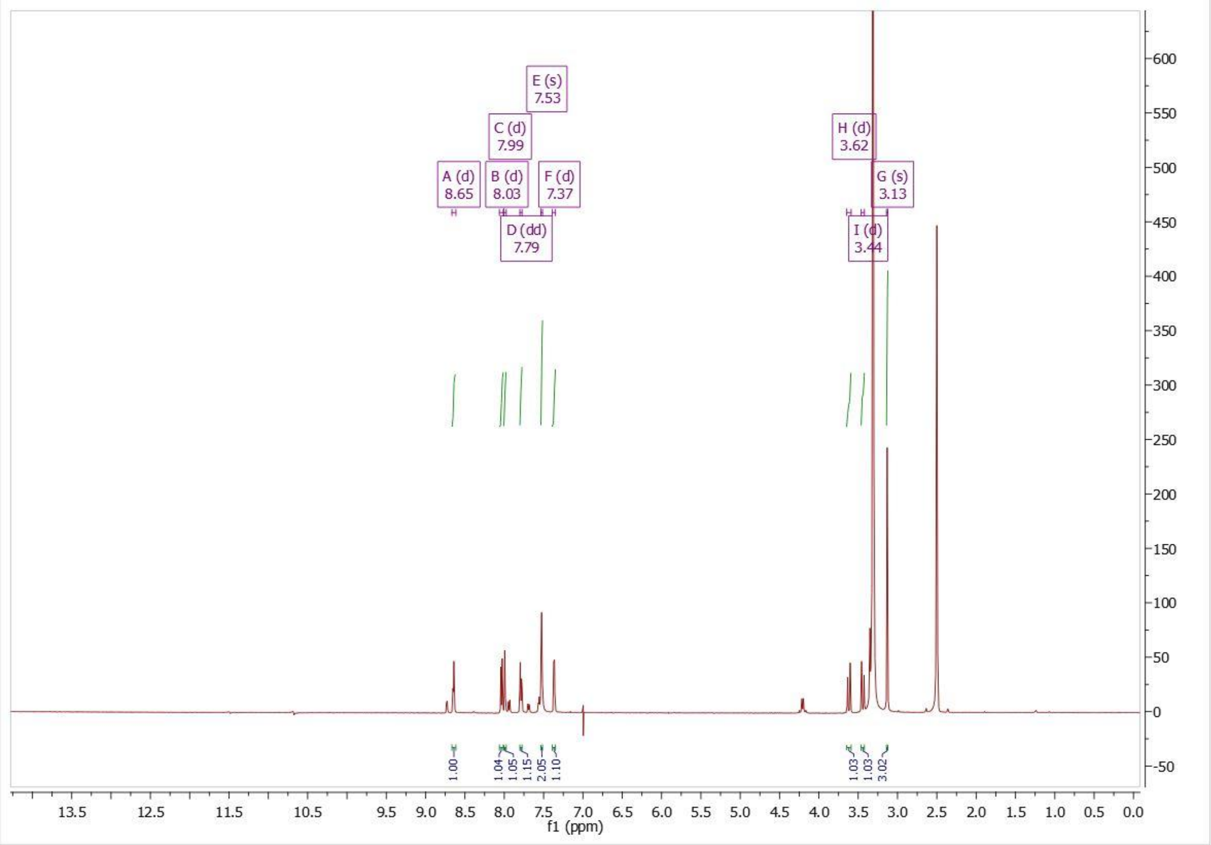
E (s)
7.53
C (d)
7.99
A (d)
8.65
B (d)
8.03
F (d)
7.37
D (dd)
7.79
H (d)
3.62
G (s)
3.13
I (d)
3.44
1.00
1.04
1.05
1.15
2.05
1.10
1.03
1.03
3.02
f1 (ppm)

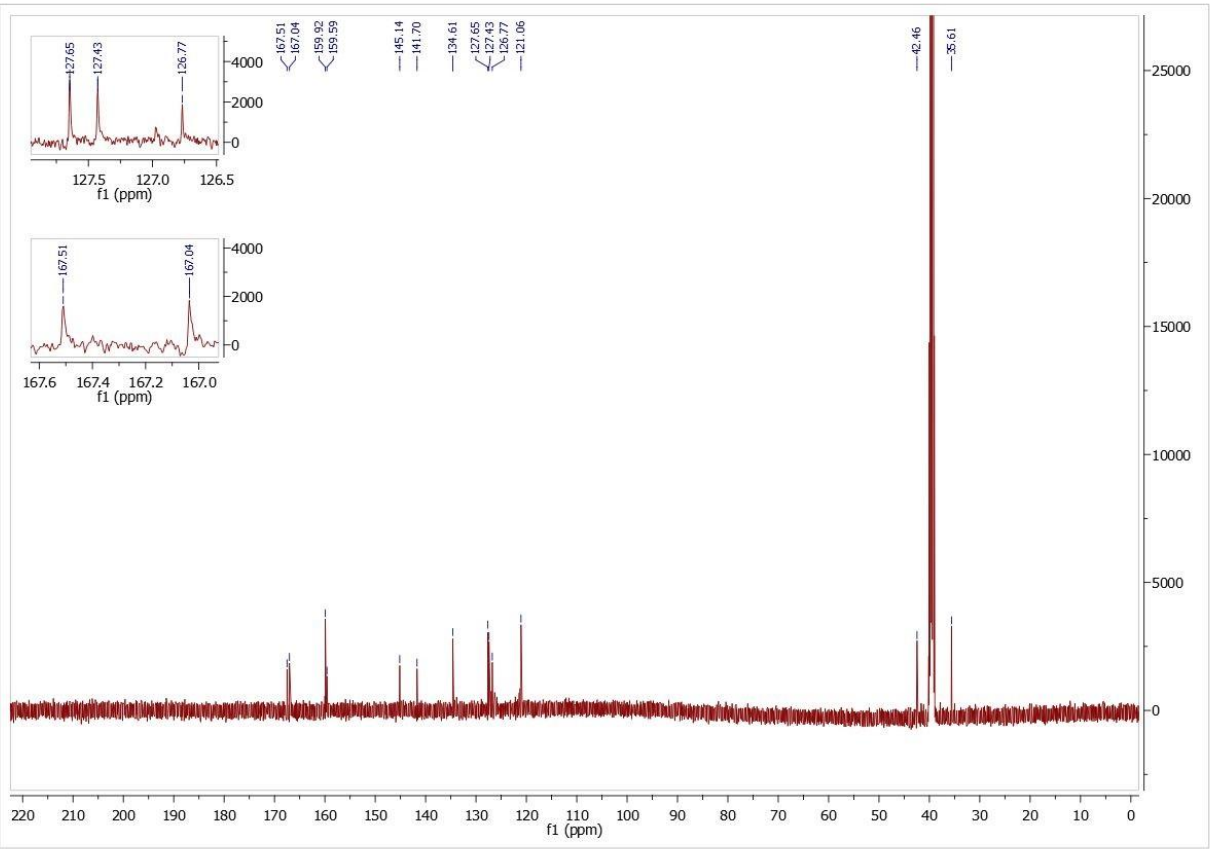
167.51
167.04
159.92
159.59
145.14
141.70
134.61
127.65
127.43
126.77
121.06
42.46
35.61
f1 (ppm)

3-(Aminomethyl)-4-bromobenzene-1-sulfonic acid (19)

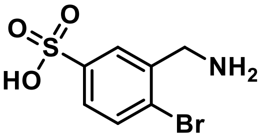

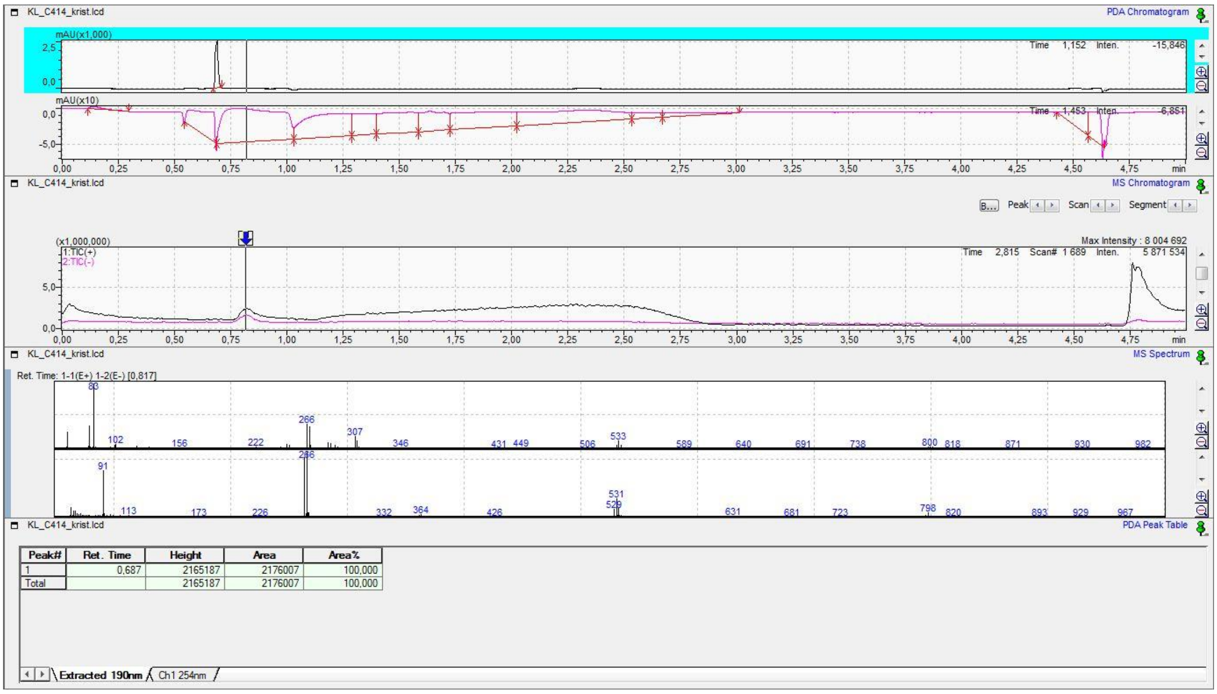

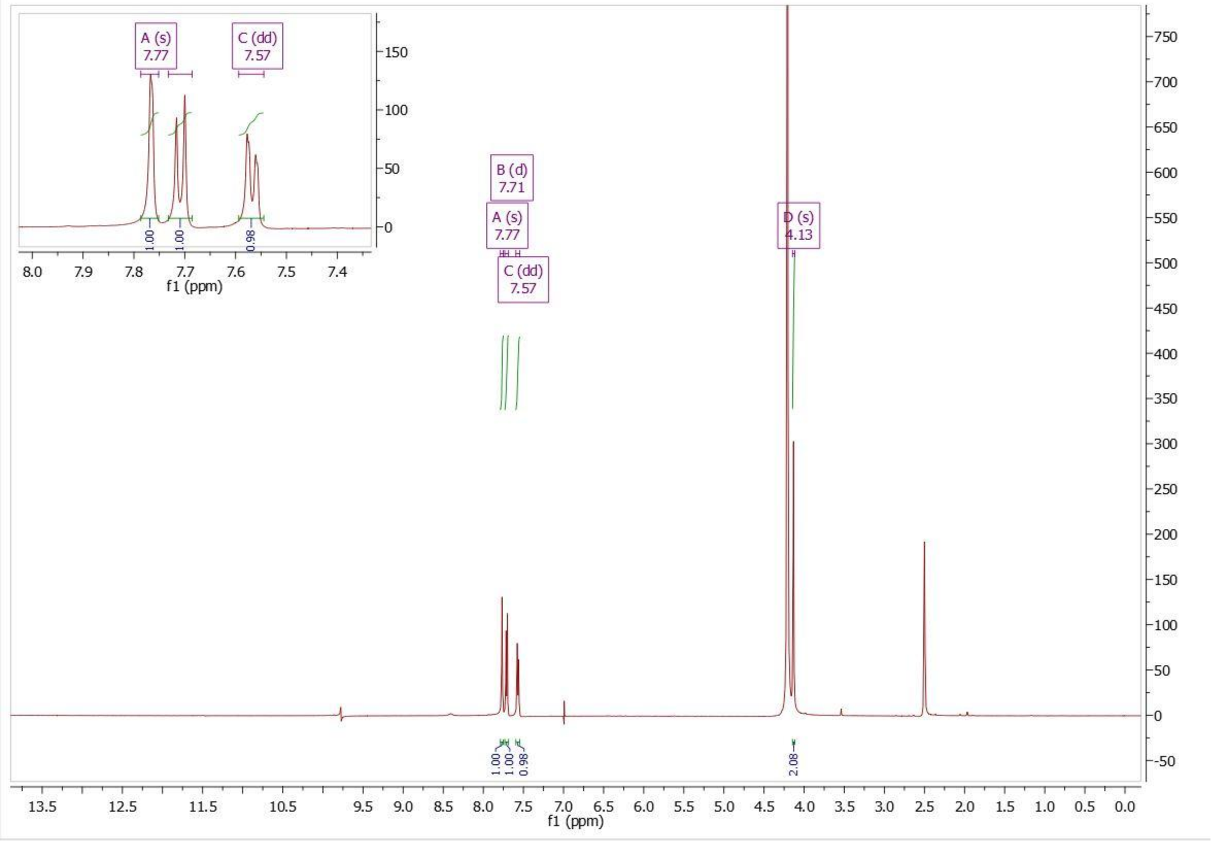

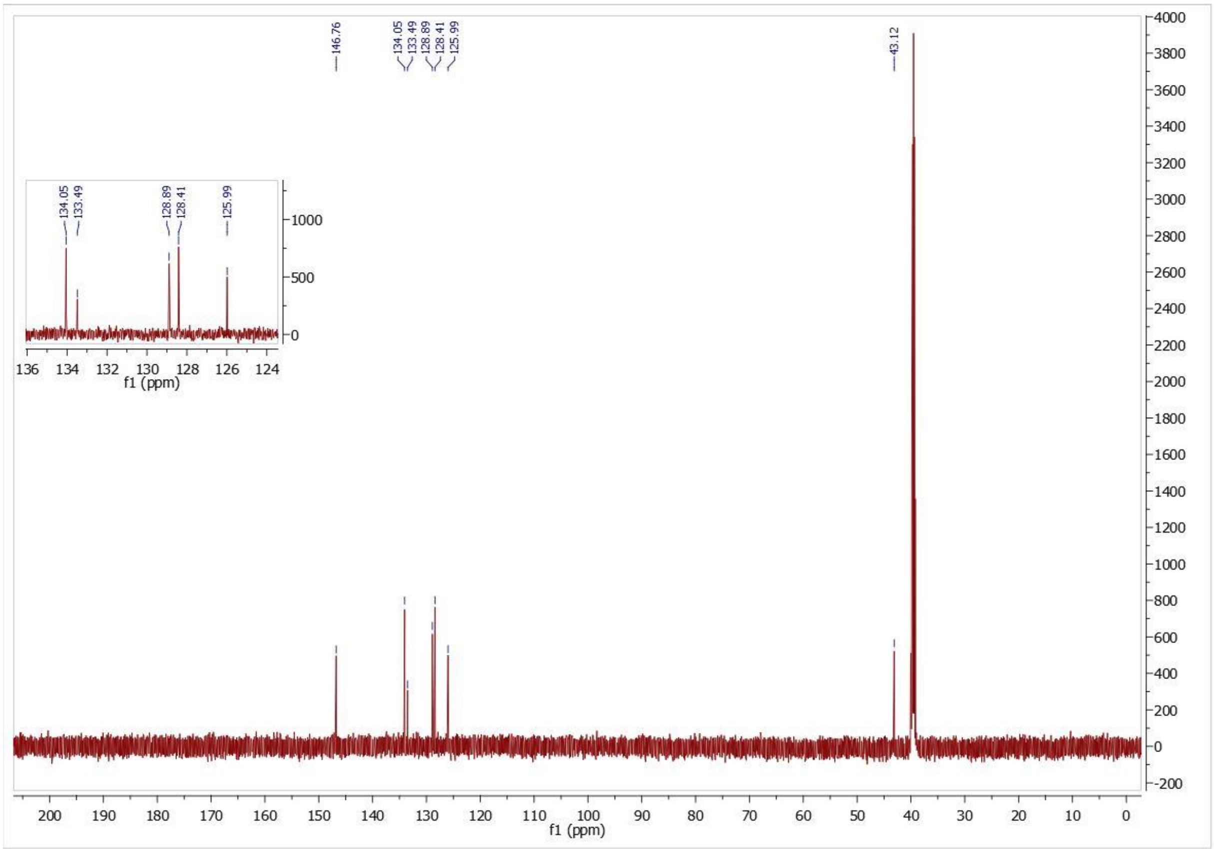

146.76
134.05
133.49
128.89
128.41
125.99
43.12
f1 (ppm)

Ethylbis(propan-2-yl)azanium 4-bromo-3-[[(2-chloropyrimidin-4yl)formamido]methyl]benzene-1-sulfonate (21)

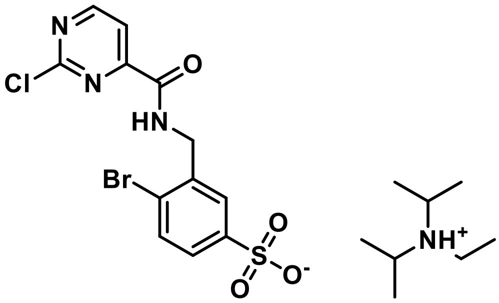

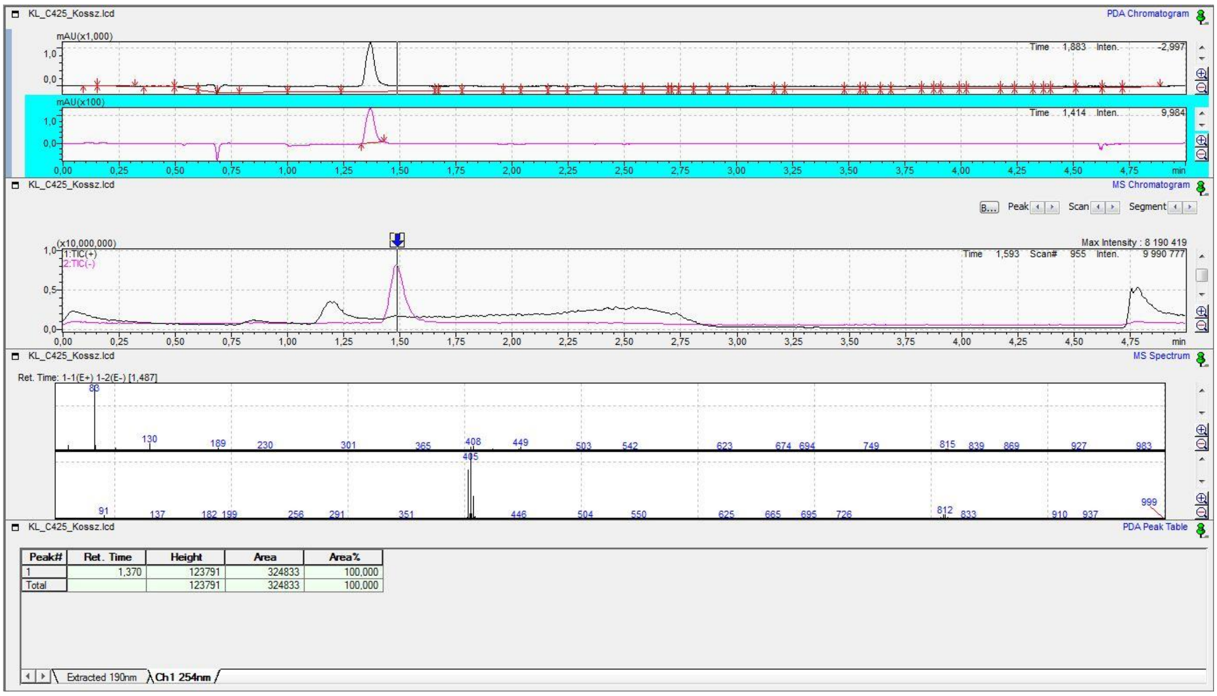

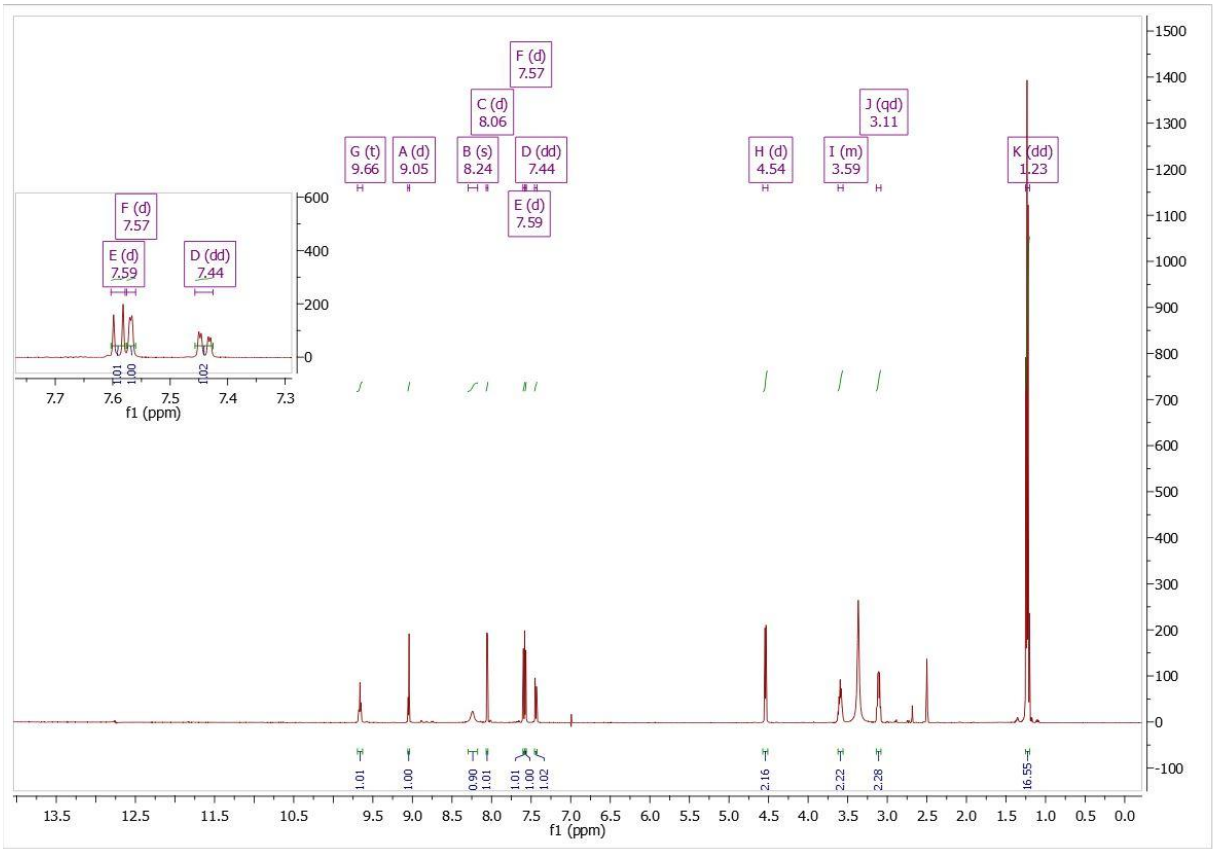

F (d)
7.57
C (d)
8.06
J (qd)
3.11
G (t)
9.66
A (d)
9.05
B (s)
8.24
D (dd)
7.44
H (d)
4.54
I (m)
3.59
K (dd)
1.23
E (d)
7.59
f1 (ppm)

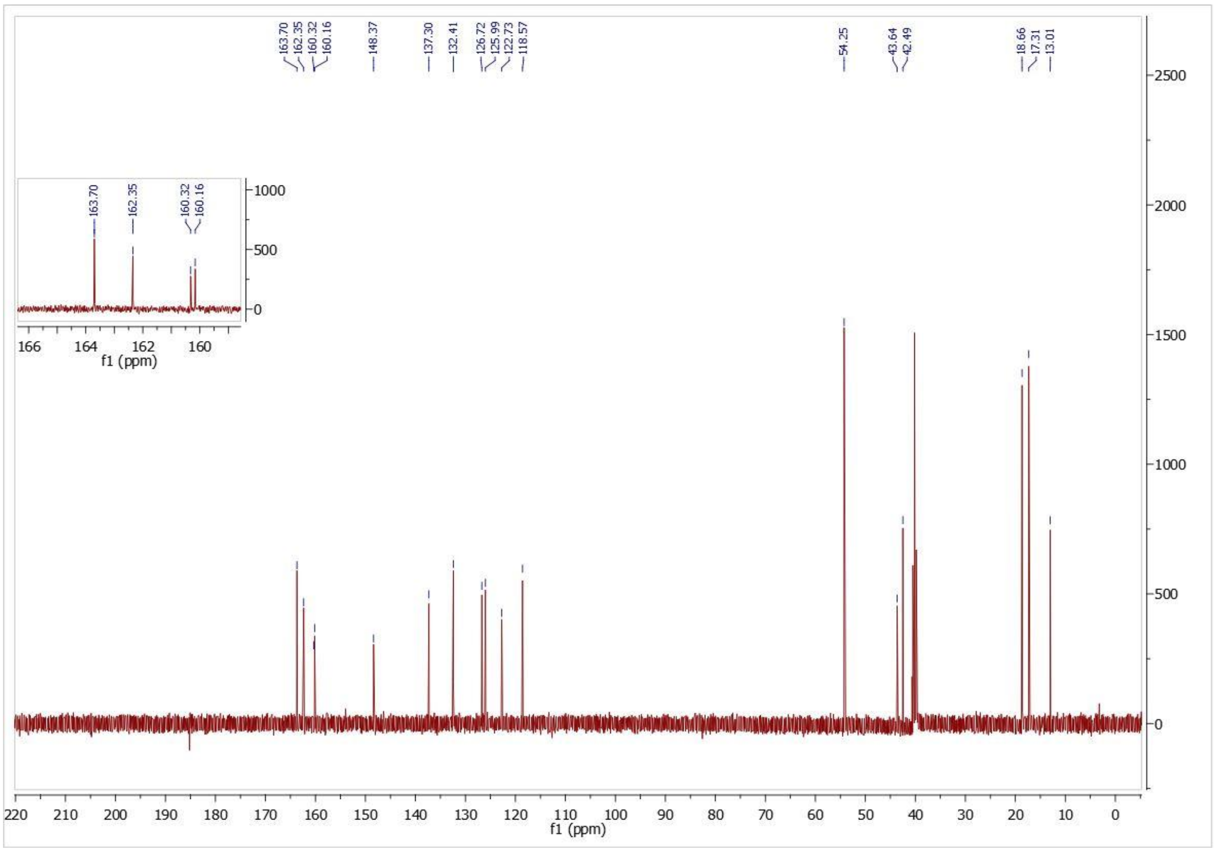

163.70
162.35
160.32
160.16
148.37
137.30
132.41
126.72
125.99
122.73
118.57
54.25
43.64
42.49
18.66
17.31
13.01
f1 (ppm)

N-[(2-bromo-5-sulfamoylphenyl)methyl]-2-chloropyrimidine-4-carboxamide (22)

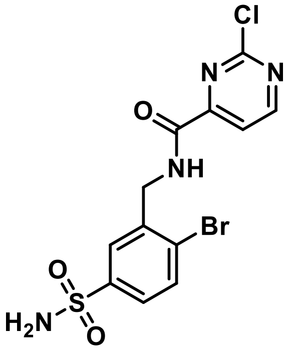

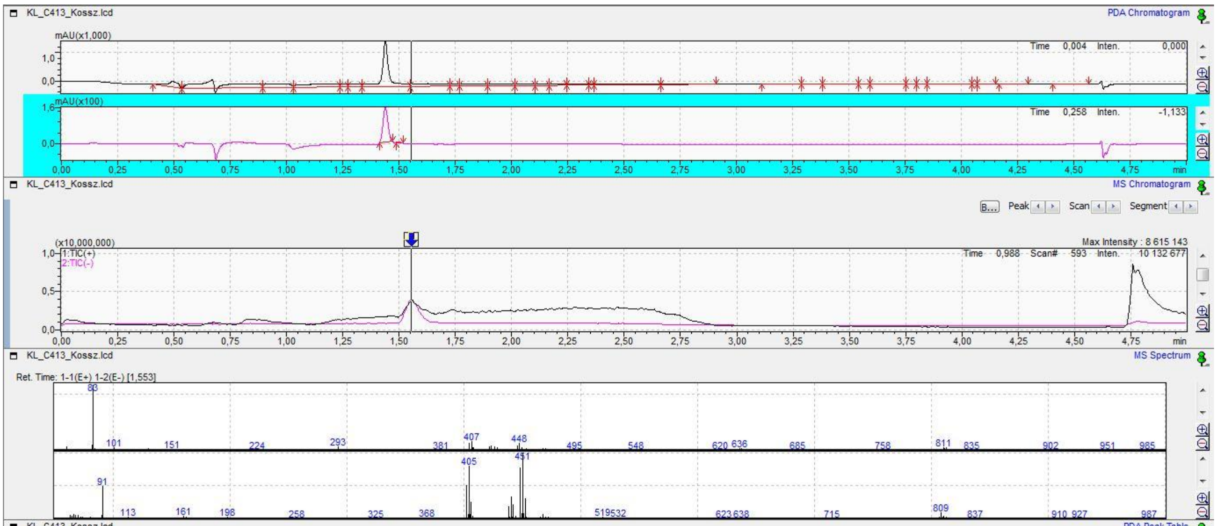

KL_C413_Kossz.lcd PDA Peak Table

Peak#	Ret. Time	Height	Area	Area%
1	1,440	147854	243370	97,919
2	1,500	4941	5173	2,081
Total		152795	248543	100,000

Extracted 190nm | Ch1 254nm

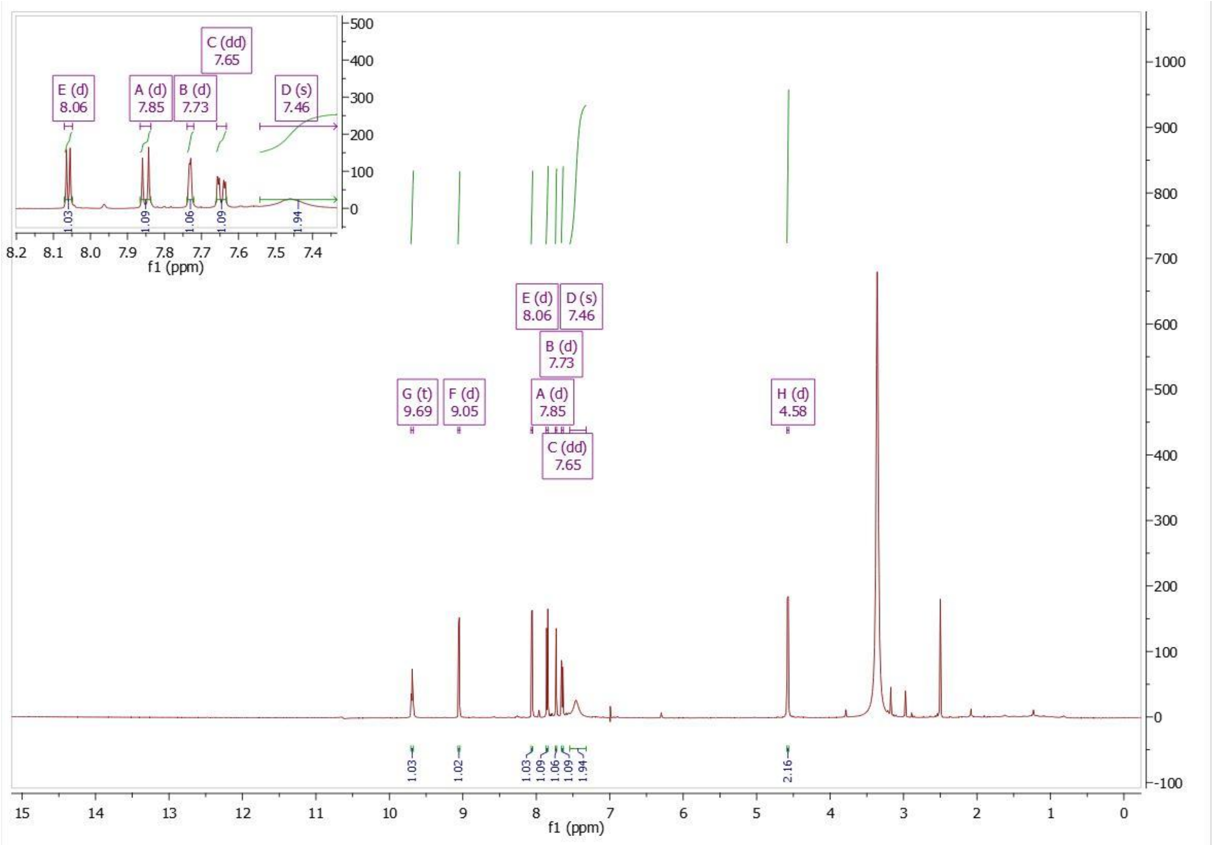

E (d) 8.06
A (d) 7.85
B (d) 7.73
C (dd) 7.65
D (s) 7.46
G (t) 9.69
F (d) 9.05
H (d) 4.58
f1 (ppm)

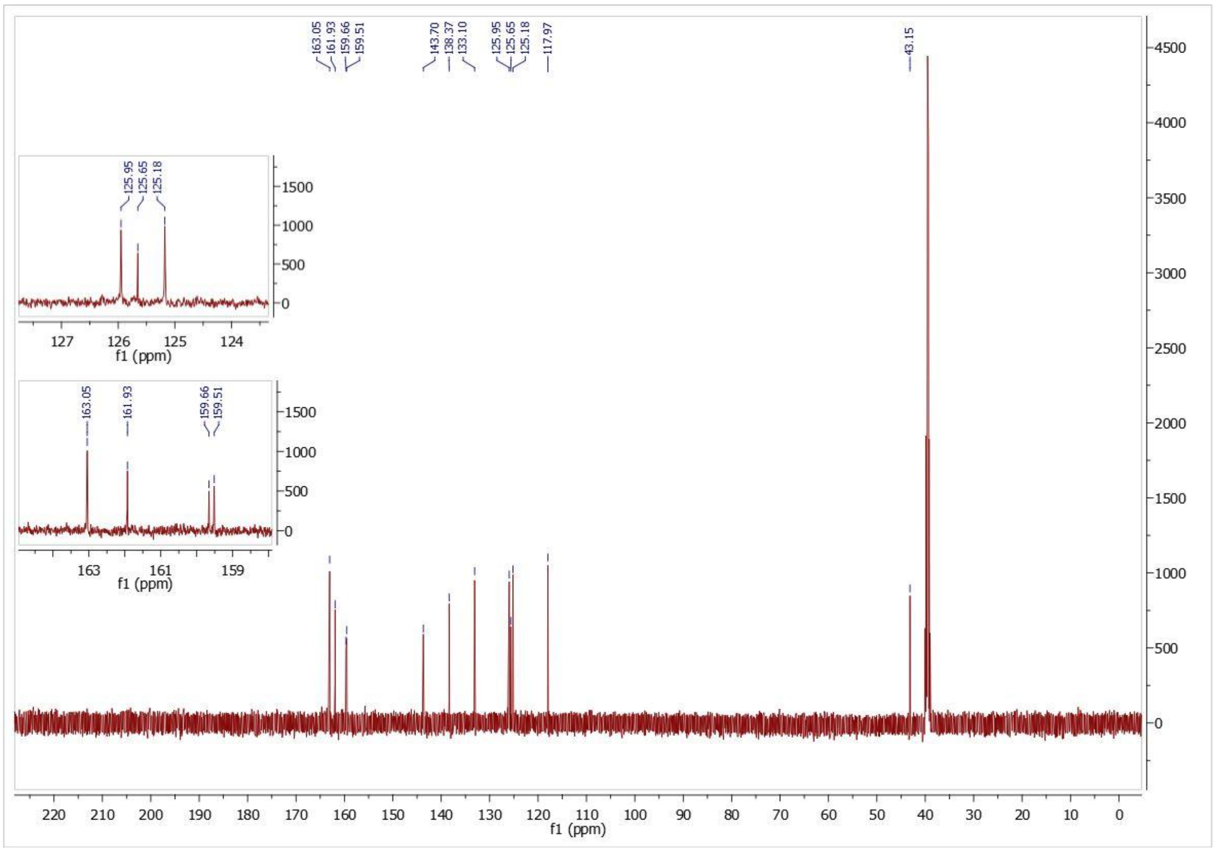

163.05
161.93
159.66
159.51
143.70
138.37
133.10
125.95
125.65
125.18
117.97
43.15
f1 (ppm)

2-(2-Bromo-5-sulfamoylphenyl)-*N*-(4-methylpyridin-3-yl)acetamide (24)

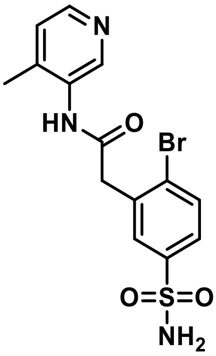

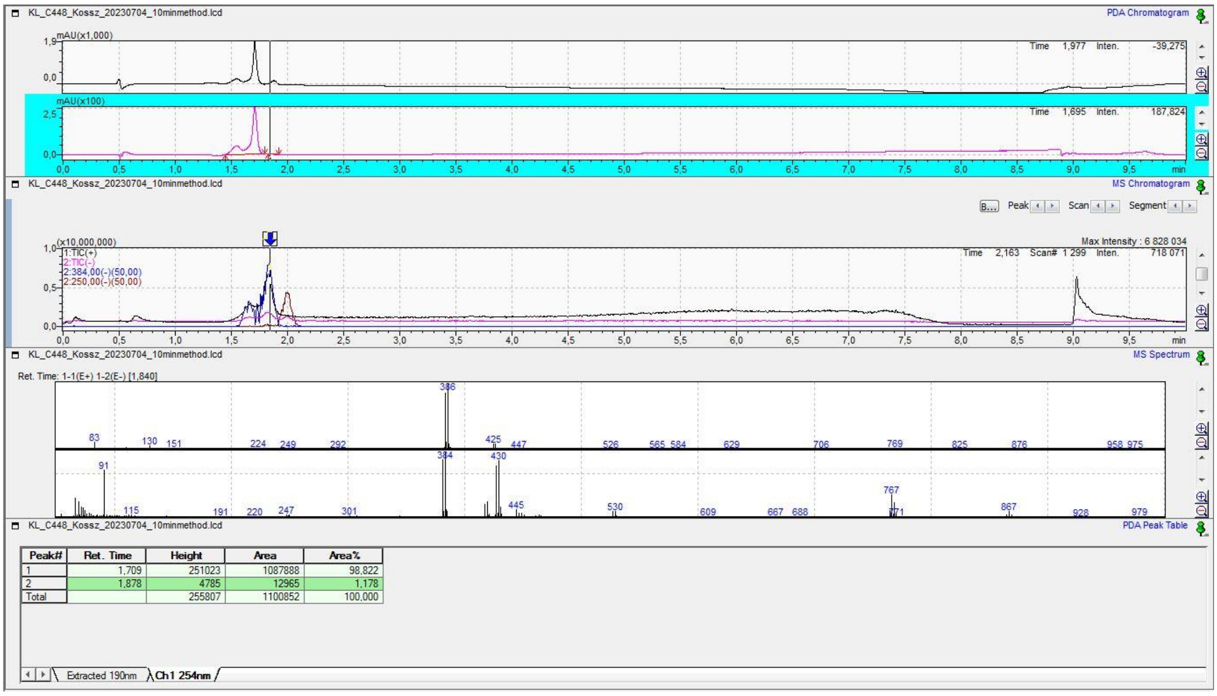

Peak#	Ret. Time	Height	Area	Area%
1	1,709	251023	1087888	98,822
2	1,878	4785	12965	1,178
Total		255807	1100852	100,000

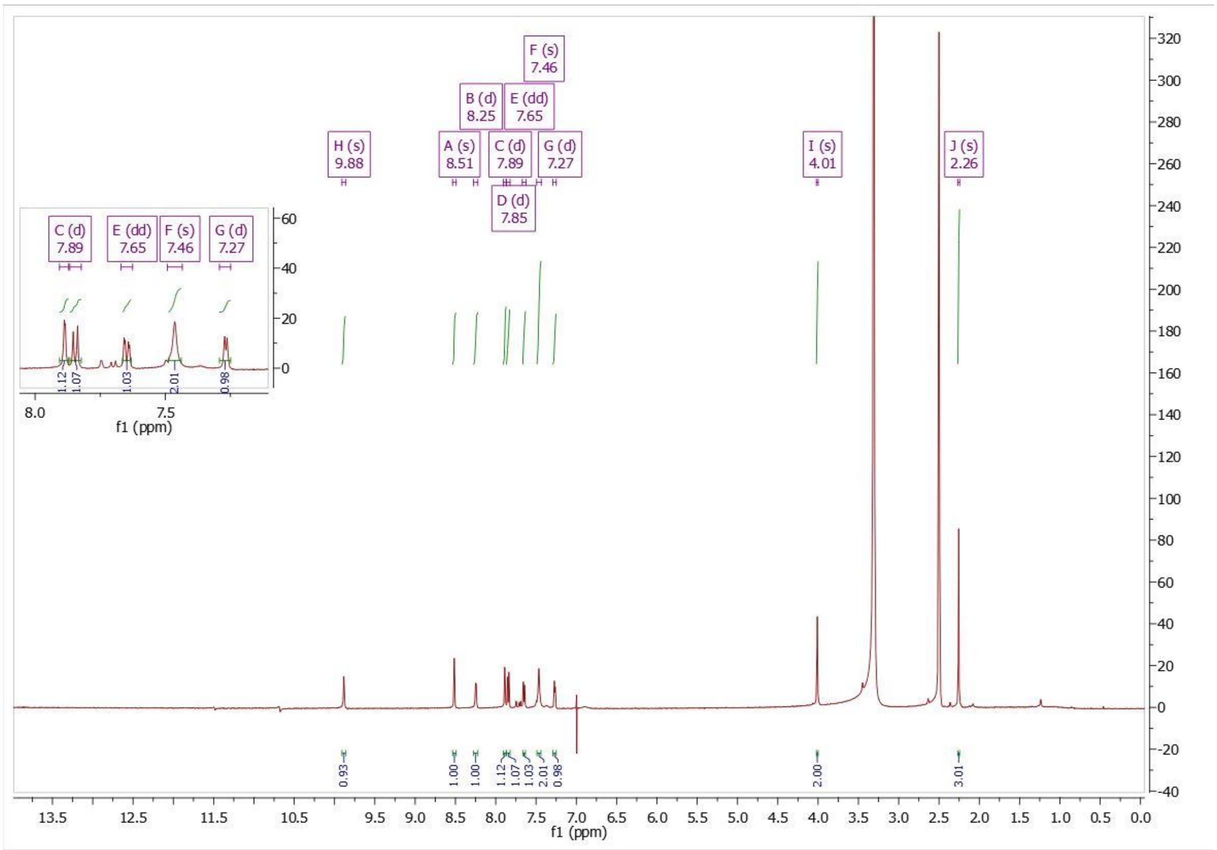
H (s) 9.88
A (s) 8.51
B (d) 8.25
C (d) 7.89
D (d) 7.85
E (dd) 7.65
F (s) 7.46
G (d) 7.27
I (s) 4.01
J (s) 2.26
0.93
1.00
1.00
1.12
1.07
1.03
2.01
0.98
2.00
3.01
f1 (ppm)

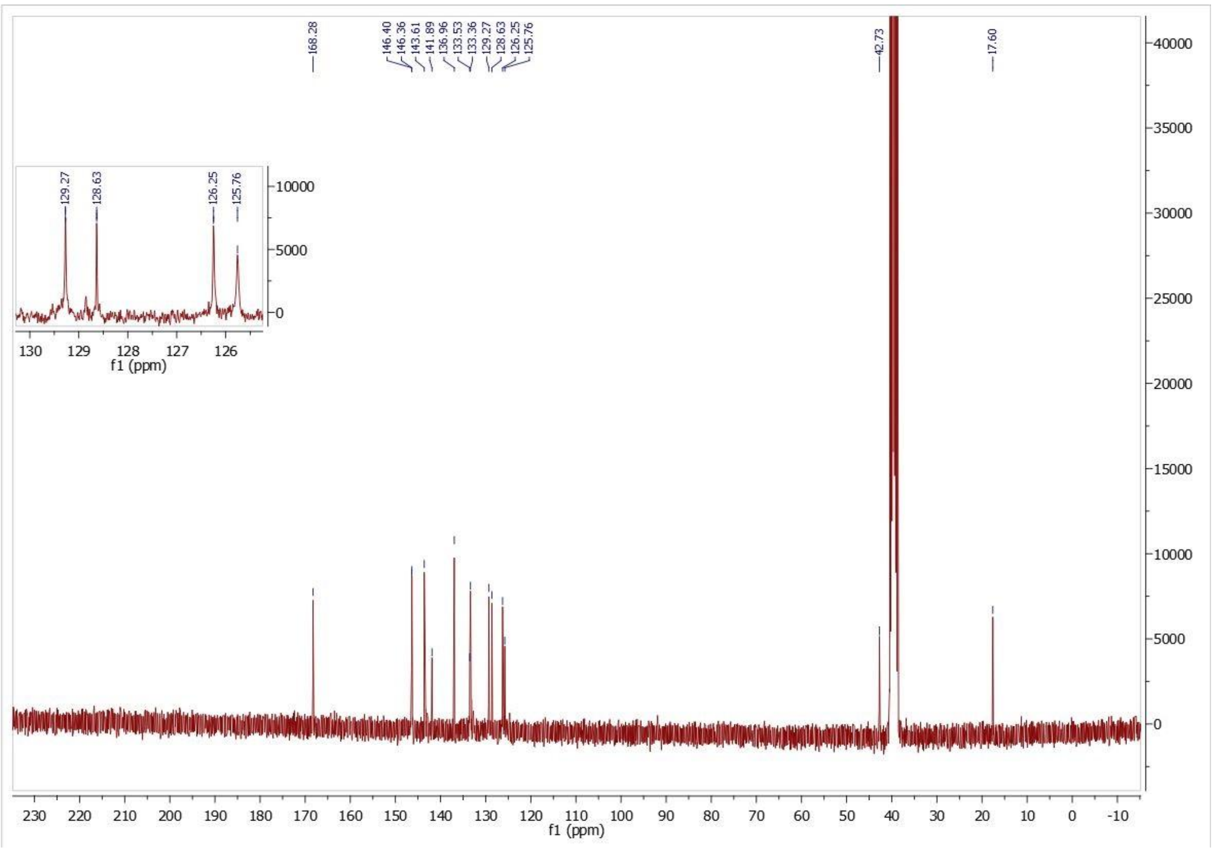
168.28
146.40
146.36
143.61
141.89
136.96
133.53
133.36
129.27
128.63
126.25
125.76
42.73
17.60
f1 (ppm)

N-[(2-benzyl-5-sulfamoylphenyl)methyl]-2-cyanopyrimidine-4-carboxamide (25)

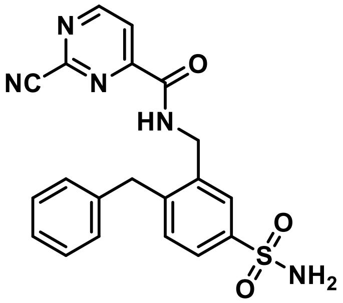

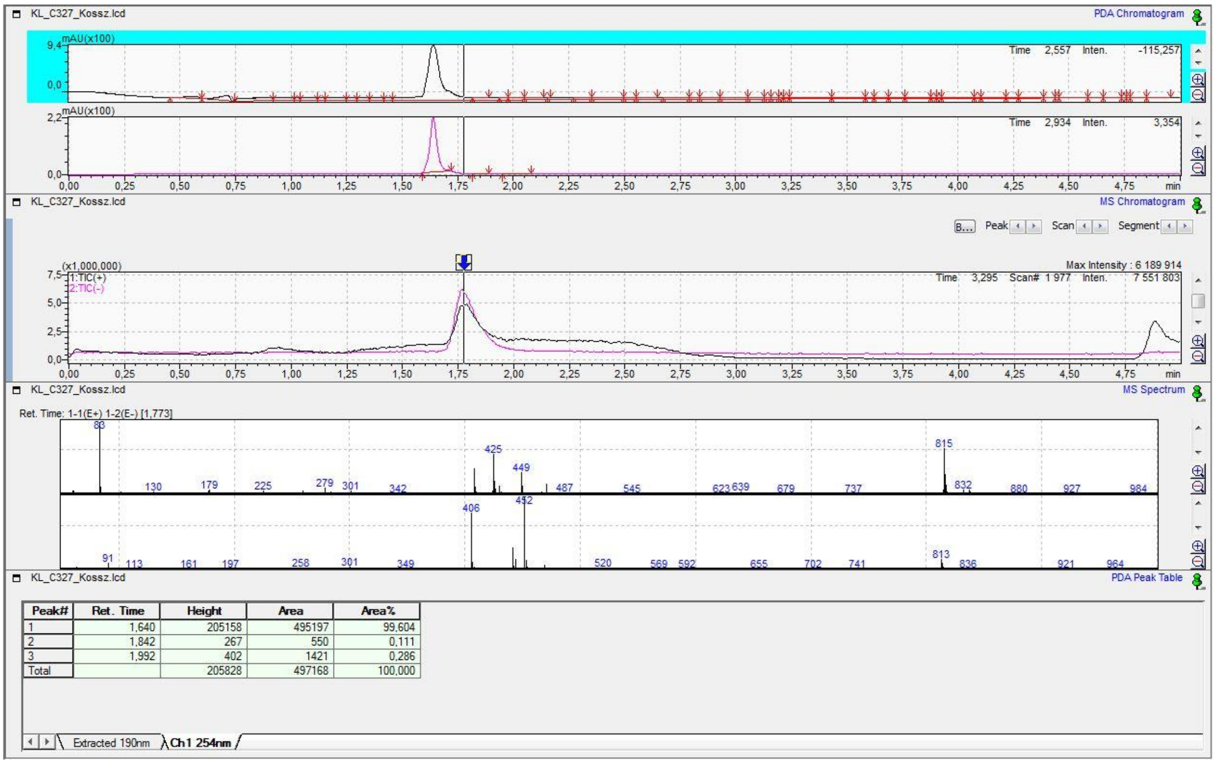

Peak#	Ret. Time	Height	Area	Area%
1	1,640	205158	495197	99,604
2	1,842	267	550	0,111
3	1,992	402	1421	0,286
Total		205828	497168	100,000

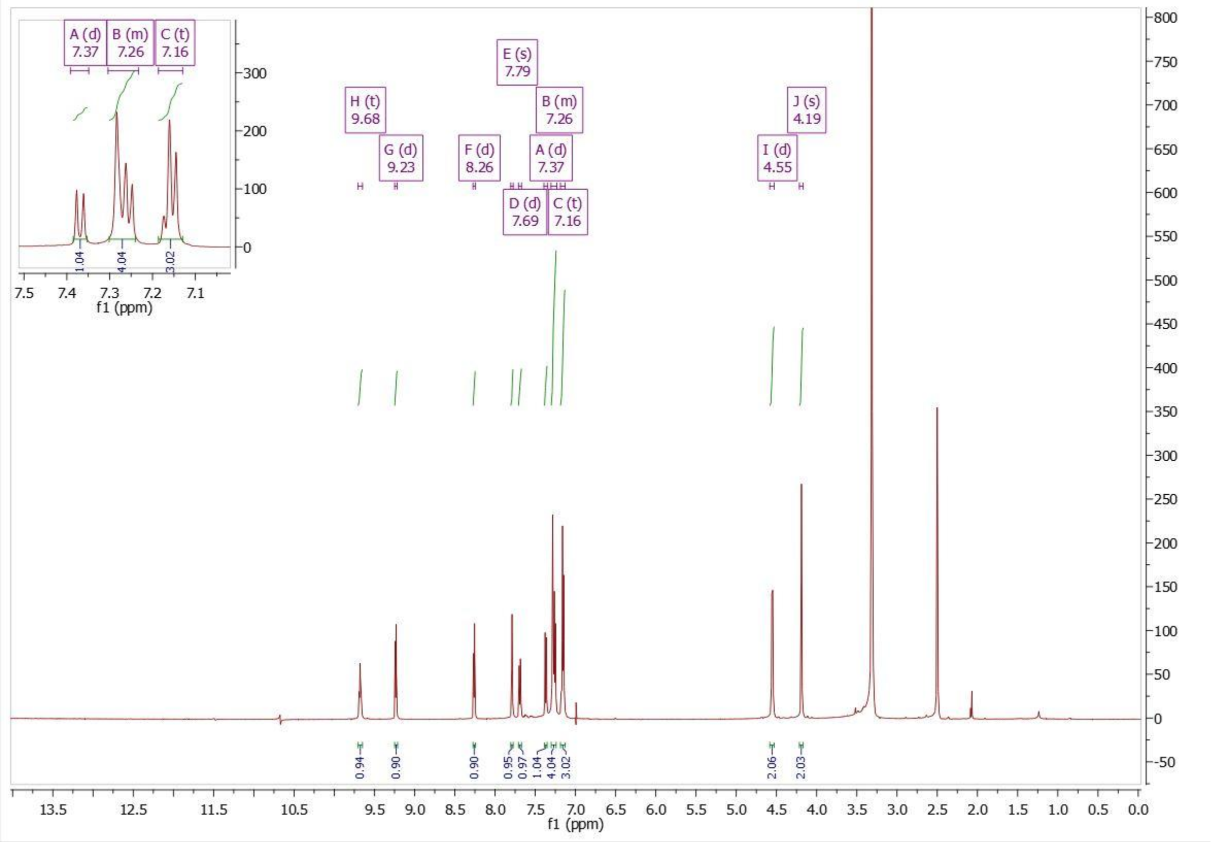

A (d) 7.37
B (m) 7.26
C (t) 7.16
E (s) 7.79
H (t) 9.68
G (d) 9.23
F (d) 8.26
D (d) 7.69
I (d) 4.55
J (s) 4.19
0.94 0.90 0.90 0.95 0.97 1.04 4.04 3.02 2.06 2.03
f1 (ppm)

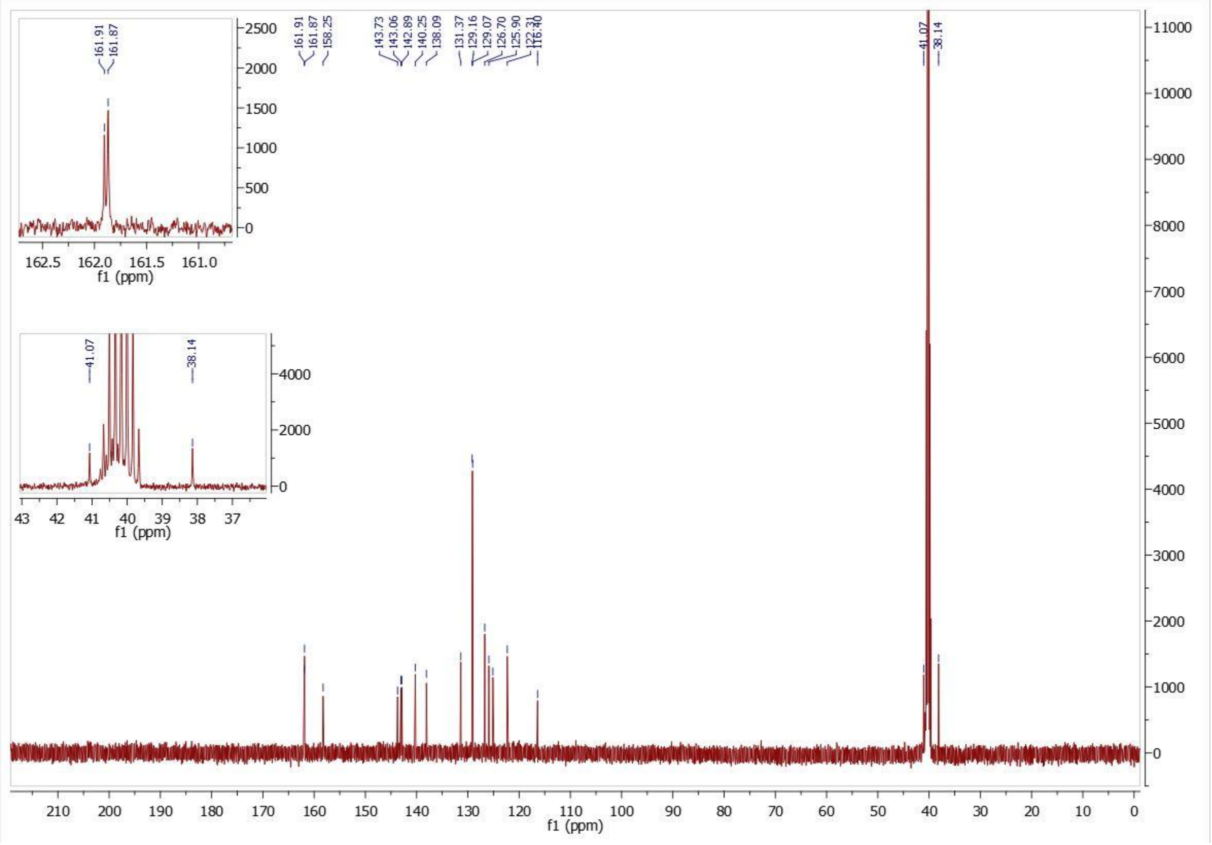

161.91
161.87
158.25
143.73
143.06
142.89
140.25
138.09
131.37
129.16
129.07
126.70
125.90
122.31
116.40
41.07
38.14
f1 (ppm)

N-{[2-(3-chlorophenoxy)-5-sulfamoylphenyl]methyl}-2-cyanopyrimidine-4-carboxamide (26)

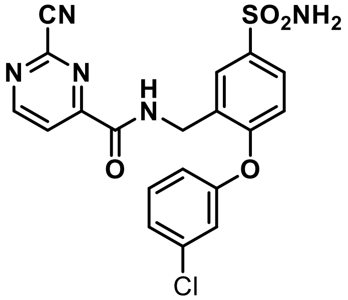

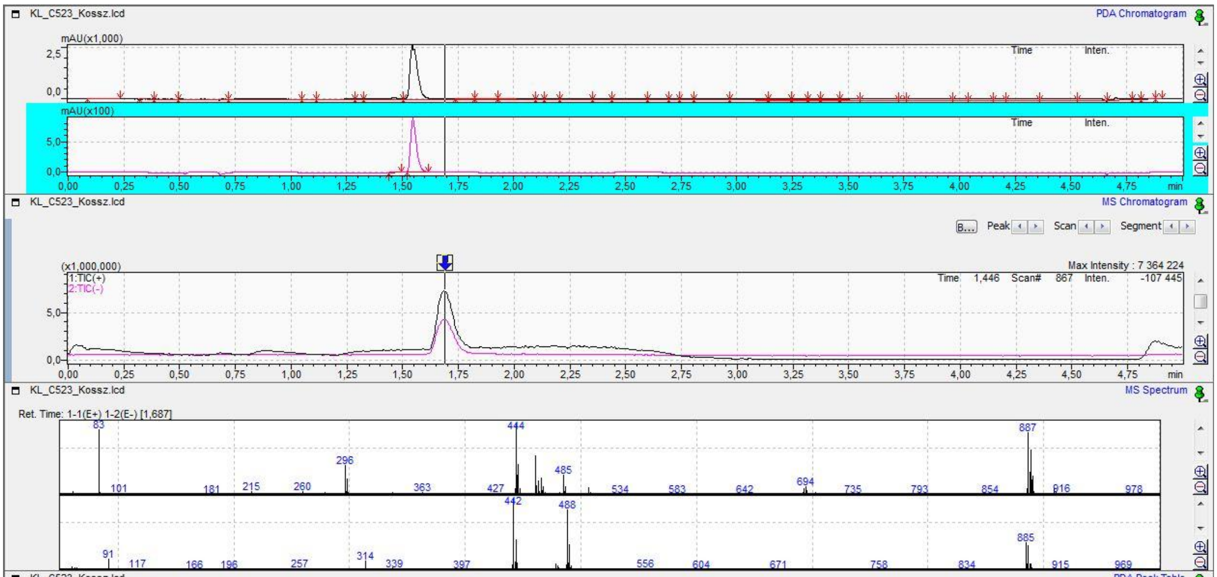

KL_C523_Kossz.lcd PDA Peak Table

Peak#	Ret. Time	Height	Area	Area%
1	1,463	5189	9241	0,575
2	1,548	868448	1597913	99,425
Total		873637	1607154	100,000

Extracted 190nm | Ch1 254nm

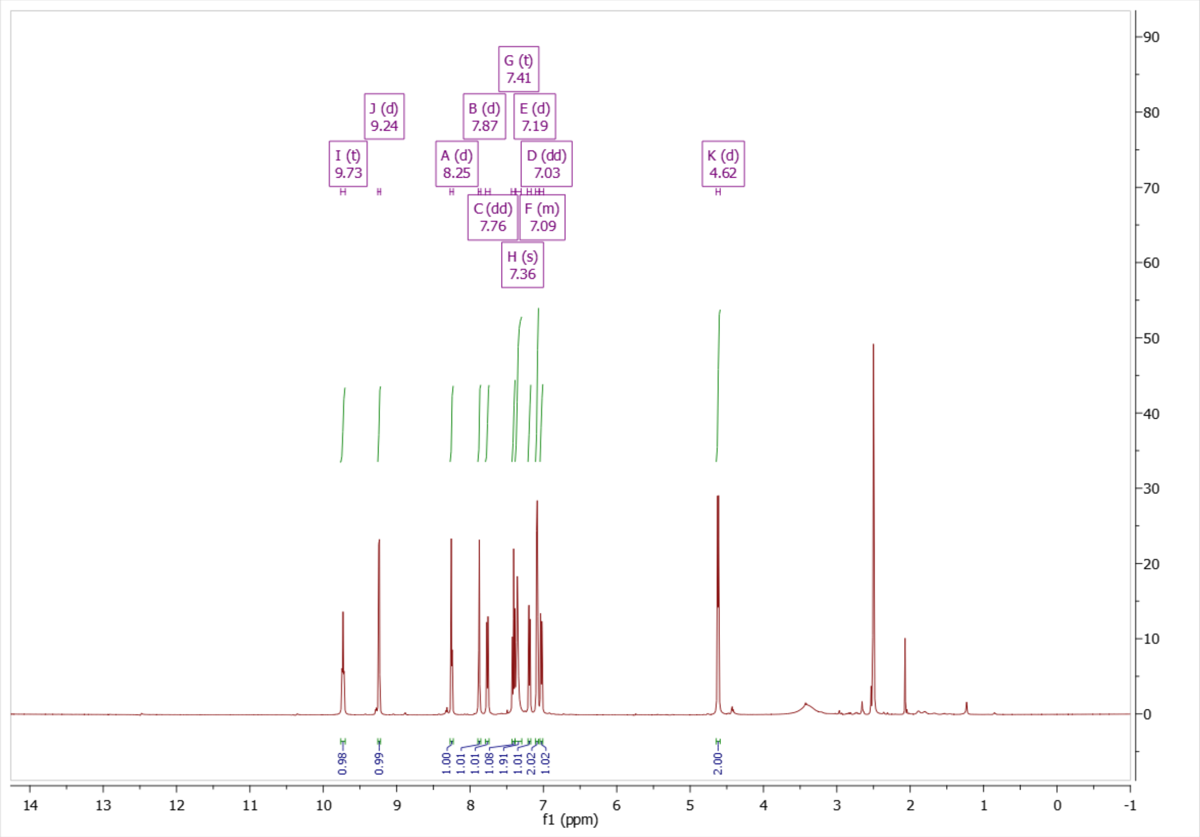
I (t) 9.73
J (d) 9.24
A (d) 8.25
B (d) 7.87
C (dd) 7.76
G (t) 7.41
H (s) 7.36
E (d) 7.19
F (m) 7.09
D (dd) 7.03
K (d) 4.62
0.98
0.99
1.00
1.01
1.01
1.08
1.91
1.01
2.02
1.02
2.00
f1 (ppm)

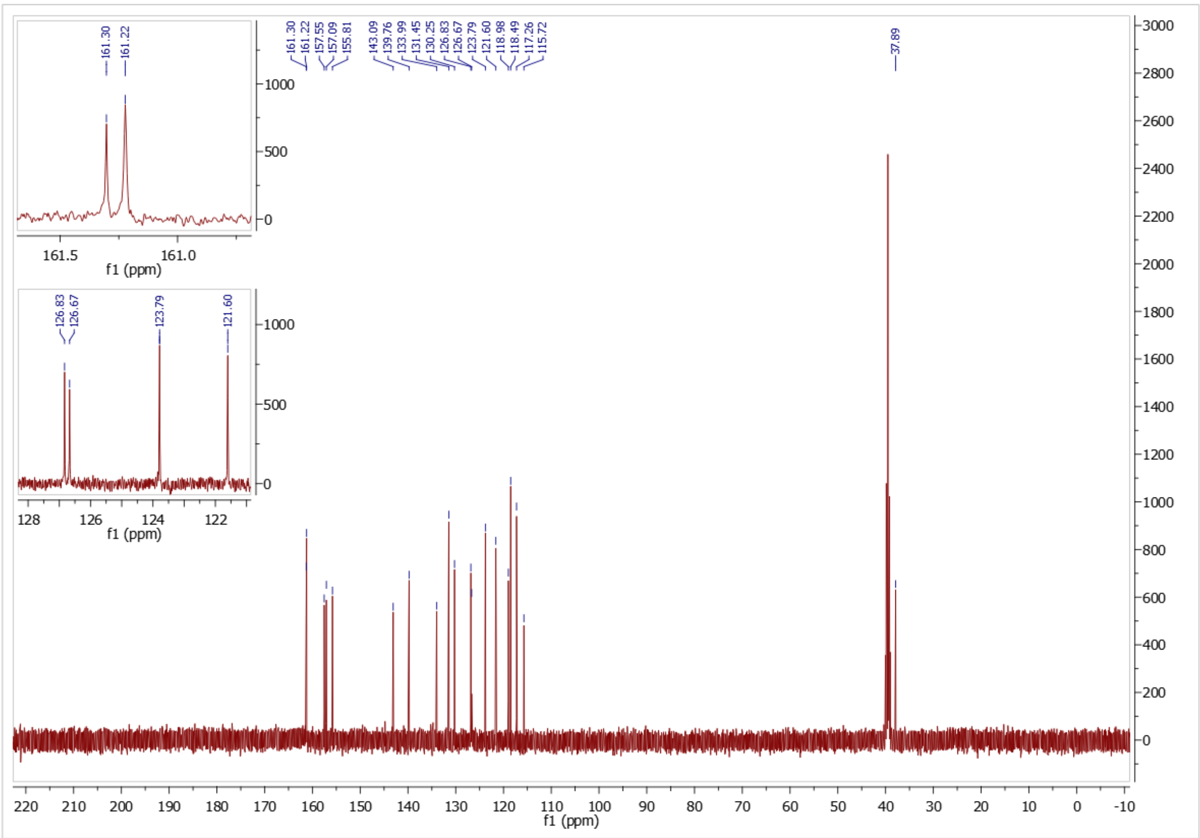
161.30
161.22
157.55
157.09
155.81
143.09
139.76
133.99
131.45
130.25
126.83
126.67
123.79
121.60
118.98
118.49
117.26
115.72
37.89
f1 (ppm)

2-Cyano-*N*-{[5-sulfamoyl-2-(4-sulfamoylphenoxy)phenyl]methyl}pyrimidine-4-carboxamide (27)

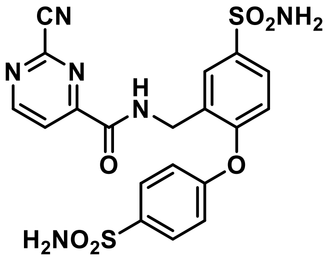

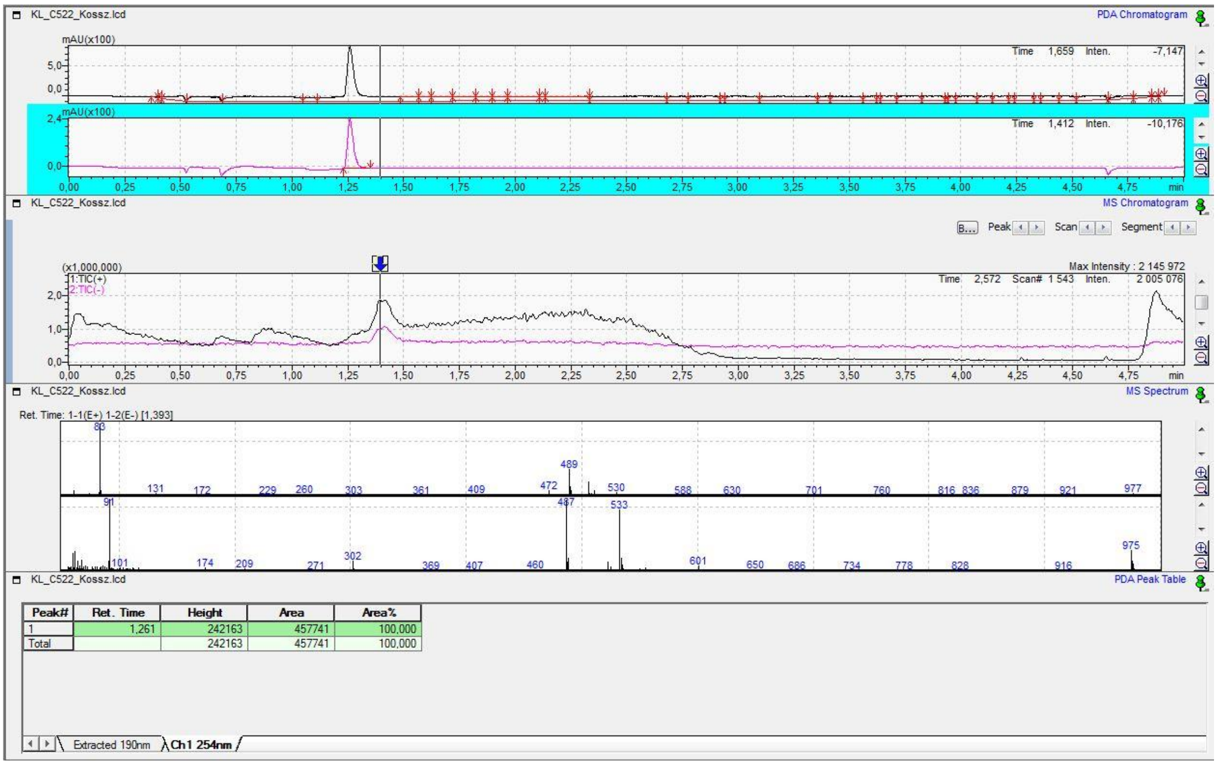

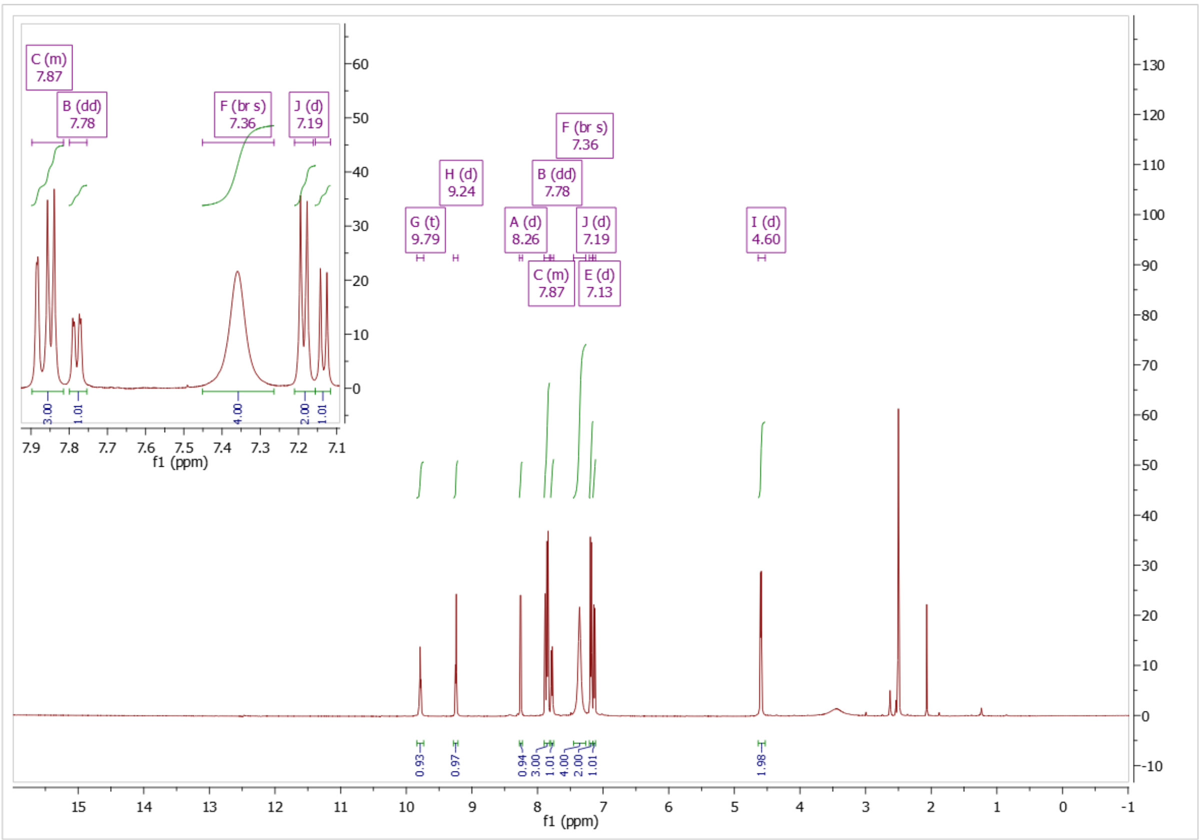

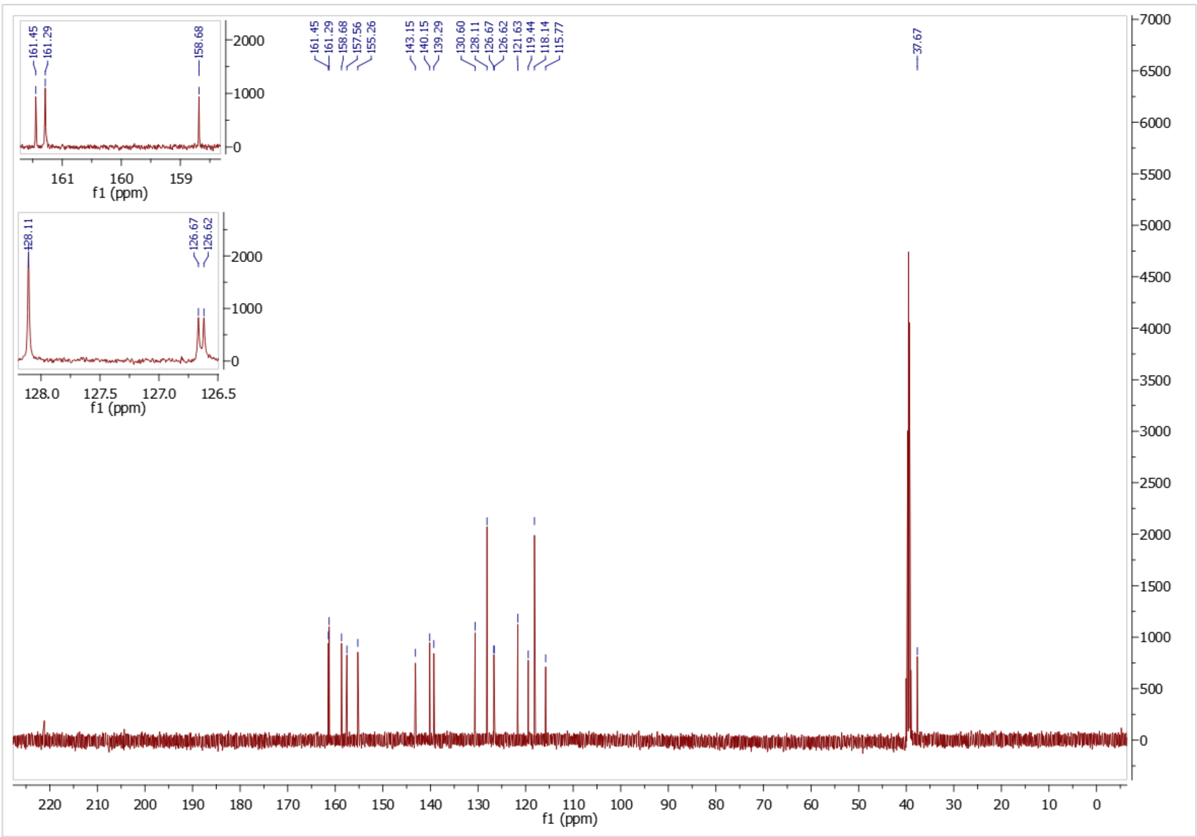

***N*-{[2-bromo-5-(phenylsulfamoyl)phenyl]methyl}-2-cyanopyrimidine-4-carboxamide (28)**

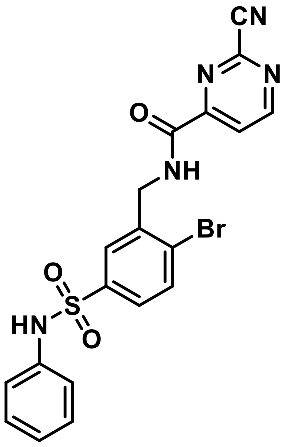

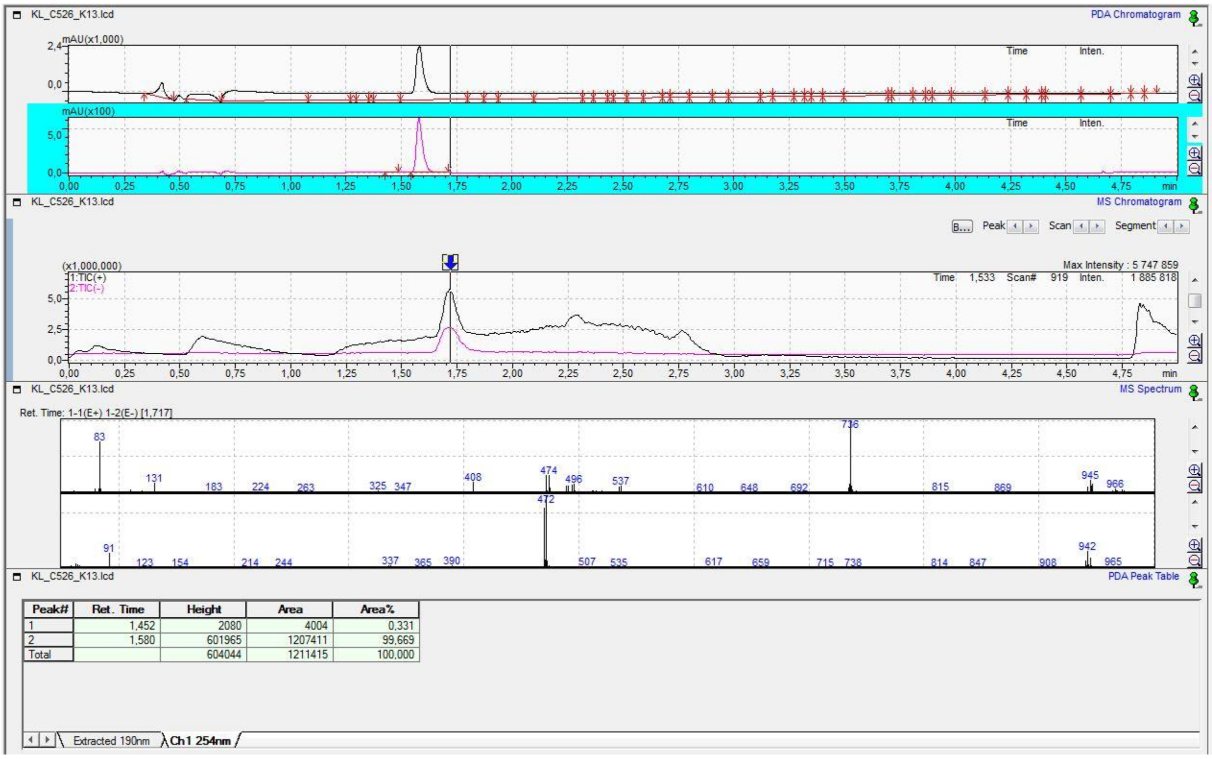

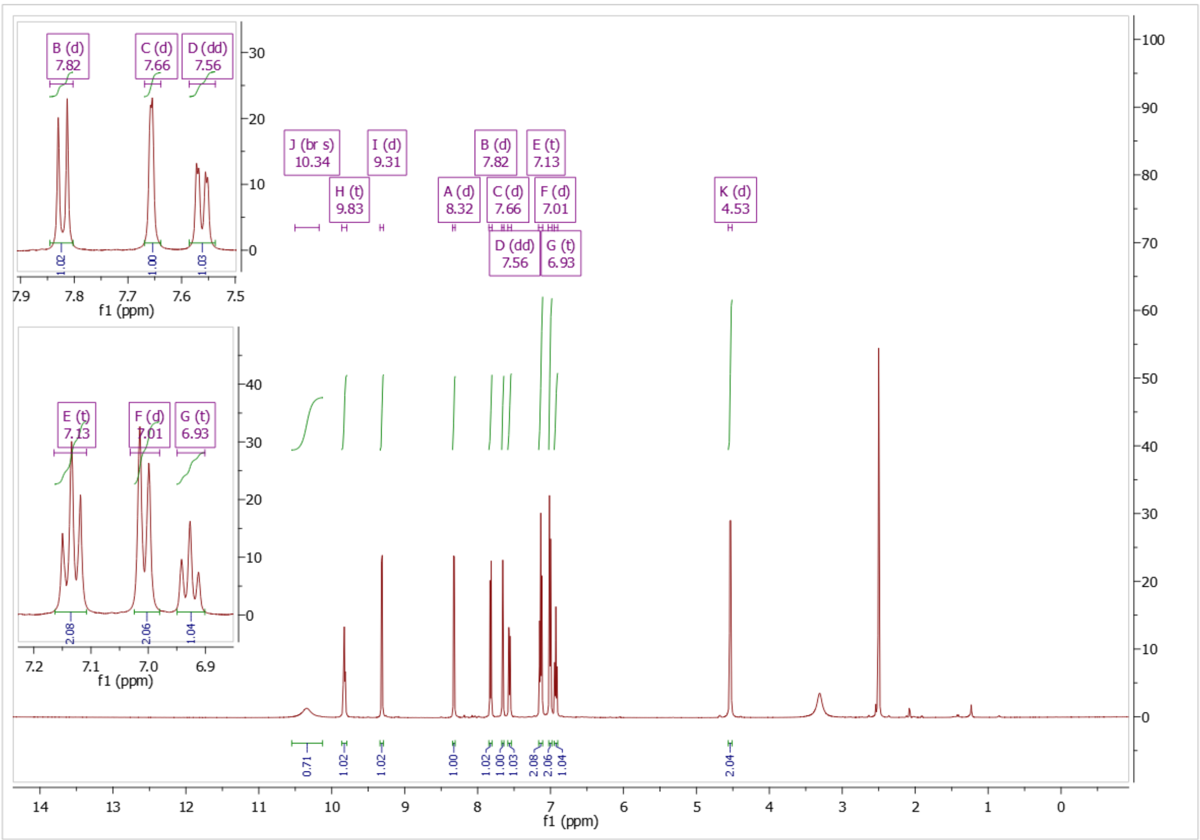

J (br s)
10.34
H (t)
9.83
I (d)
9.31
A (d)
8.32
B (d)
7.82
C (d)
7.66
D (dd)
7.56
E (t)
7.13
F (d)
7.01
G (t)
6.93
K (d)
4.53
f1 (ppm)

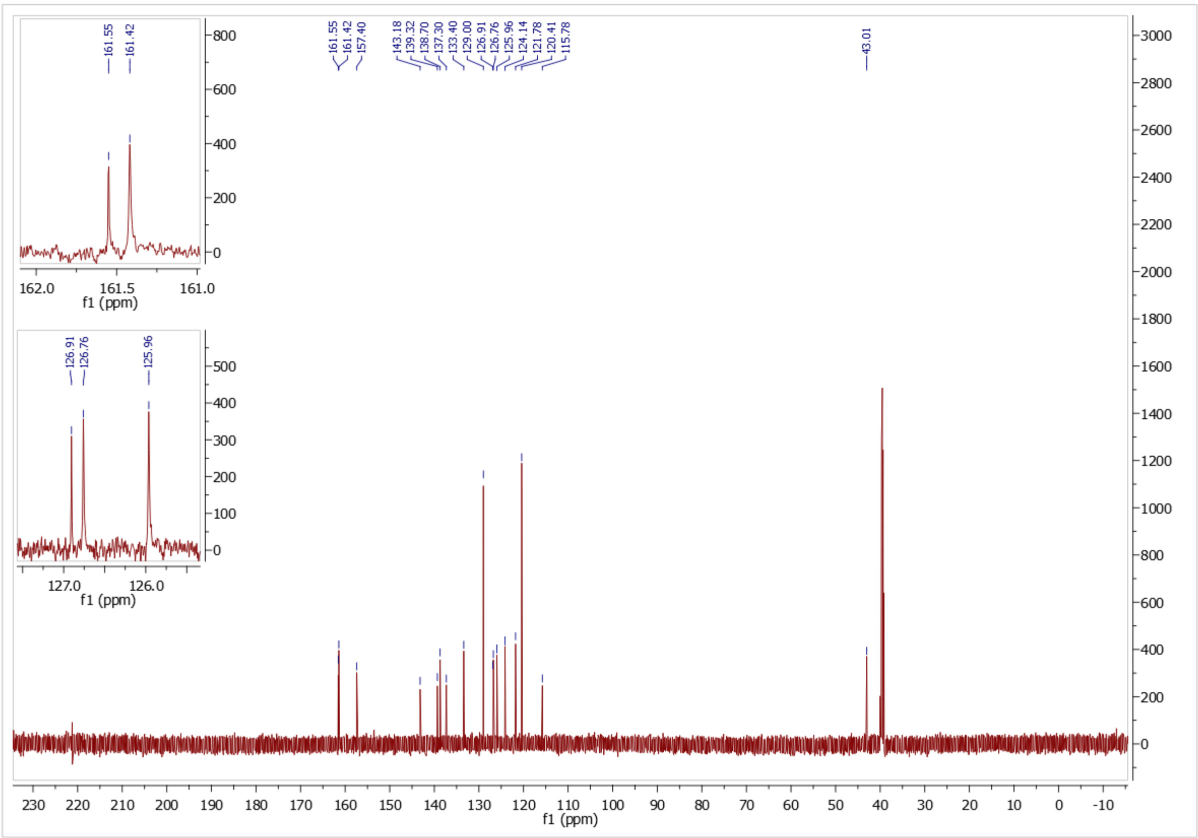

161.55
161.42
157.40
143.18
139.32
138.70
137.30
133.40
129.00
126.91
126.76
125.96
124.14
121.78
120.41
115.78
43.01
f1 (ppm)

3-(Aminomethyl)-4-benzylbenzene-1-sulfonic acid (30)

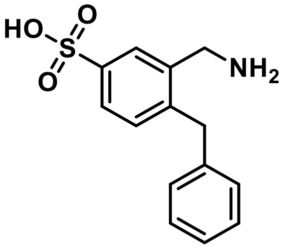

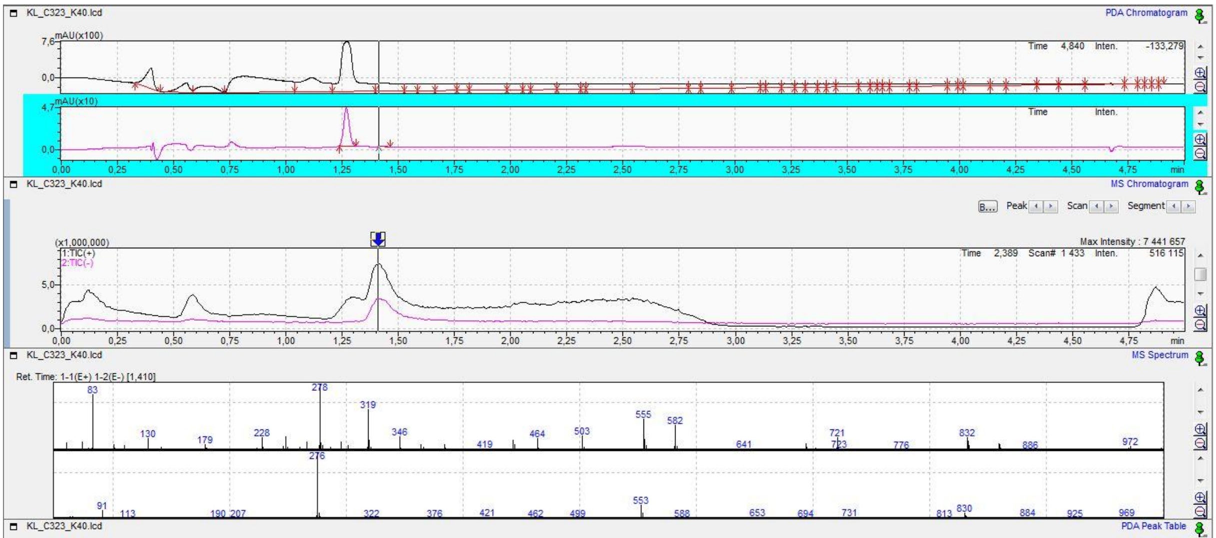

Peak#	Ret. Time	Height	Area	Area%
1	1,269	42029	74381	98,796
2	1,432	542	907	1,204
Total		42570	75288	100,000

Extracted 190nm | Ch1 254nm

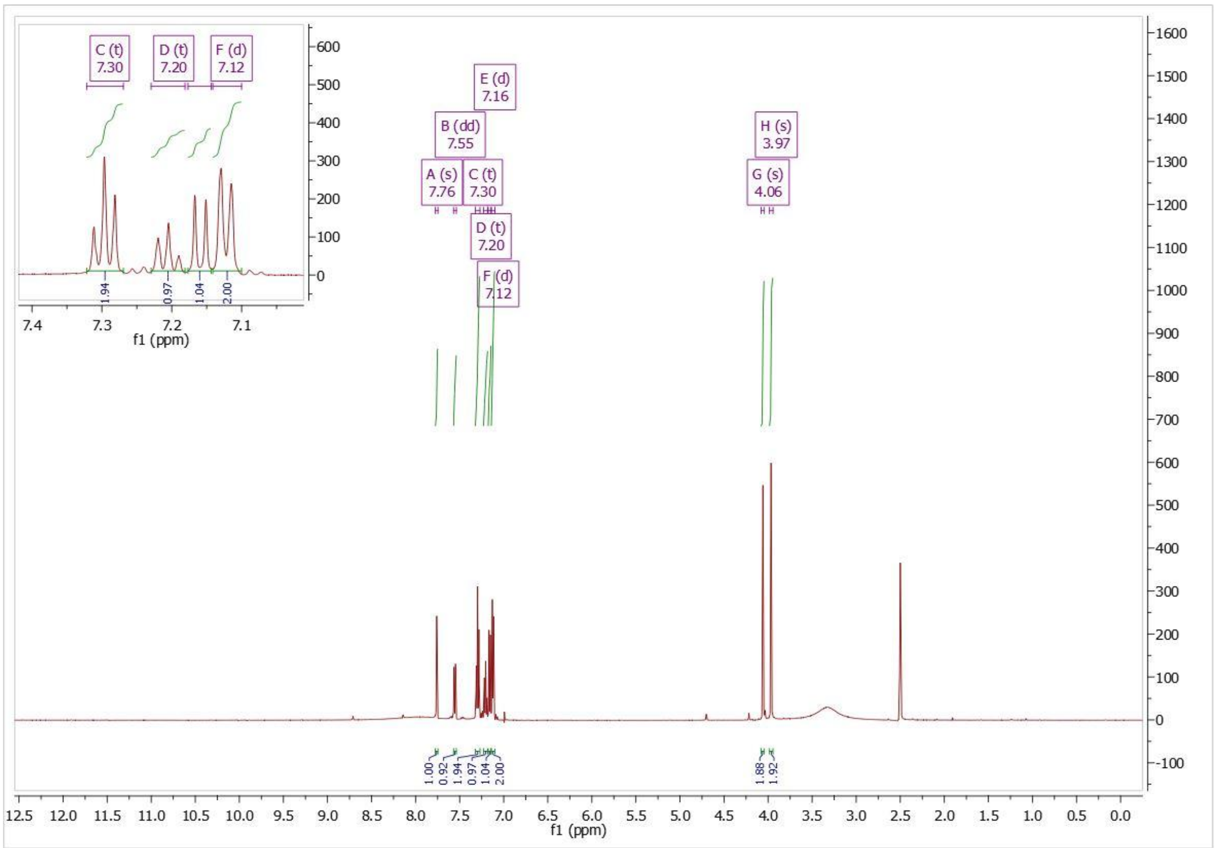

C (t) 7.30
D (t) 7.20
F (d) 7.12
1.94
0.97
1.04
2.00
E (d) 7.16
B (dd) 7.55
A (s) 7.76
C (t) 7.30
D (t) 7.20
F (d) 7.12
H (s) 3.97
G (s) 4.06
1.00
0.92
1.94
0.97
1.04
2.00
1.88
1.92
f1 (ppm)

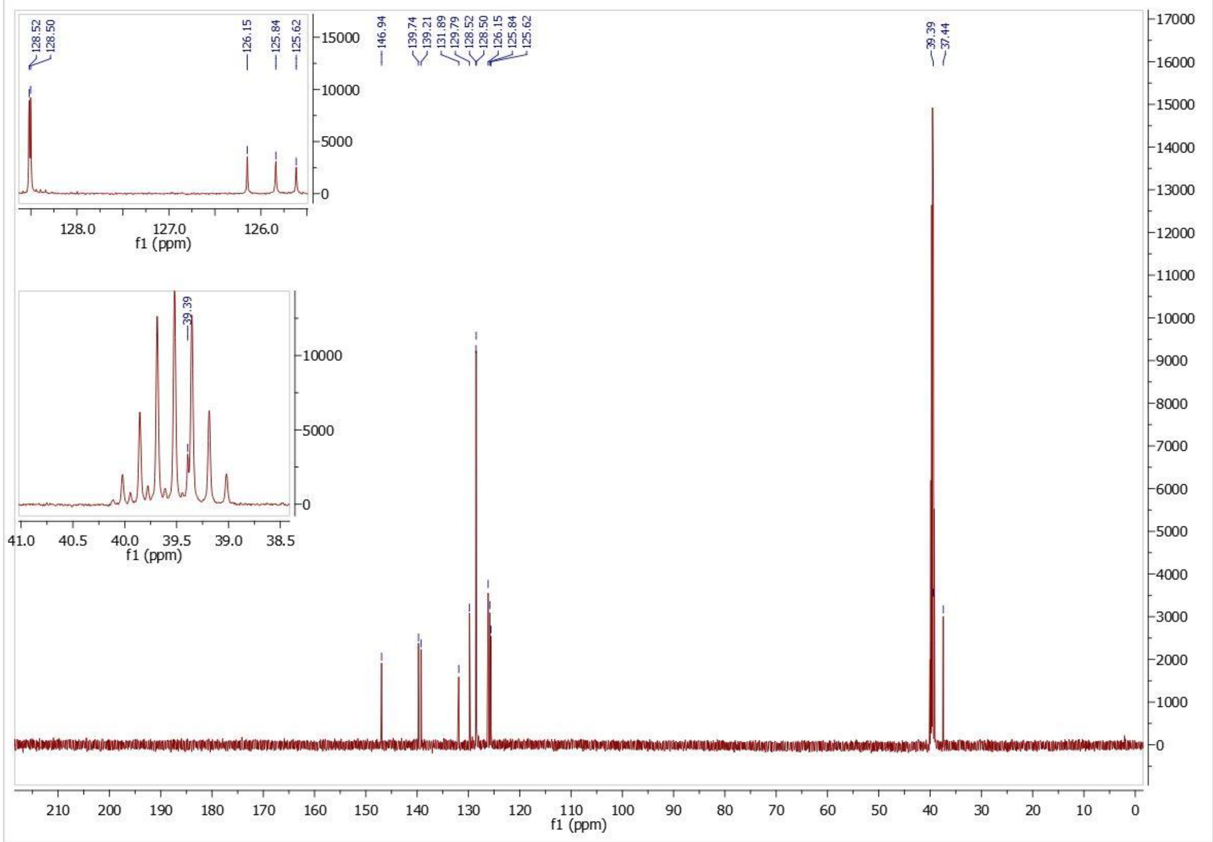

128.52
128.50
126.15
125.84
125.62
146.94
139.74
139.21
131.89
129.79
128.52
128.50
126.15
125.84
125.62
39.39
37.44
f1 (ppm)
39.39

Ethylbis(propan-2-yl)azanium 4-benzyl-3-[[(2-chloropyrimidin-4-yl)formamido]methyl]benzene-1-sulfonate (31)

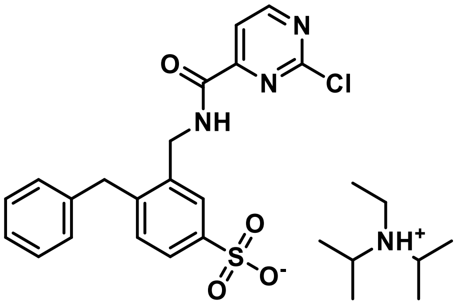

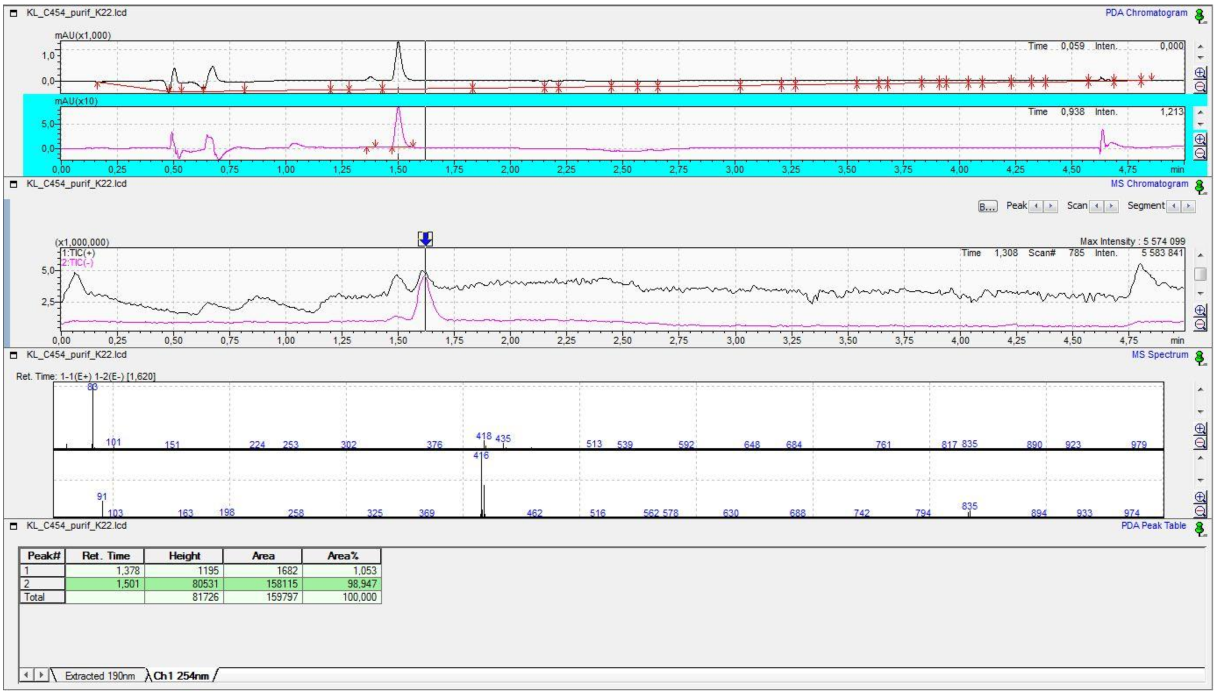

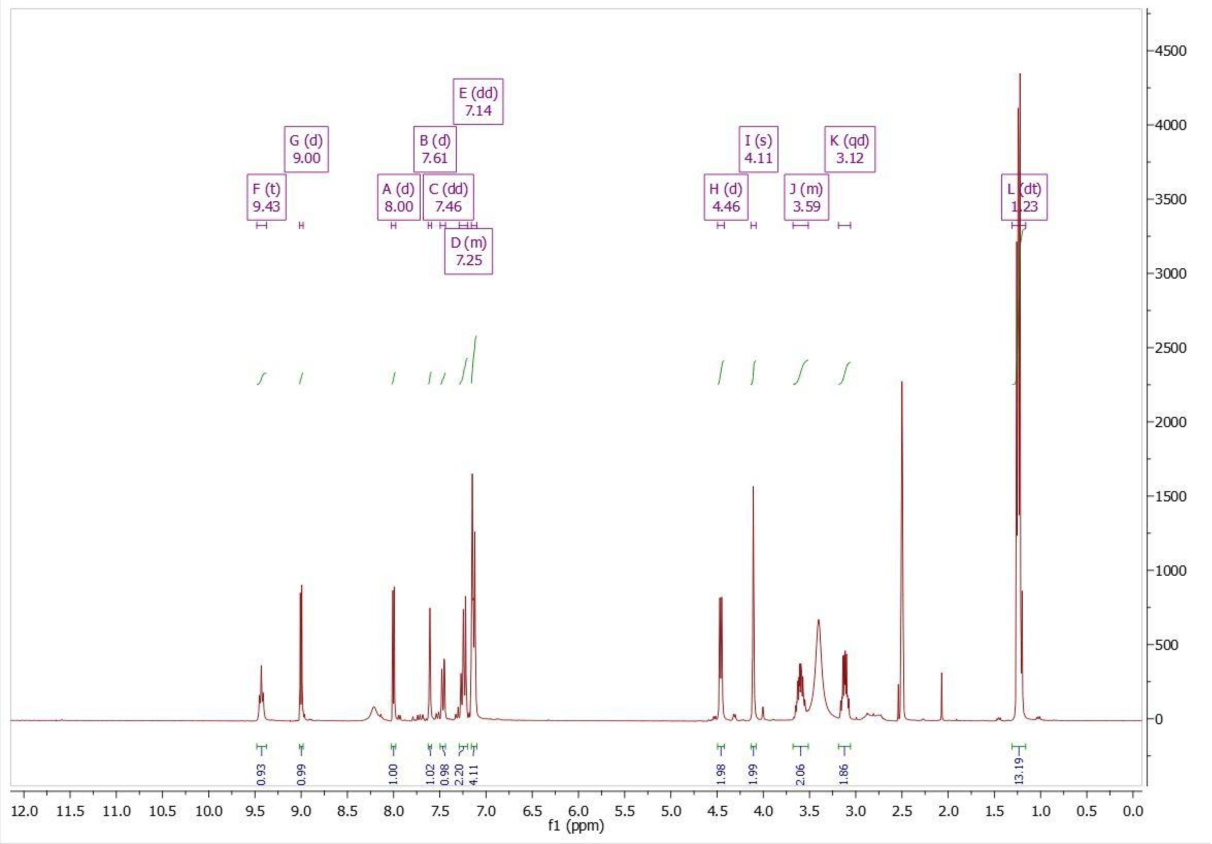
F (t) 9.43
G (d) 9.00
A (d) 8.00
B (d) 7.61
C (dd) 7.46
E (dd) 7.14
D (m) 7.25
H (d) 4.46
I (s) 4.11
J (m) 3.59
K (qd) 3.12
L (dt) 1.23
0.93 0.99 1.00 1.02 0.98 2.20 4.11 1.98 1.99 2.06 1.86 13.19
f1 (ppm)

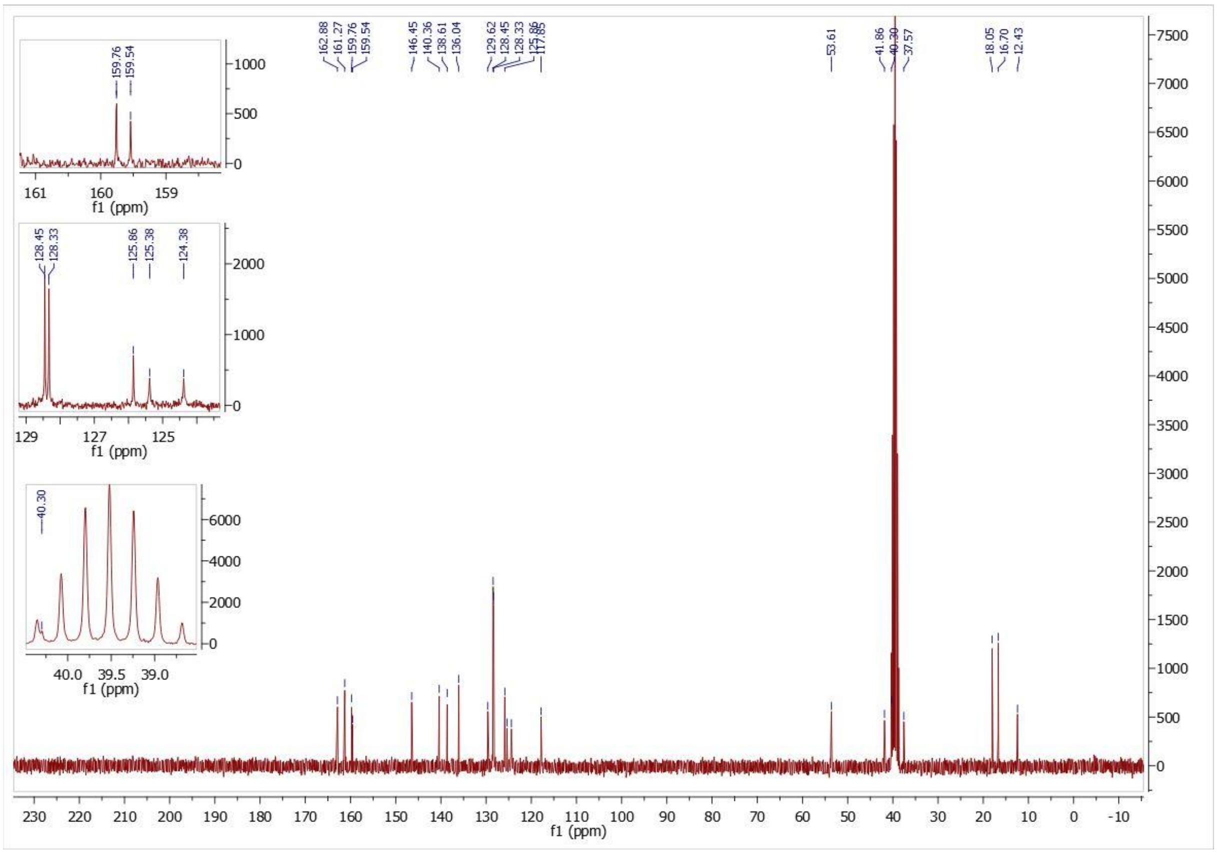
162.88 161.27 159.76 159.54 146.45 140.36 138.61 136.04 129.62 128.45 128.33 125.86 117.85 53.61 41.86 40.30 37.57 18.05 16.70 12.43
159.76 159.54
128.45 128.33 125.86 125.38 124.38
40.30
f1 (ppm)

N-[(2-benzyl-5-sulfamoylphenyl)methyl]-2-chloropyrimidine-4-carboxamide (32)

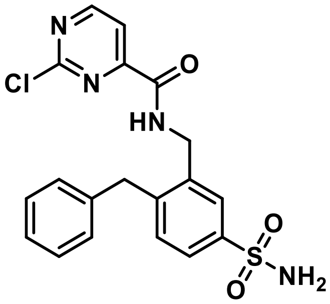

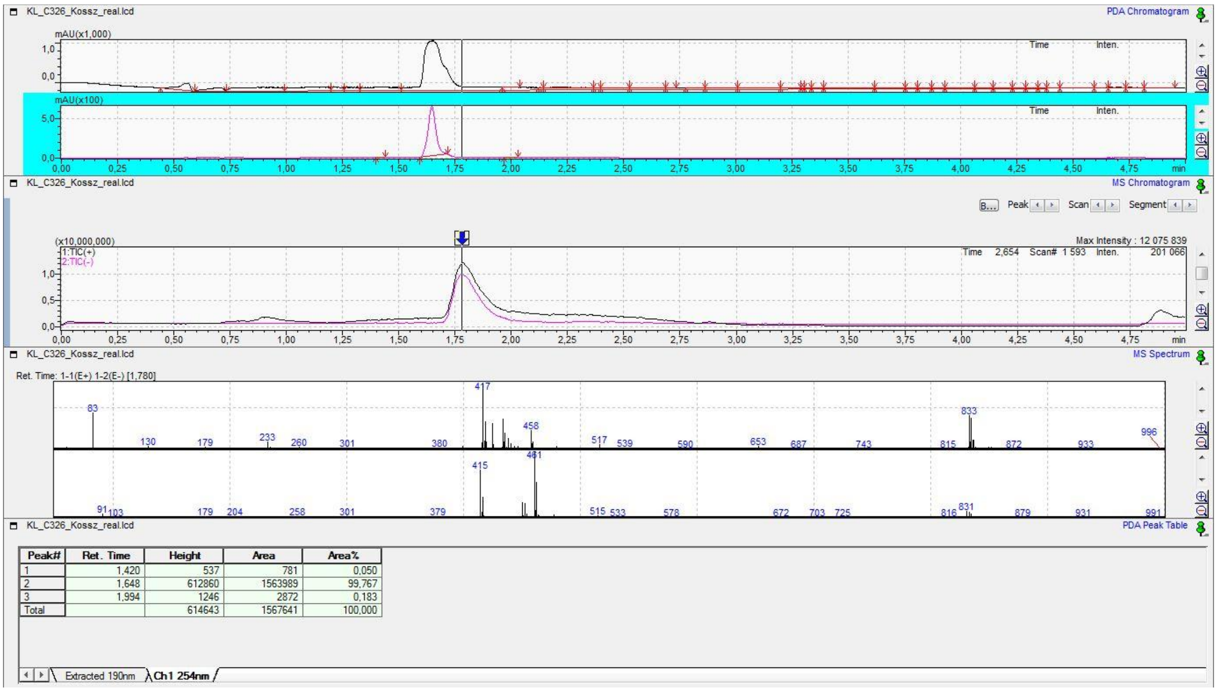

Peak#	Ret. Time	Height	Area	Area%
1	1,420	537	781	0,050
2	1,648	612860	1563989	99,767
3	1,994	1246	2872	0,183
Total		614643	1567641	100,000

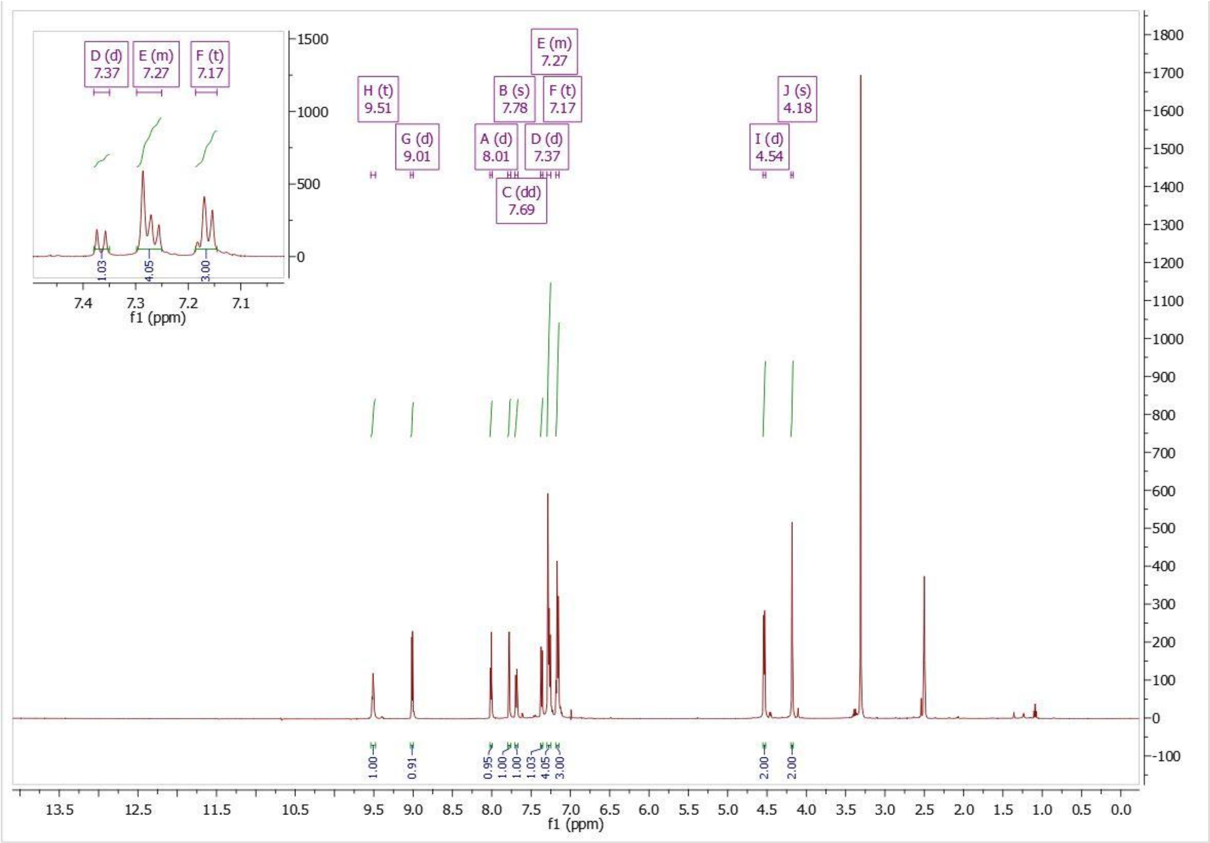
H (t)
9.51
G (d)
9.01
A (d)
8.01
B (s)
7.78
C (dd)
7.69
D (d)
7.37
E (m)
7.27
F (t)
7.17
I (d)
4.54
J (s)
4.18
f1 (ppm)

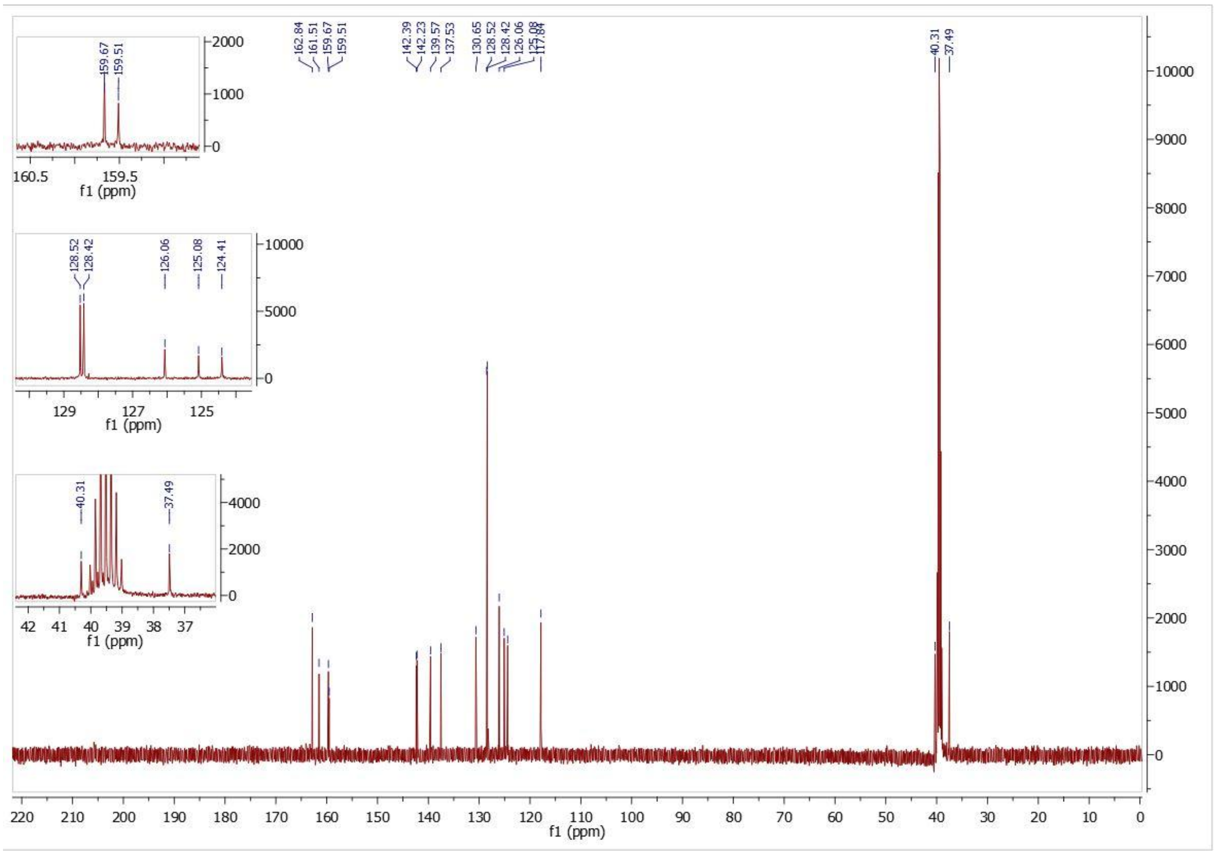
162.84
161.51
159.67
159.51
142.39
142.23
139.57
137.53
130.65
128.52
128.42
126.06
125.08
124.41
117.84
40.31
37.49
f1 (ppm)

3-Cyano-4-(4-sulfamoylphenoxy)benzene-1-sulfonamide (36)

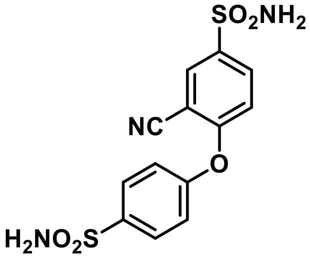

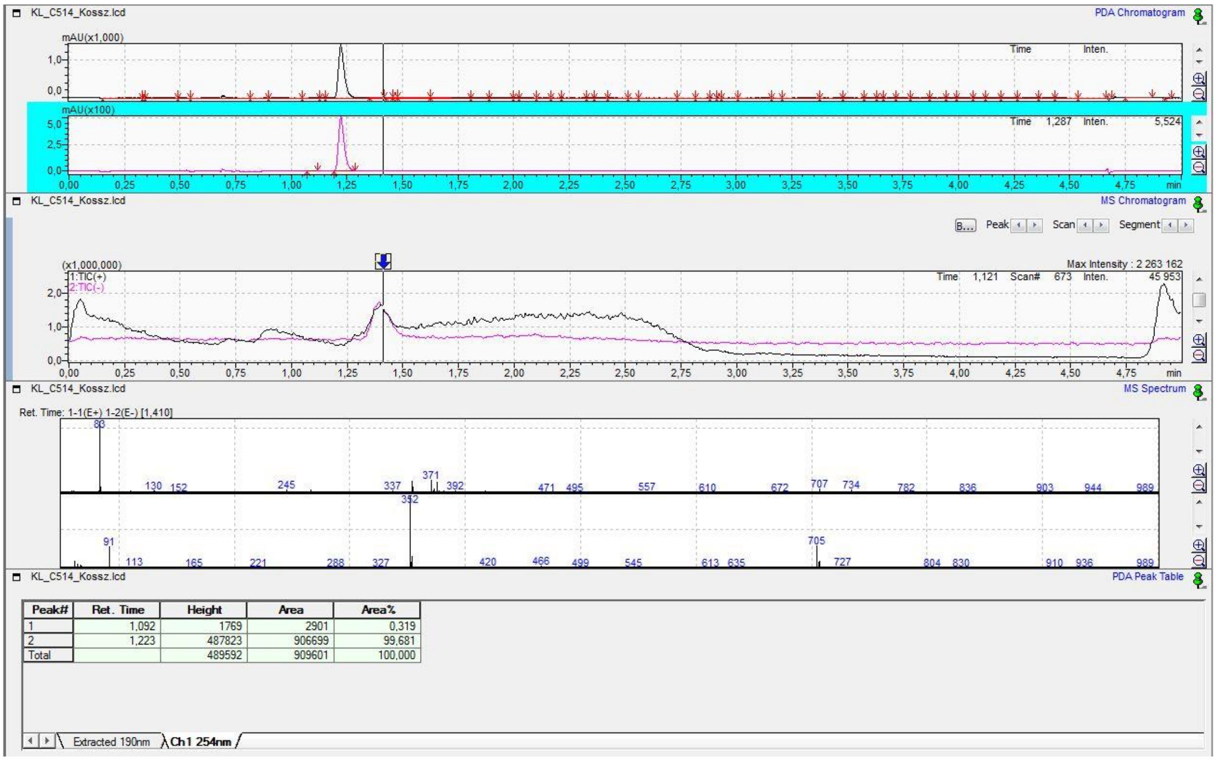

Peak#	Ret. Time	Height	Area	Area%
1	1,092	1769	2901	0,319
2	1,223	487823	906699	99,681
Total		489592	909601	100,000

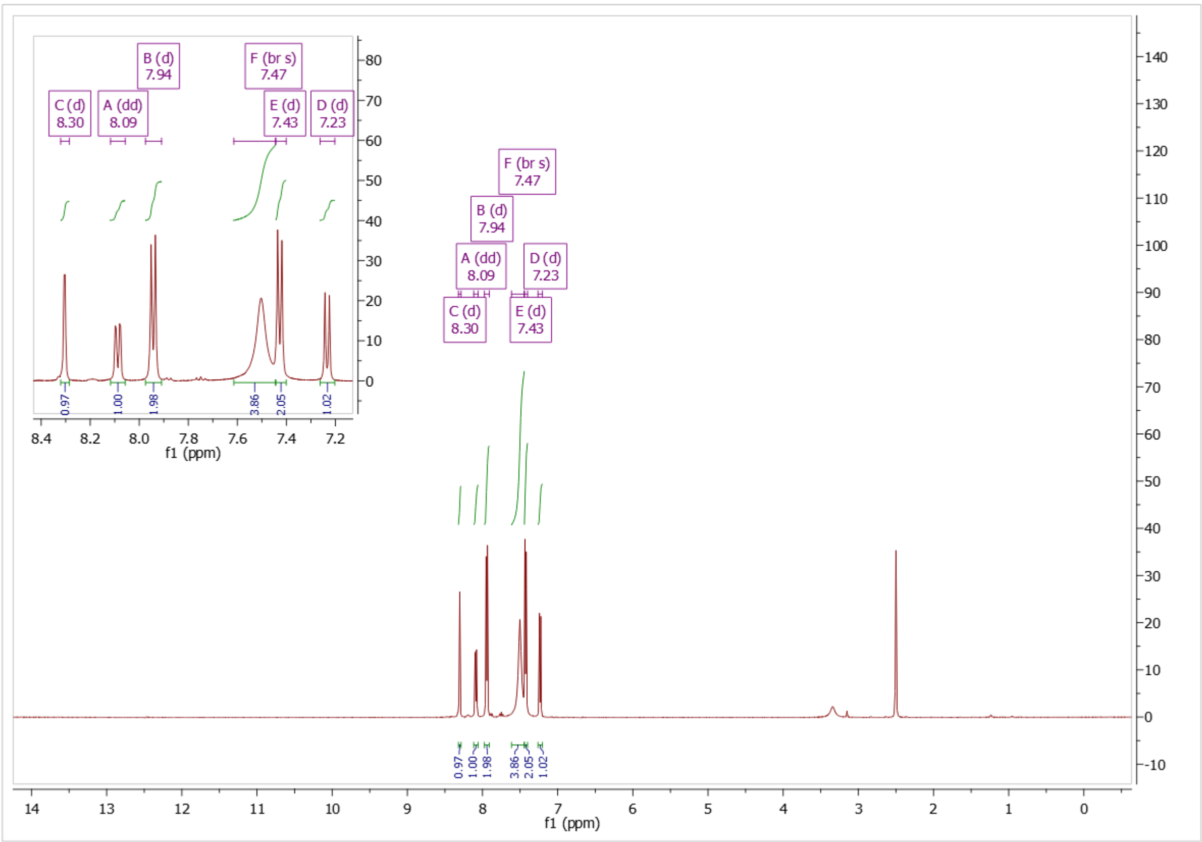
C (d) 8.30
A (dd) 8.09
B (d) 7.94
F (br s) 7.47
E (d) 7.43
D (d) 7.23
0.97
1.00
1.98
3.86
2.05
1.02
f1 (ppm)

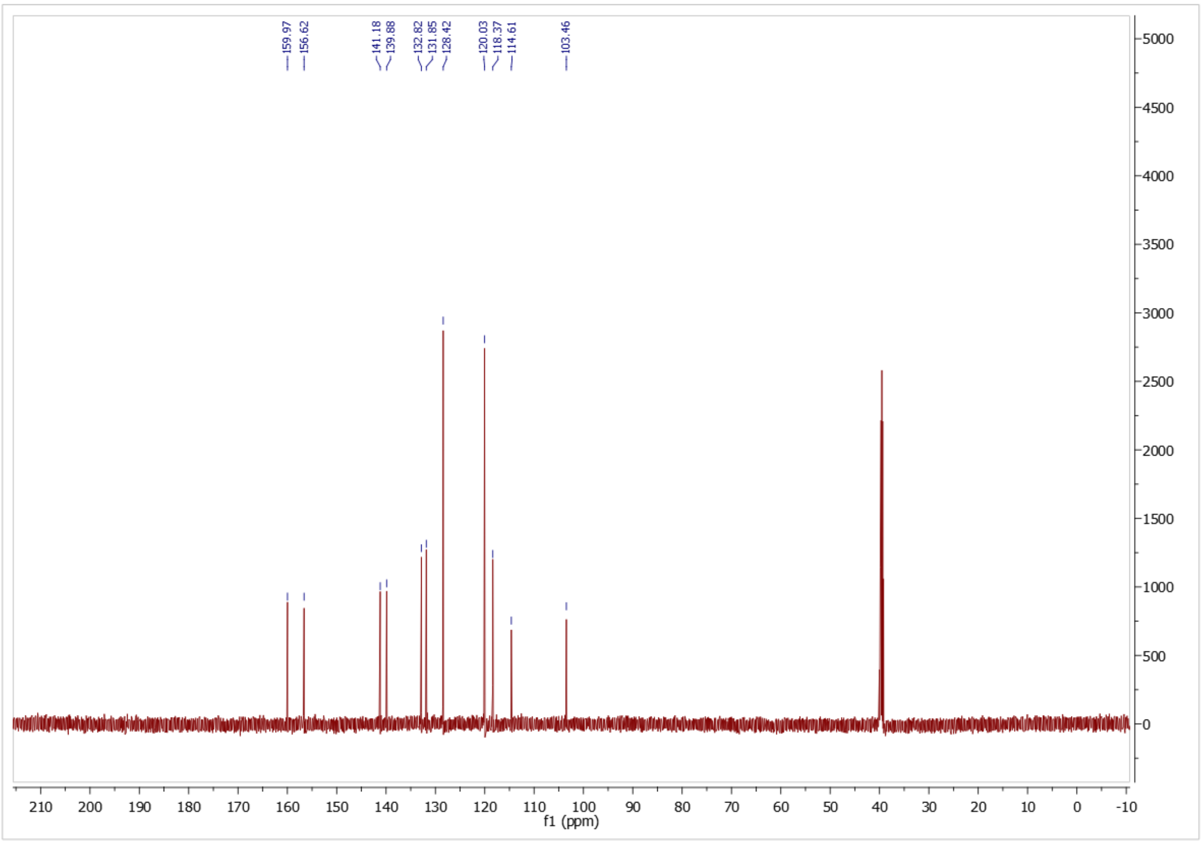
159.97
156.62
141.18
139.88
132.82
131.85
128.42
120.03
118.37
114.61
103.46
f1 (ppm)

4-(3-Chlorophenoxy)-3-cyanobenzene-1-sulfonamide (37)

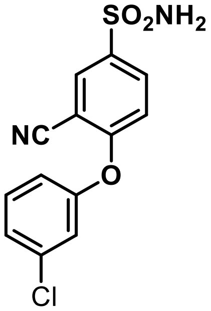

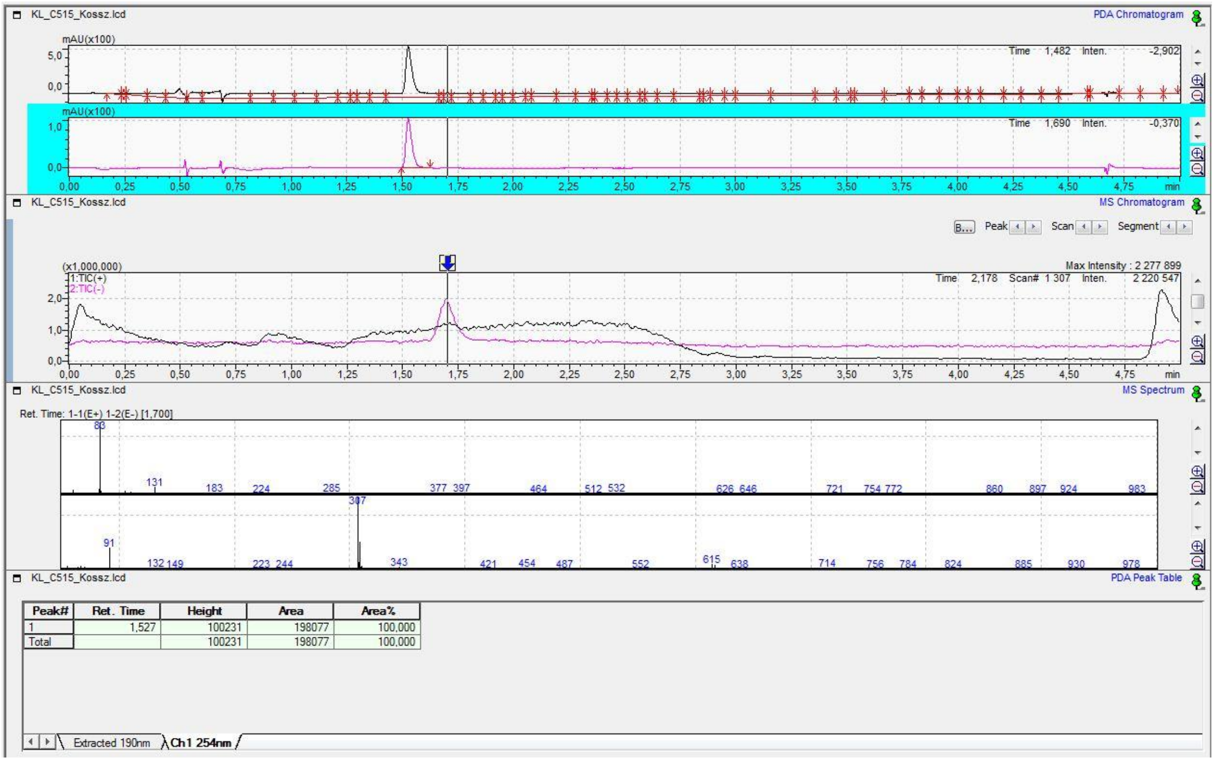

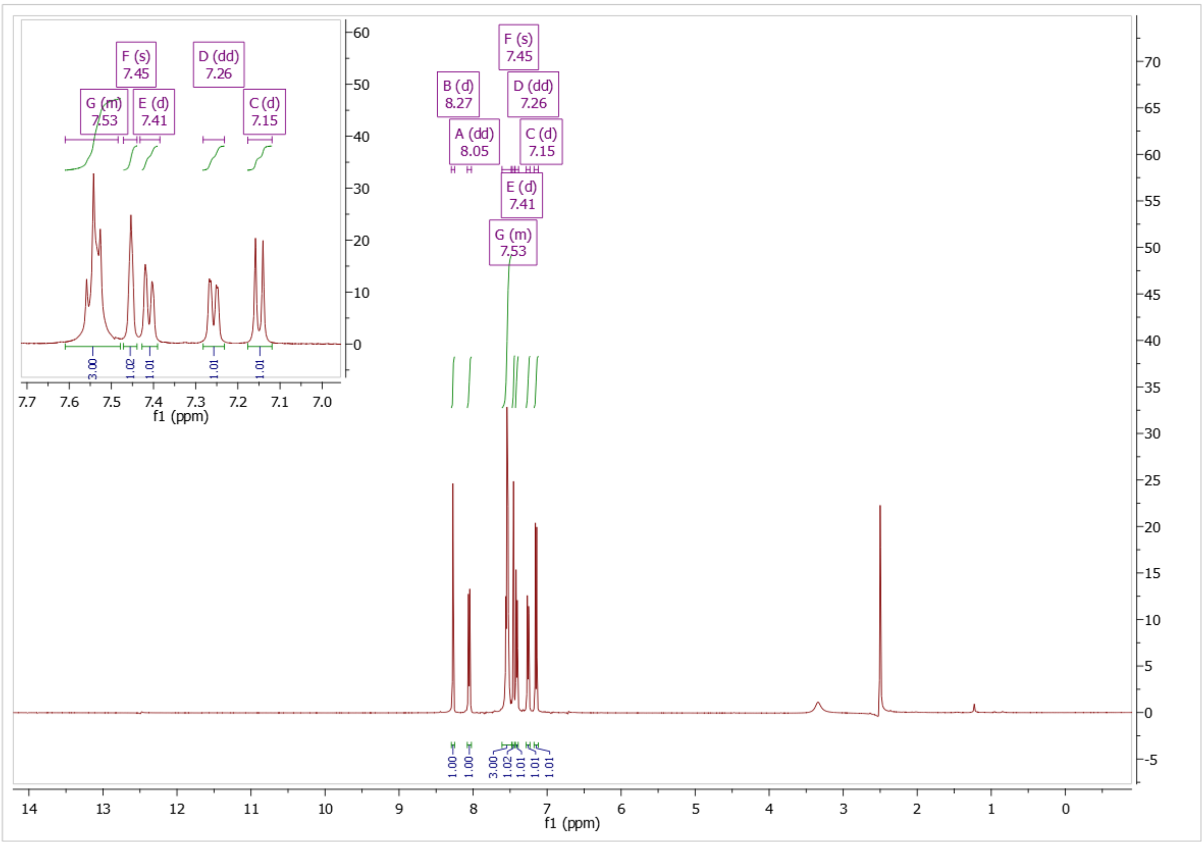
F (s) 7.45
D (dd) 7.26
G (m) 7.53
E (d) 7.41
C (d) 7.15
B (d) 8.27
A (dd) 8.05
3.00
1.02
1.01
1.01
1.01
1.00
1.00
f1 (ppm)

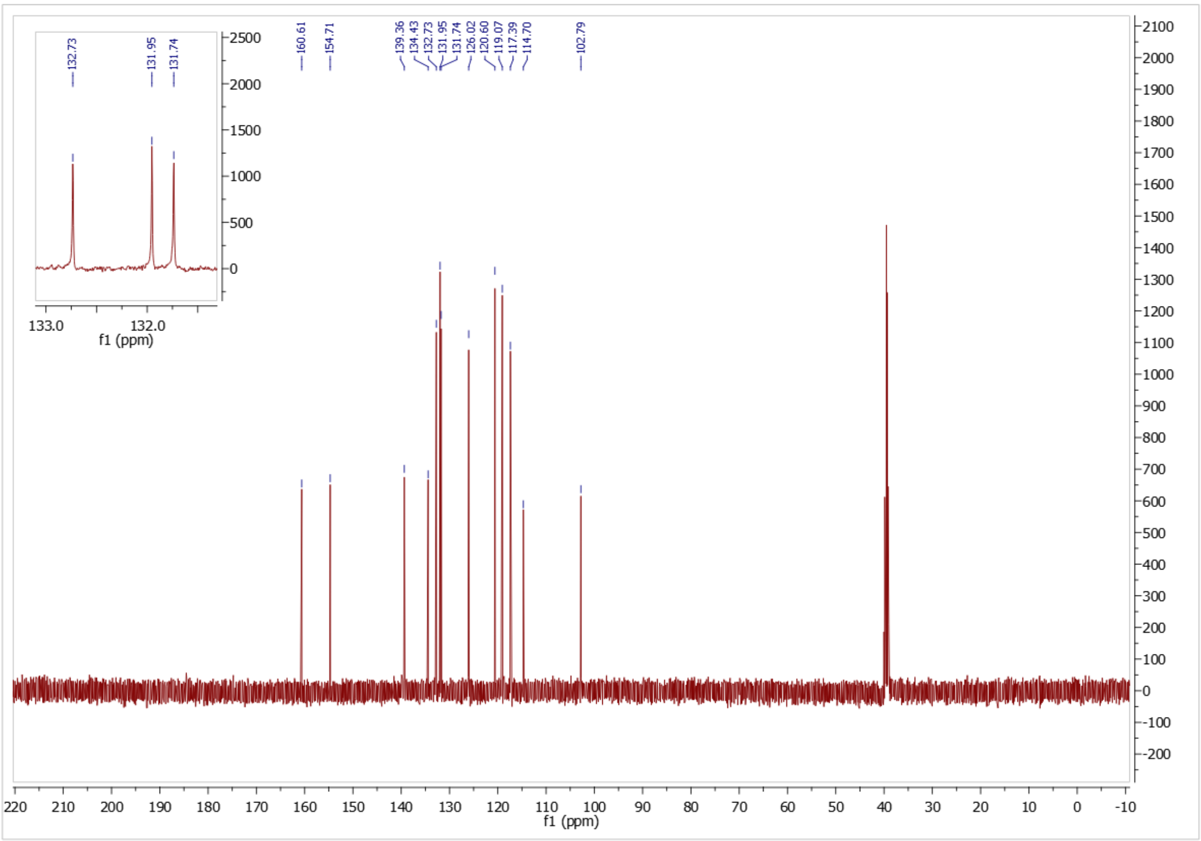
132.73
131.95
131.74
160.61
154.71
139.36
134.43
132.73
131.95
131.74
126.02
120.60
119.07
117.39
114.70
102.79
f1 (ppm)

3-(Aminomethyl)-4-(4-sulfamoylphenoxy)benzene-1-sulfonamide (38)

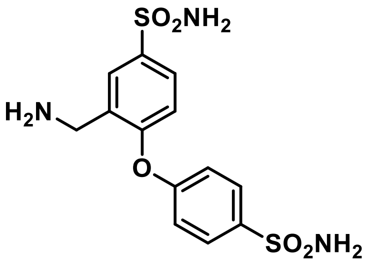

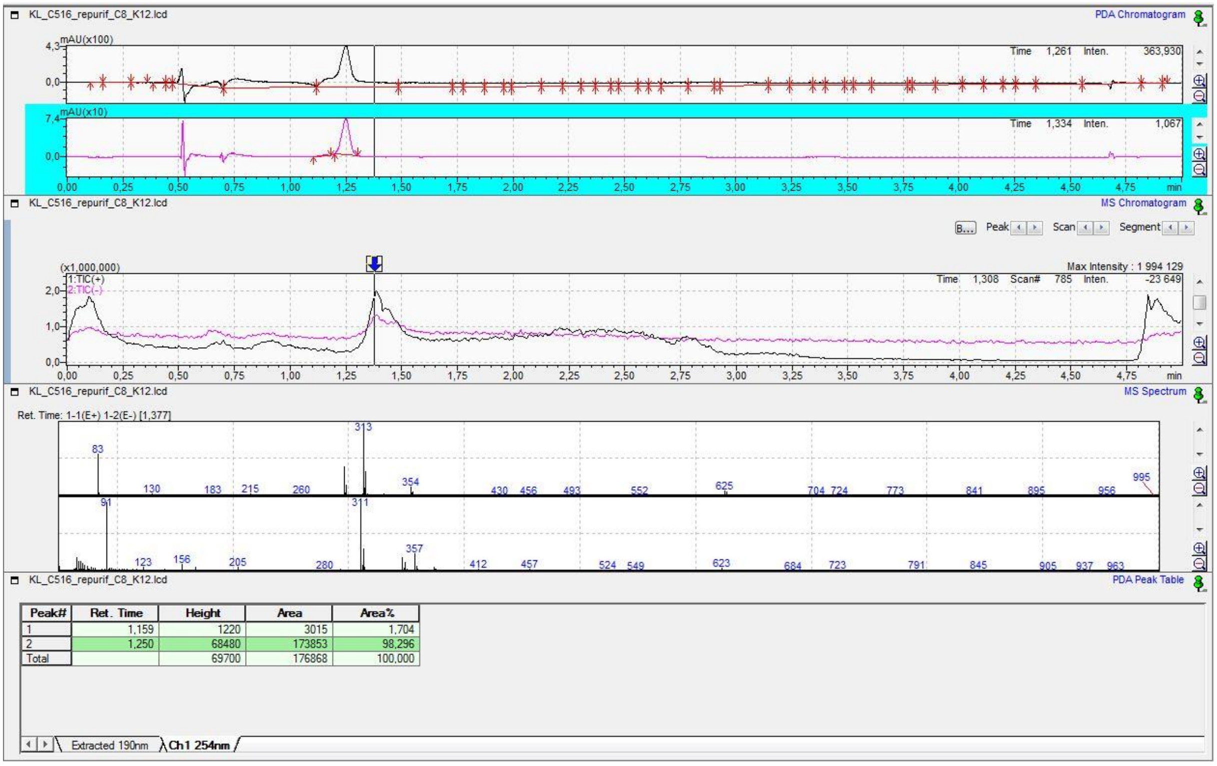

Peak#	Ret. Time	Height	Area	Area%
1	1,159	1220	3015	1,704
2	1,250	68480	173853	98,296
Total		69700	176868	100,000

3-(Aminomethyl)-4-(3-chlorophenoxy)benzene-1-sulfonamide (39)

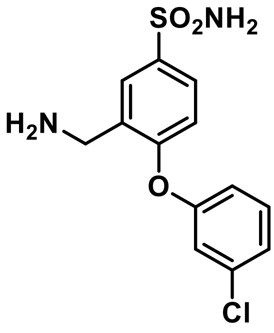

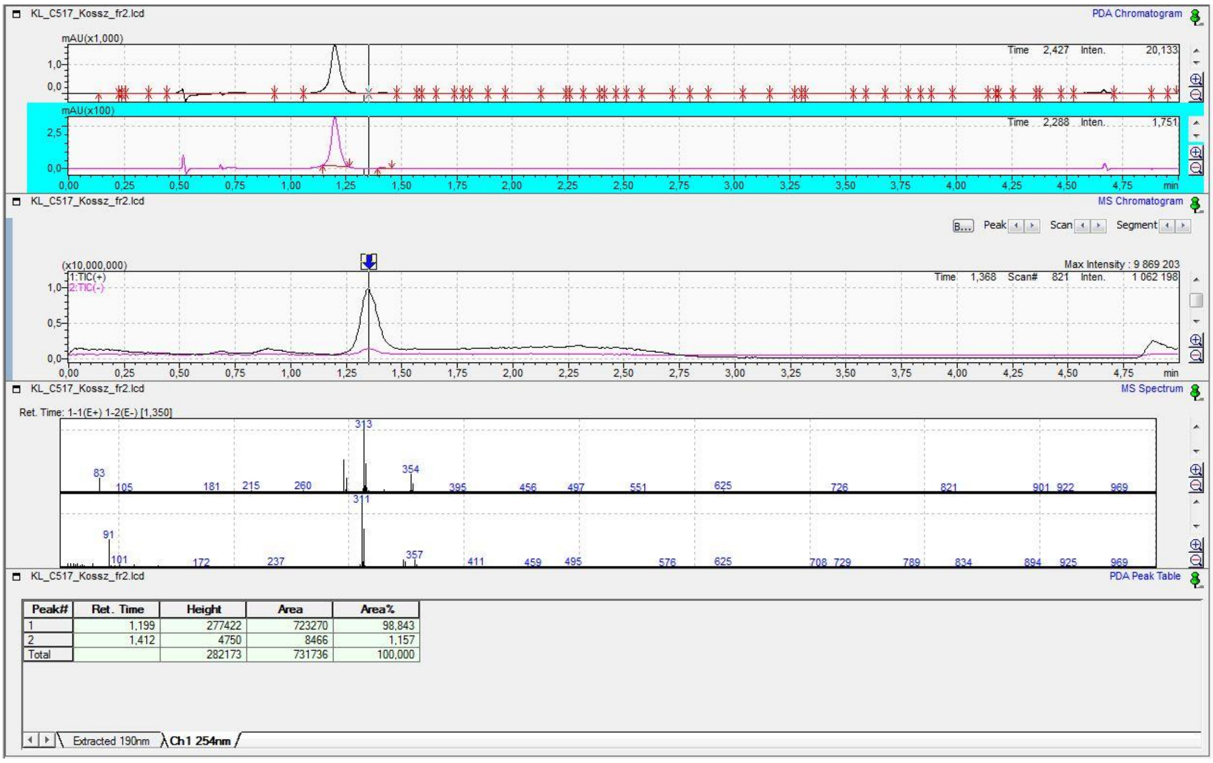

Peak#	Ret. Time	Height	Area	Area%
1	1,199	277422	723270	98,843
2	1,412	4750	8466	1,157
Total		282173	731736	100,000

2-Chloro-*N*-{[5-sulfamoyl-2-(4-sulfamoylphenoxy)phenyl]methyl}pyrimidine-4-carboxamide (40)

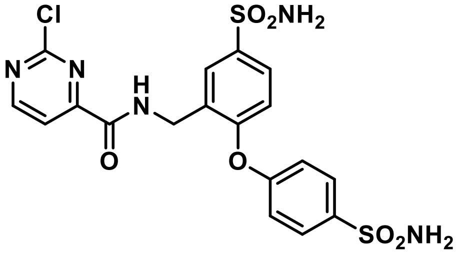

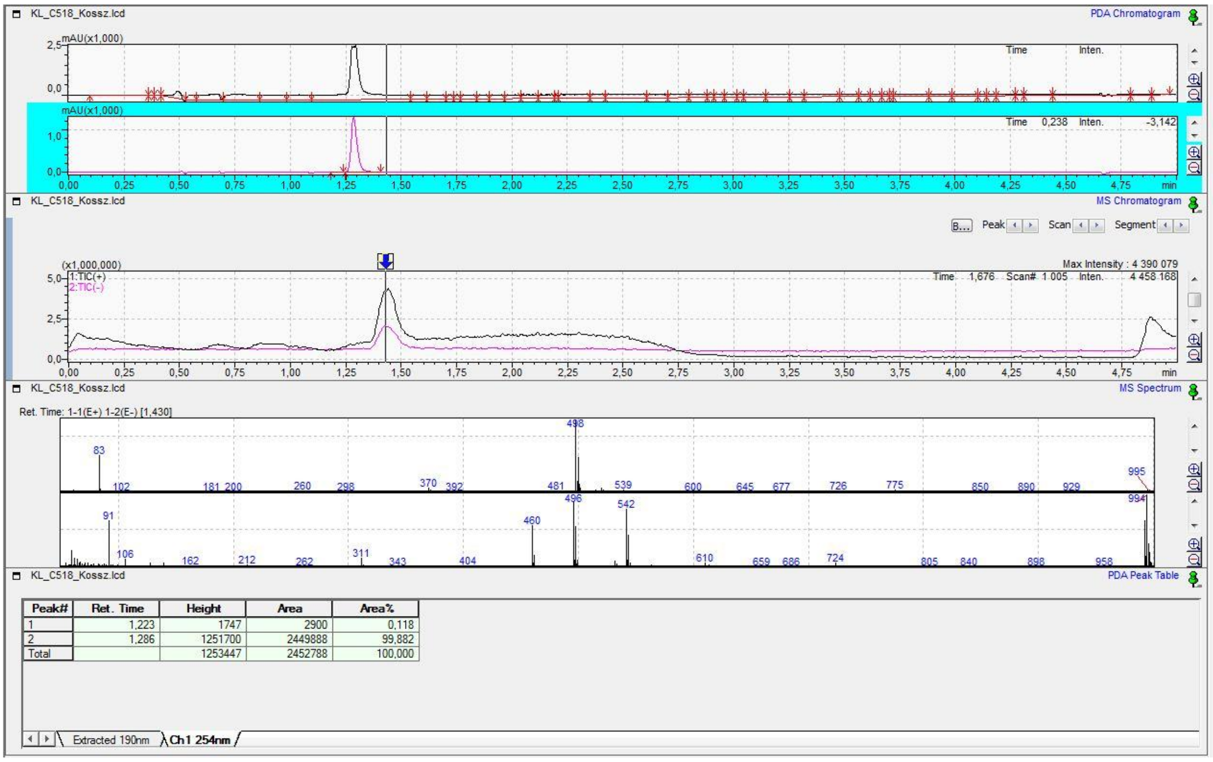
KL_C518_Kossz.lcd
PDA Chromatogram
mAU(x1,000)
Time
Inten.
Time 0,238 Inten. -3,142
min
MS Chromatogram
Max Intensity : 4 390 079
Time 1,676 Scan# 1 005 Inten. 4 458 168
(x1,000,000)
1:TIC(+)
2:TIC(-)
MS Spectrum
Ret. Time: 1-1(E+) 1-2(E-) [1,430]
498
83
995
994
496
542
460
91
PDA Peak Table
Peak# | Ret. Time | Height | Area | Area%
1 | 1,223 | 1747 | 2900 | 0,118
2 | 1,286 | 1251700 | 2449888 | 99,882
Total | | 1253447 | 2452788 | 100,000
Extracted 190nm
Ch1 254nm

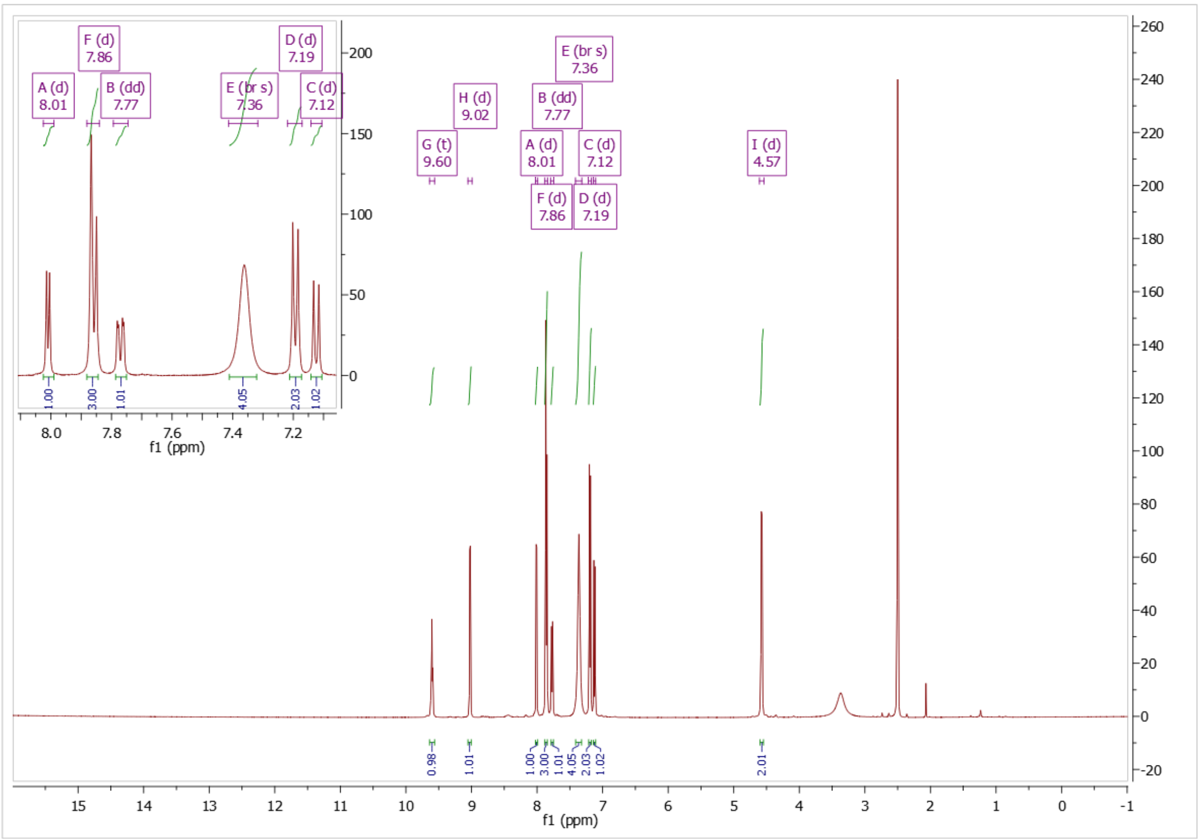
A (d) 8.01
F (d) 7.86
B (dd) 7.77
E (br s) 7.36
D (d) 7.19
C (d) 7.12
G (t) 9.60
H (d) 9.02
I (d) 4.57
f1 (ppm)

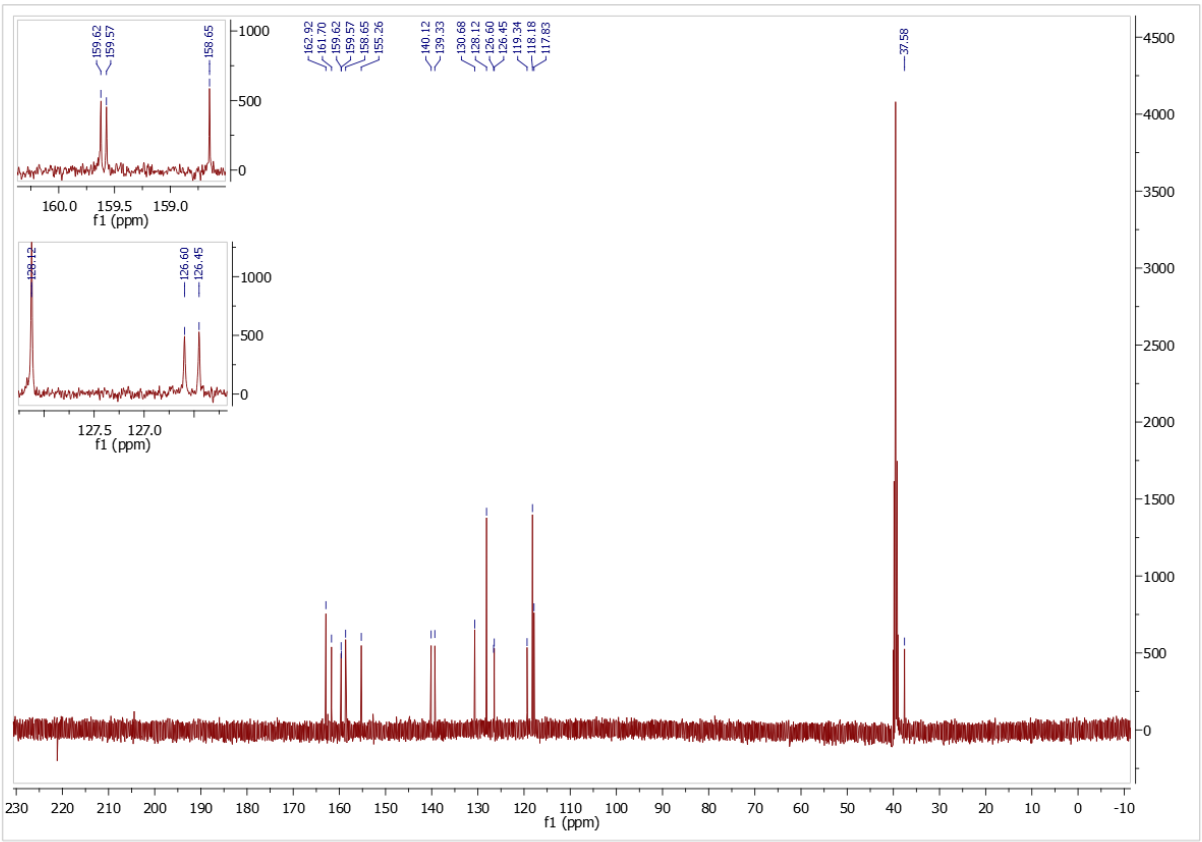

2-Chloro-*N*-{[2-(3-chlorophenoxy)-5-sulfamoylphenyl]methyl}pyrimidine-4-carboxamide (41)

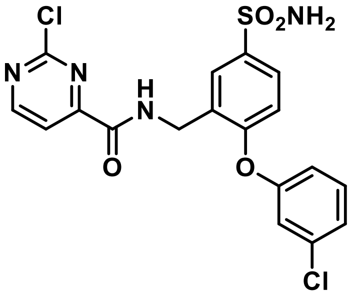

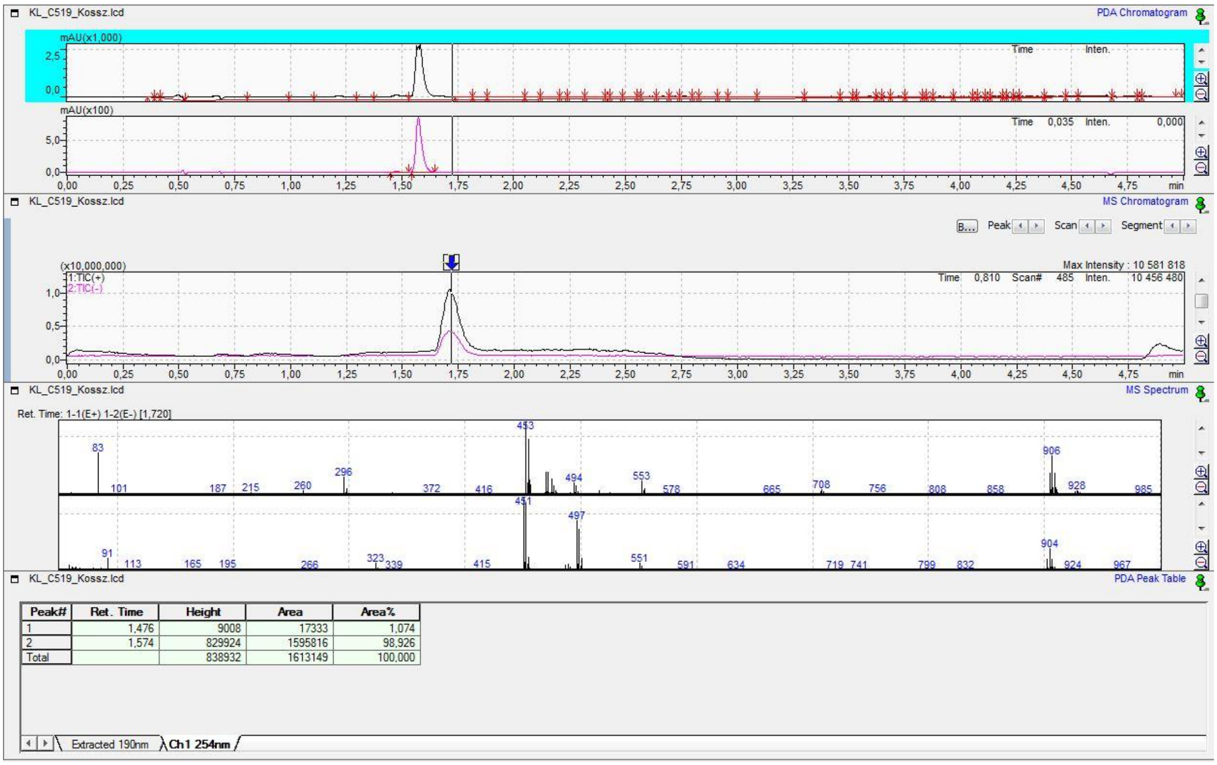

Peak#	Ret. Time	Height	Area	Area%
1	1,476	9008	17333	1,074
2	1,574	829924	1595816	98,926
Total		838932	1613149	100,000

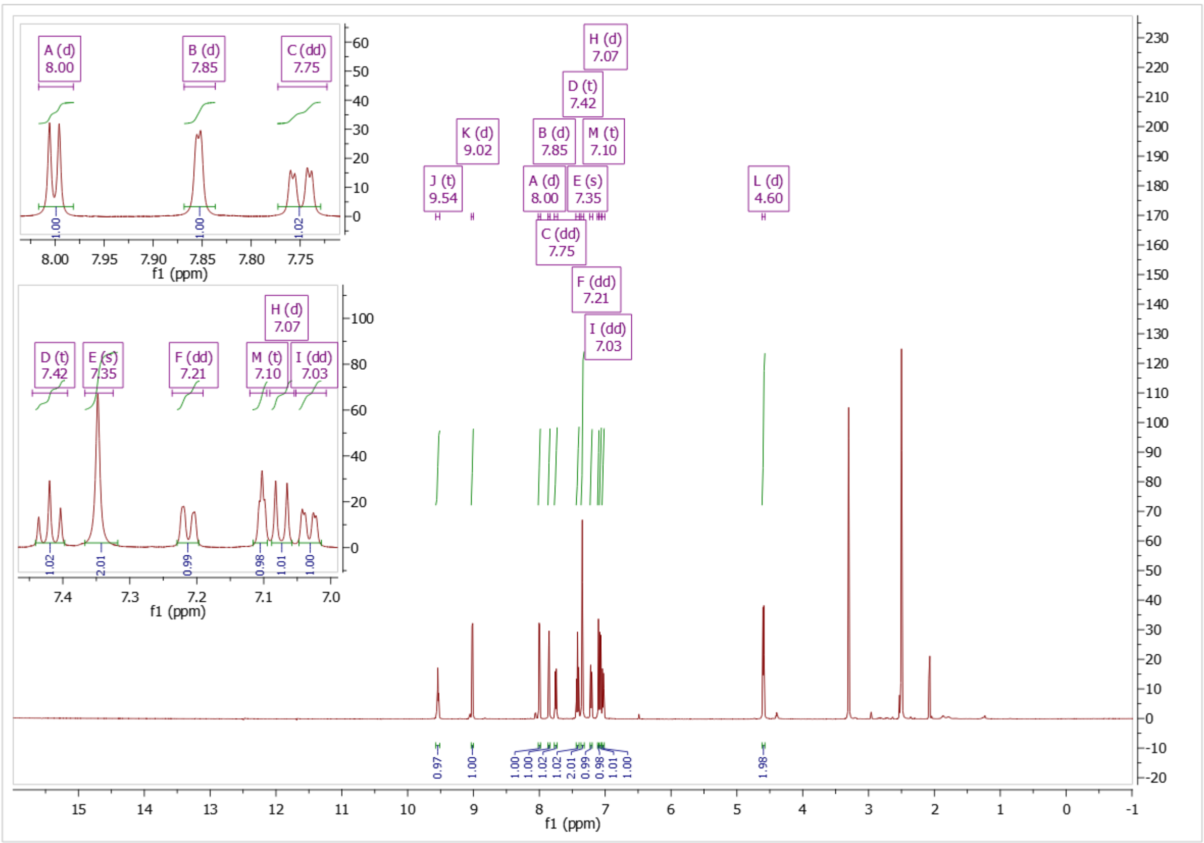

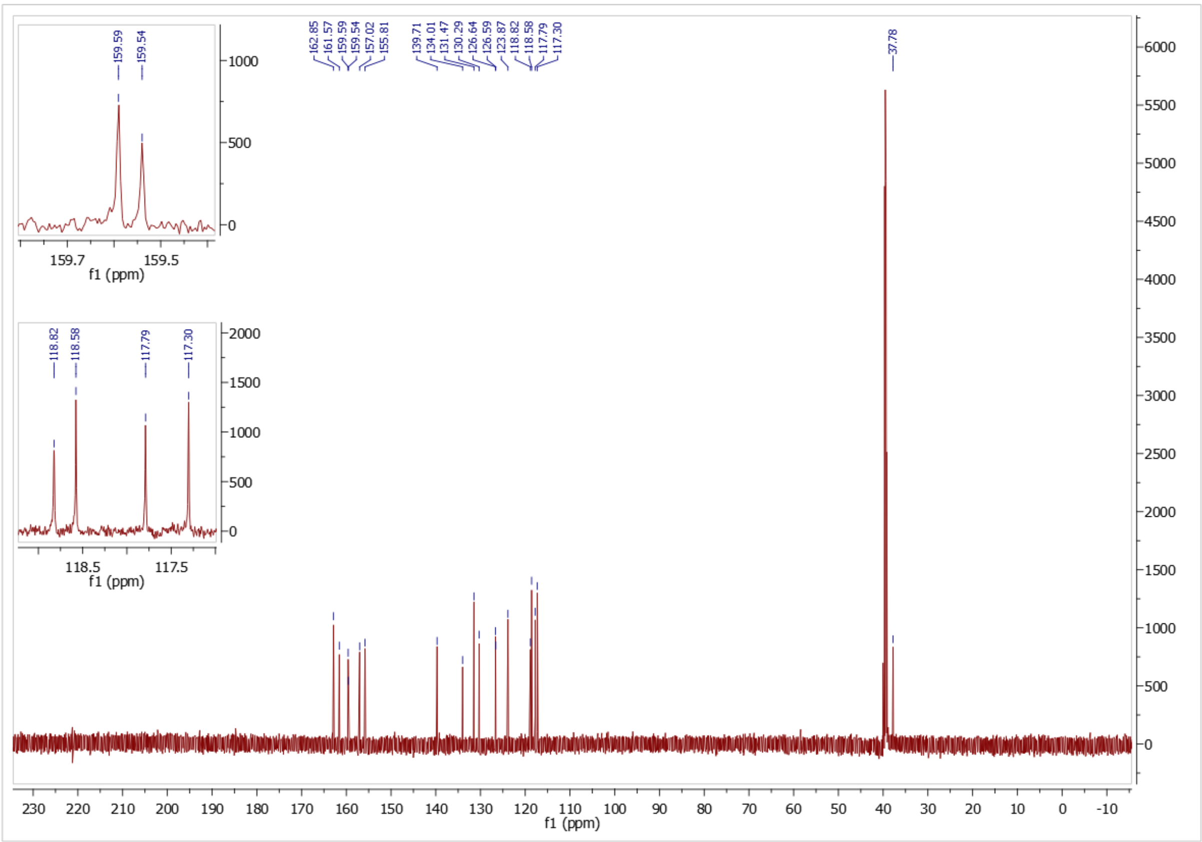

N-{[2-bromo-5-(phenylsulfamoyl)phenyl]methyl}-2-chloropyrimidine-4-carboxamide (42)

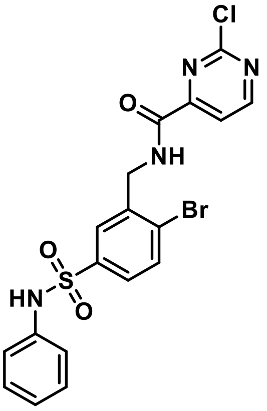

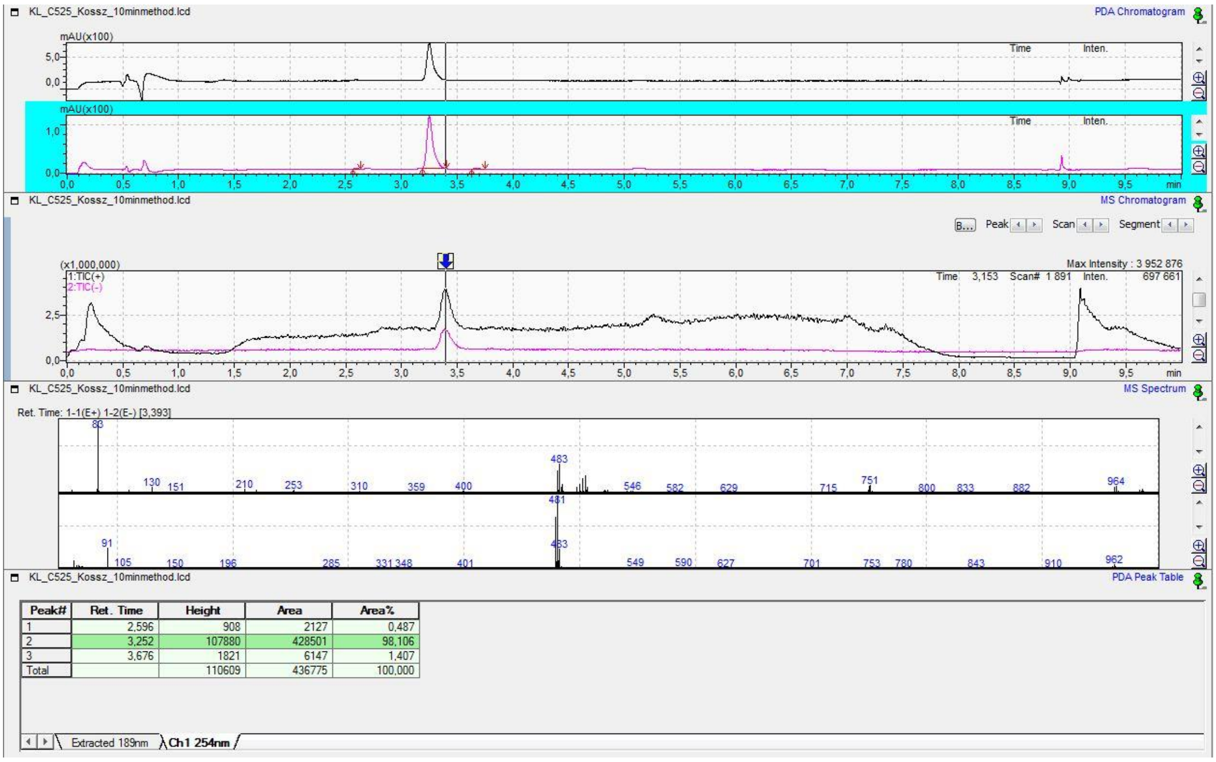
KL_C525_Kossz_10minmethod.lcd
PDA Chromatogram
MS Chromatogram
MS Spectrum
PDA Peak Table
Max Intensity : 3 952 876
Time 3,153 Scan# 1 891 Inten. 697 661
Ret. Time: 1-1(E+) 1-2(E-) [3,393]
Peak# Ret. Time Height Area Area%
1 2,596 908 2127 0,487
2 3,252 107880 428501 98,106
3 3,676 1821 6147 1,407
Total 110609 436775 100,000
Extracted 189nm
Ch1 254nm

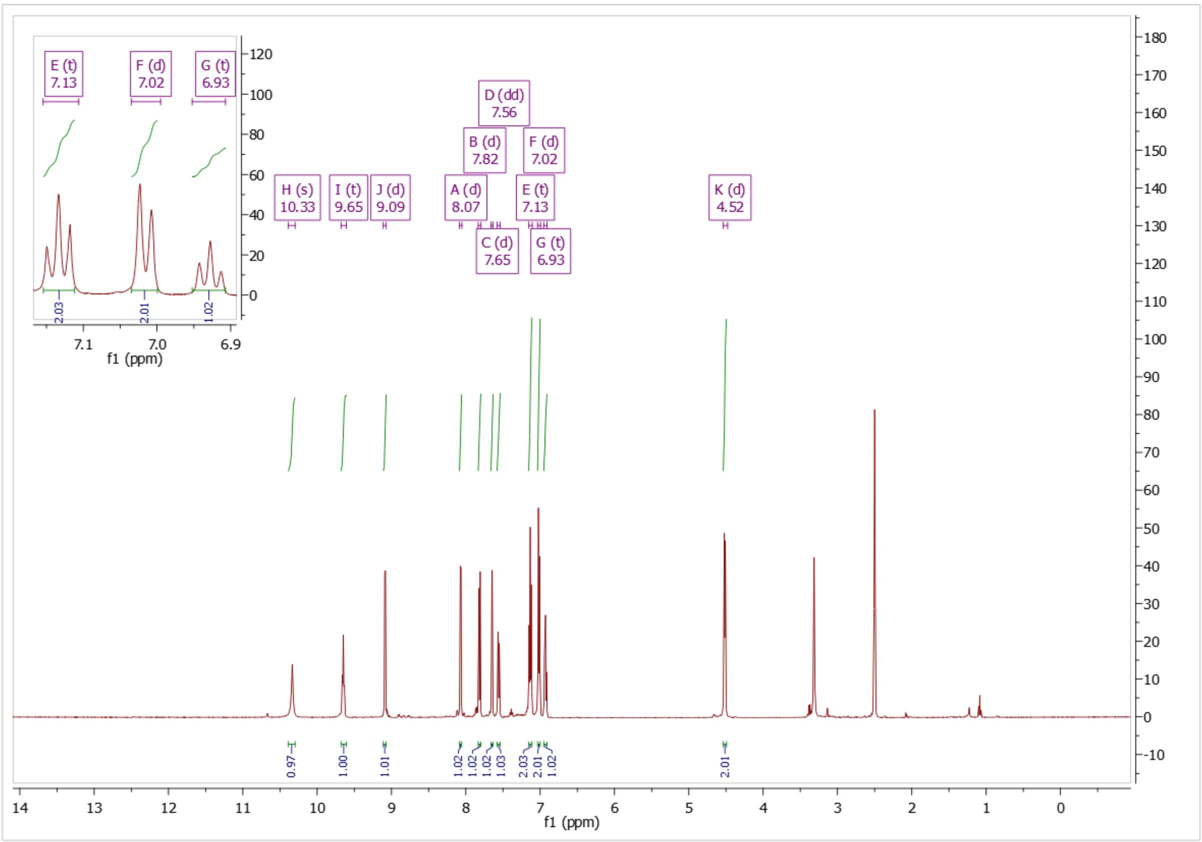
H (s) 10.33
I (t) 9.65
J (d) 9.09
A (d) 8.07
B (d) 7.82
C (d) 7.65
D (dd) 7.56
E (t) 7.13
F (d) 7.02
G (t) 6.93
K (d) 4.52
f1 (ppm)

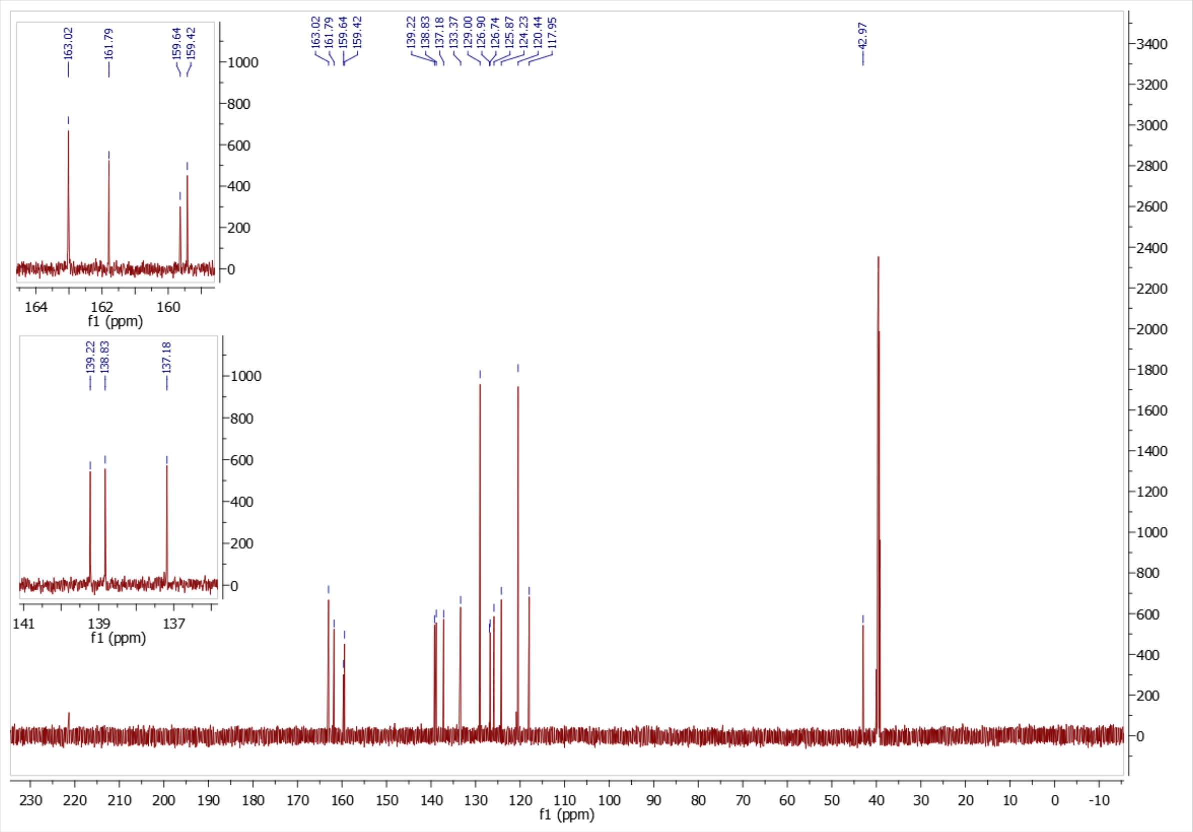

4-Bromo-*N*-phenylbenzene-1-sulfonamide (43)

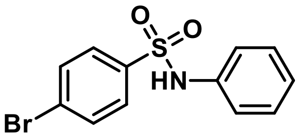

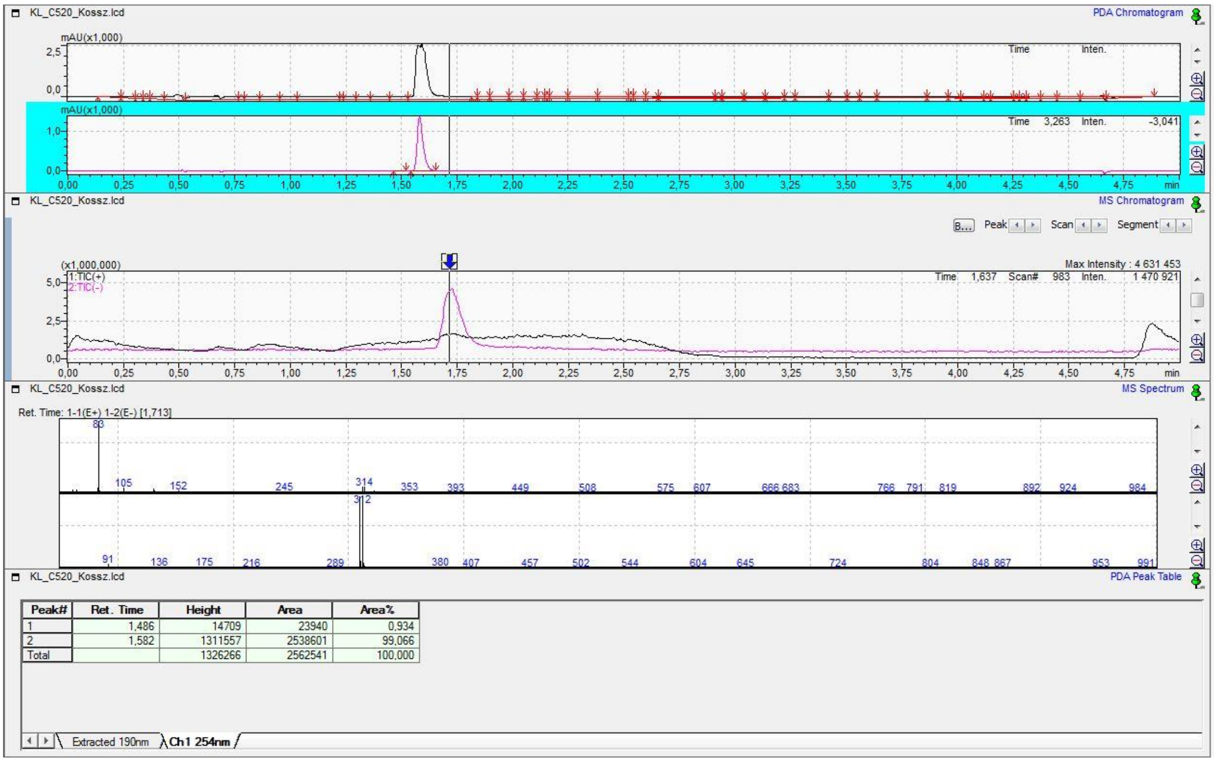

Peak#	Ret. Time	Height	Area	Area%
1	1,486	14709	23940	0,934
2	1,582	1311557	2538601	99,066
Total		1326266	2562541	100,000

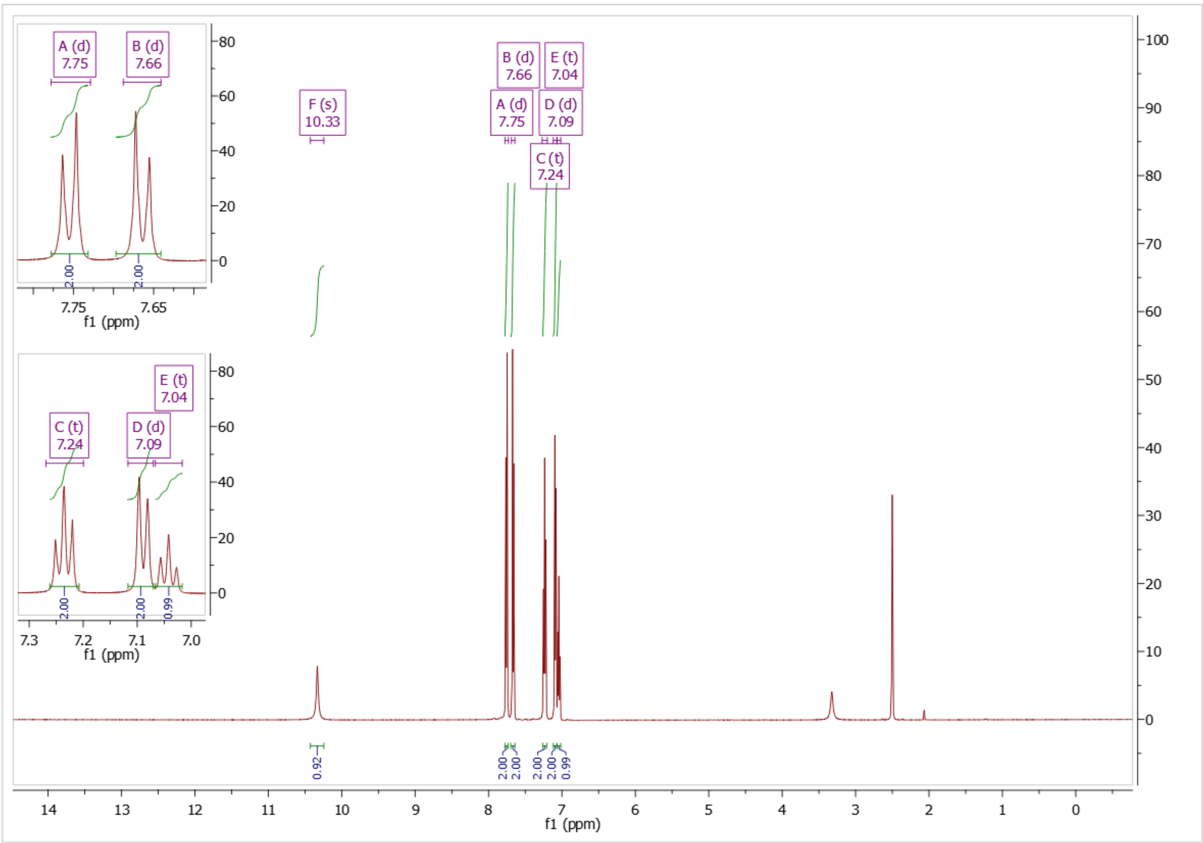

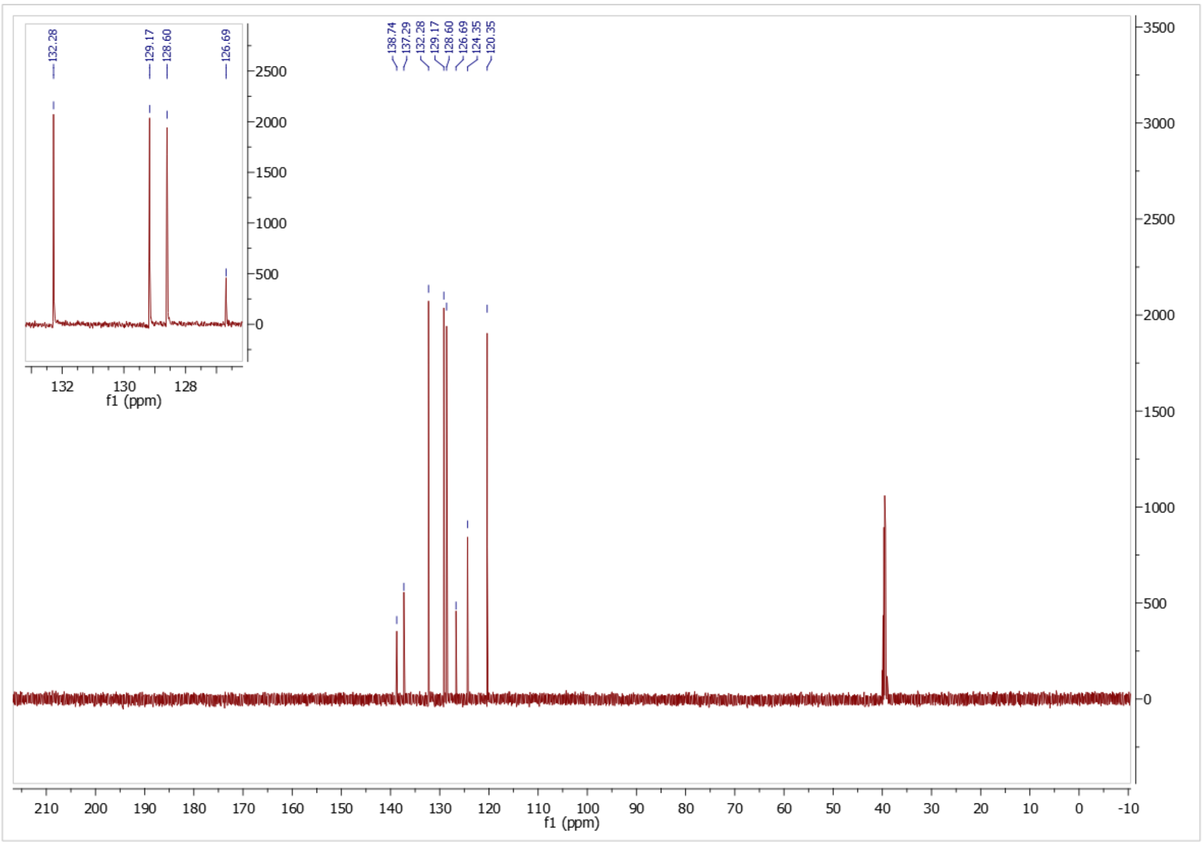

4-Bromo-3-fluoro-*N*,*N*-bis[(4-methoxyphenyl)methyl]benzene-1-sulfonamide (45)

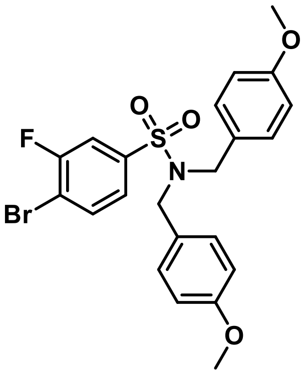

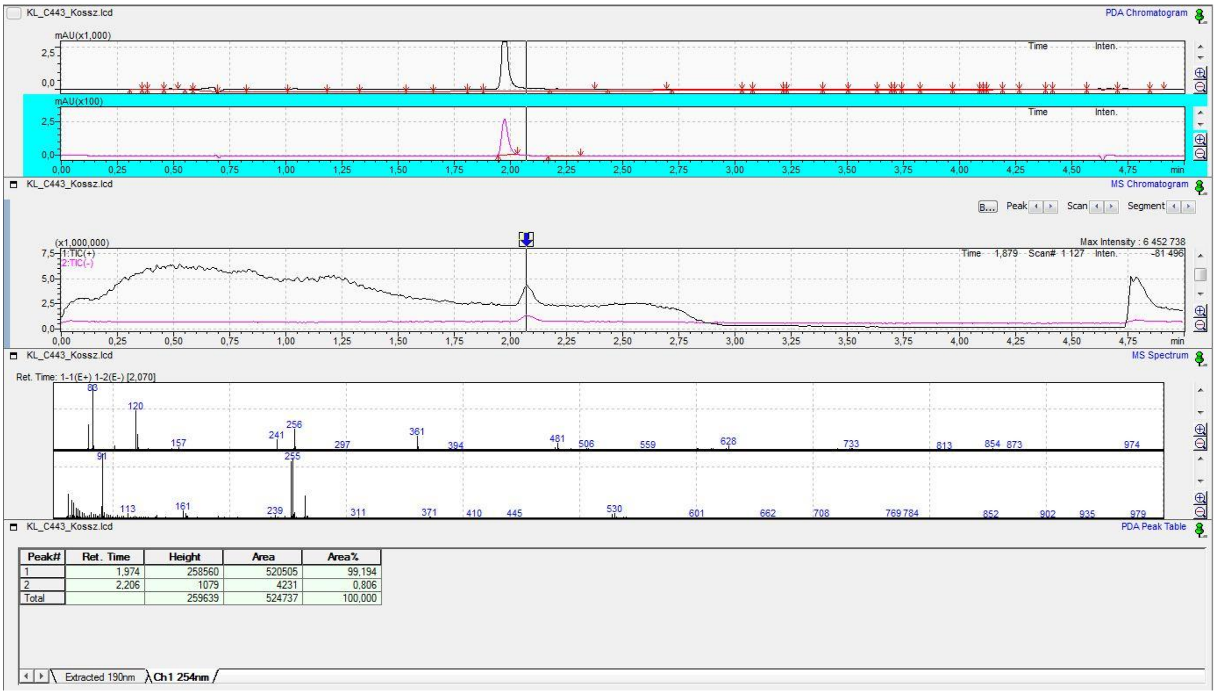

Peak#	Ret. Time	Height	Area	Area%
1	1,974	258560	520505	99,194
2	2,206	1079	4231	0,806
Total		259639	524737	100,000

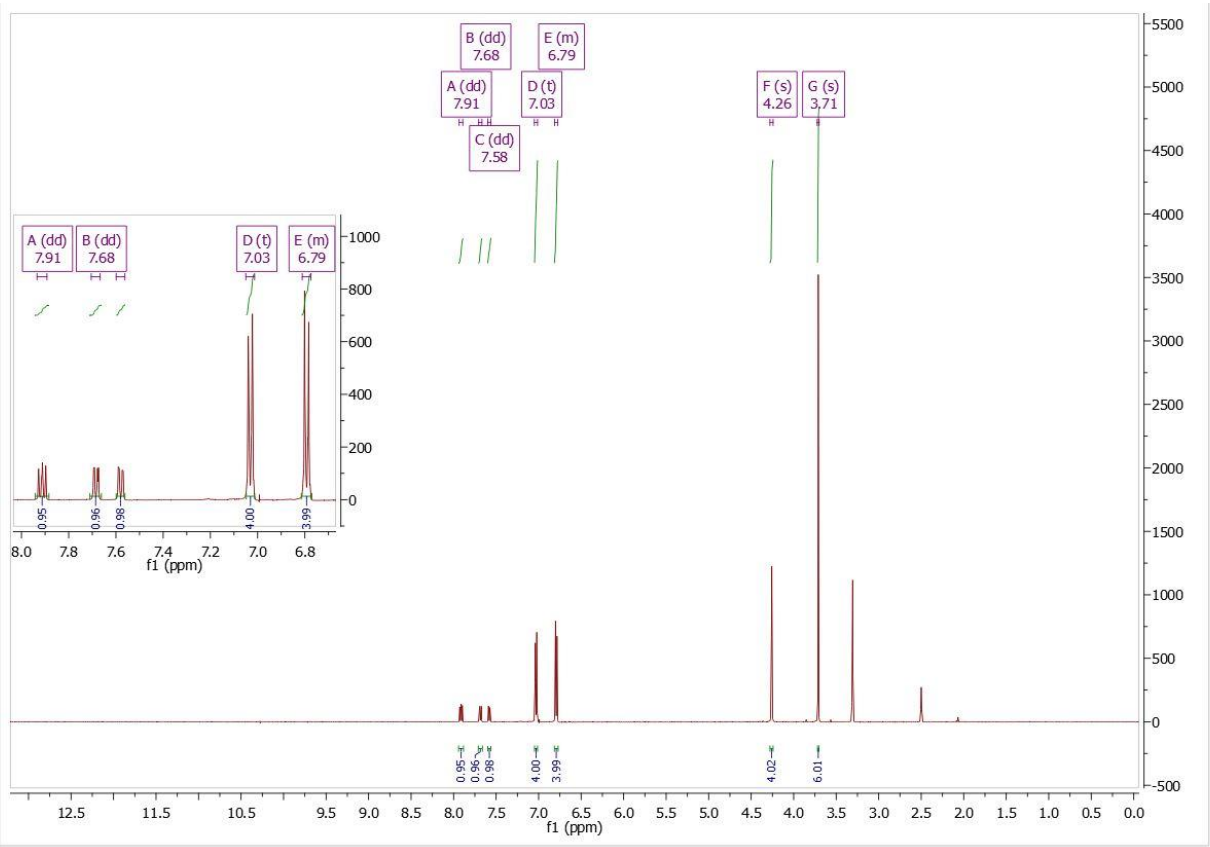

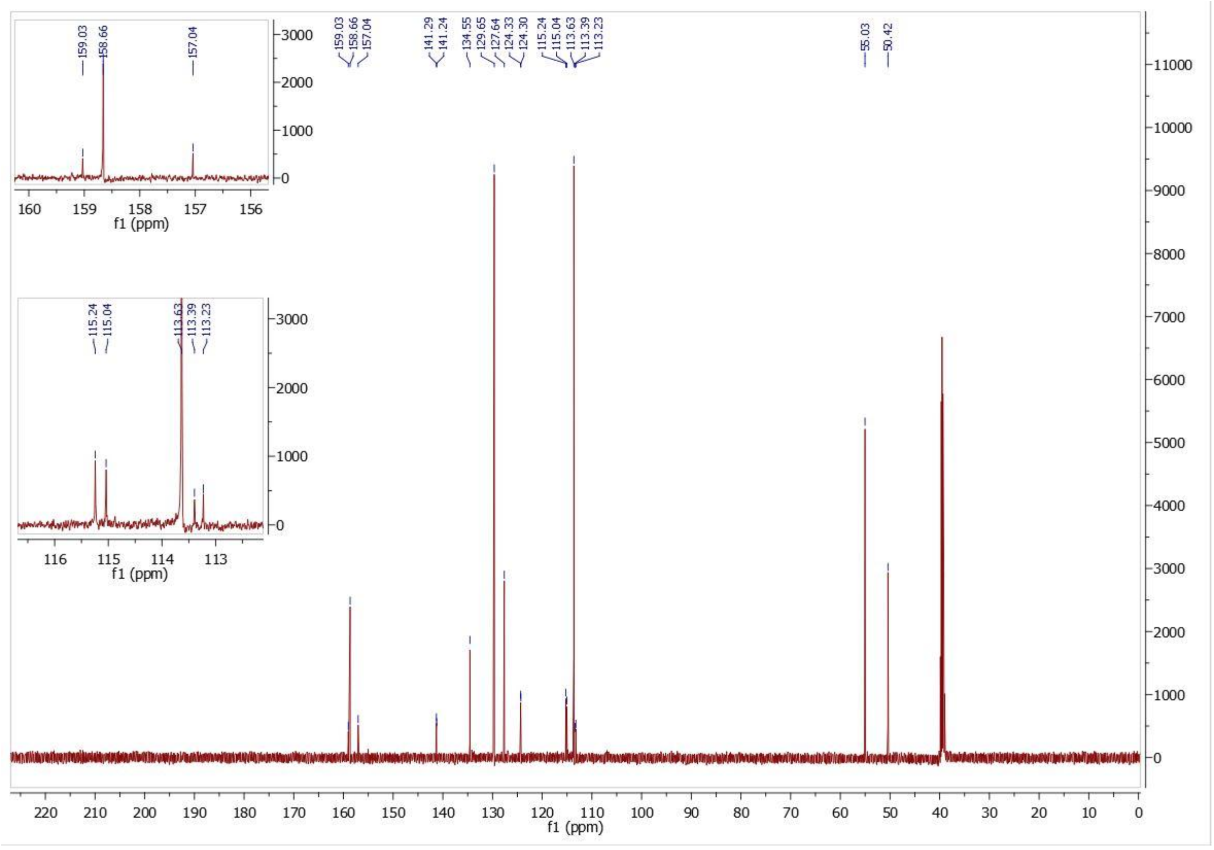

1,3-Diethyl 2-(5-[bis[(4-methoxyphenyl)methyl]sulfamoyl]-2-bromophenyl)propanedioate (48)

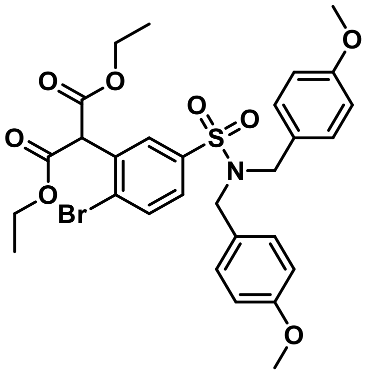

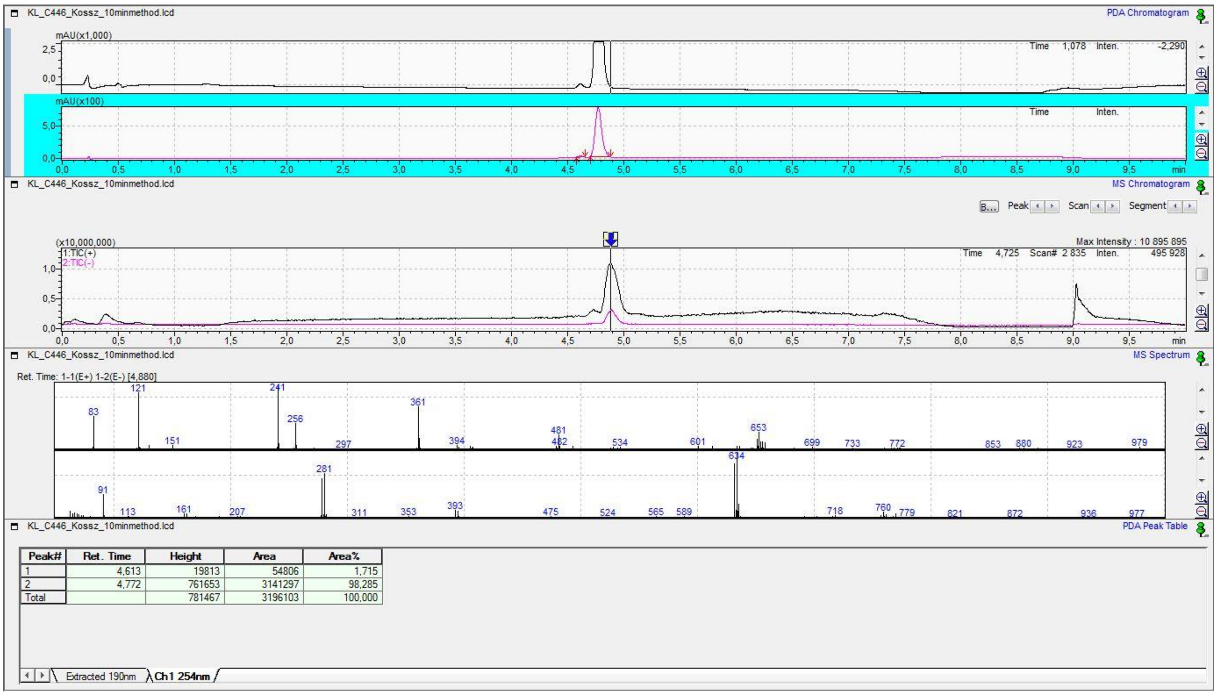

Peak#	Ret. Time	Height	Area	Area%
1	4,613	19813	54806	1,715
2	4,772	761653	3141297	98,285
Total		781467	3196103	100,000

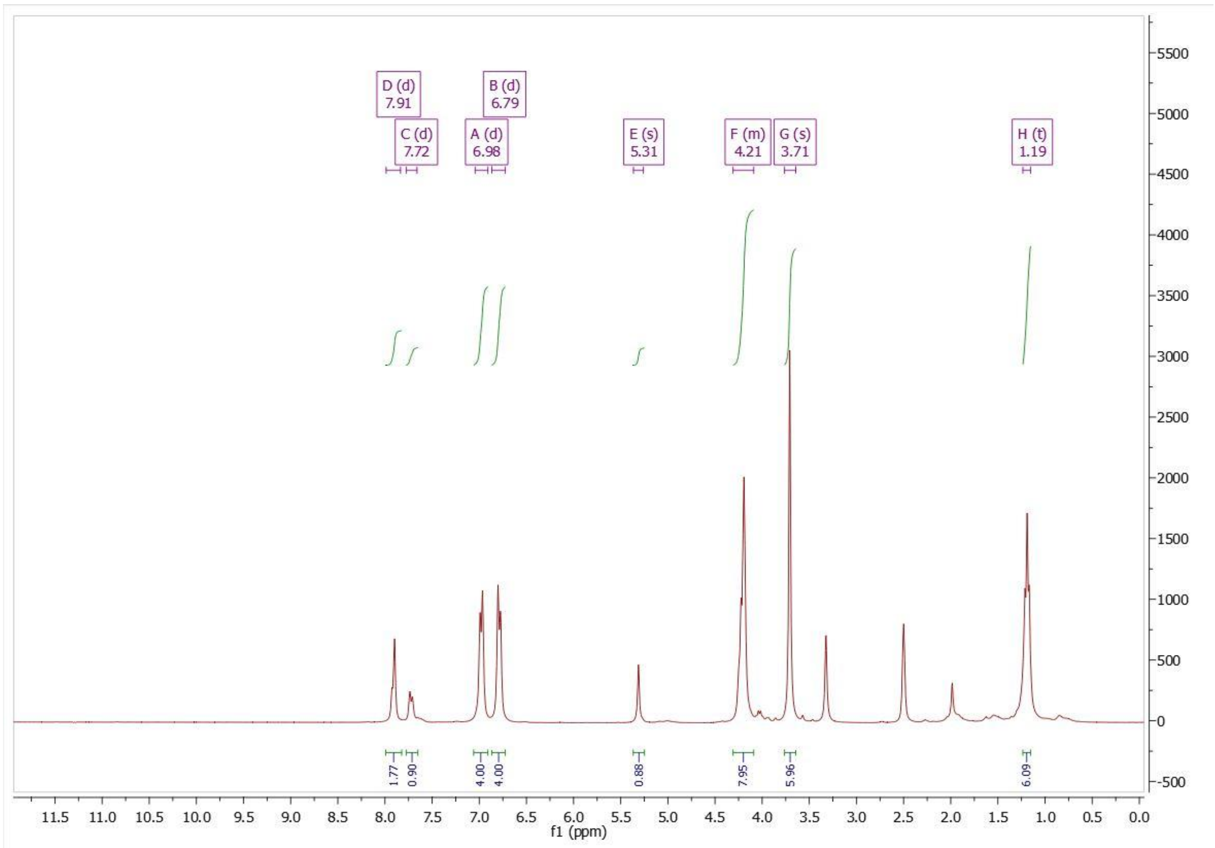

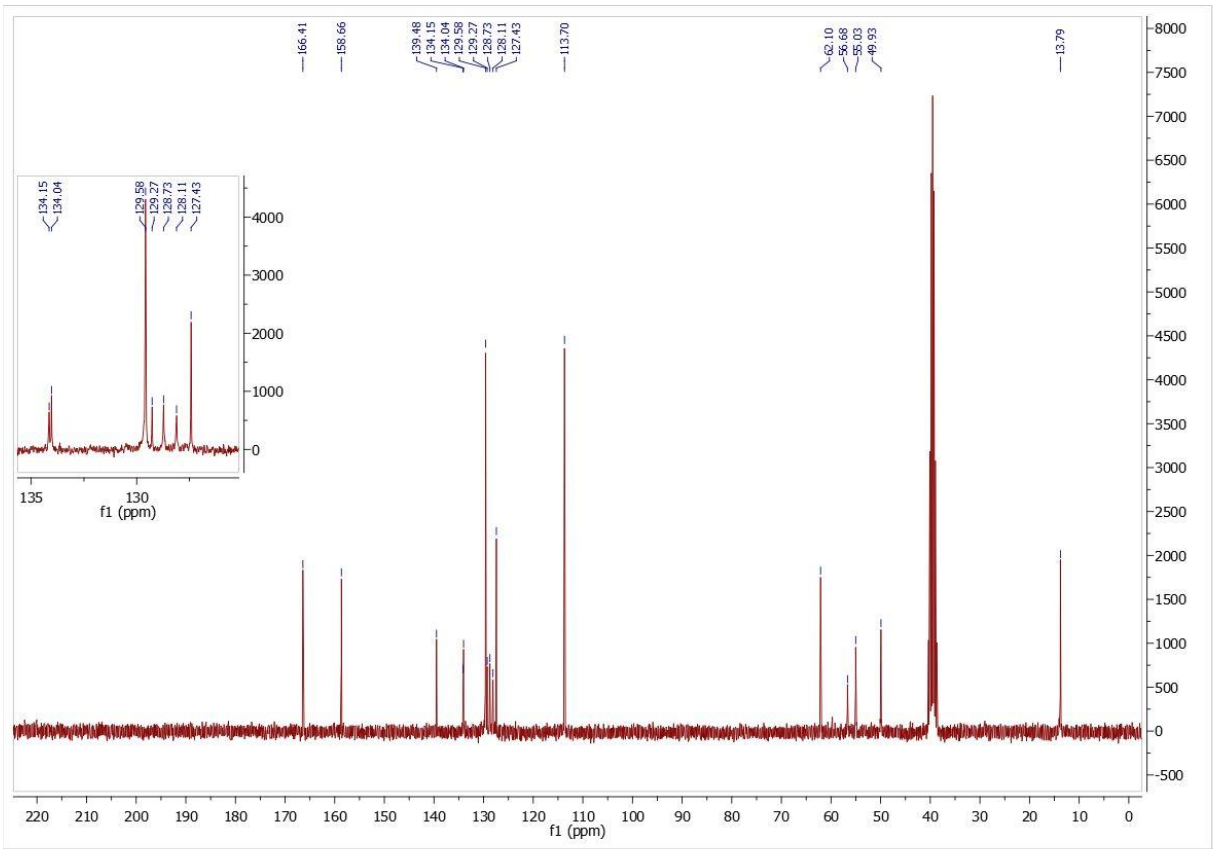

166.41
158.66
139.48
134.15
134.04
129.58
129.27
128.73
128.11
127.43
113.70
62.10
56.68
55.03
49.93
13.79
f1 (ppm)

2-(2-Bromo-5-sulfamoylphenyl)acetic acid (49)

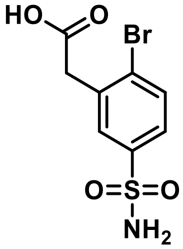

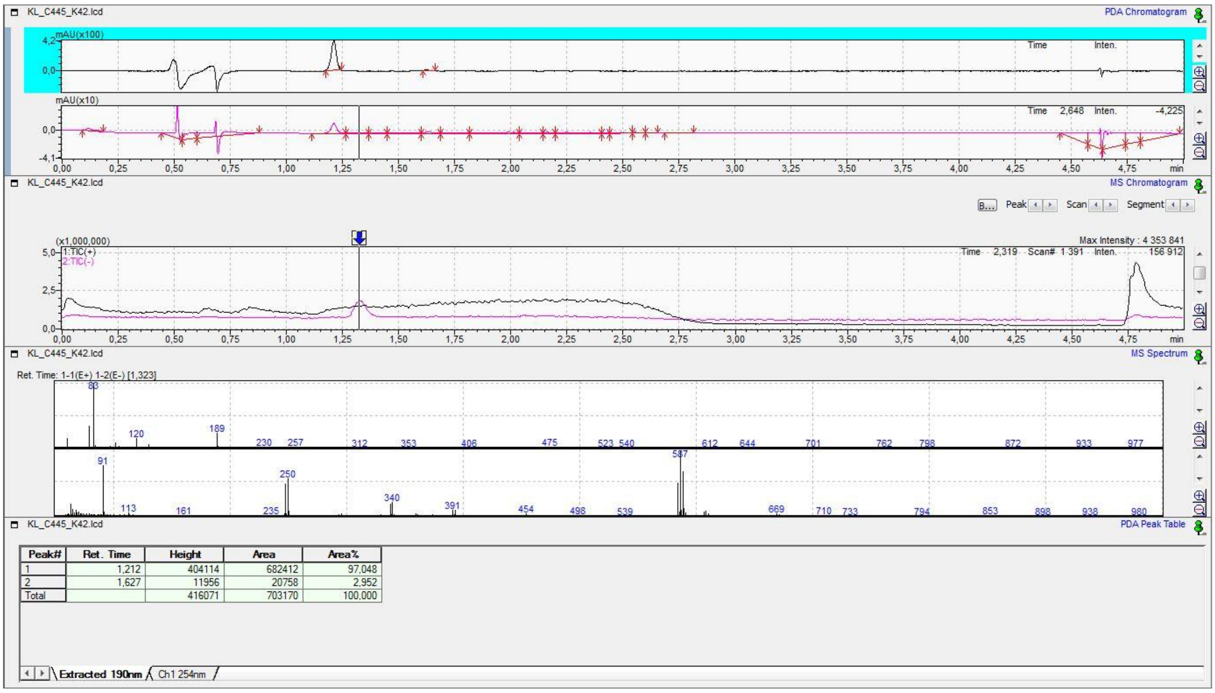

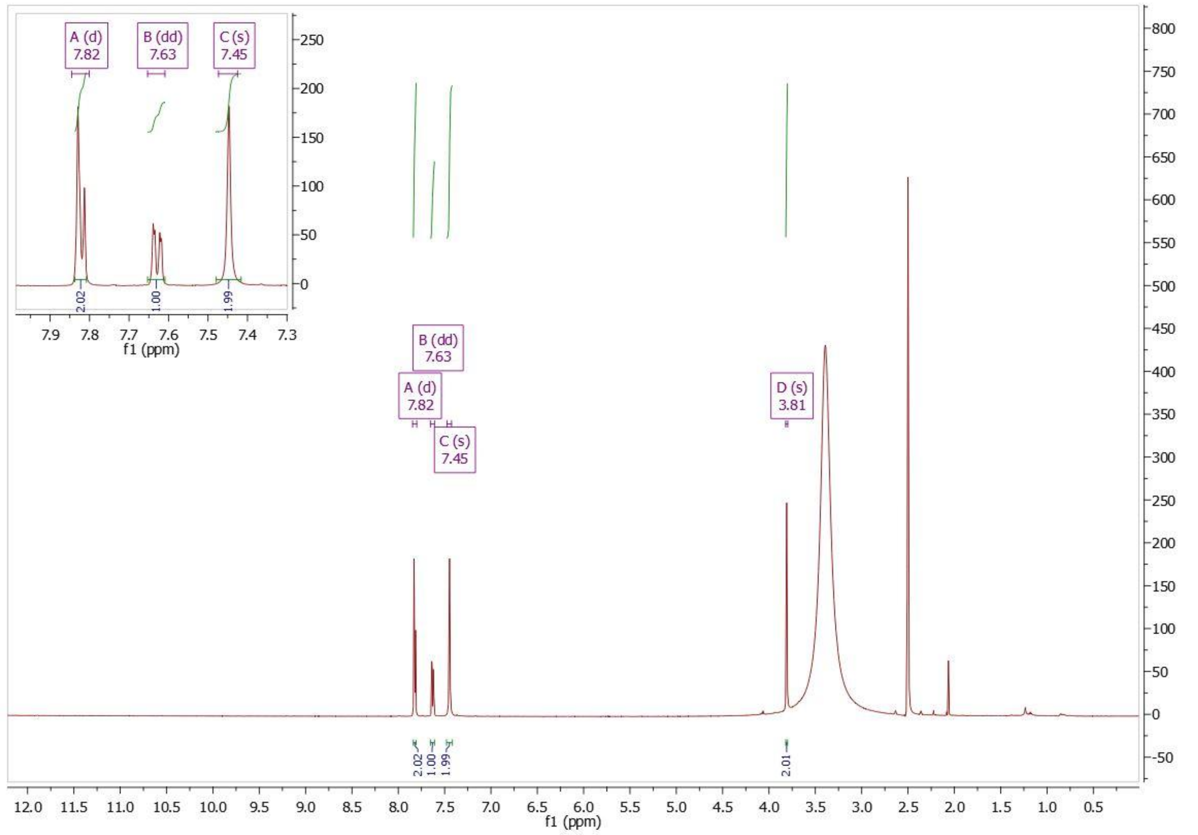
A (d)
7.82
B (dd)
7.63
C (s)
7.45
D (s)
3.81
2.02
1.00
1.99
2.01
f1 (ppm)

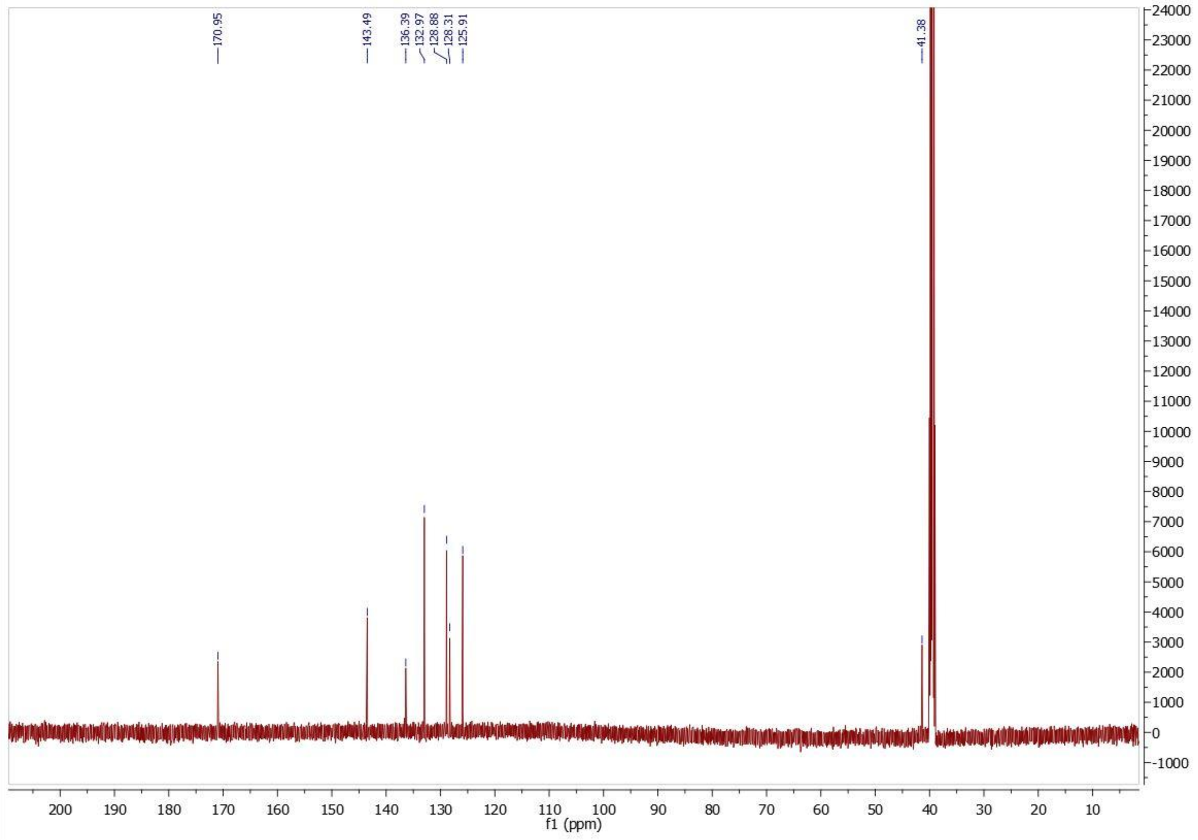
170.95
143.49
136.39
132.97
128.88
128.31
125.91
41.38
f1 (ppm)

2-Cyano-*N*-[(3-sulfamoylphenyl)methyl]pyrimidine-4-carboxamide (51)

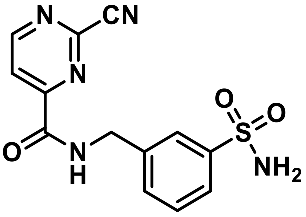

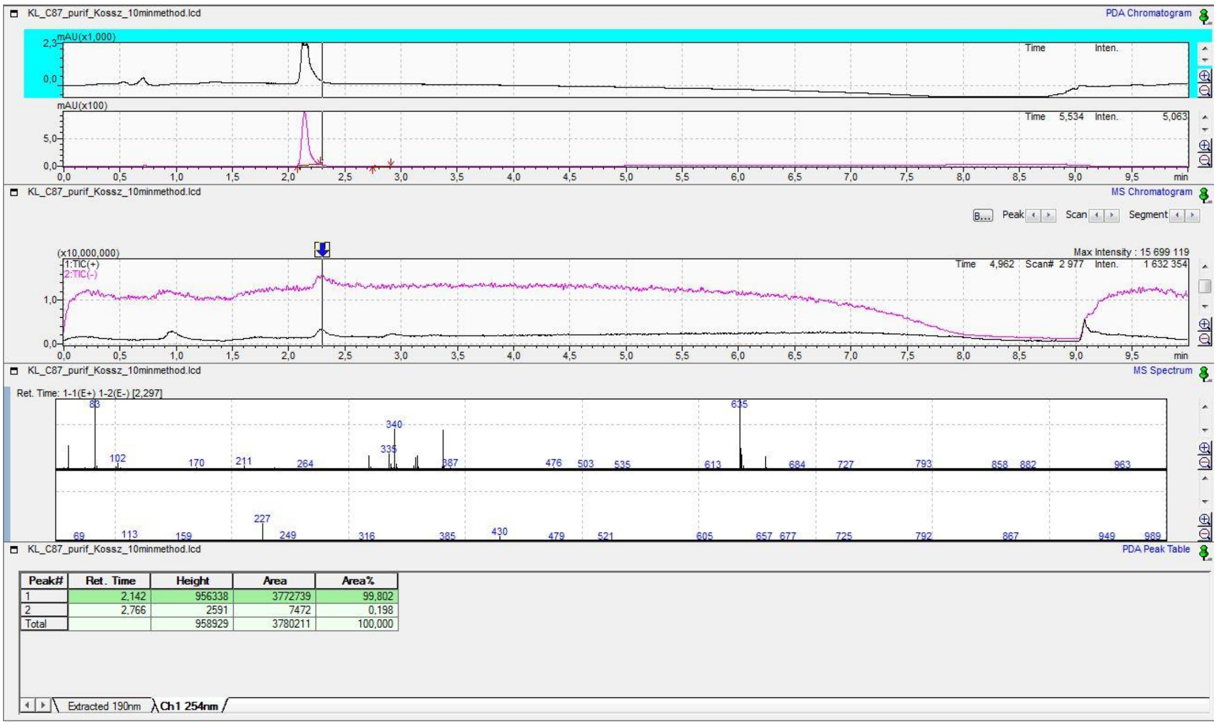

Peak#	Ret. Time	Height	Area	Area%
1	2,142	956338	3772739	99,802
2	2,766	2591	7472	0,198
Total		958929	3780211	100,000

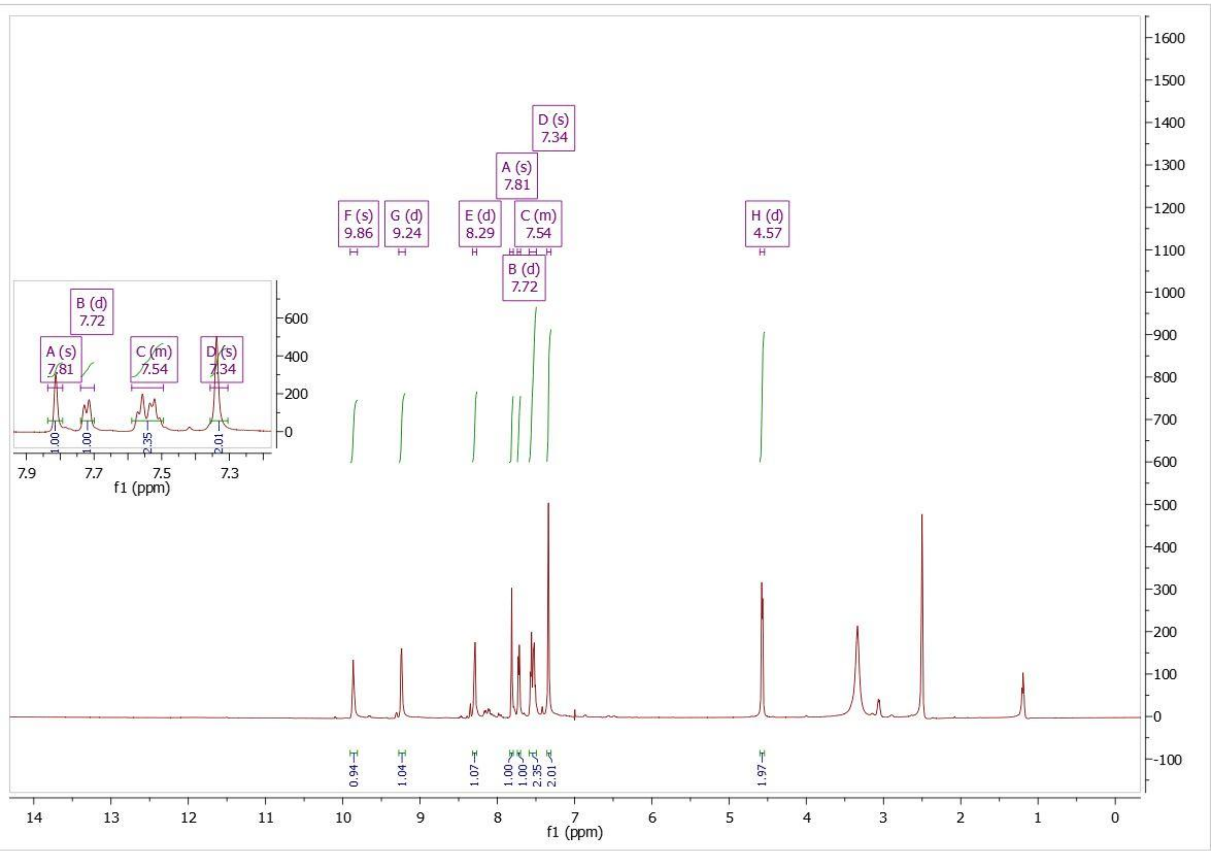
F (s) 9.86
G (d) 9.24
E (d) 8.29
A (s) 7.81
B (d) 7.72
C (m) 7.54
D (s) 7.34
H (d) 4.57
0.94
1.04
1.07
1.00
1.00
2.35
2.01
1.97
f1 (ppm)

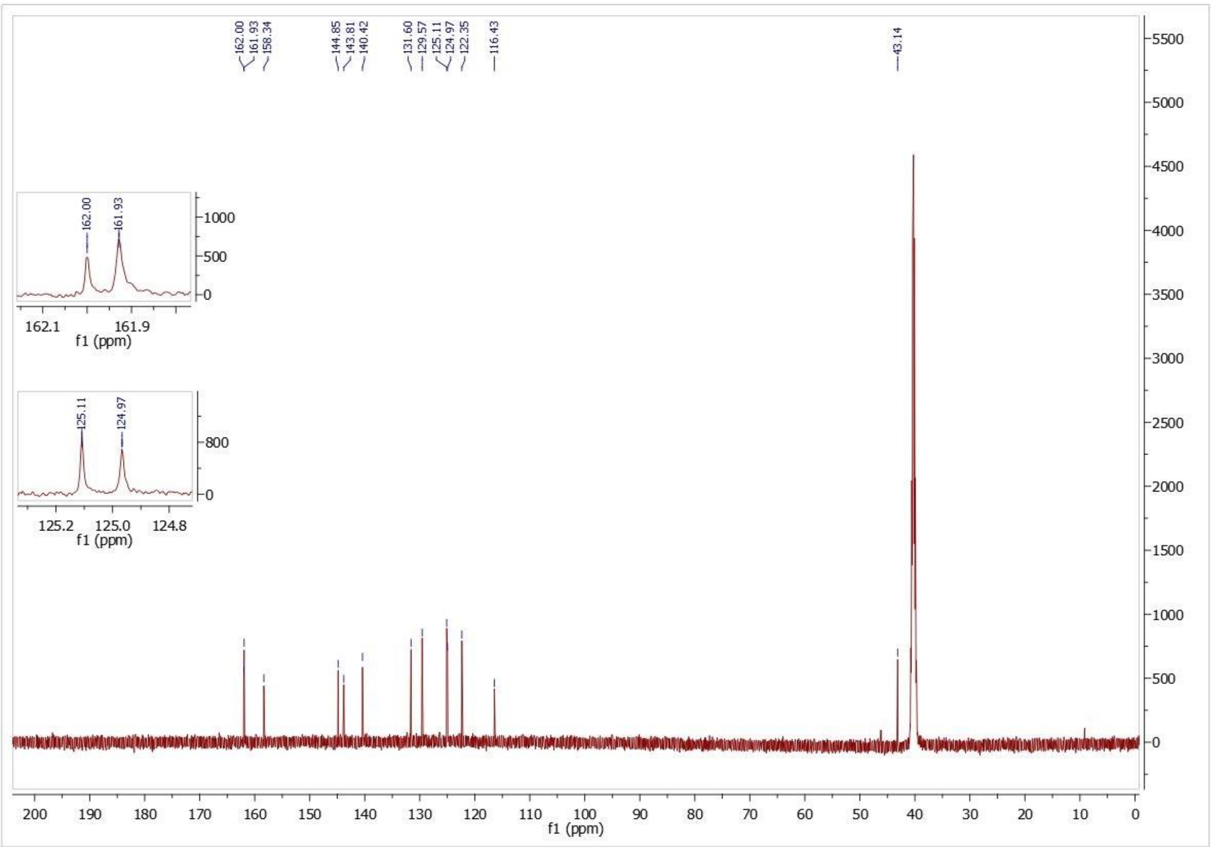
162.00
161.93
158.34
144.85
143.81
140.42
131.60
129.57
125.11
124.97
122.35
116.43
43.14
f1 (ppm)

N-[(2-chloro-5-sulfamoylphenyl)methyl]-2-cyanopyrimidine-4-carboxamide (52)

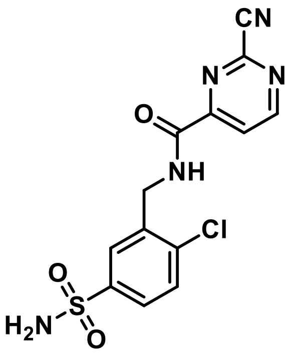

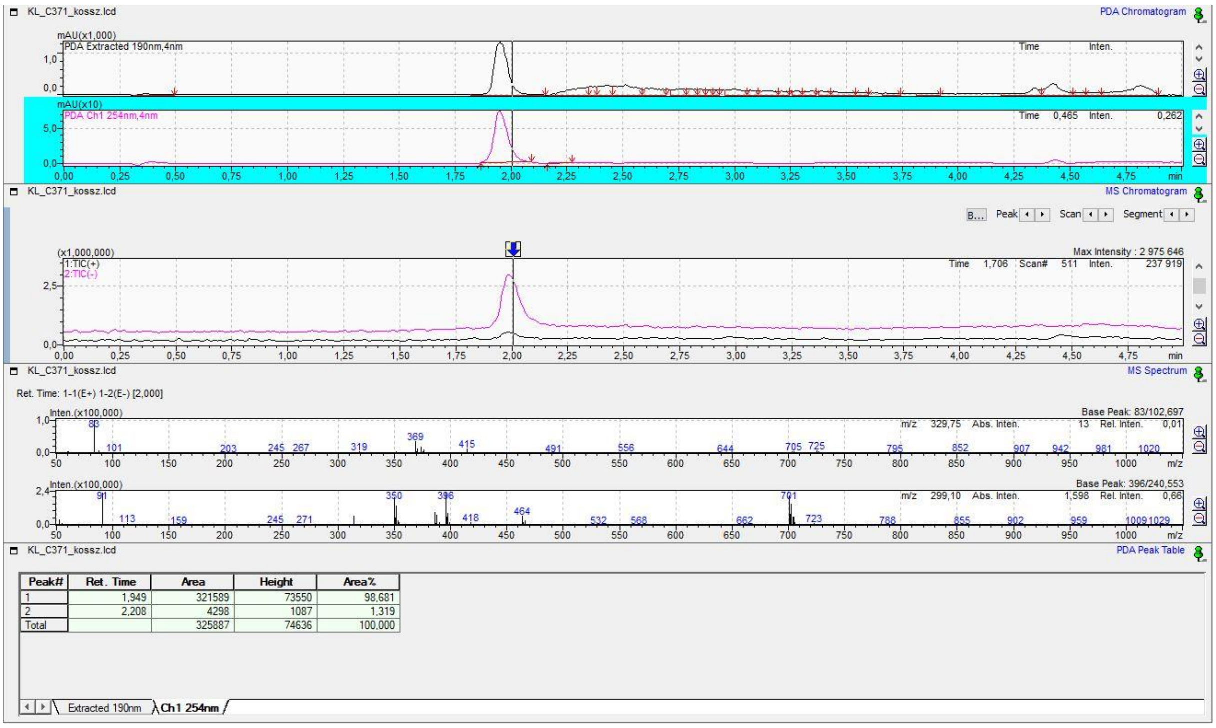

Peak#	Ret. Time	Area	Height	Area%
1	1,949	321589	73550	98,681
2	2,208	4298	1087	1,319
Total		325887	74636	100,000

Extracted 190nm | Ch1 254nm

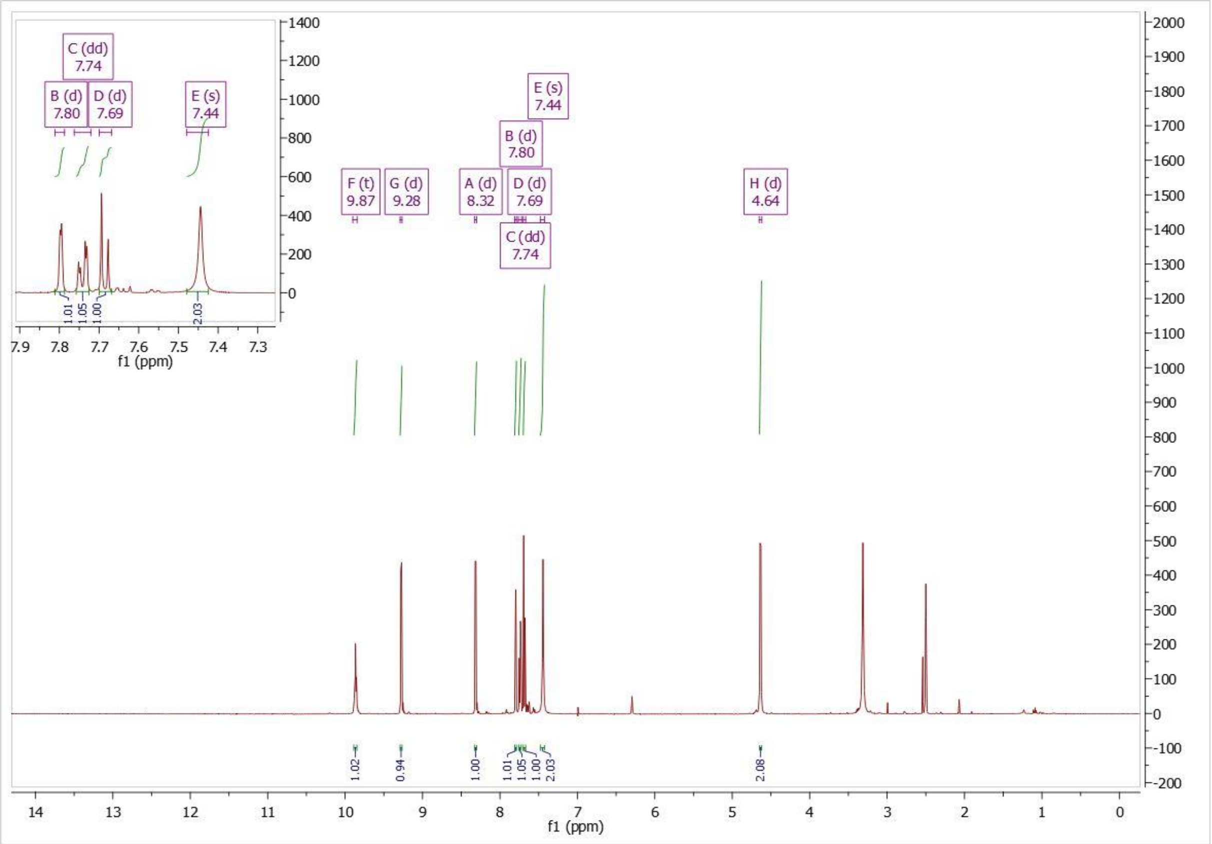

C (dd) 7.74
B (d) 7.80
D (d) 7.69
E (s) 7.44
F (t) 9.87
G (d) 9.28
A (d) 8.32
H (d) 4.64
1.02
0.94
1.00
1.01
1.05
1.00
2.03
2.08
f1 (ppm)

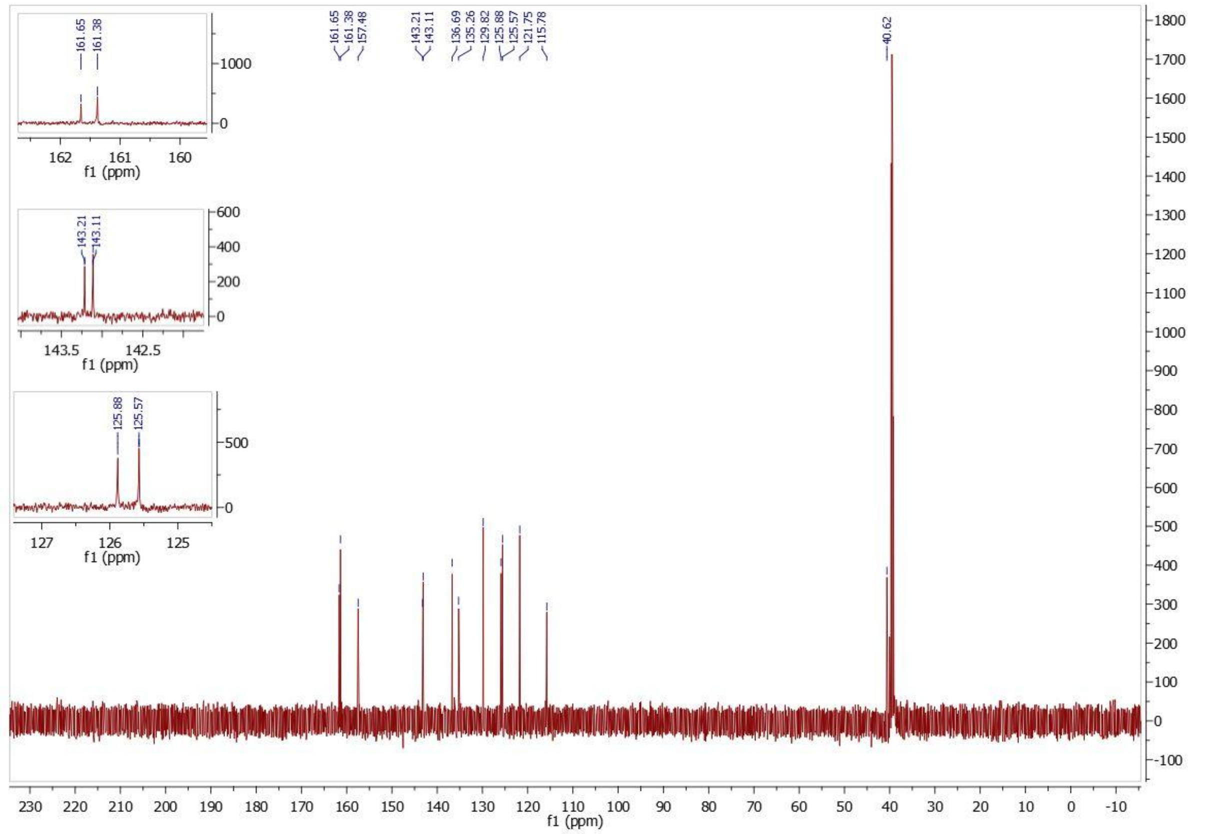

161.65
161.38
157.48
143.21
143.11
136.69
135.26
129.82
125.88
125.57
121.75
115.78
40.62
f1 (ppm)

2-Chloro-*N*-[(3-sulfamoylphenyl)methyl]pyrimidine-4-carboxamide (54)

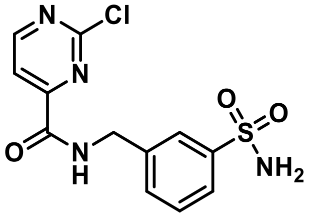

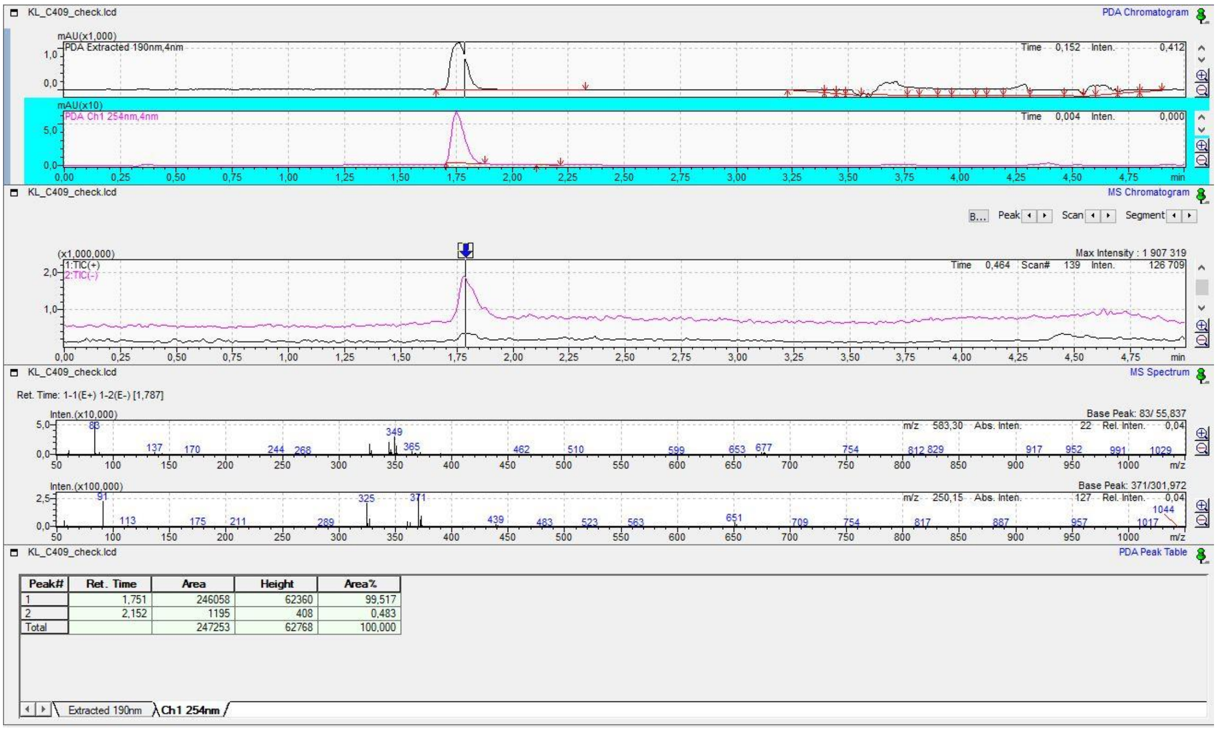

Peak#	Ret. Time	Area	Height	Area%
1	1,751	246058	62360	99,517
2	2,152	1195	408	0,483
Total		247253	62768	100,000

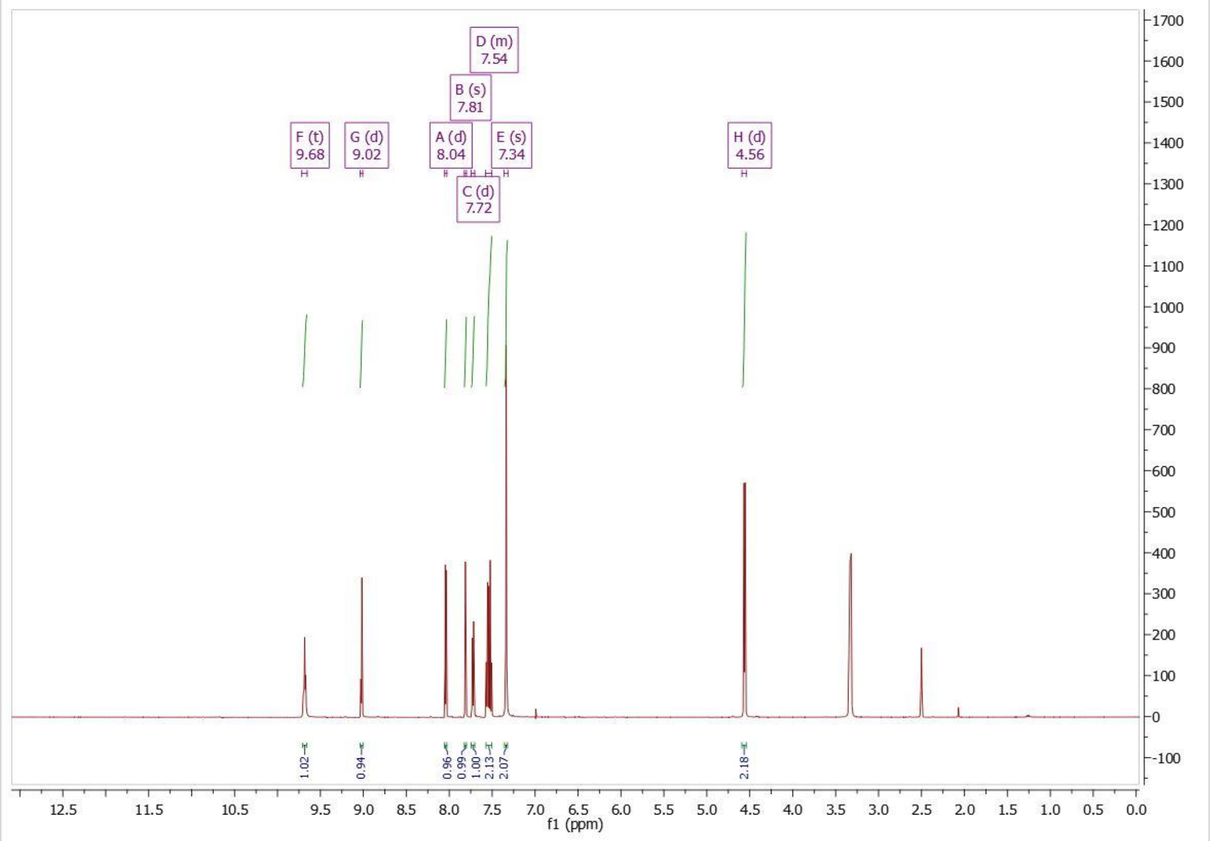
D (m)
7.54
B (s)
7.81
F (t)
9.68
G (d)
9.02
A (d)
8.04
E (s)
7.34
H (d)
4.56
C (d)
7.72
1.02
0.94
0.96
0.99
1.00
2.13
2.07
2.18
f1 (ppm)

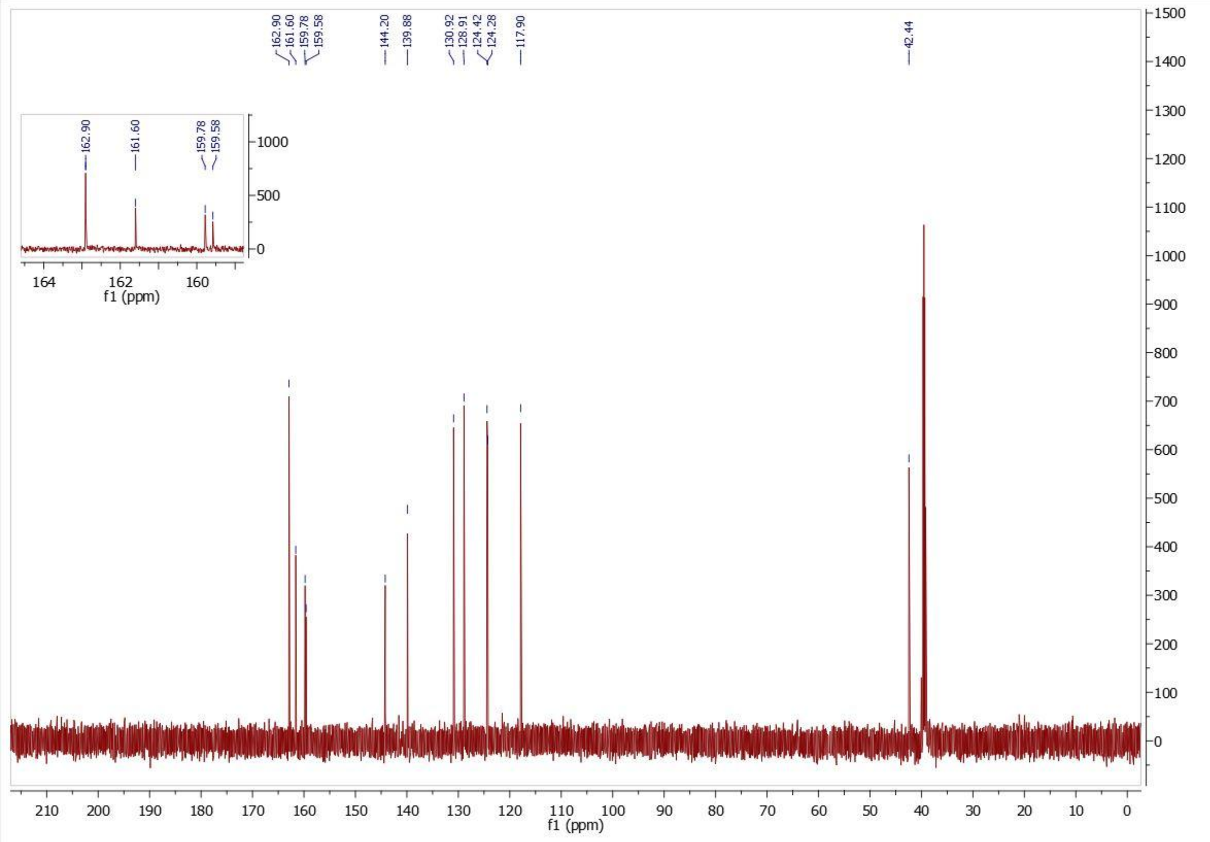
162.90
161.60
159.78
159.58
144.20
139.88
130.92
128.91
124.42
124.28
117.90
42.44
f1 (ppm)

3-(Aminomethyl)-4-chlorobenzene-1-sulfonic acid (56)

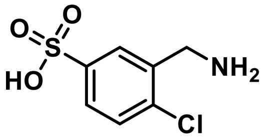

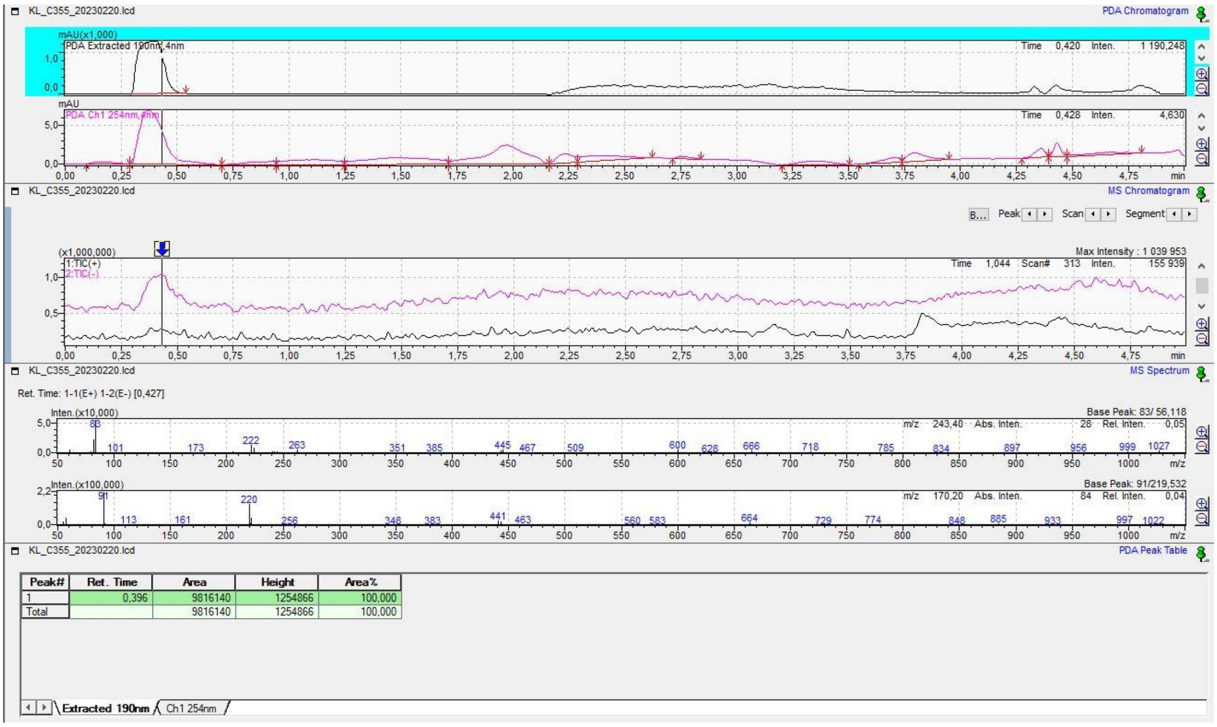

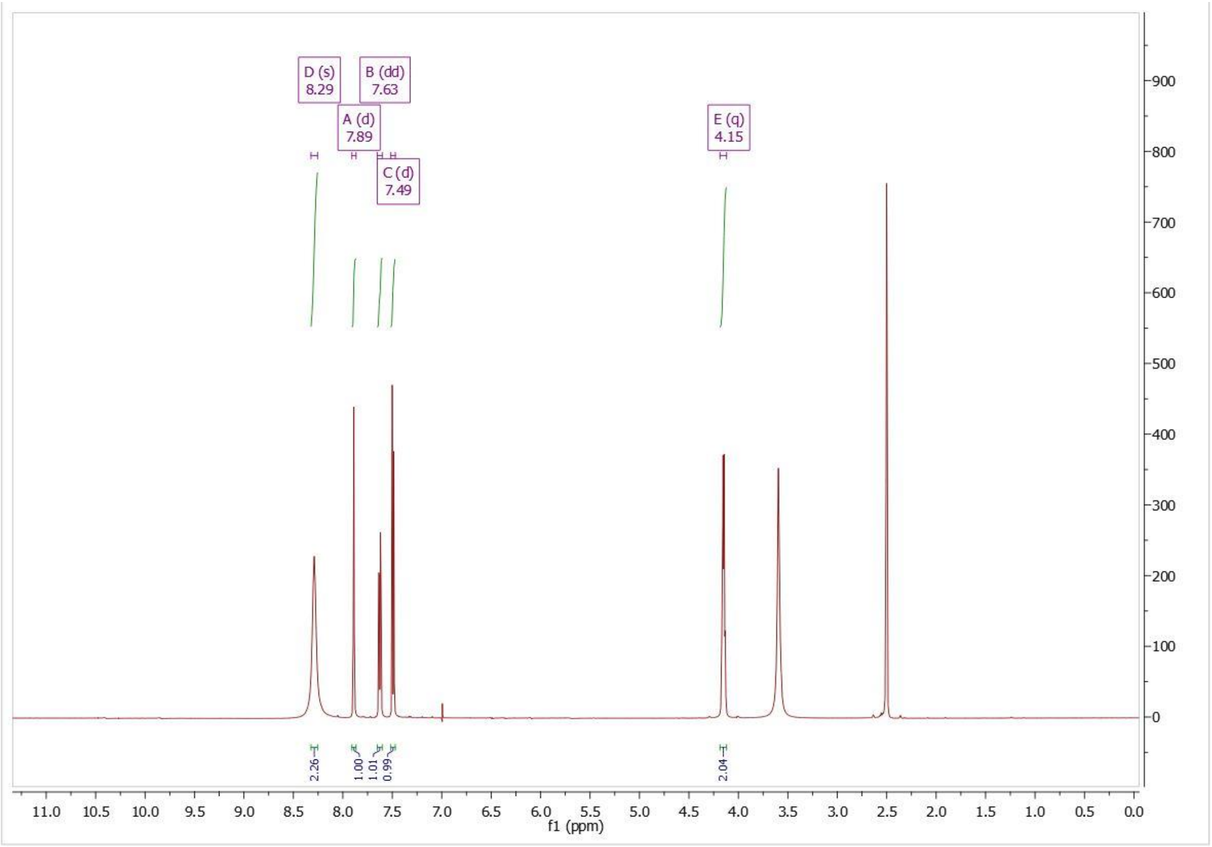

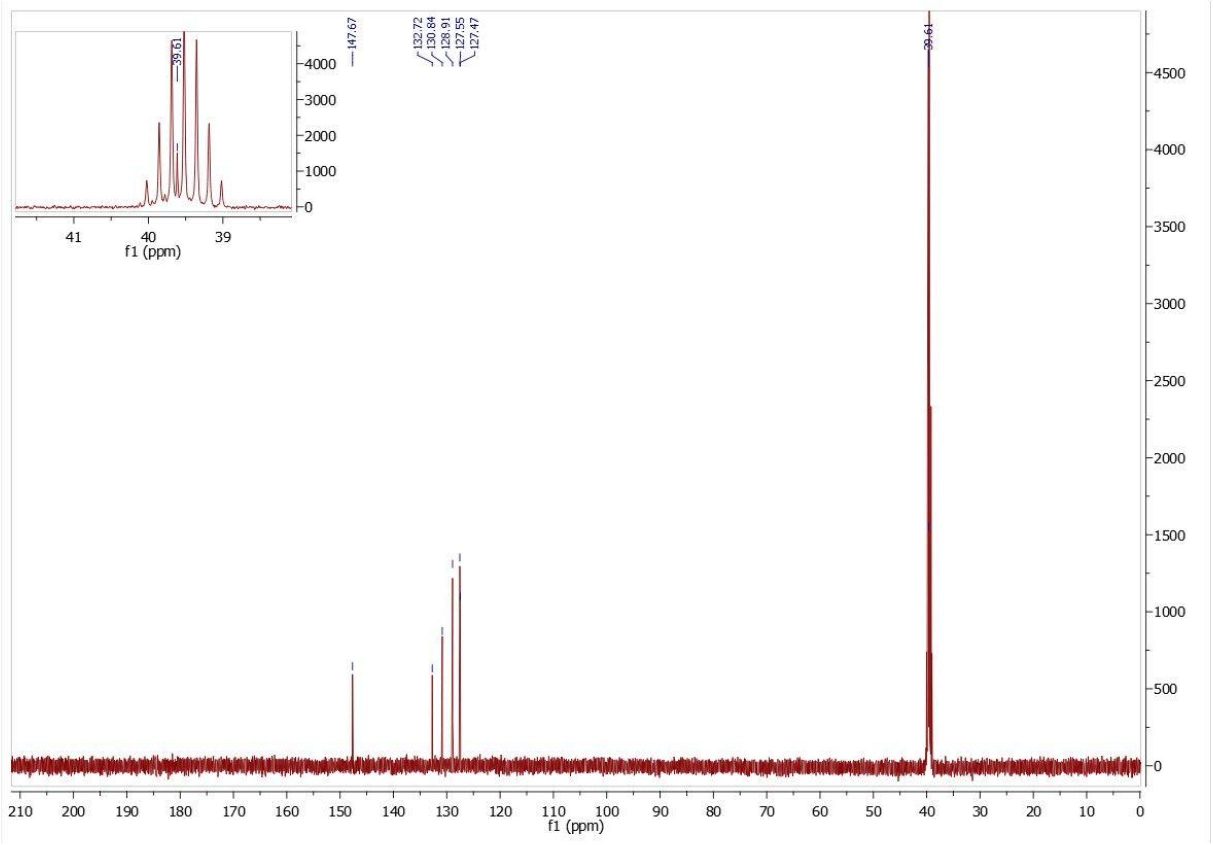
147.67
132.72
130.84
128.91
127.55
127.47
39.61
f1 (ppm)

Ethylbis(propan-2-yl)azanium 4-chloro-3-[[(2-chloropyrimidin-4-yl)formamido]methyl]benzene-1-sulfonate (57)

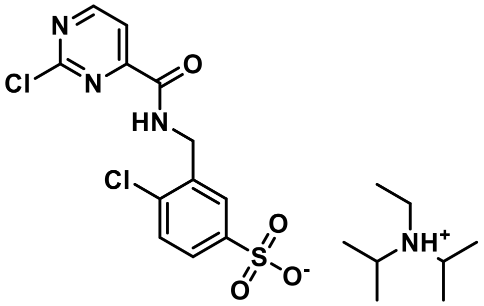

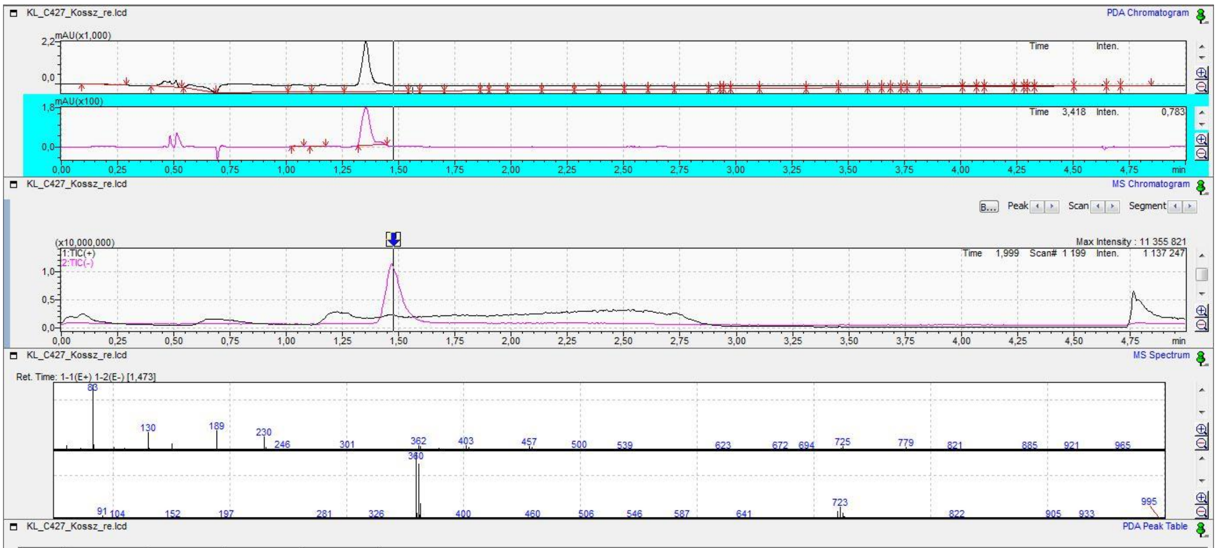

Peak#	Ret. Time	Height	Area	Area%
1	1,040	2550	4953	1,074
2	1,134	1031	1791	0,388
3	1,355	167746	454387	98,538
Total		171327	461131	100,000

Extracted 190nm | Ch1 254nm

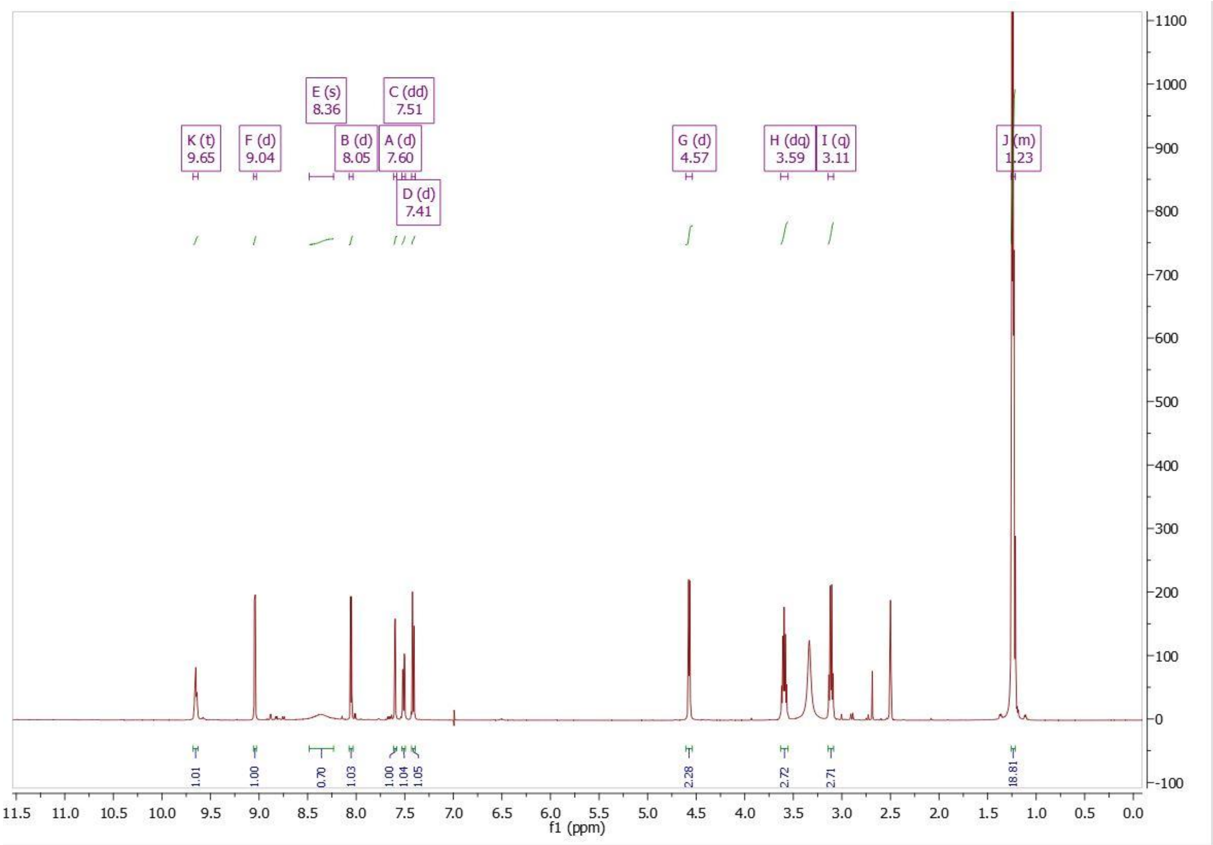
K (t)
9.65
F (d)
9.04
E (s)
8.36
B (d)
8.05
C (dd)
7.51
A (d)
7.60
D (d)
7.41
G (d)
4.57
H (dq)
3.59
I (q)
3.11
J (m)
1.23
1.01
1.00
0.70
1.03
1.00
1.04
1.05
2.28
2.72
2.71
18.81
f1 (ppm)

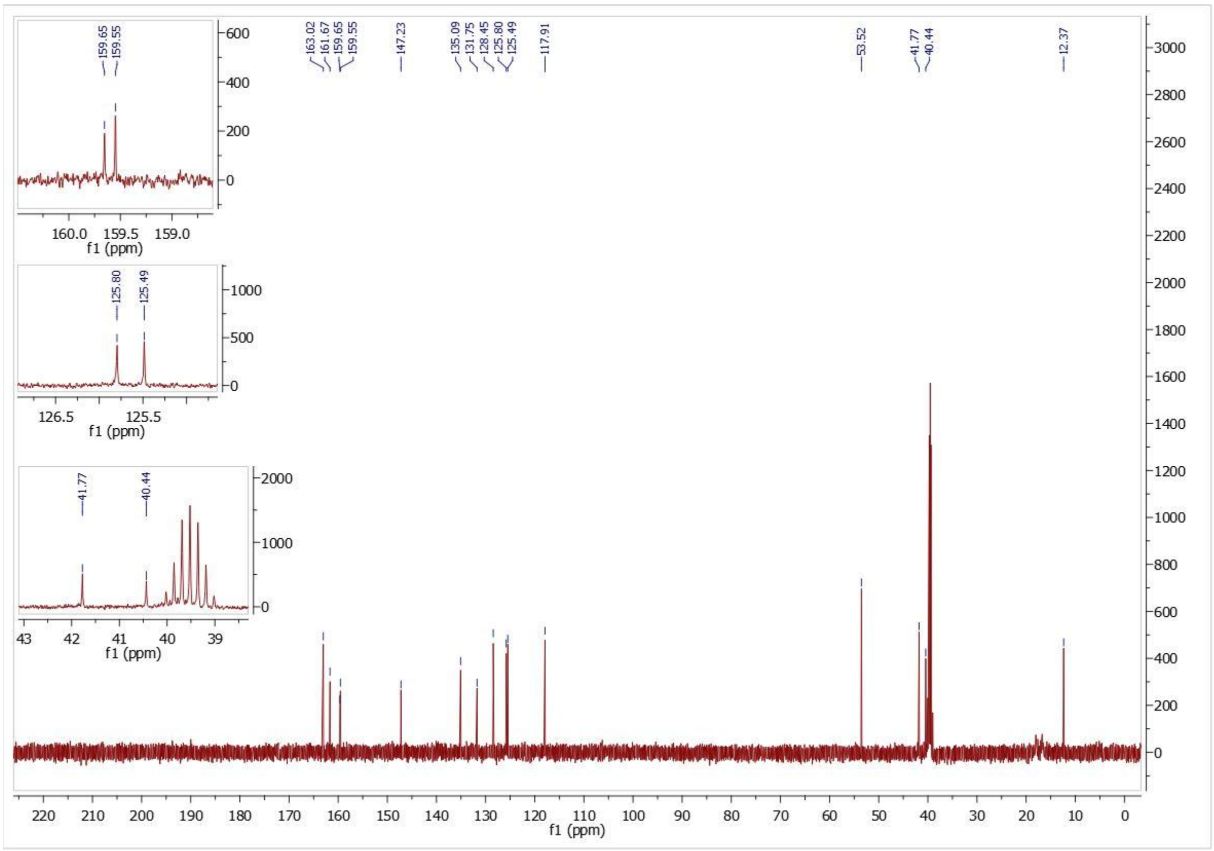
163.02
161.67
159.65
159.55
147.23
135.09
131.75
128.45
125.80
125.49
117.91
53.52
41.77
40.44
12.37
f1 (ppm)

2-Chloro-*N*-[(2-chloro-5-sulfamoylphenyl)methyl]pyrimidine-4-carboxamide (58)

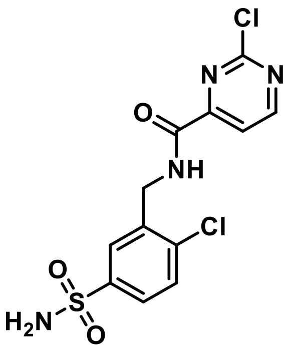

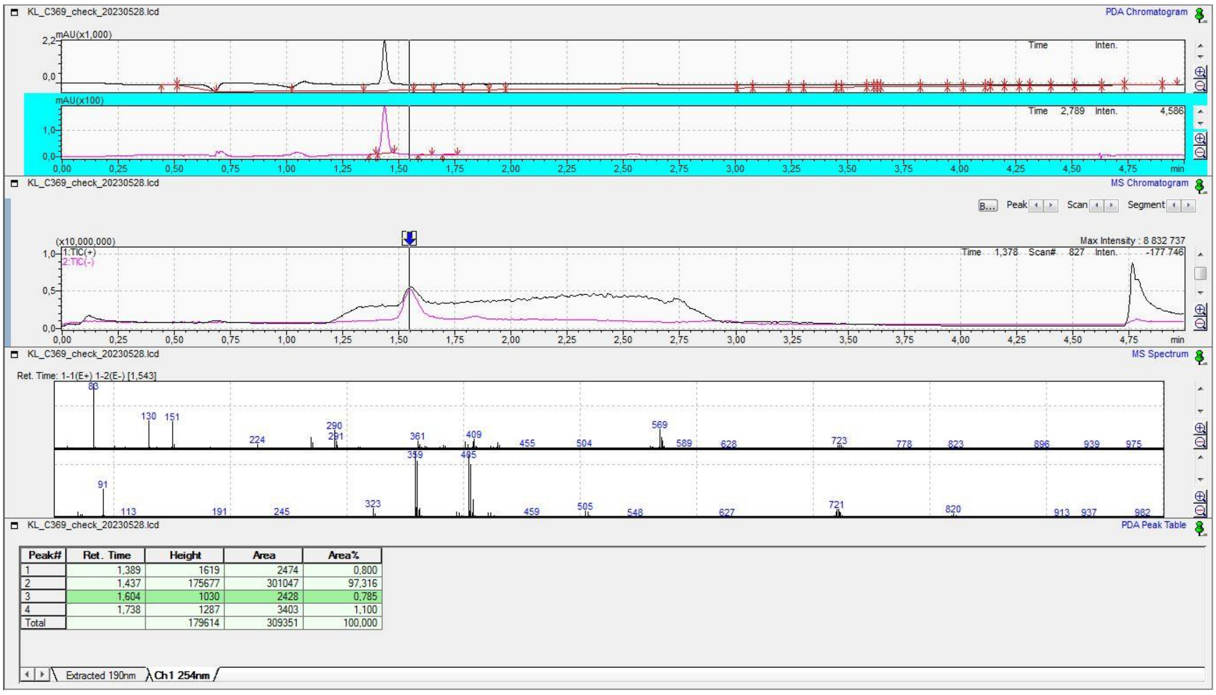

Peak#	Ret. Time	Height	Area	Area%
1	1,389	1619	2474	0,800
2	1,437	175677	301047	97,316
3	1,604	1030	2428	0,785
4	1,738	1287	3403	1,100
Total		179614	309351	100,000

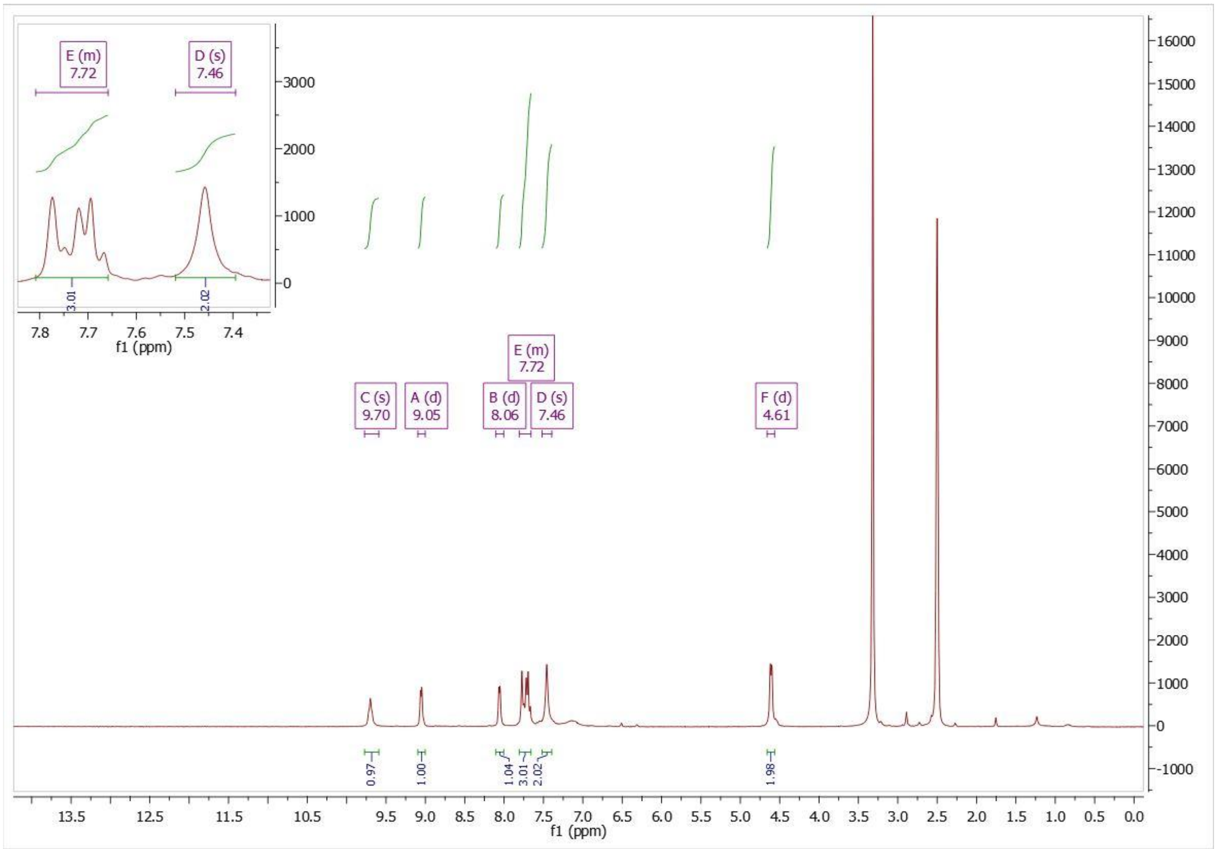
E (m)
7.72
D (s)
7.46
3.01
2.02
f1 (ppm)
C (s)
9.70
A (d)
9.05
B (d)
8.06
E (m)
7.72
D (s)
7.46
F (d)
4.61
0.97
1.00
1.04
3.01
2.02
1.98
f1 (ppm)

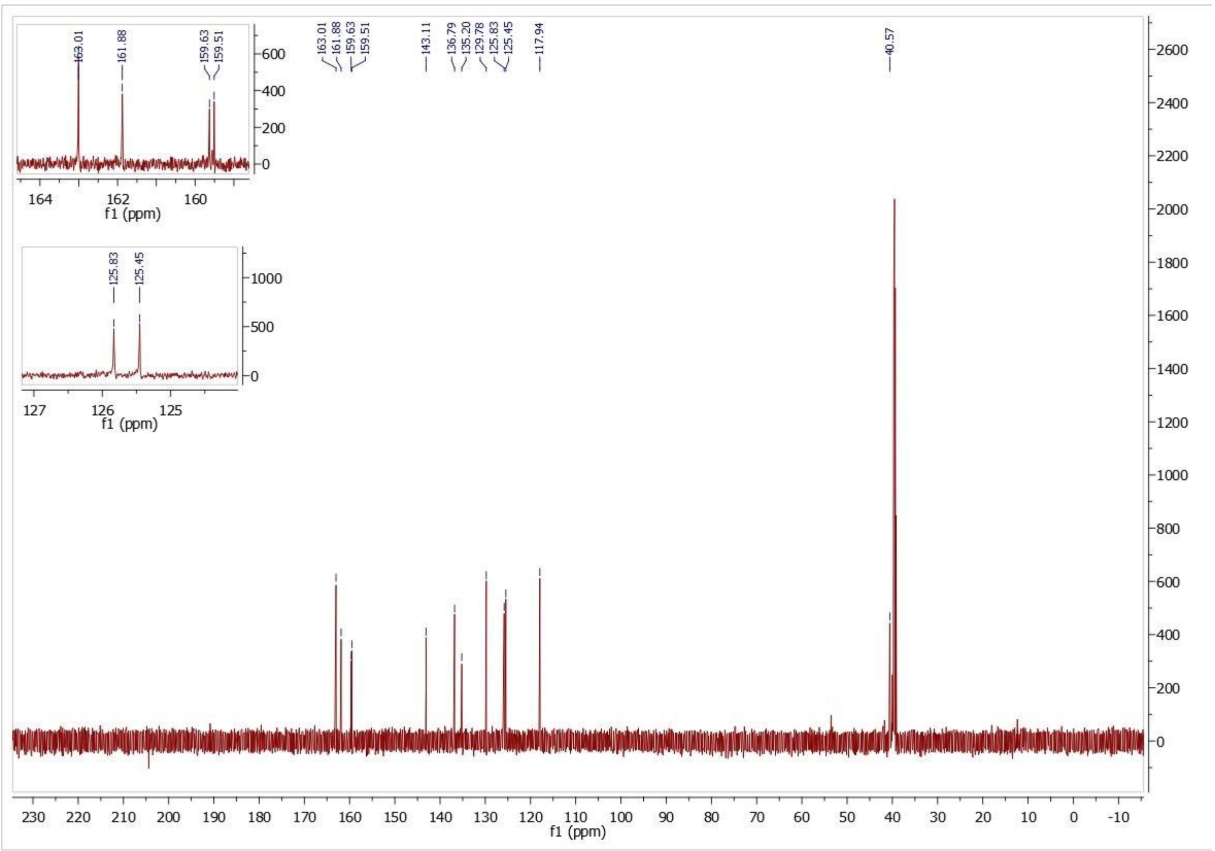
163.01
161.88
159.63
159.51
f1 (ppm)
125.83
125.45
f1 (ppm)
163.01
161.88
159.63
159.51
143.11
136.79
135.20
129.78
125.83
125.45
117.94
40.57
f1 (ppm)

***N*-[(2-bromo-5-sulfamoylphenyl)methyl]-2-cyano-6-methylpyrimidine-4-carboxamide (59)**

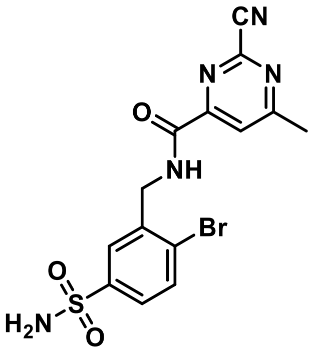

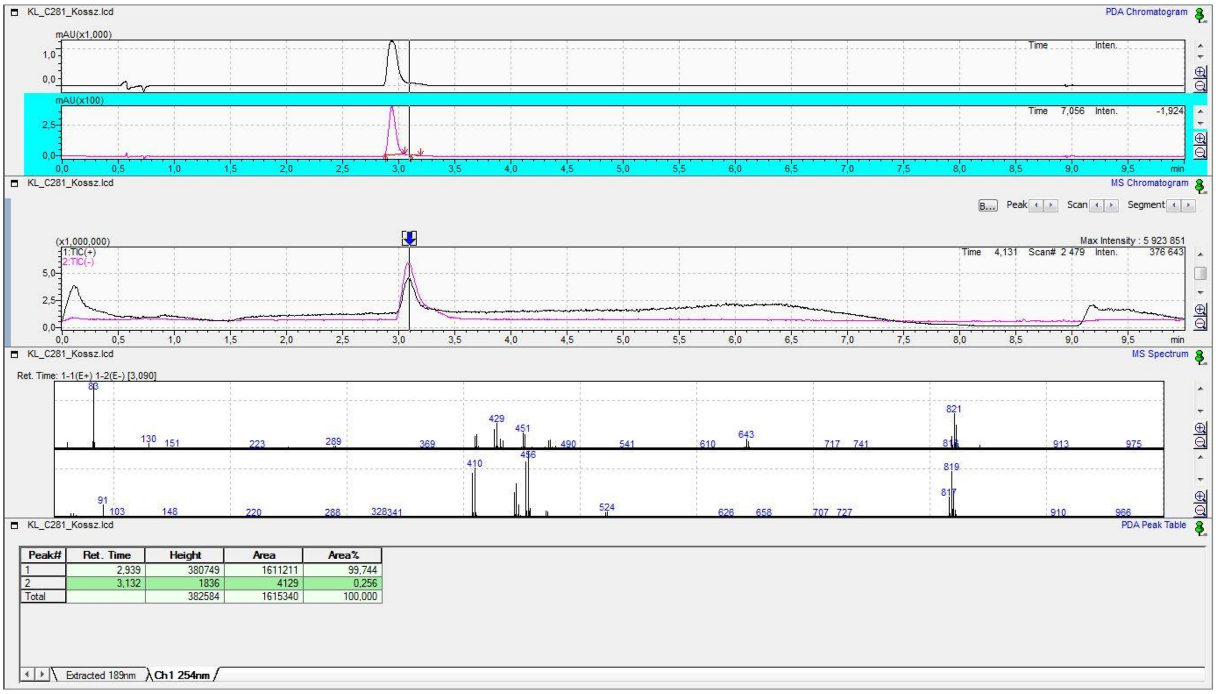

Peak#	Ret. Time	Height	Area	Area%
1	2,939	380749	1611211	99,744
2	3,132	1836	4129	0,256
Total		382584	1615340	100,000

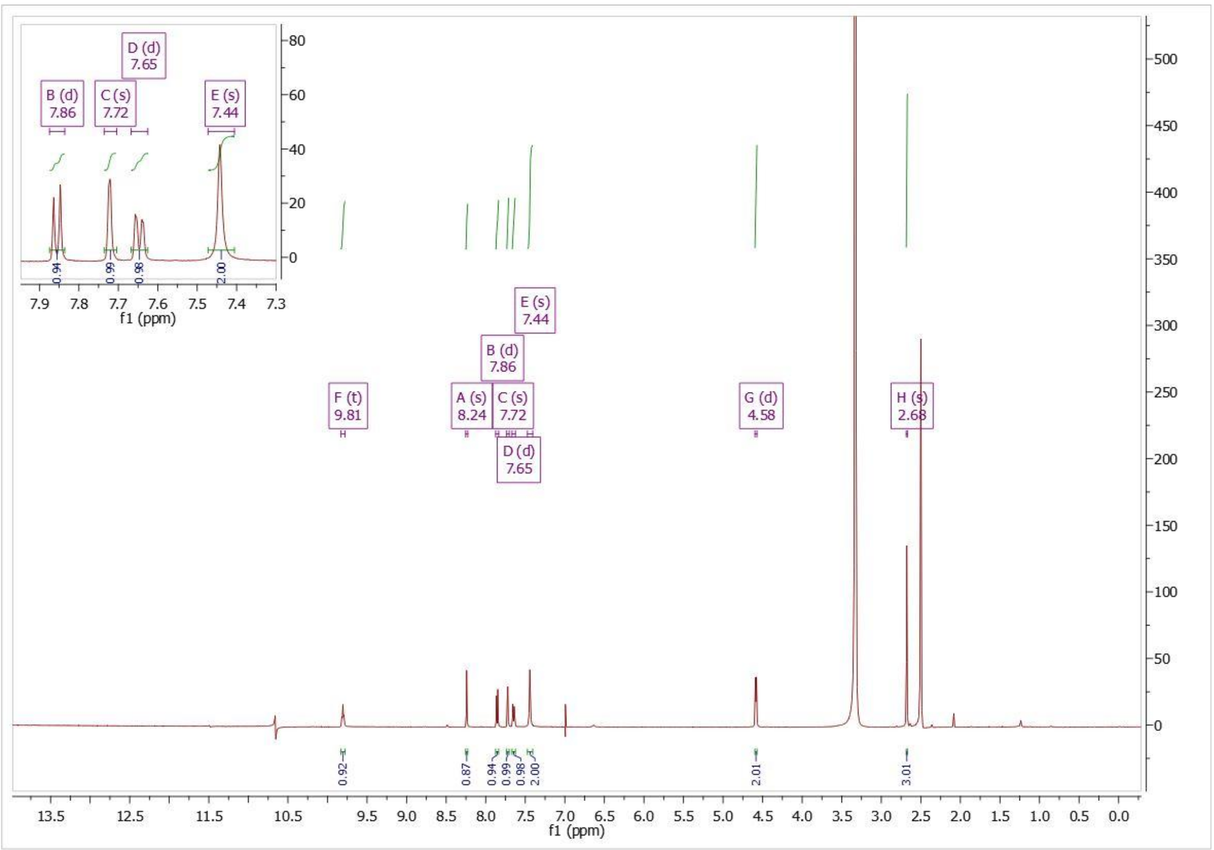
B (d) 7.86
C (s) 7.72
D (d) 7.65
E (s) 7.44
F (t) 9.81
A (s) 8.24
G (d) 4.58
H (s) 2.68
0.92
0.87
0.94
0.99
0.98
2.00
2.01
3.01
f1 (ppm)

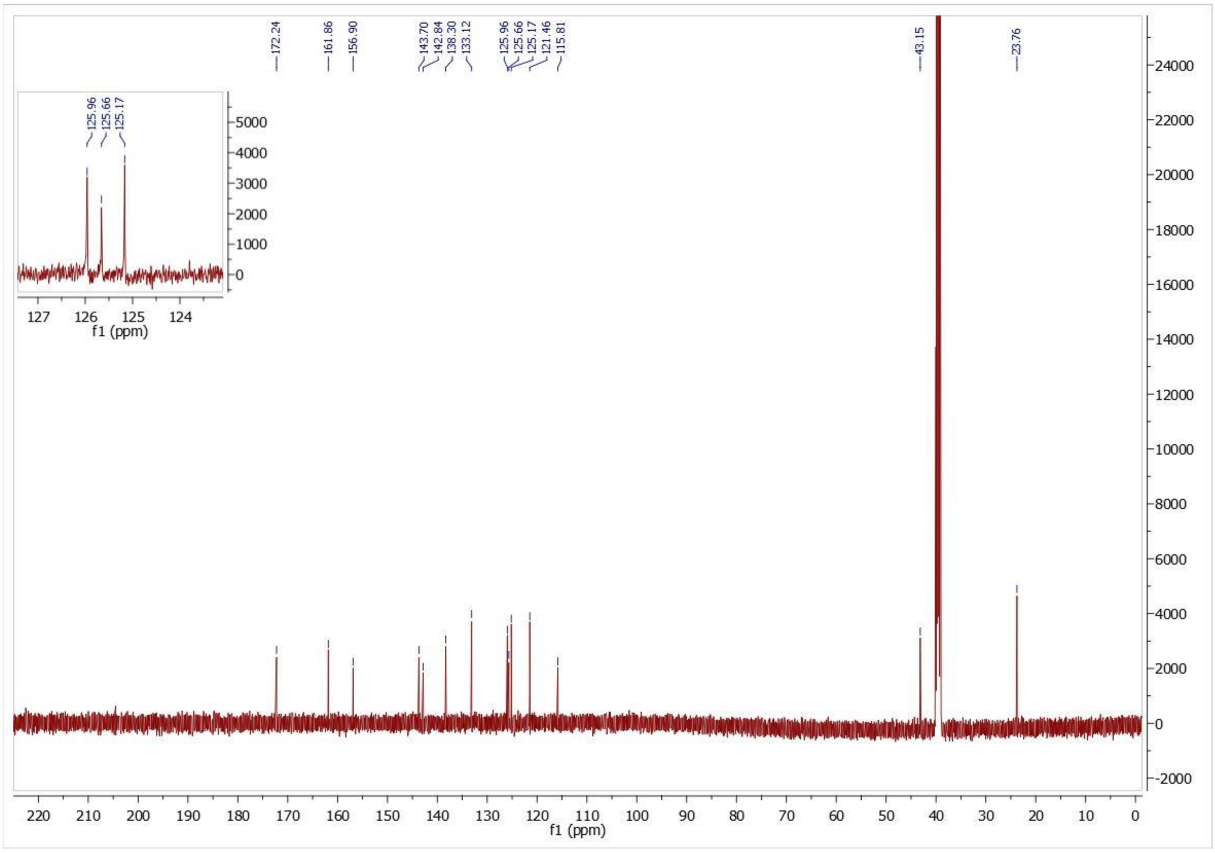
172.24
161.86
156.90
143.70
142.84
138.30
133.12
125.96
125.66
125.17
121.46
115.81
43.15
23.76
f1 (ppm)

Ethylbis(propan-2-yl)azanium 4-bromo-3[[(2-chloro-6-methylpyrimidin-4-yl)formamido]methyl]benzene-1- sulfonate (61)

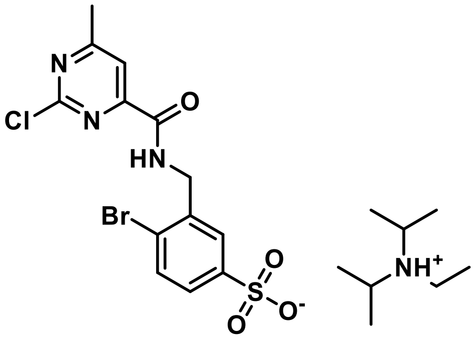

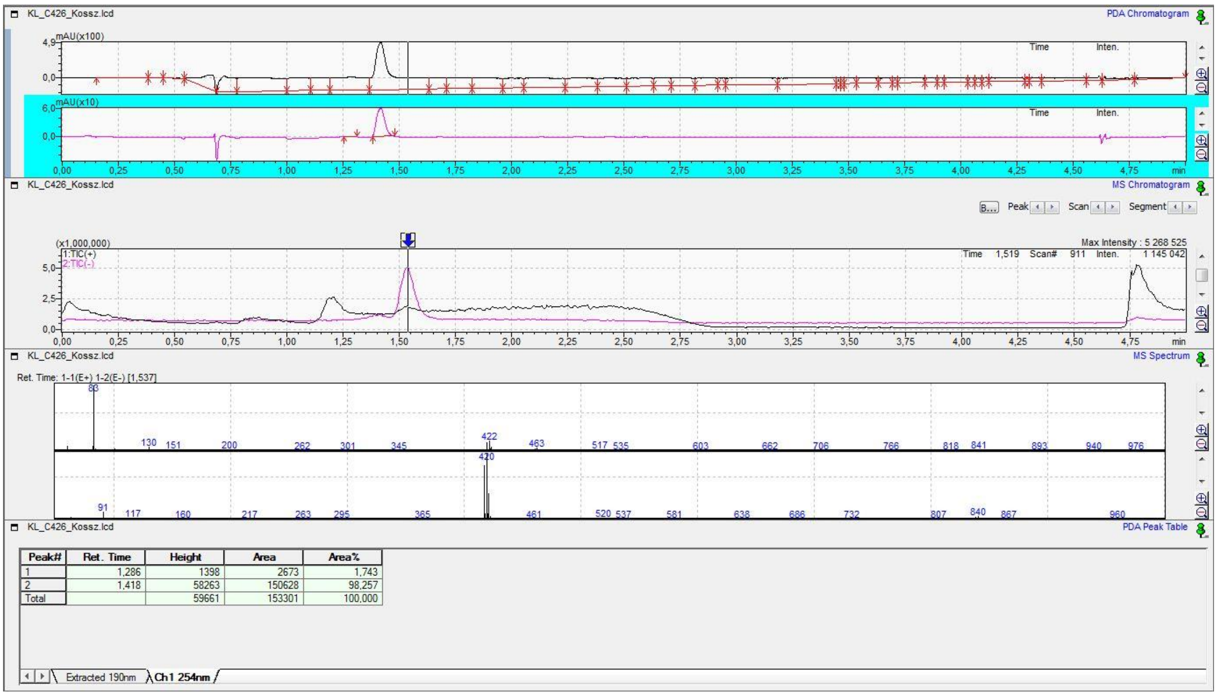

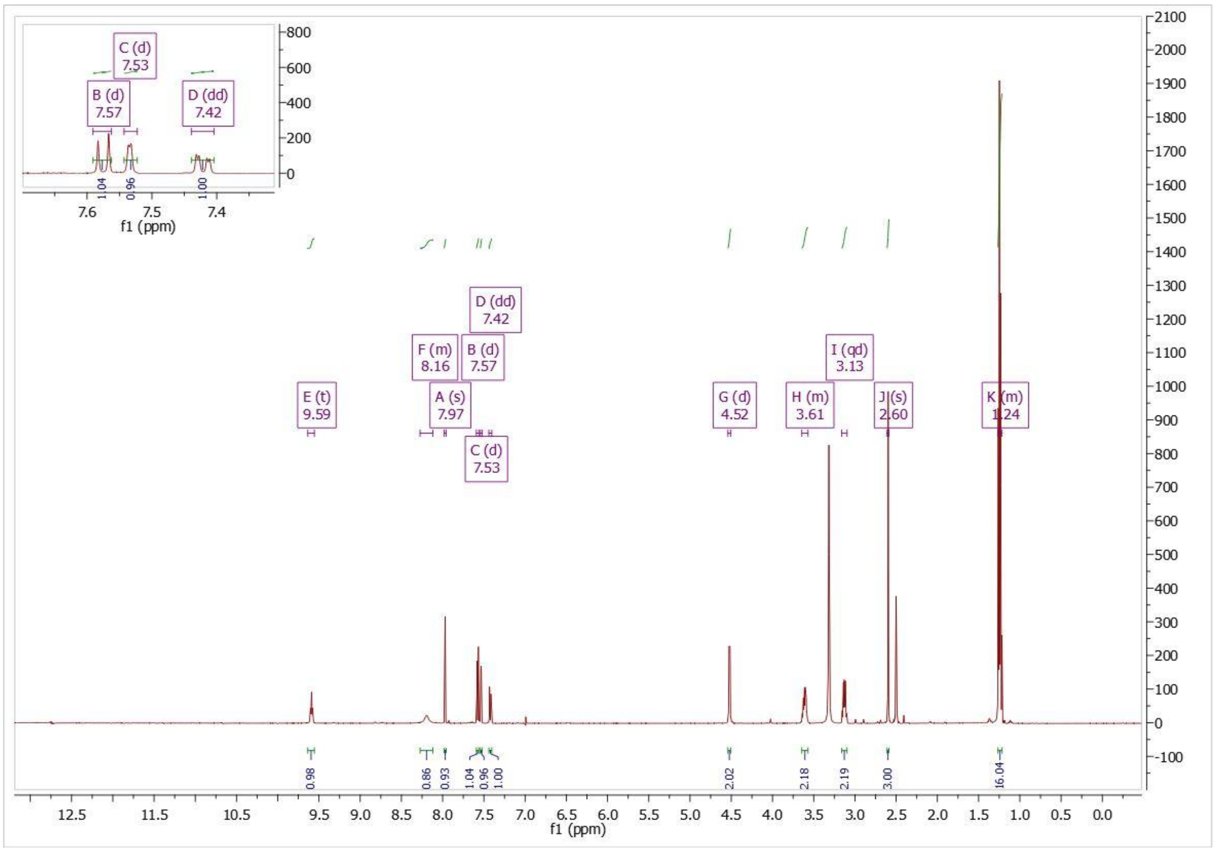
C (d)
7.53
B (d)
7.57
D (dd)
7.42
1.04
0.96
1.00
7.6
7.5
7.4
f1 (ppm)
E (t)
9.59
F (m)
8.16
A (s)
7.97
B (d)
7.57
D (dd)
7.42
C (d)
7.53
G (d)
4.52
H (m)
3.61
I (qd)
3.13
J (s)
2.60
K (m)
1.24
0.98
0.86
0.93
1.04
0.96
1.00
2.02
2.18
2.19
3.00
16.04
f1 (ppm)

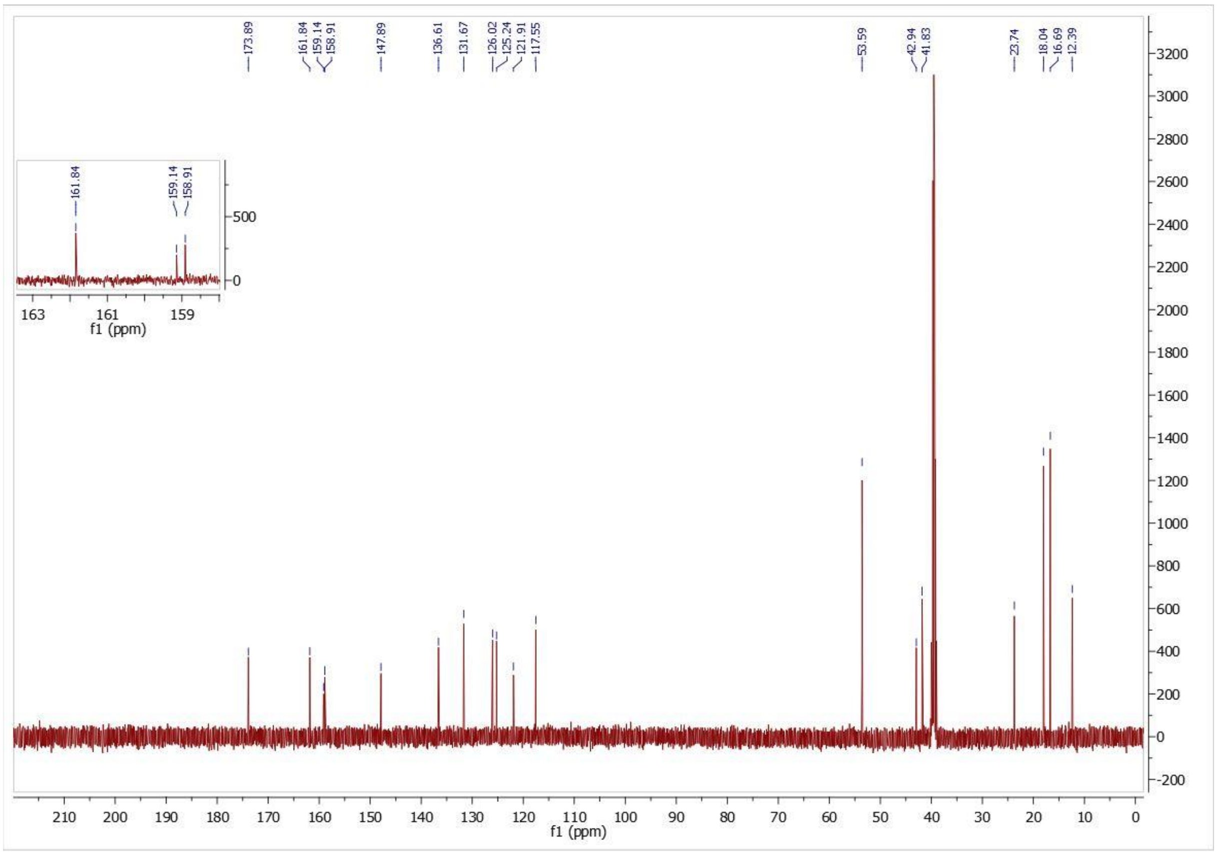
173.89
161.84
159.14
158.91
147.89
136.61
131.67
126.02
125.24
121.91
117.55
53.59
42.94
41.83
23.74
18.04
16.69
12.39
161.84
159.14
158.91
163
161
159
f1 (ppm)
f1 (ppm)

N-[(2-bromo-5-sulfamoylphenyl)methyl]-2-chloro-6-methylpyrimidine-4-carboxamide (62)

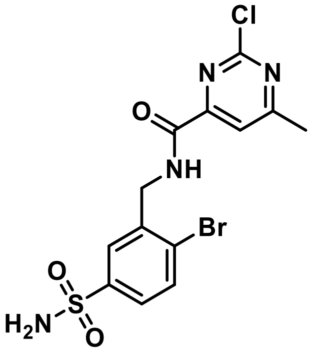

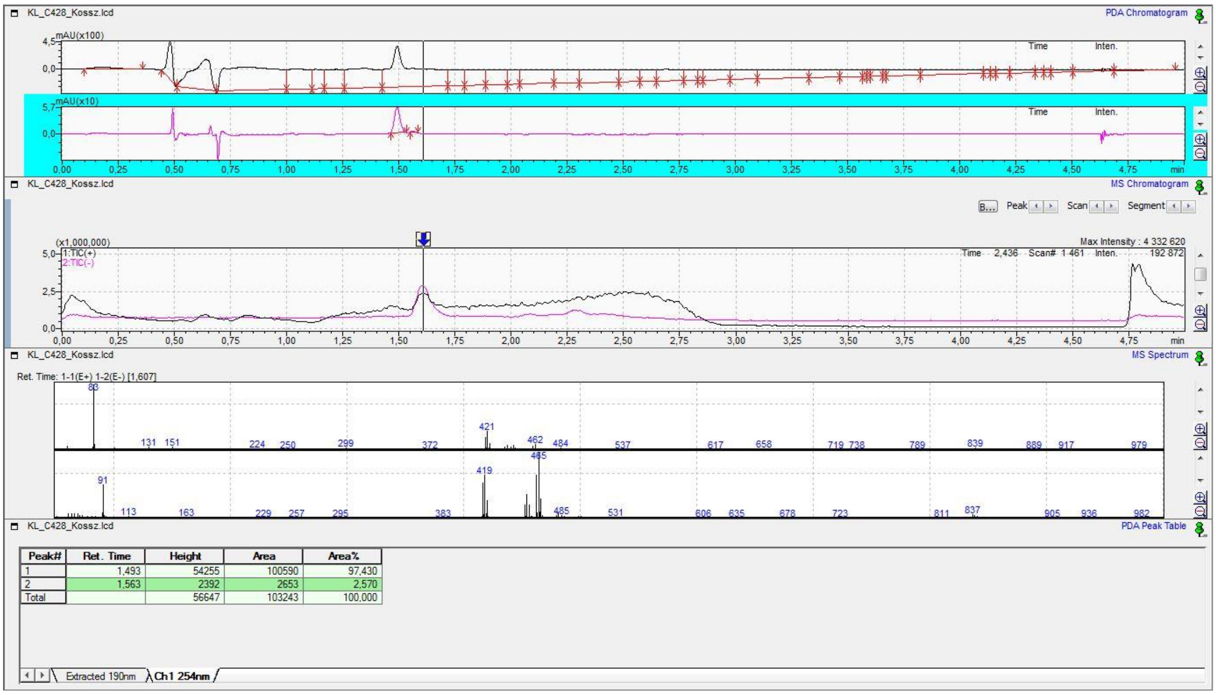

Peak#	Ret. Time	Height	Area	Area%
1	1,493	54255	100590	97,430
2	1,563	2392	2653	2,570
Total		56647	103243	100,000

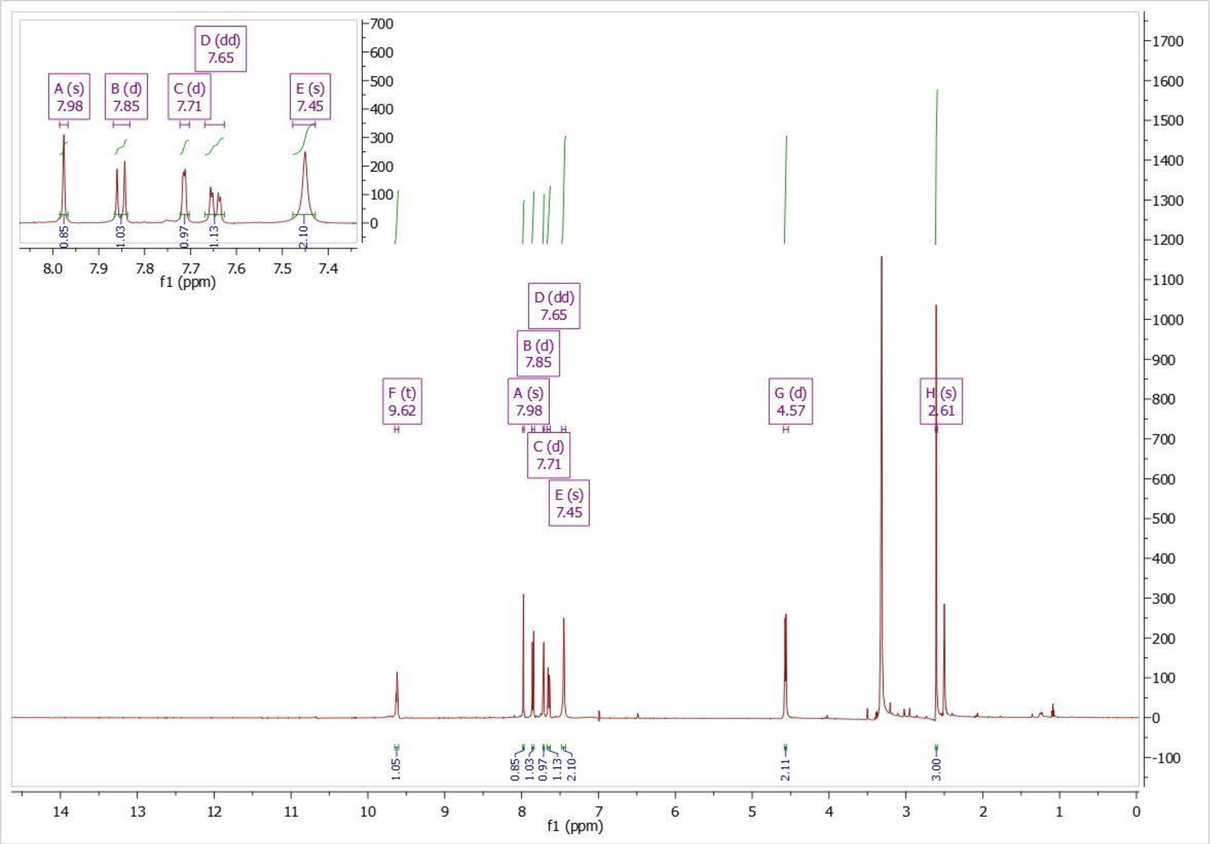

A (s)
7.98
B (d)
7.85
C (d)
7.71
D (dd)
7.65
E (s)
7.45
F (t)
9.62
G (d)
4.57
H (s)
2.61
f1 (ppm)

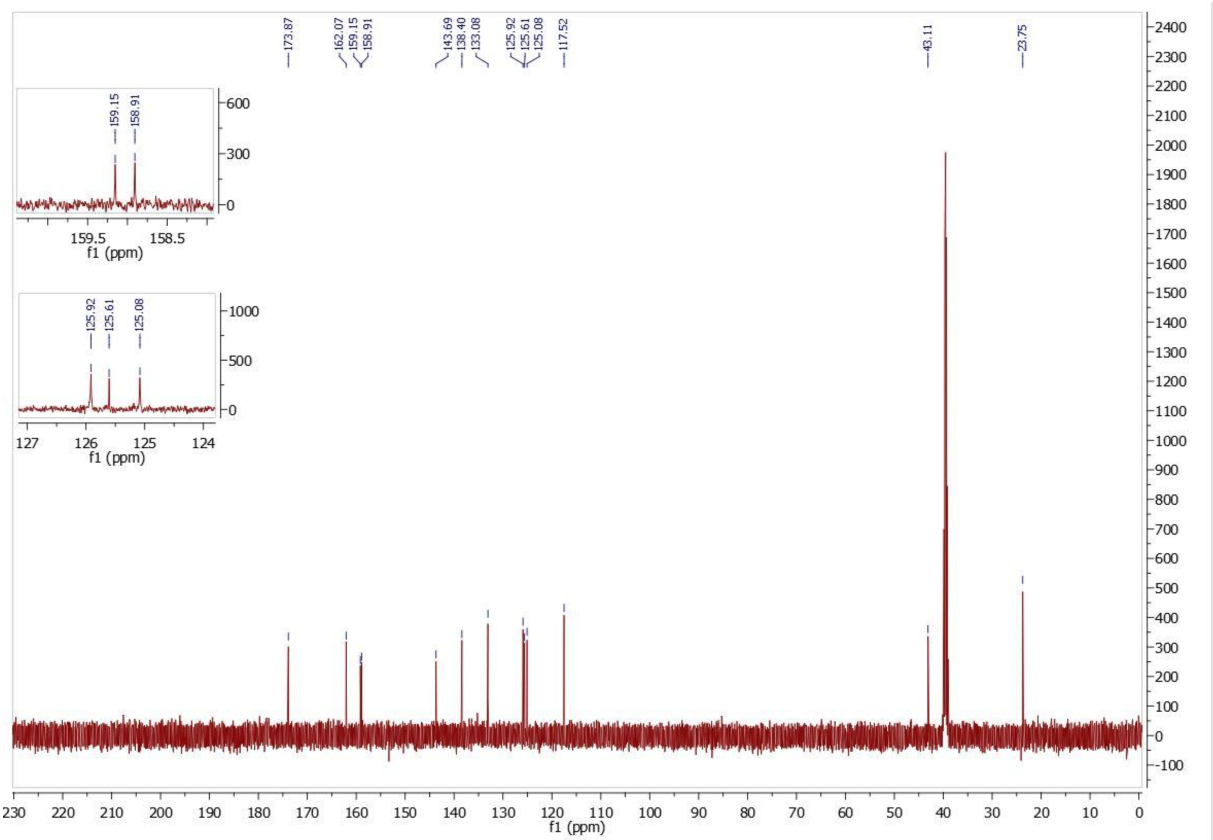

173.87
162.07
159.15
158.91
143.69
138.40
133.08
125.92
125.61
125.08
117.52
43.11
23.75
f1 (ppm)

2-Cyano-*N*-[(5-sulfamoyl-2-[[(thiophen-2-yl)methyl]amino]phenyl)methyl]pyrimidine-4-carboxamide (63)

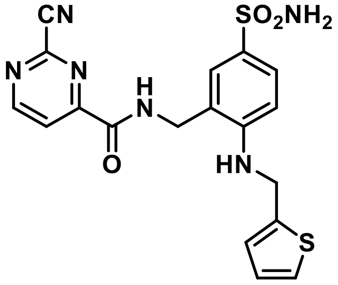

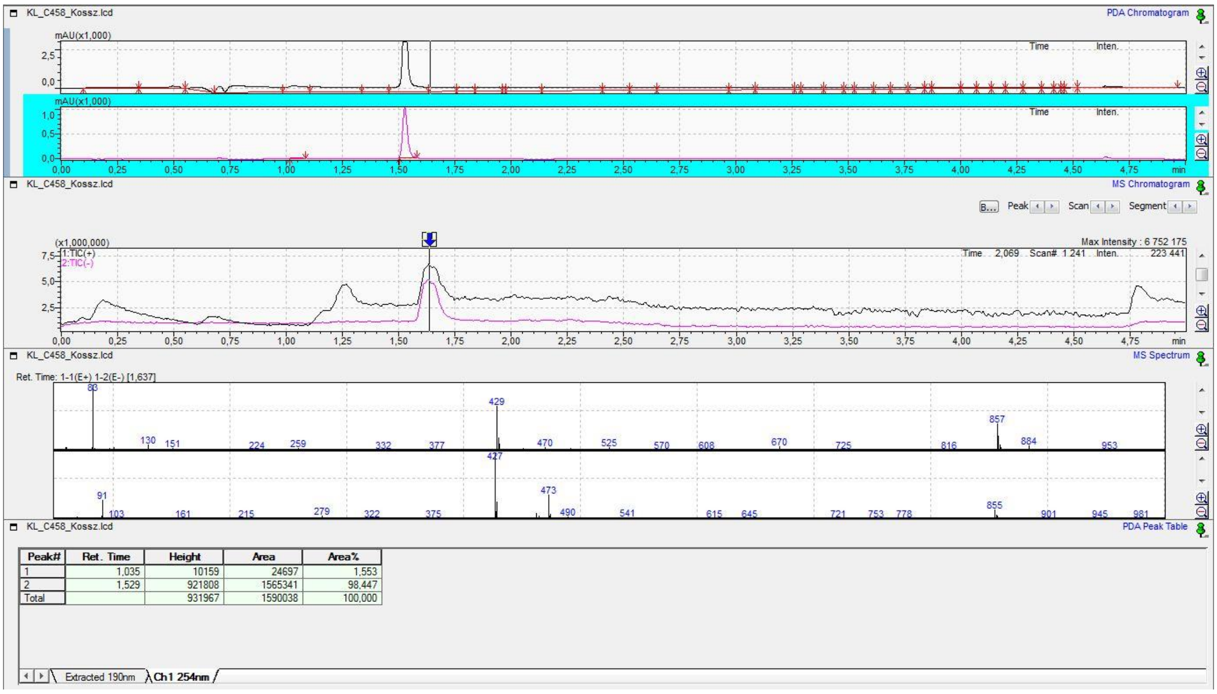

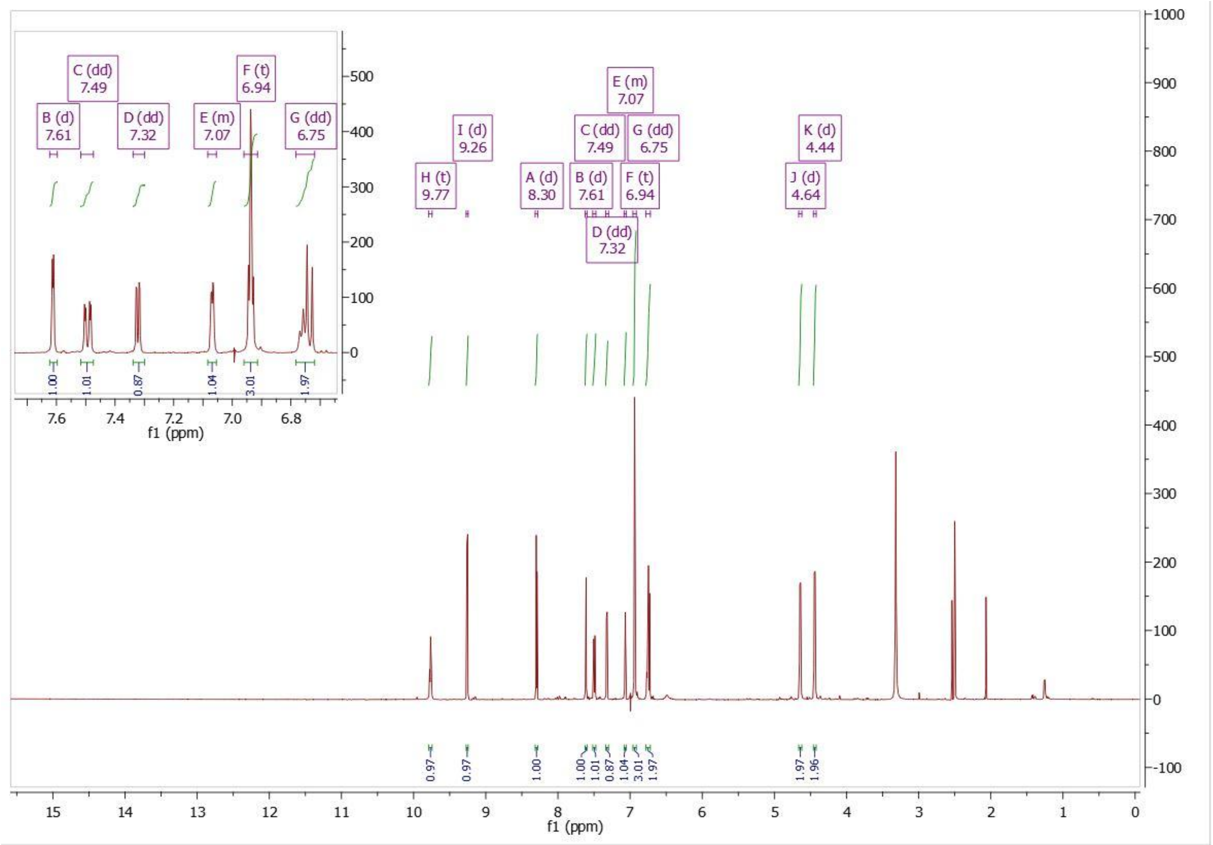
H (t) 9.77
I (d) 9.26
A (d) 8.30
B (d) 7.61
C (dd) 7.49
D (dd) 7.32
E (m) 7.07
F (t) 6.94
G (dd) 6.75
J (d) 4.64
K (d) 4.44
f1 (ppm)

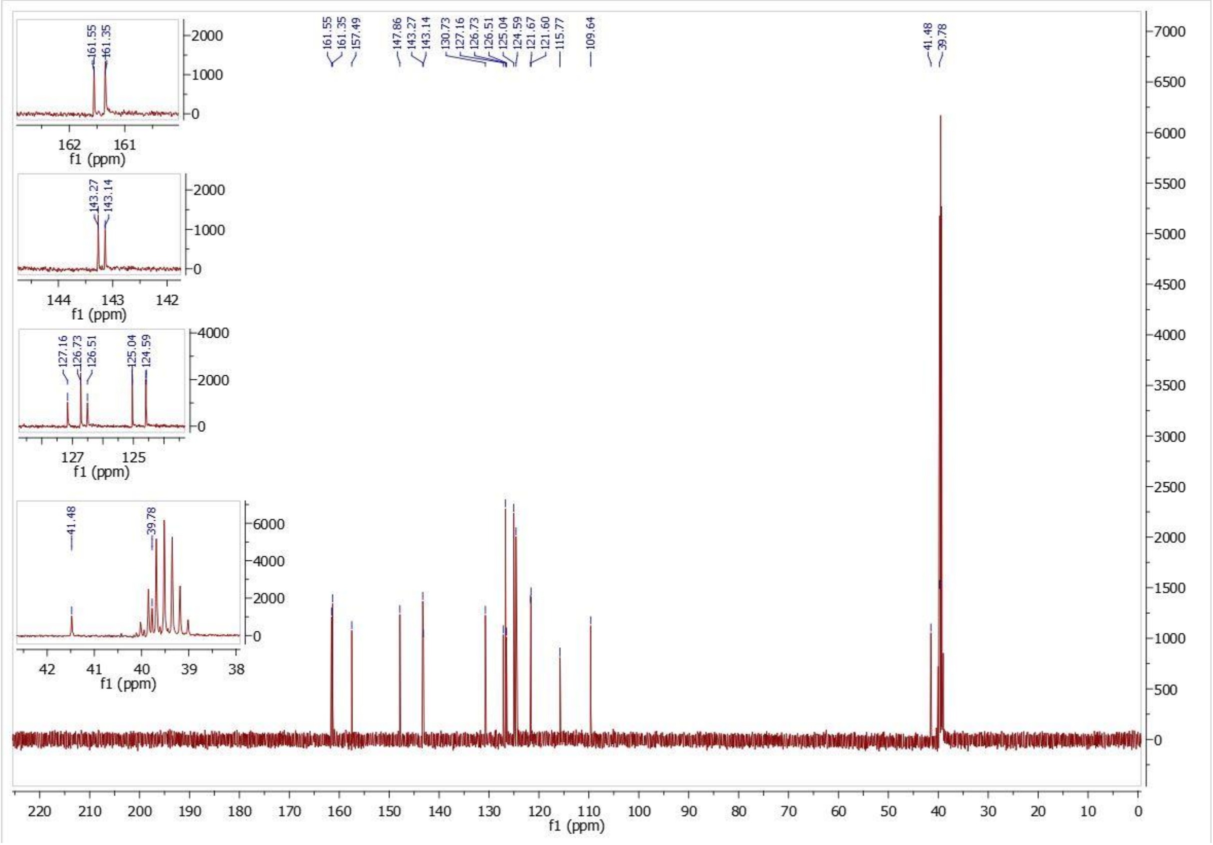
161.55
161.35
157.49
147.86
143.27
143.14
130.73
127.16
126.73
126.51
125.04
124.59
121.67
121.60
115.77
109.64
41.48
39.78
f1 (ppm)

3-Cyano-4-[[(thiophen-2-yl)methyl]amino]benzene-1-sulfonamide (65)

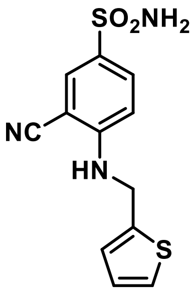

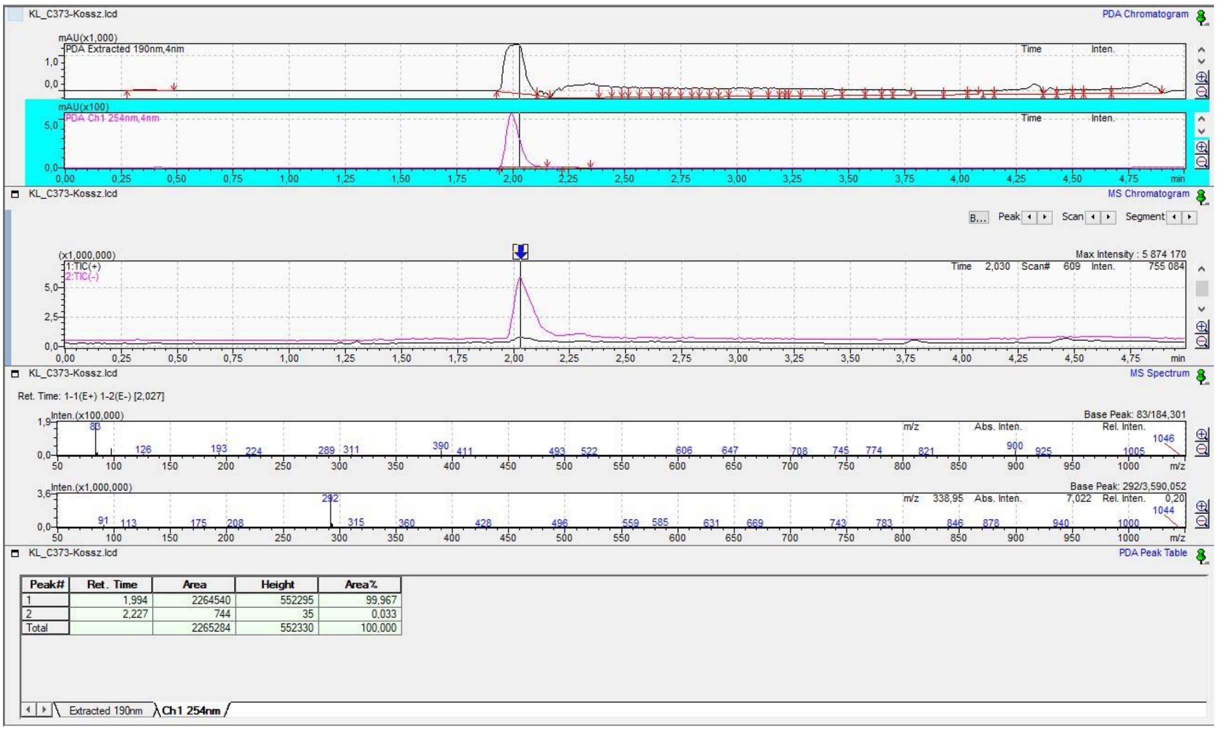

Peak#	Ret. Time	Area	Height	Area%
1	1,994	2264540	552295	99,967
2	2,227	744	35	0,033
Total		2265284	552330	100,000

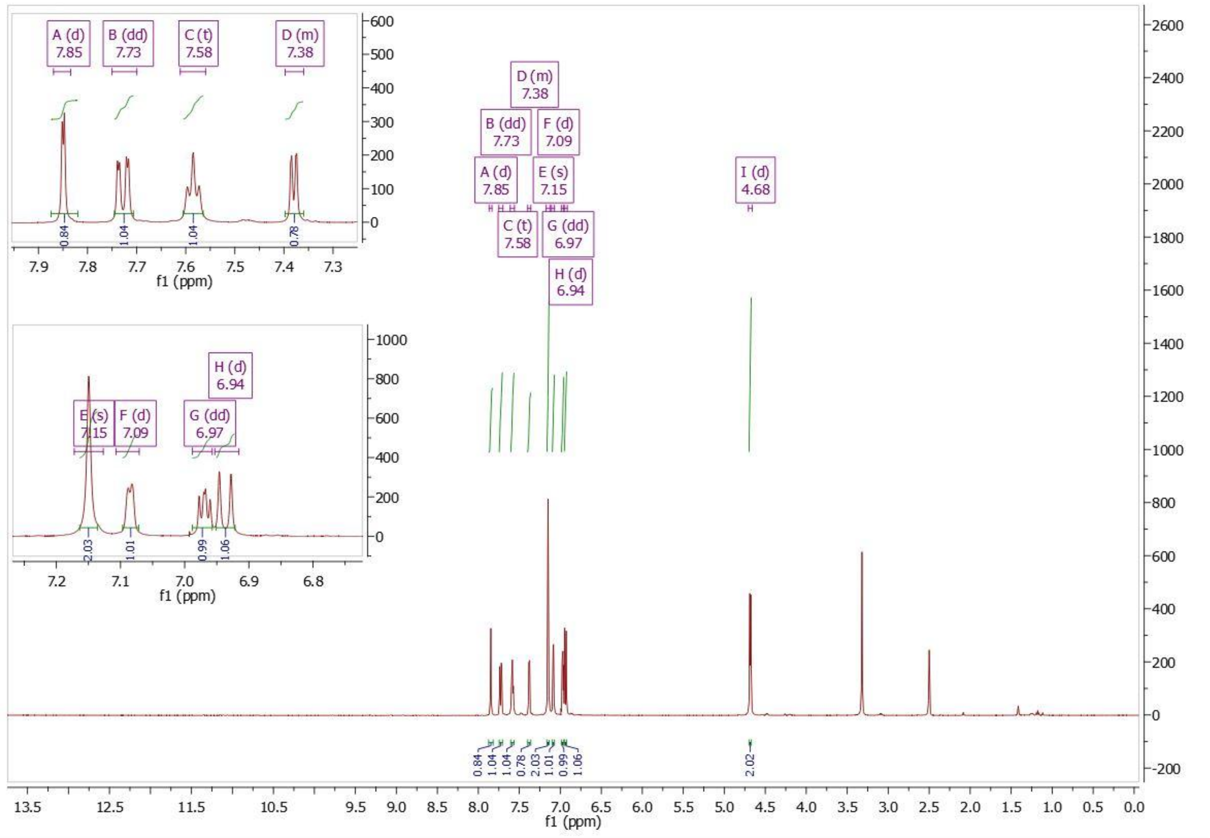
A (d) 7.85
B (dd) 7.73
C (t) 7.58
D (m) 7.38
E (s) 7.15
F (d) 7.09
G (dd) 6.97
H (d) 6.94
I (d) 4.68
0.84
1.04
1.04
0.78
2.03
1.01
0.99
1.06
2.02
f1 (ppm)

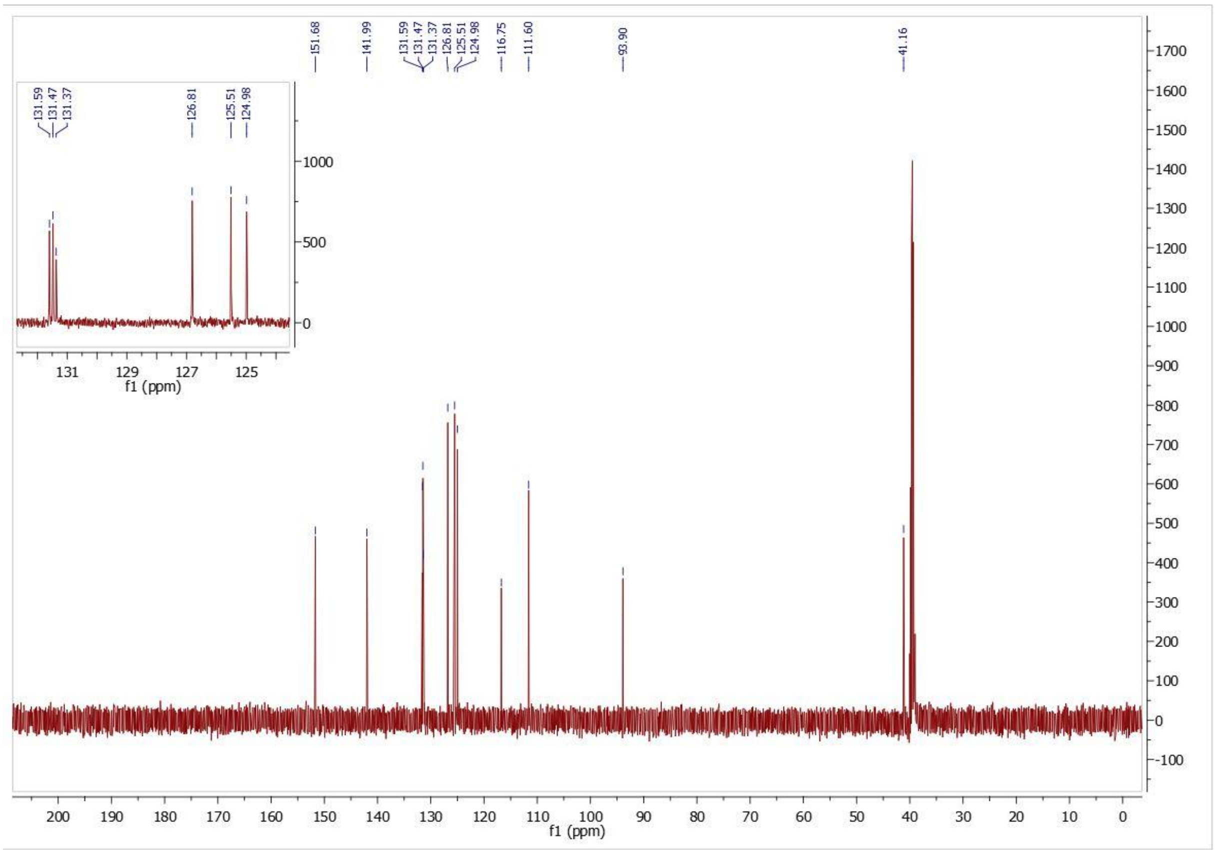
151.68
141.99
131.59
131.47
131.37
126.81
125.51
124.98
116.75
111.60
93.90
41.16
f1 (ppm)

3-(Aminomethyl)-4-[[(thiophen-2-yl)methyl]amino]benzene-1-sulfonamide (66)

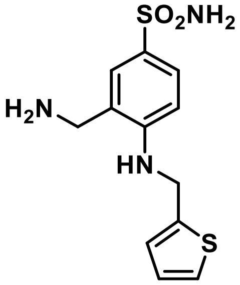

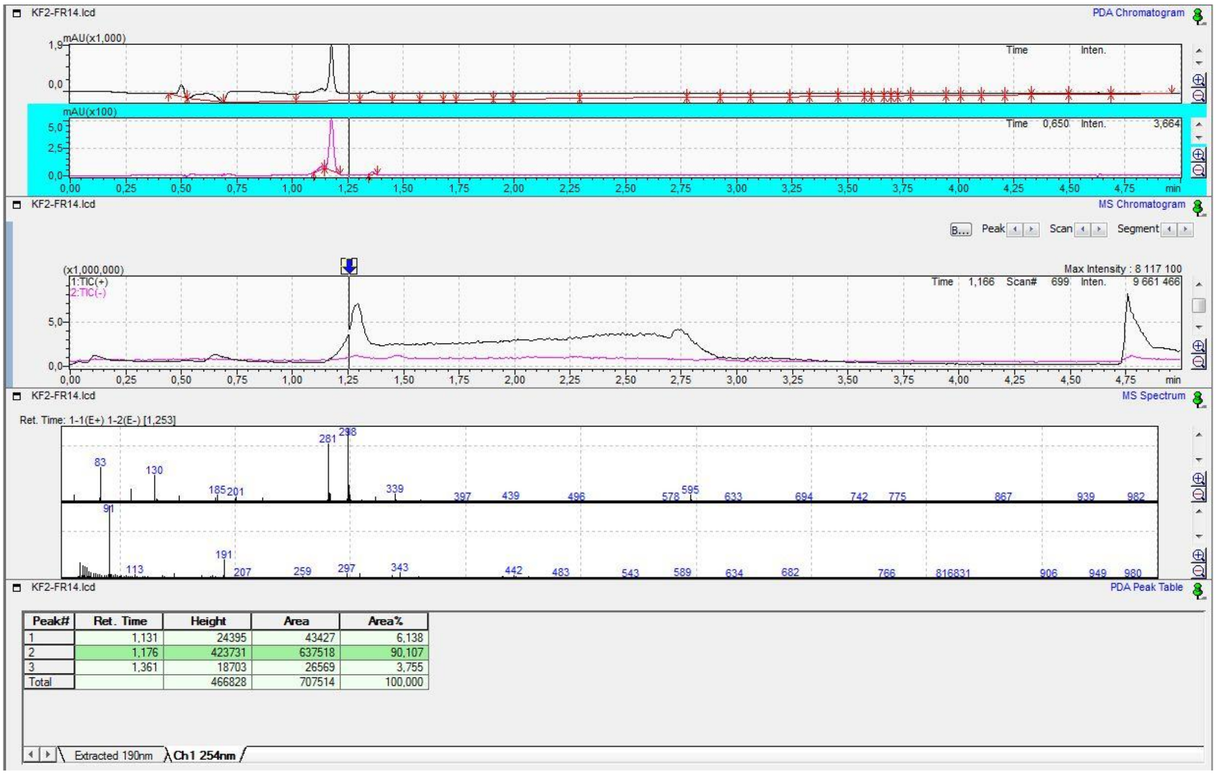

Peak#	Ret. Time	Height	Area	Area%
1	1,131	24395	43427	6,138
2	1,176	423731	637518	90,107
3	1,361	18703	26569	3,755
Total		466828	707514	100,000

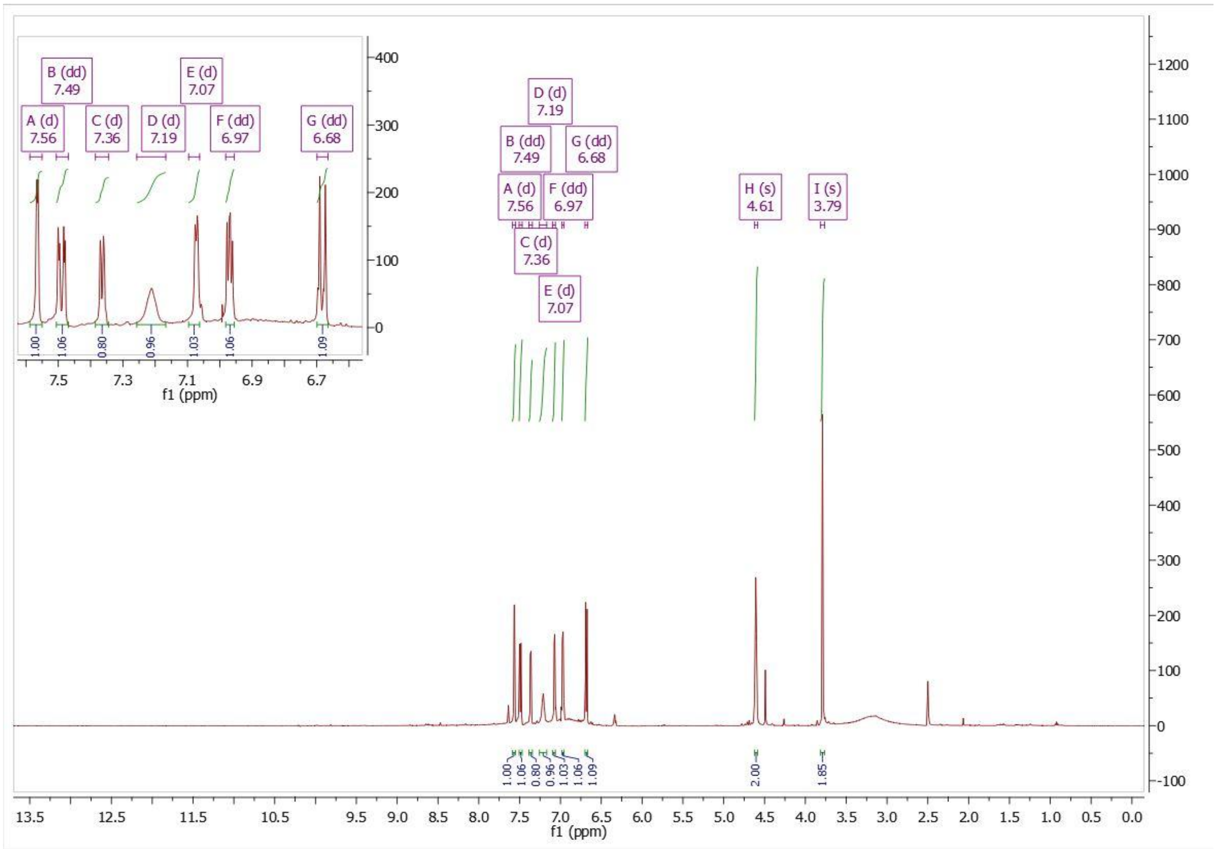
A (d) 7.56
B (dd) 7.49
C (d) 7.36
D (d) 7.19
E (d) 7.07
F (dd) 6.97
G (dd) 6.68
H (s) 4.61
I (s) 3.79
1.00
1.06
0.80
0.96
1.03
1.06
1.09
2.00
1.85
f1 (ppm)

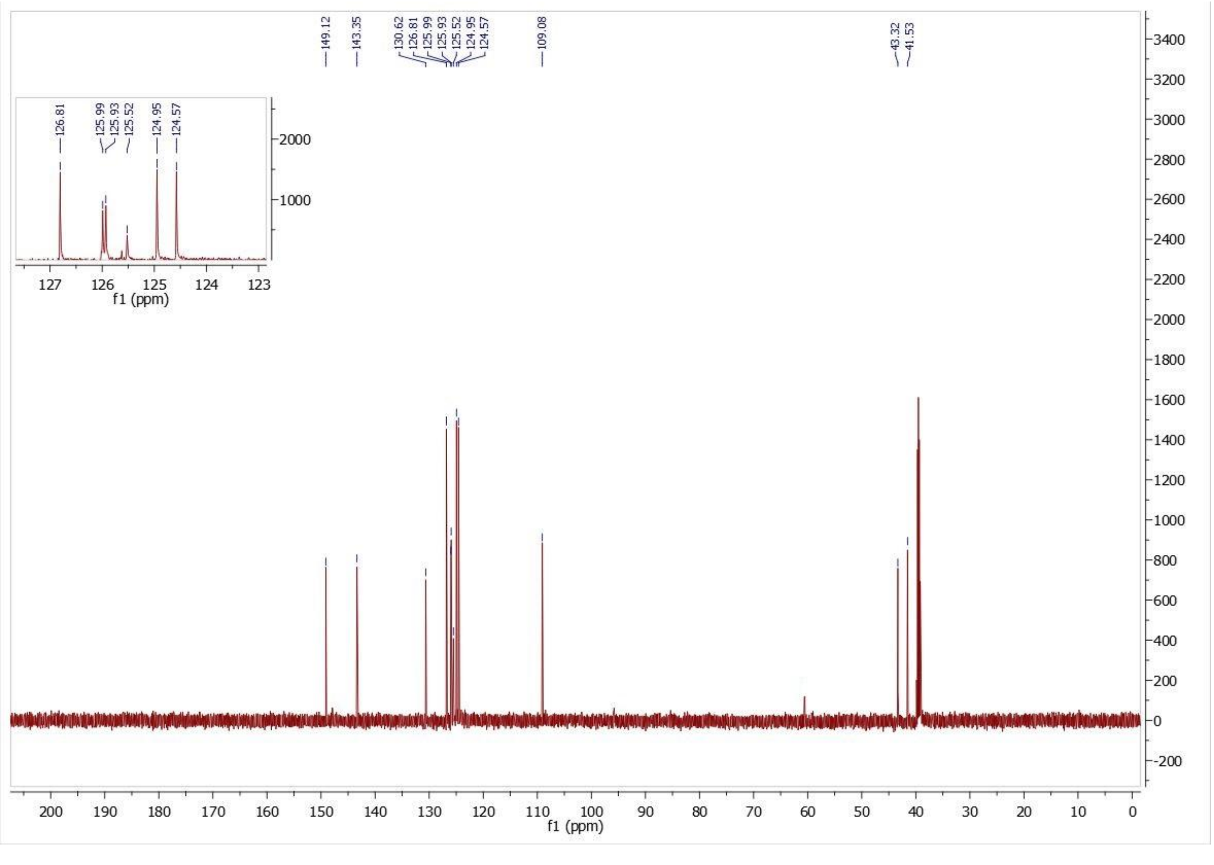
149.12
143.35
130.62
126.81
125.99
125.93
125.52
124.95
124.57
109.08
43.32
41.53
f1 (ppm)

2-Chloro-*N*-[(5-sulfamoyl-2-[[(thiophen-2-yl)methyl]amino]phenyl)methyl]pyrimidine-4-carboxamide (67)

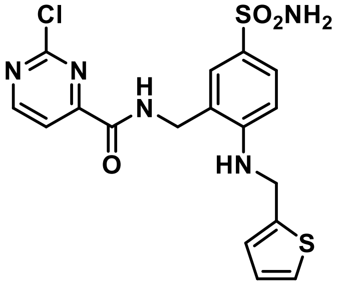

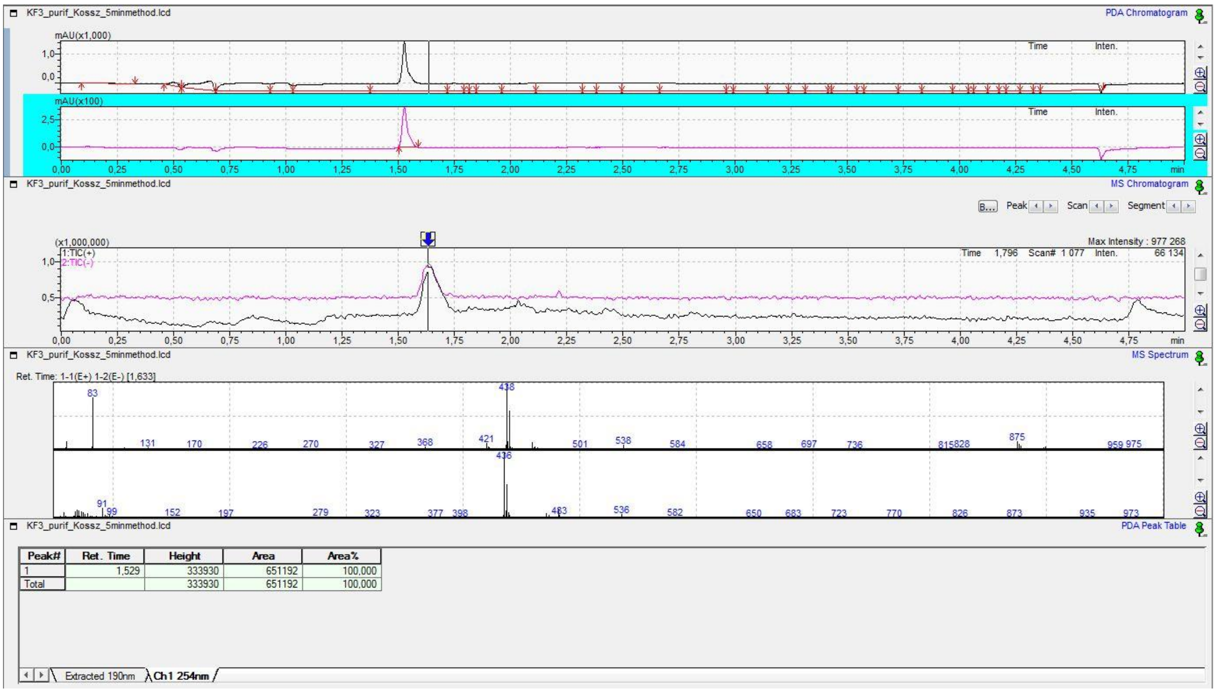

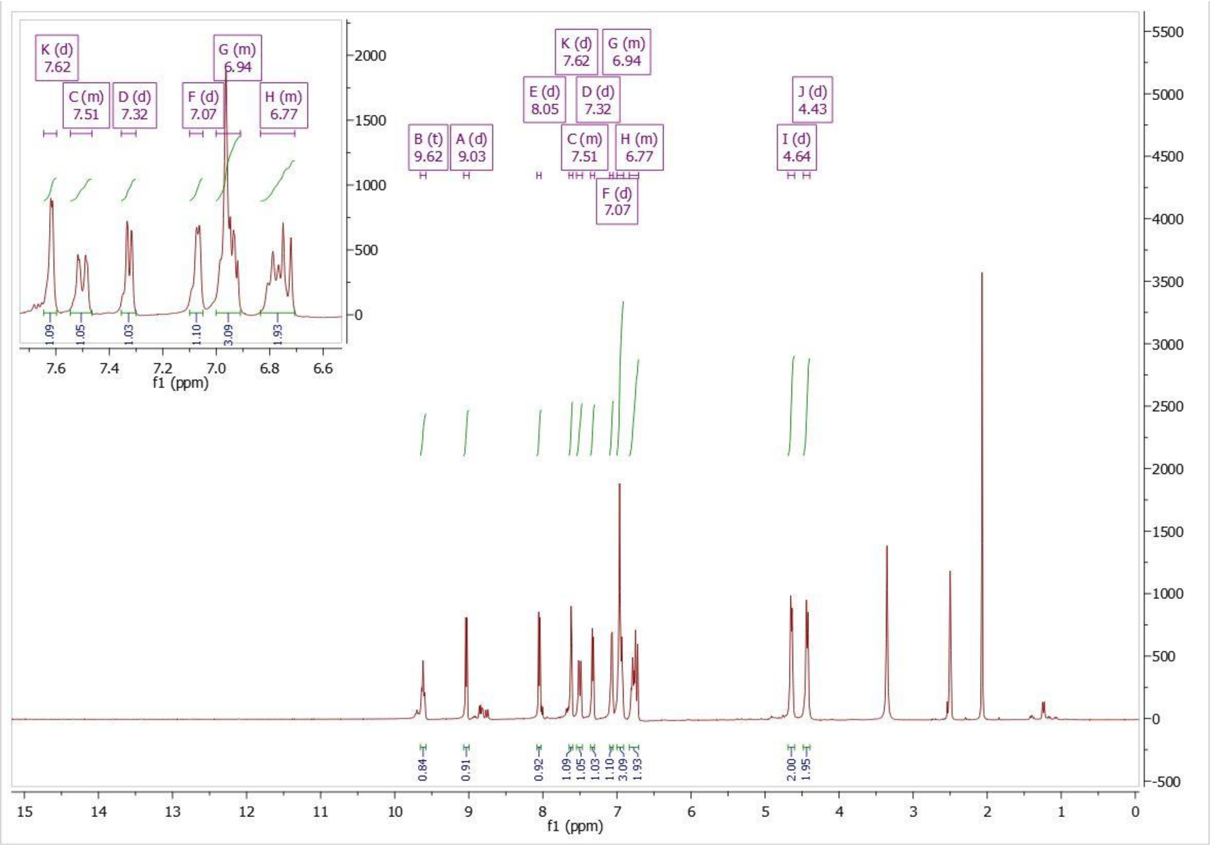
K (d) 7.62
C (m) 7.51
D (d) 7.32
F (d) 7.07
G (m) 6.94
H (m) 6.77
E (d) 8.05
B (t) 9.62
A (d) 9.03
J (d) 4.43
I (d) 4.64
1.09 1.05 1.03 1.10 3.09 1.93
0.84 0.91 0.92 2.00 1.95
f1 (ppm)

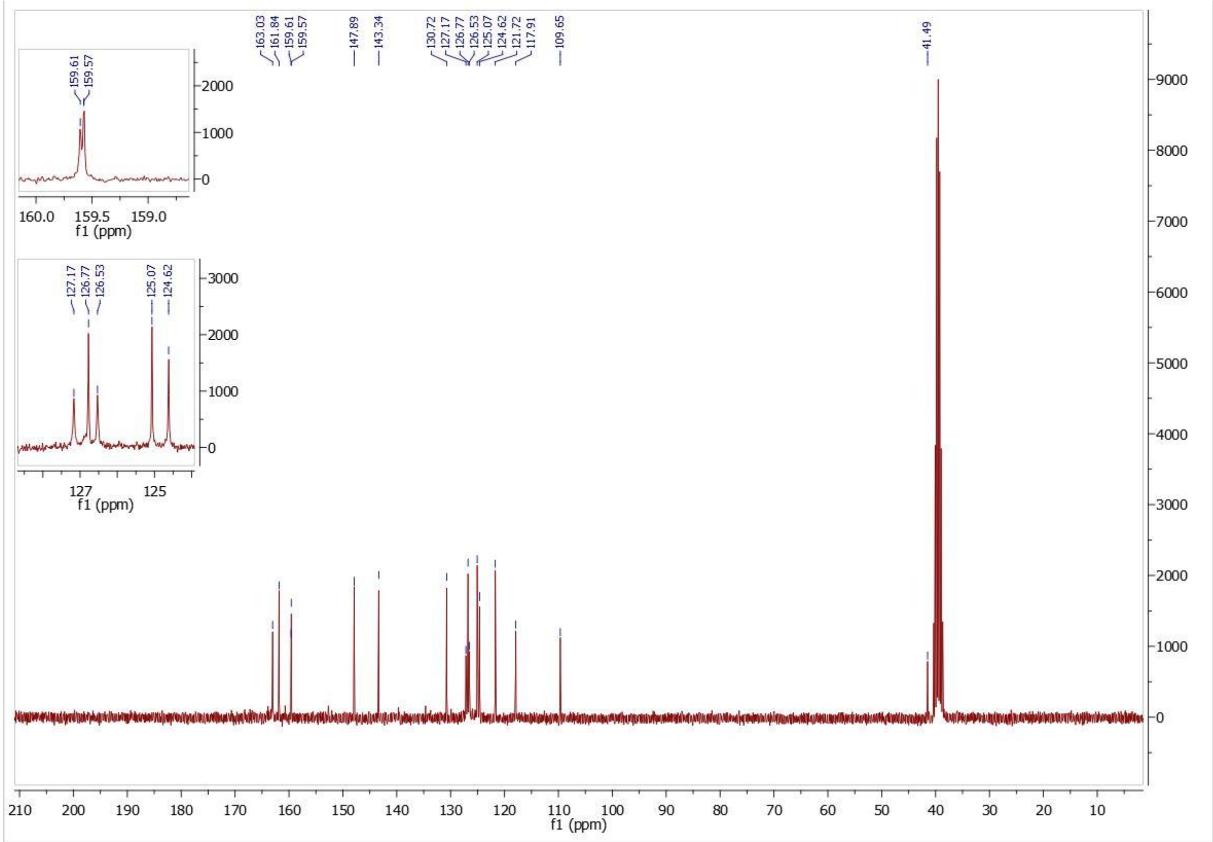
163.03 161.84 159.61 159.57 147.89 143.34 130.72 127.17 126.77 126.53 125.07 124.62 121.72 117.91 109.65 41.49
159.61 159.57
127.17 126.77 126.53 125.07 124.62
f1 (ppm)

2-Cyclopropyl-6-methyl-*N*-[(4-sulfamoylphenyl)methyl]pyrimidine-4-carboxamide (68)

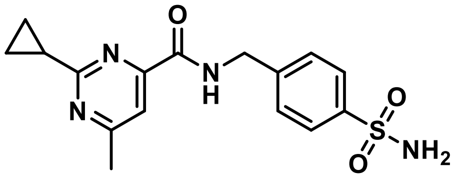

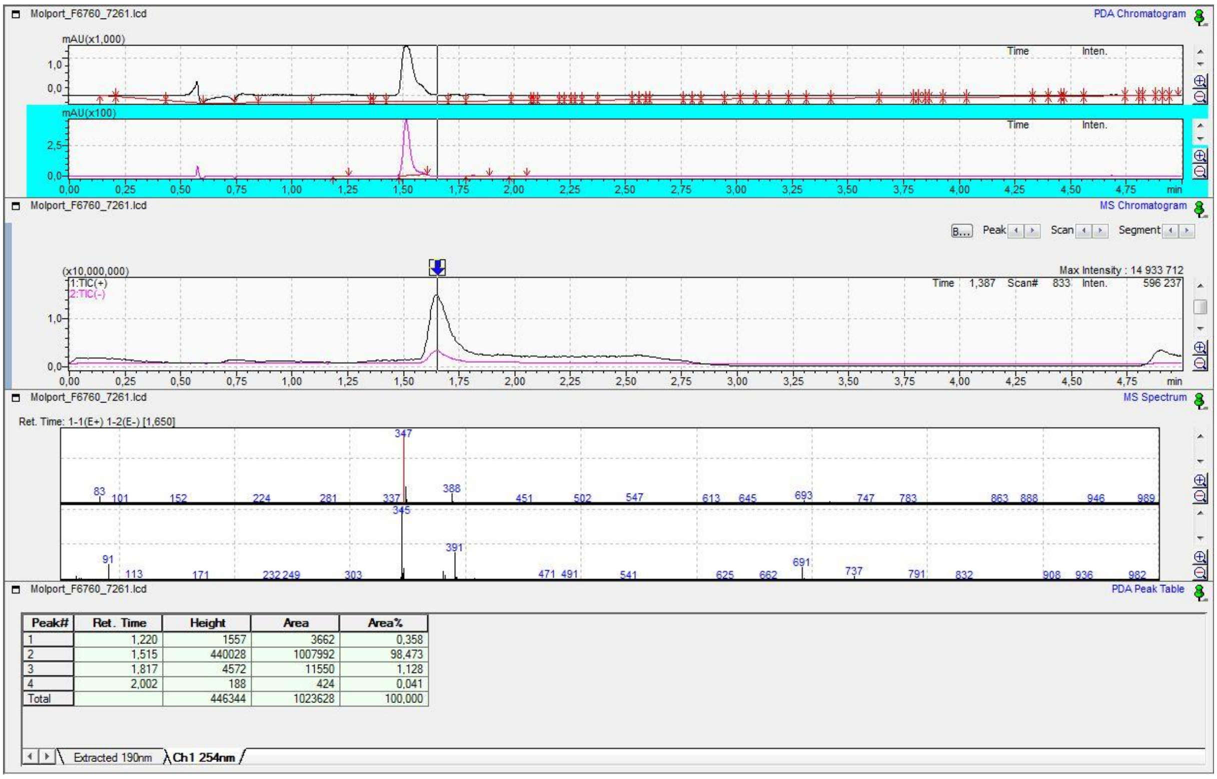

Peak#	Ret. Time	Height	Area	Area%
1	1,220	1557	3662	0,358
2	1,515	440028	1007992	98,473
3	1,817	4572	11550	1,128
4	2,002	188	424	0,041
Total		446344	1023628	100,000

***N*-[1-(4-methanesulfonylphenyl)ethyl]-2,6-dimethylpyrimidine-4-carboxamide (69)**

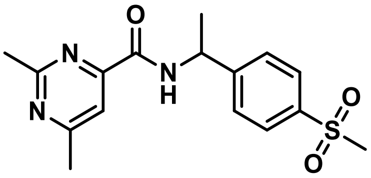

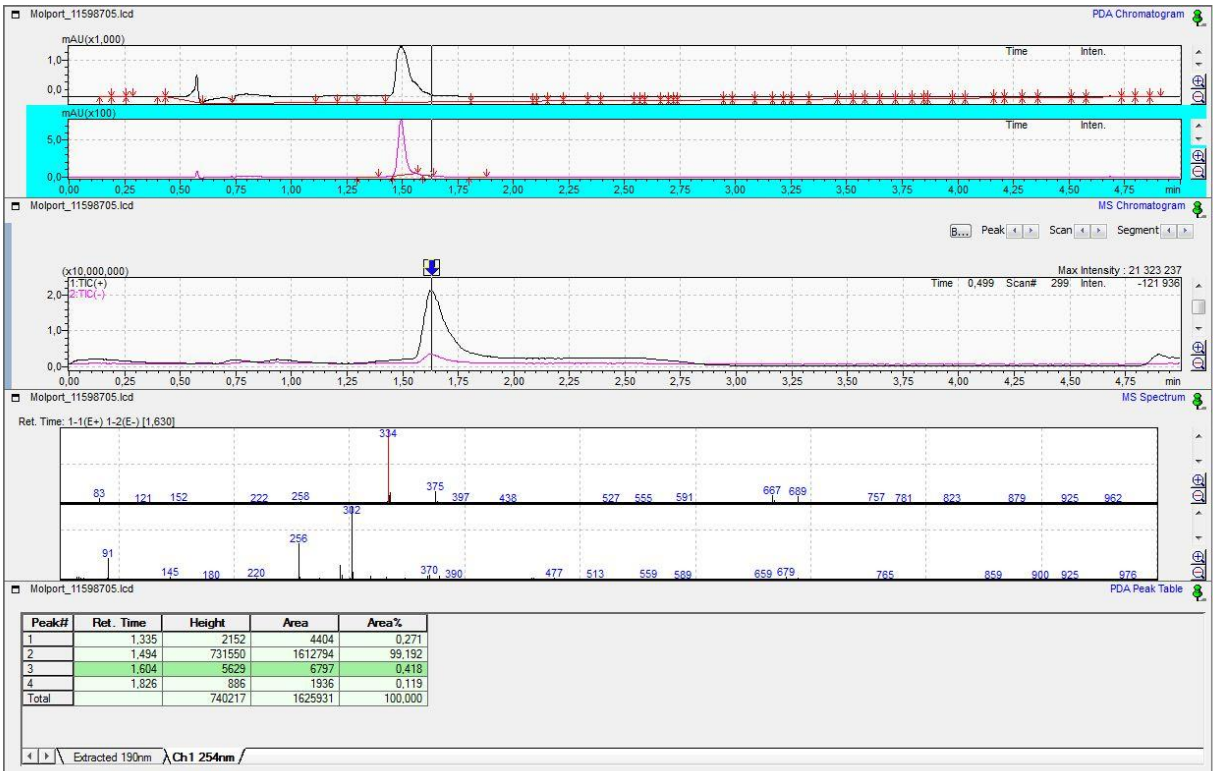

2-Methyl-*N*-[(4-sulfamoylphenyl)methyl]-6-(trifluoromethyl)pyrimidine-4-carboxamide (70)

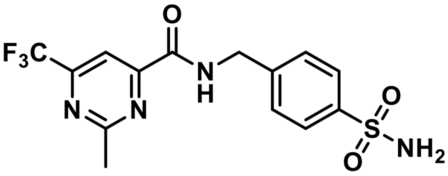

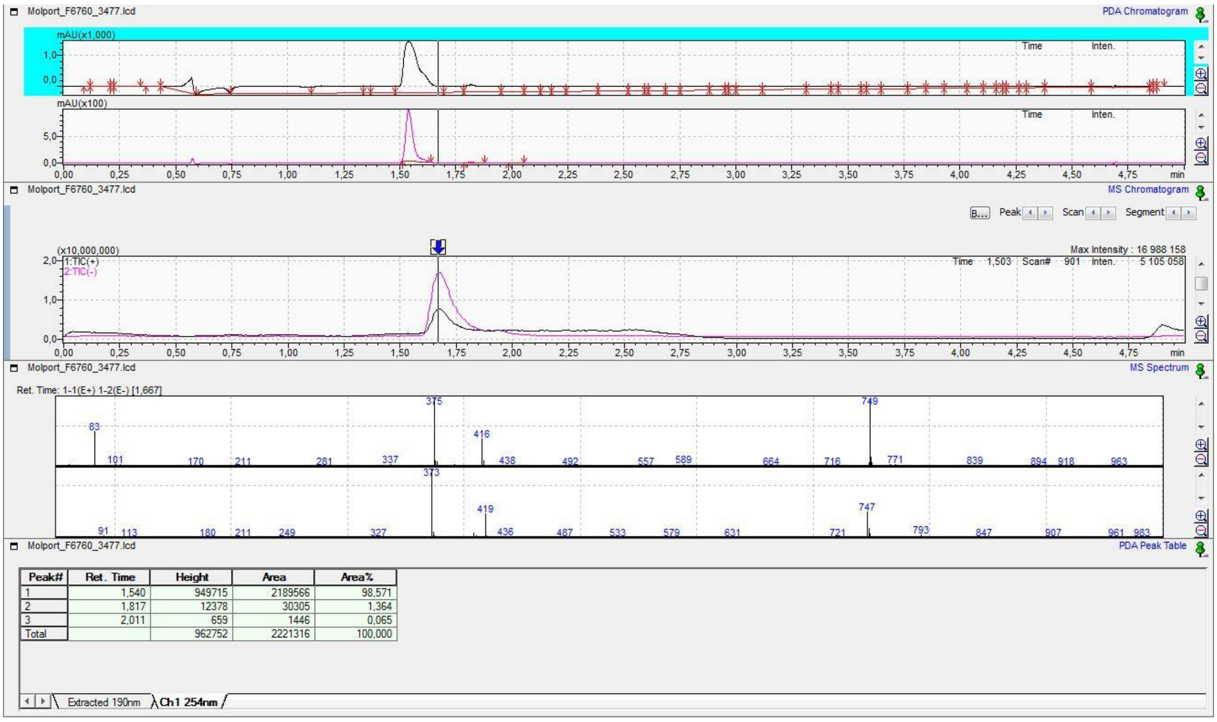

N-[(2-bromo-5-sulfamoylphenyl)methyl]pyrimidine-4-carboxamide (71)

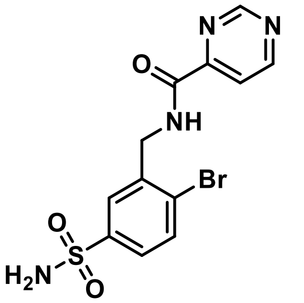

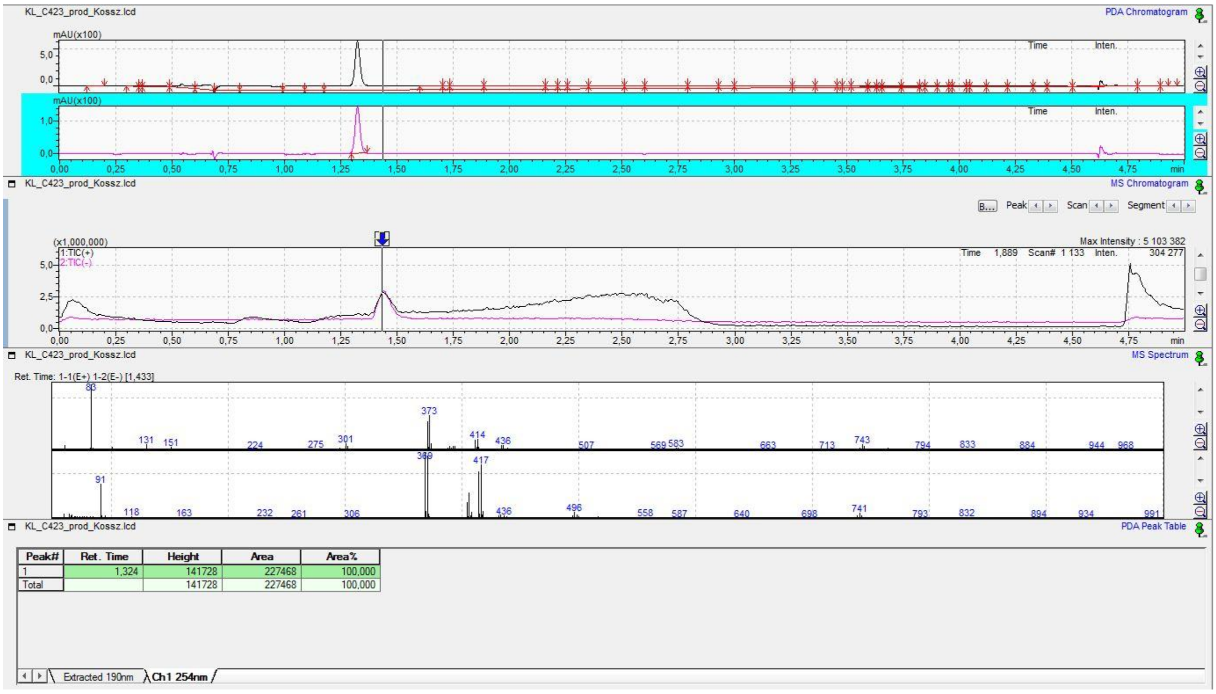

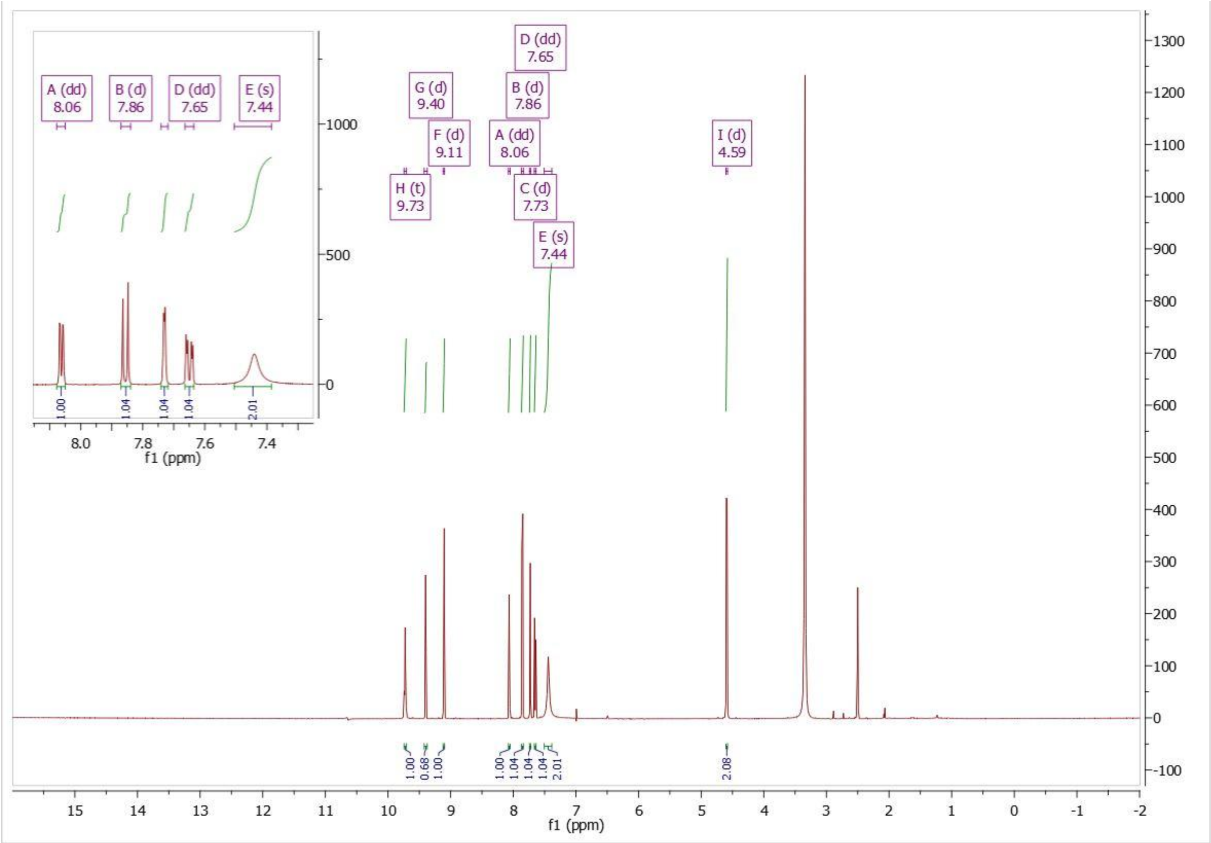
A (dd)
8.06
B (d)
7.86
C (d)
7.73
D (dd)
7.65
E (s)
7.44
F (d)
9.11
G (d)
9.40
H (t)
9.73
I (d)
4.59
f1 (ppm)

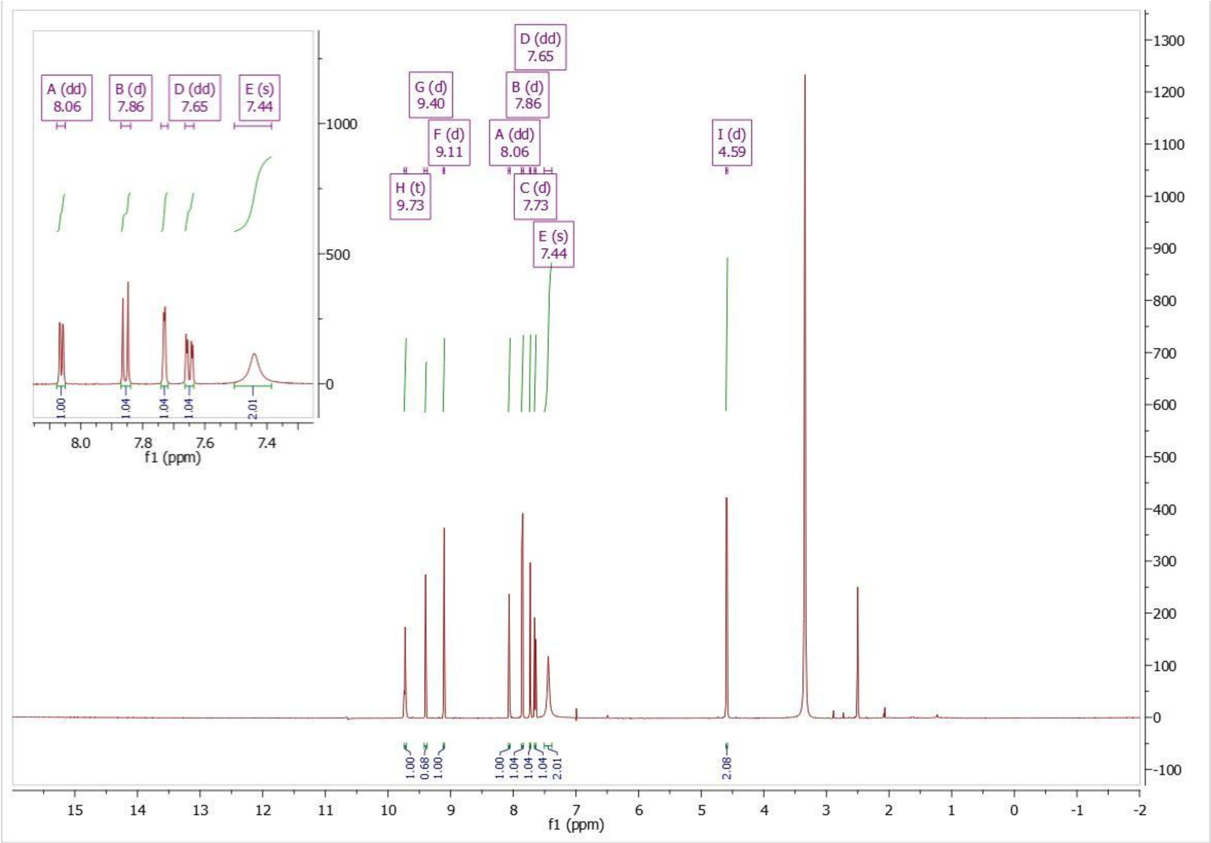
A (dd)
8.06
B (d)
7.86
C (d)
7.73
D (dd)
7.65
E (s)
7.44
F (d)
9.11
G (d)
9.40
H (t)
9.73
I (d)
4.59
f1 (ppm)

Ethylbis(propan-2-yl)azanium 4-bromo-3-[[(pyrimidin-4-yl)formamido]methyl]benzene-1-sulfonate (73)

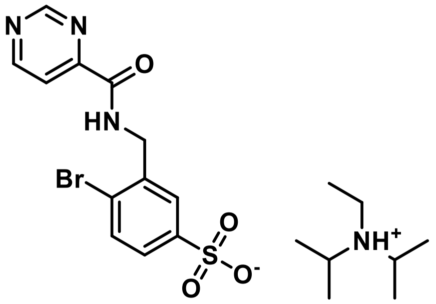

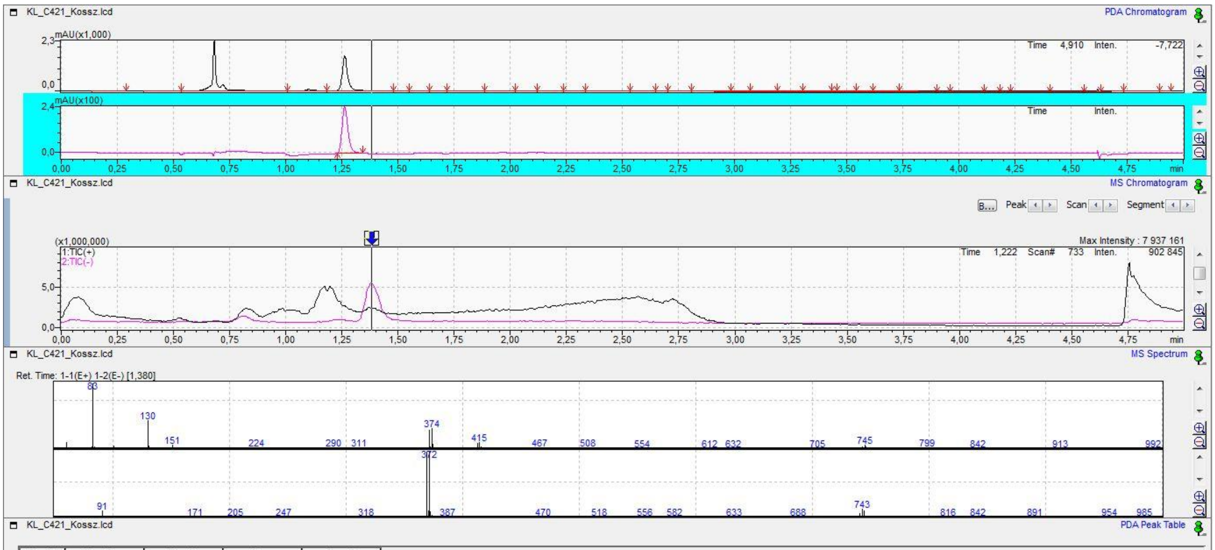

Peak#	Ret. Time	Height	Area	Area%
1	1,263	232249	443630	100,000
Total		232249	443630	100,000

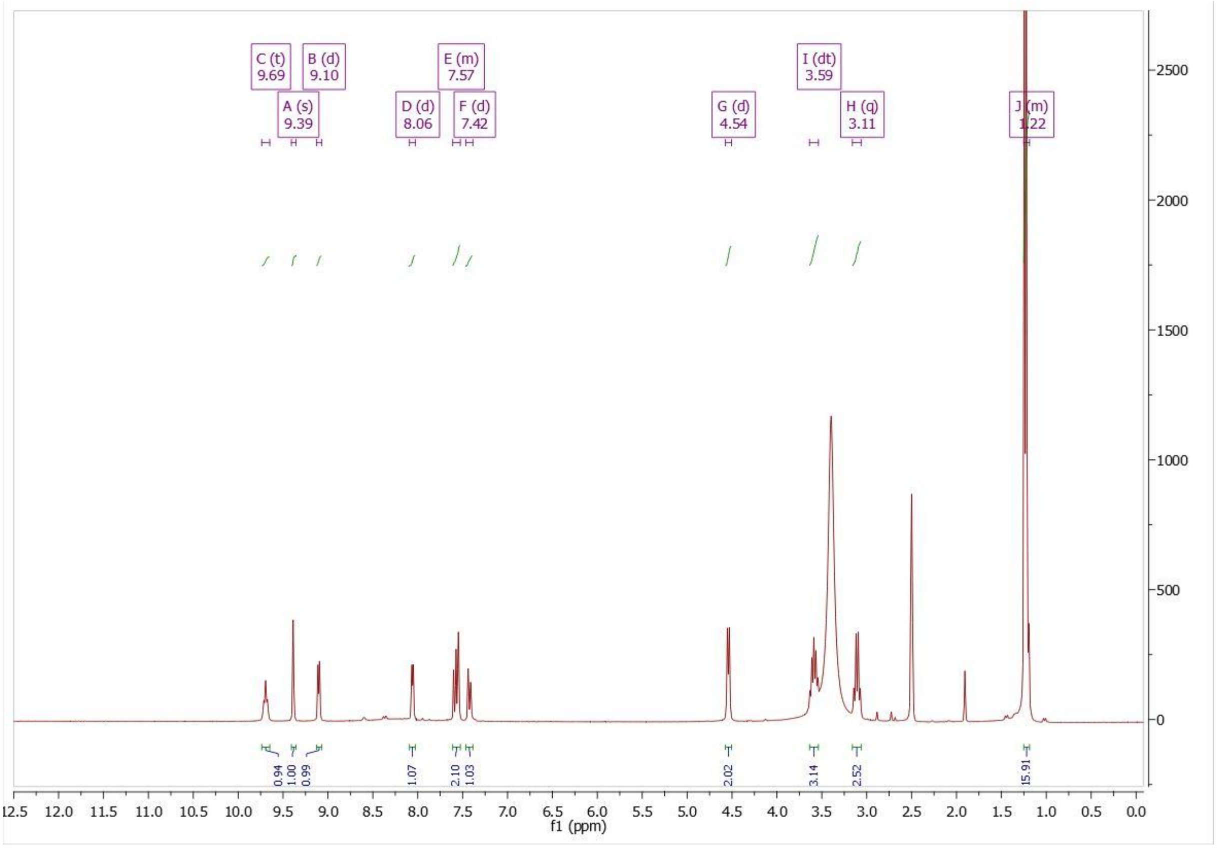
C (t) 9.69
B (d) 9.10
E (m) 7.57
I (dt) 3.59
A (s) 9.39
D (d) 8.06
F (d) 7.42
G (d) 4.54
H (q) 3.11
J (m) 1.22
0.94
1.00
0.99
1.07
2.10
1.03
2.02
3.14
2.52
15.91
f1 (ppm)

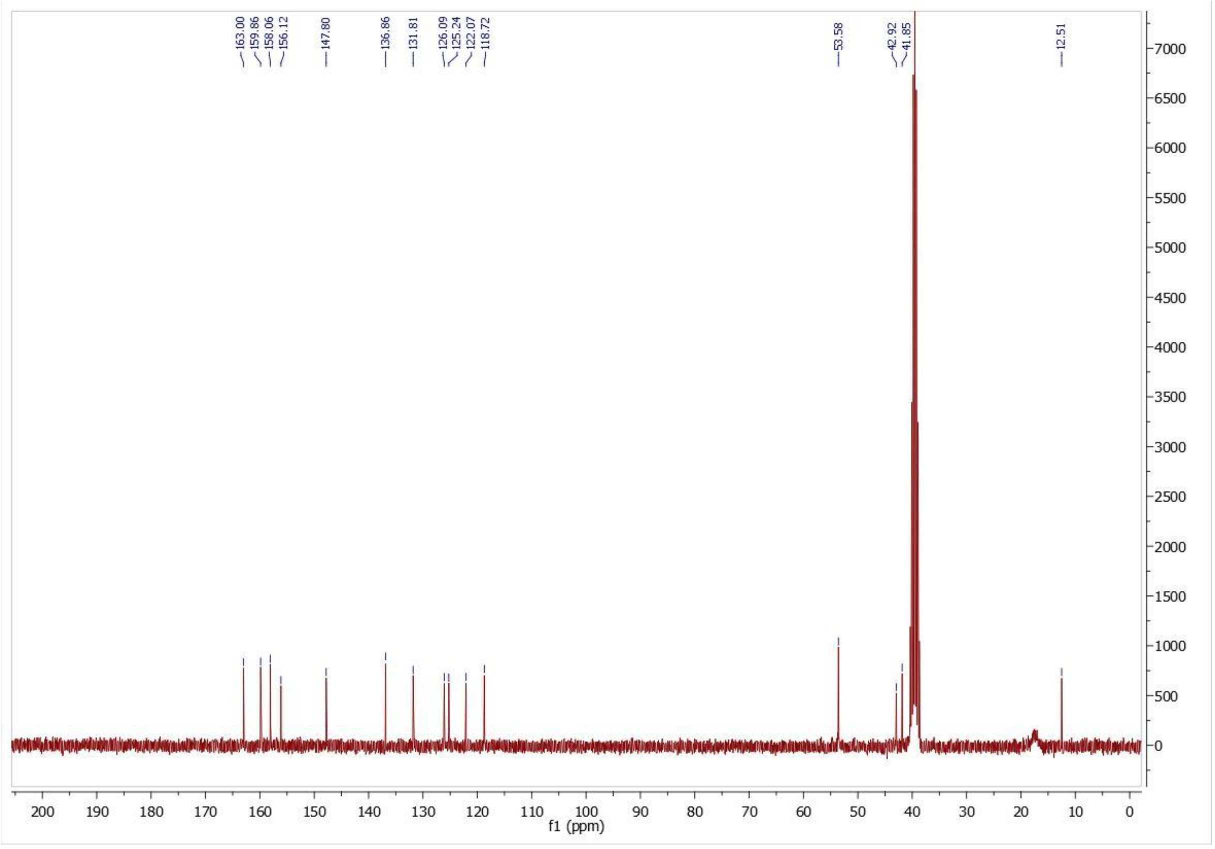
163.00
159.86
158.06
156.12
147.80
136.86
131.81
126.09
125.24
122.07
118.72
53.58
42.92
41.85
12.51
f1 (ppm)

N-(4-methylpyridin-3-yl)acetamide (74)

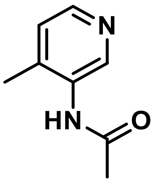

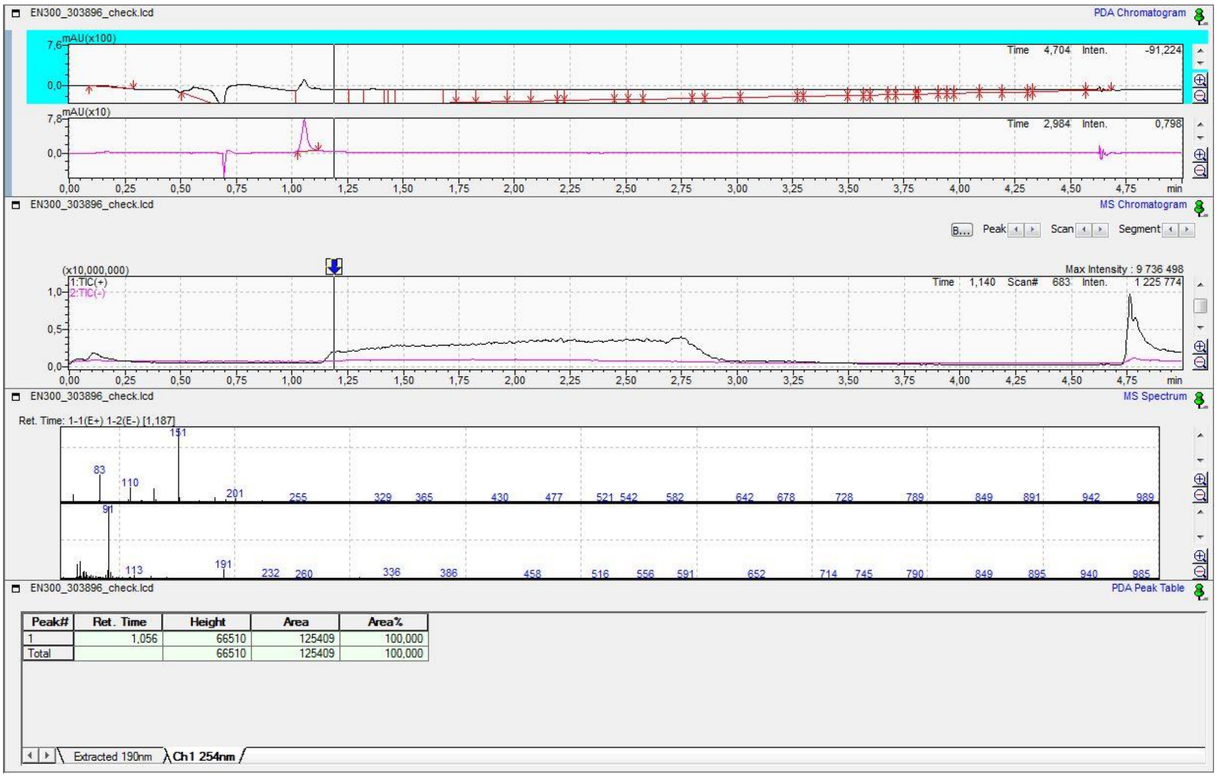

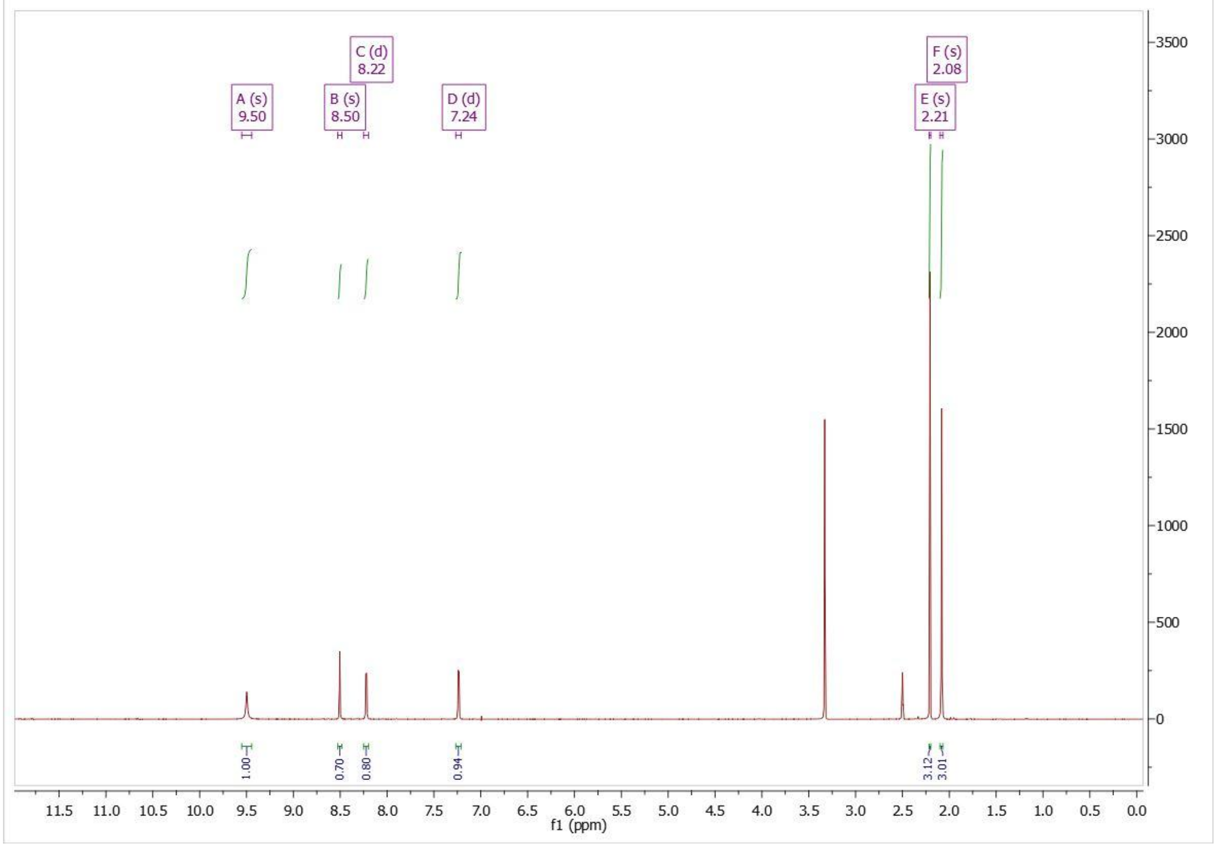
A (s)
9.50
B (s)
8.50
C (d)
8.22
D (d)
7.24
E (s)
2.21
F (s)
2.08
1.00
0.70
0.80
0.94
3.12
3.01
f1 (ppm)

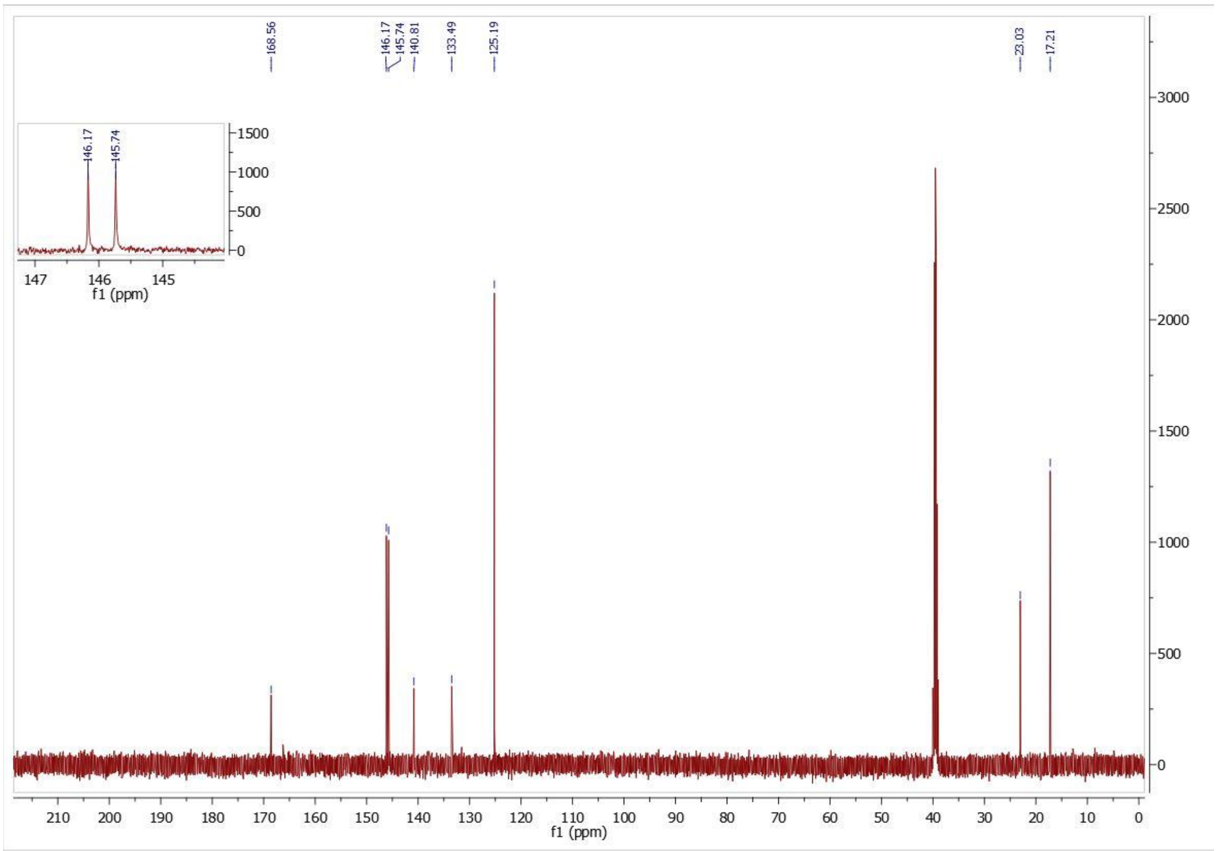
168.56
146.17
145.74
140.81
133.49
125.19
23.03
17.21
f1 (ppm)

10. Supplementary Figures

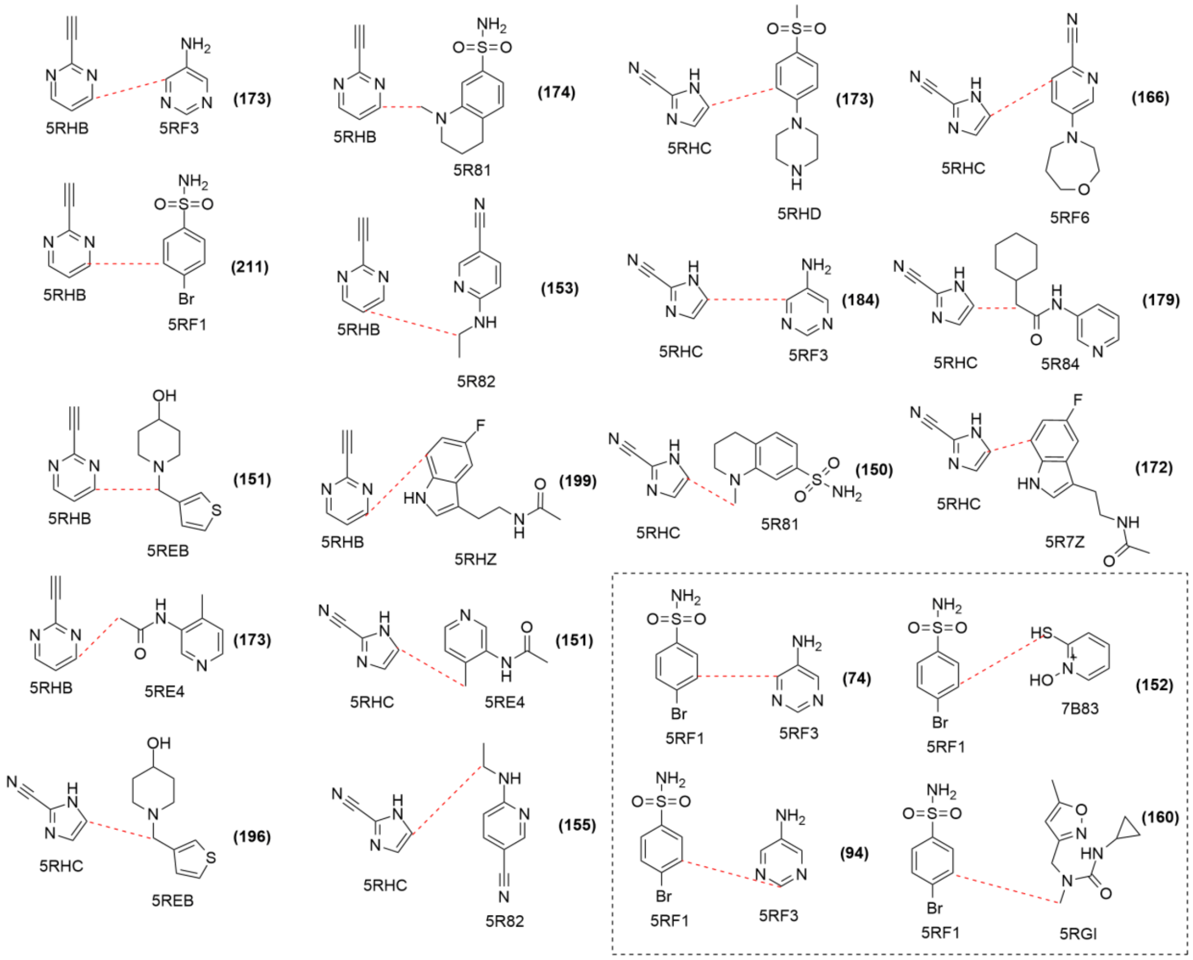

Figure S1. Fragment pairs subjected to DeLinker. The linking between the two anchor atoms is shown with red dashes. The numbers in the brackets show the number of generated molecules. The origin of the given fragment is shown as PDB codes under the molecules. Non-covalent pairs are shown within the dashed rectangle.

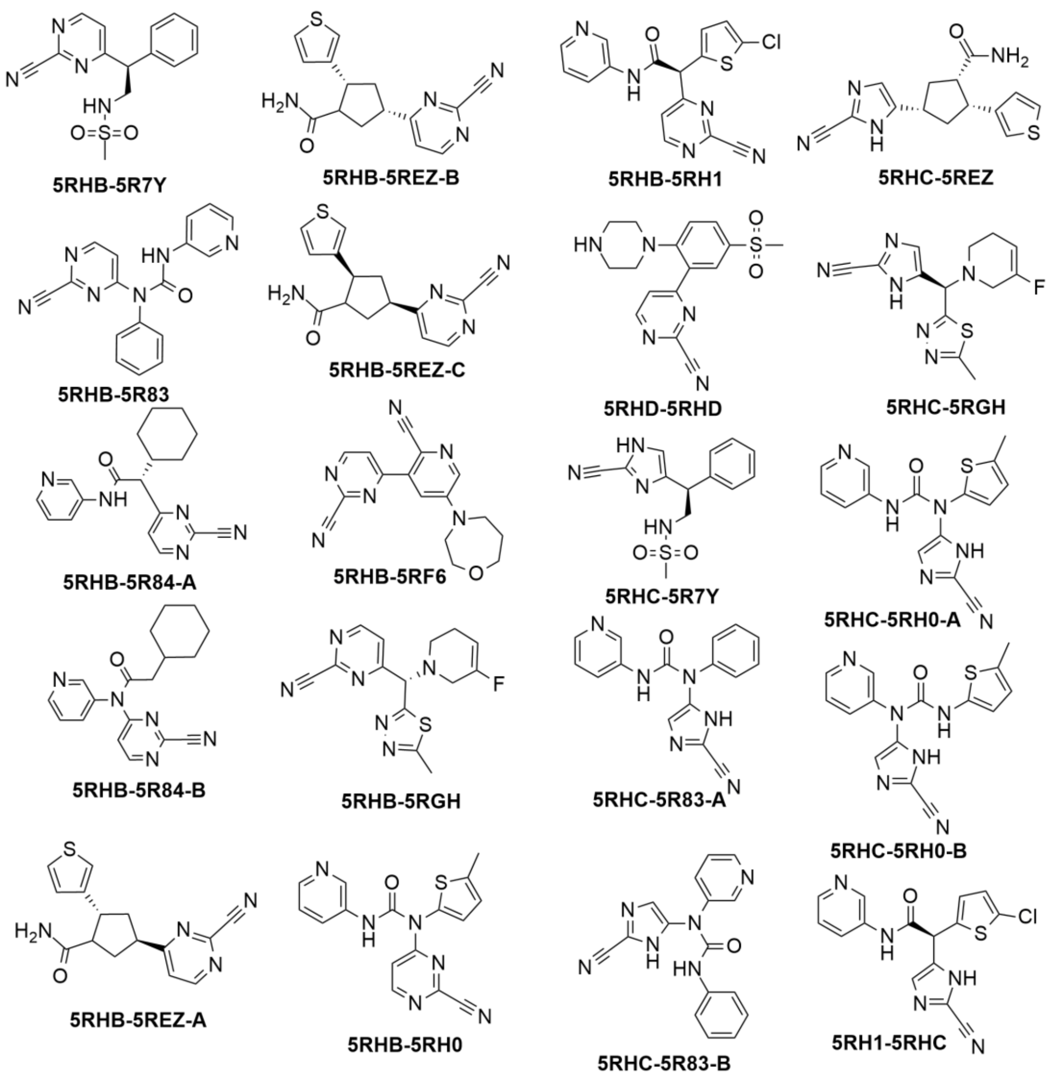

Figure S2. Designed compounds using direct fragment merging. The names contain the merged fragments, where more than one possibility emerged an additional letter denotes the different variations.

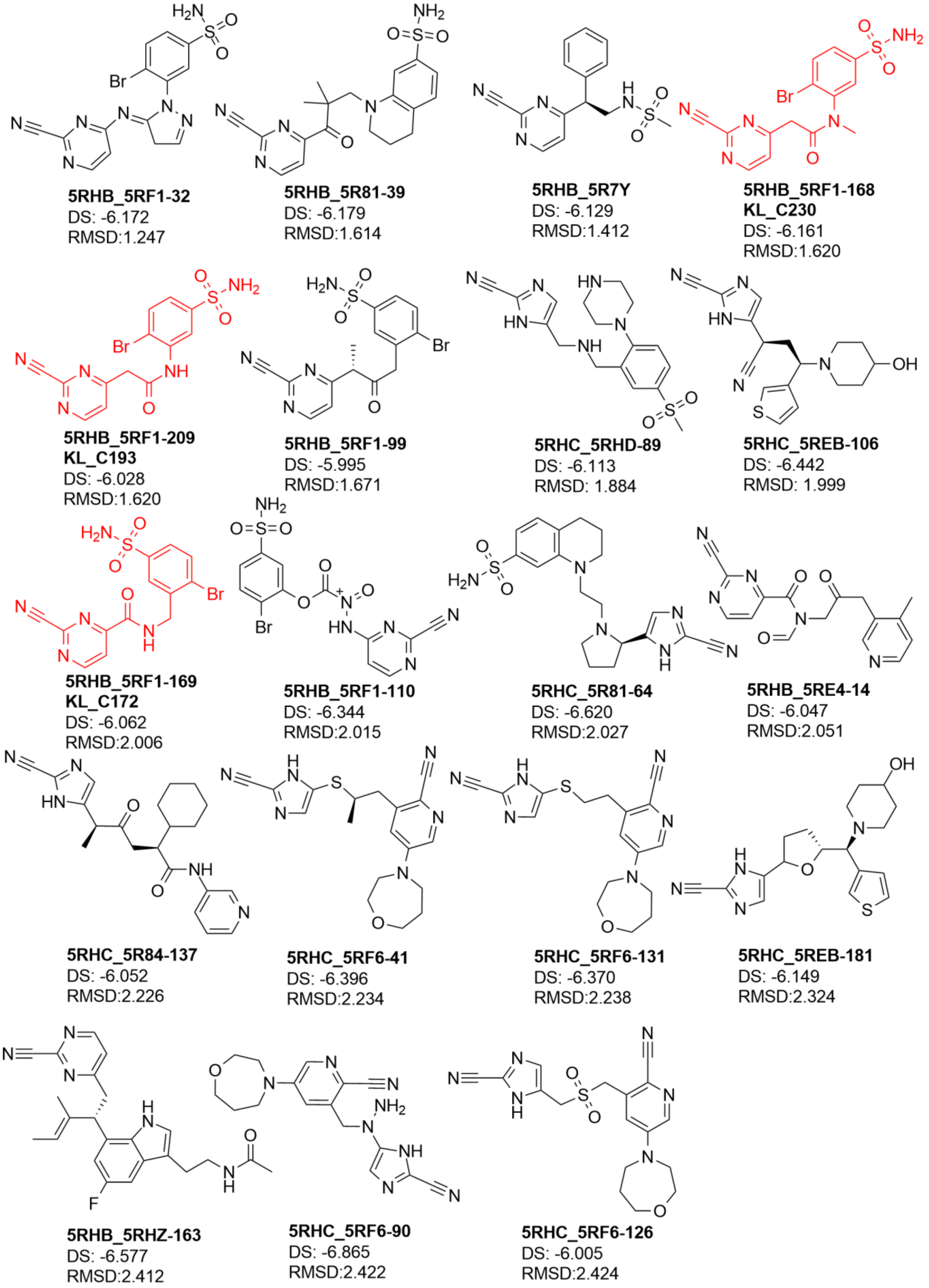

Figure S3. Designed compounds that were evaluated for synthetic feasibility. The naming emerges from the linked fragments. The number following the PDB codes is the serial number in the respective DeLinker job. The docking score values and RMSD compared to the original fragment positions are shown. Selected compounds are shown in red.

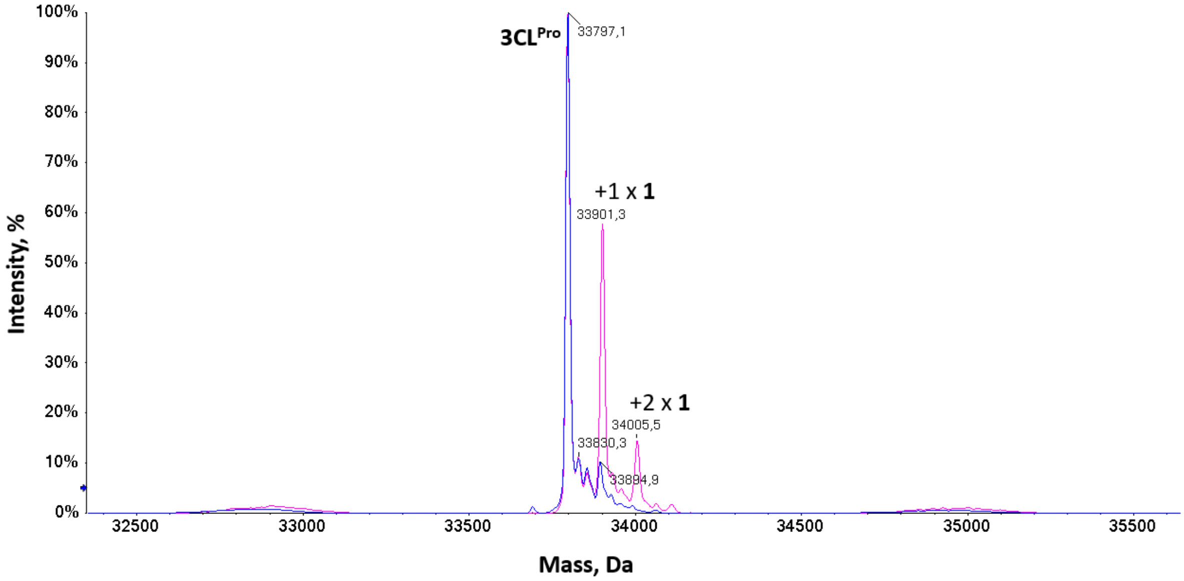

Figure S4. Deconvoluted mass spectrum of the reference 3CL^{pro} (blue) and the **1** modified 3CL^{pro} (pink).

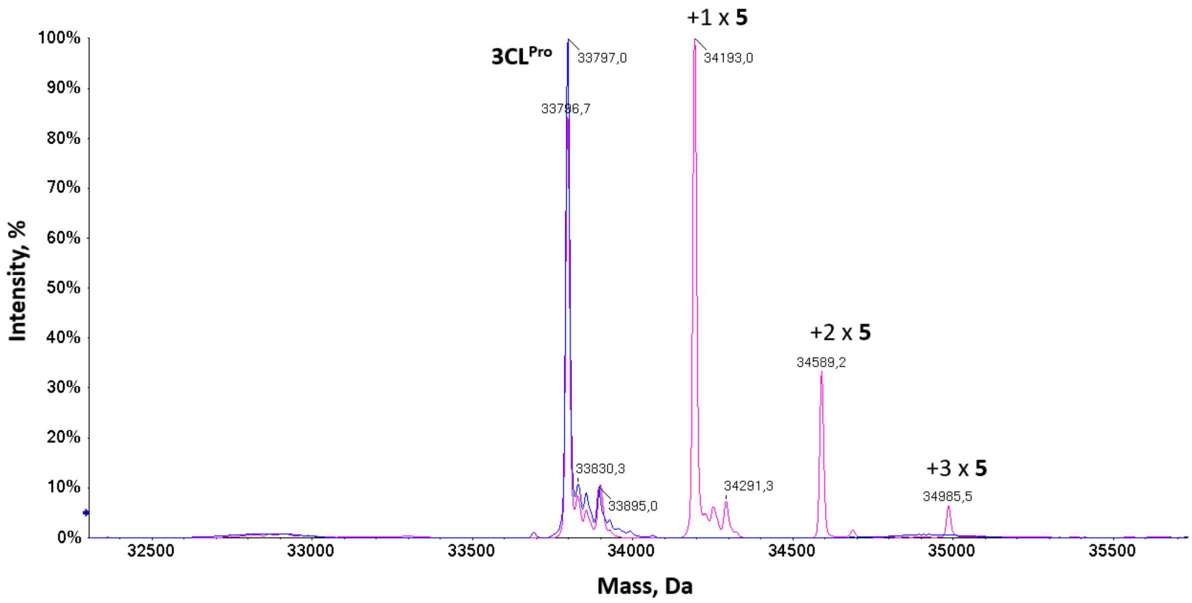

Figure S5. Deconvoluted mass spectrum of the reference 3CL^{pro} (blue) and the **5** modified 3CL^{pro} (pink).

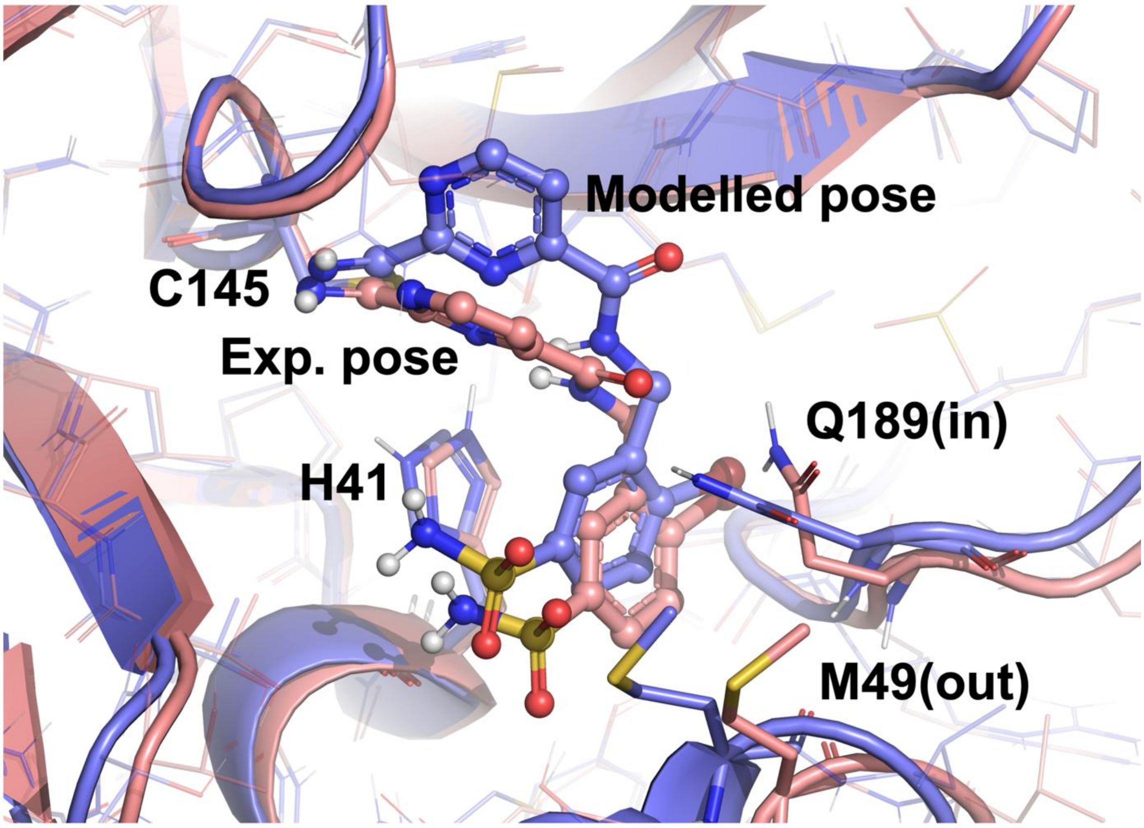

Figure S6. Modeled structure (purple) of the **5**-3CL^{pro} complex acquired by covalent docking into the M49(out)-Q189(in) structure of the main protease (7RNK) vs. its experimentally determined binding mode (salmon).

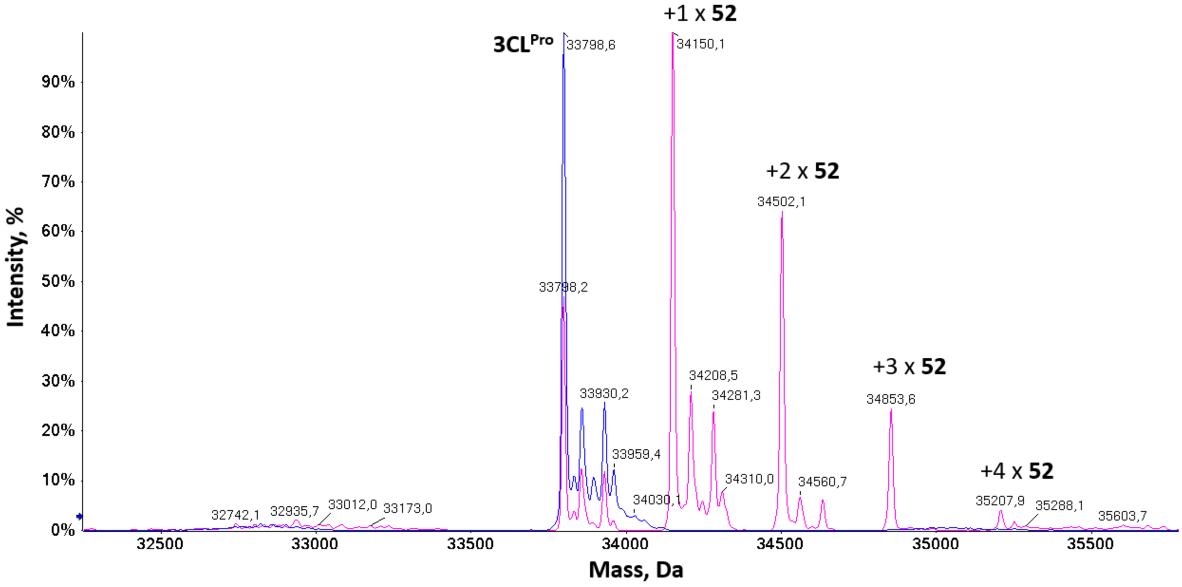

Figure S7. Deconvoluted mass spectrum of the reference 3CL^{pro} (blue) and the **52** modified 3CL^{pro} (pink).

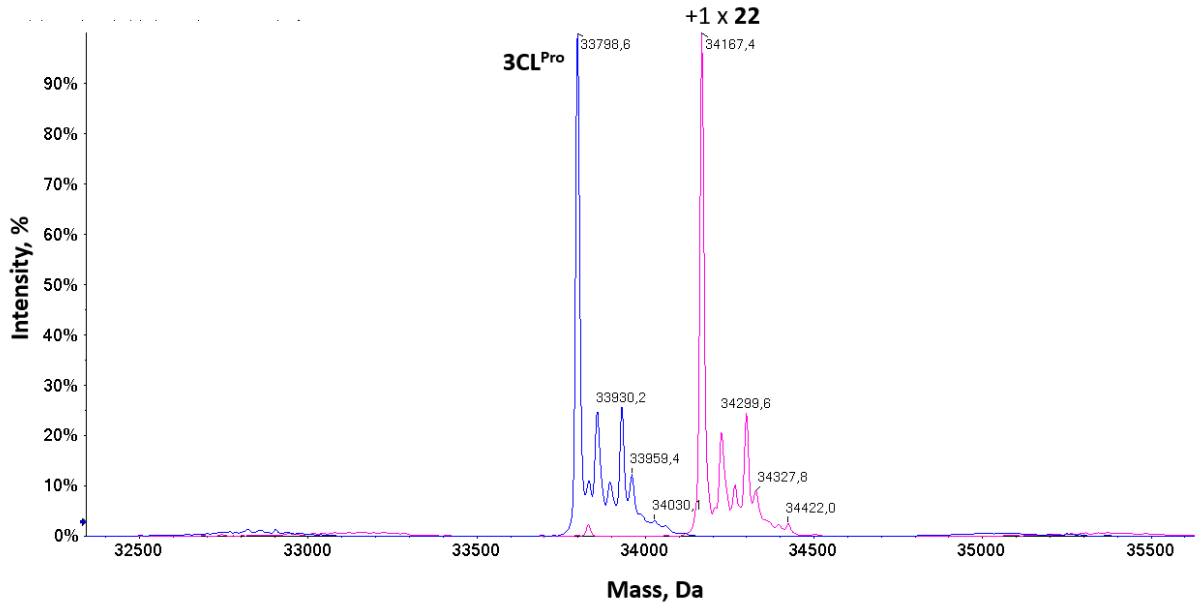

Figure S8. Deconvoluted mass spectrum of the reference 3CL^{pro} (blue) and the **22** modified 3CL^{pro} (pink).

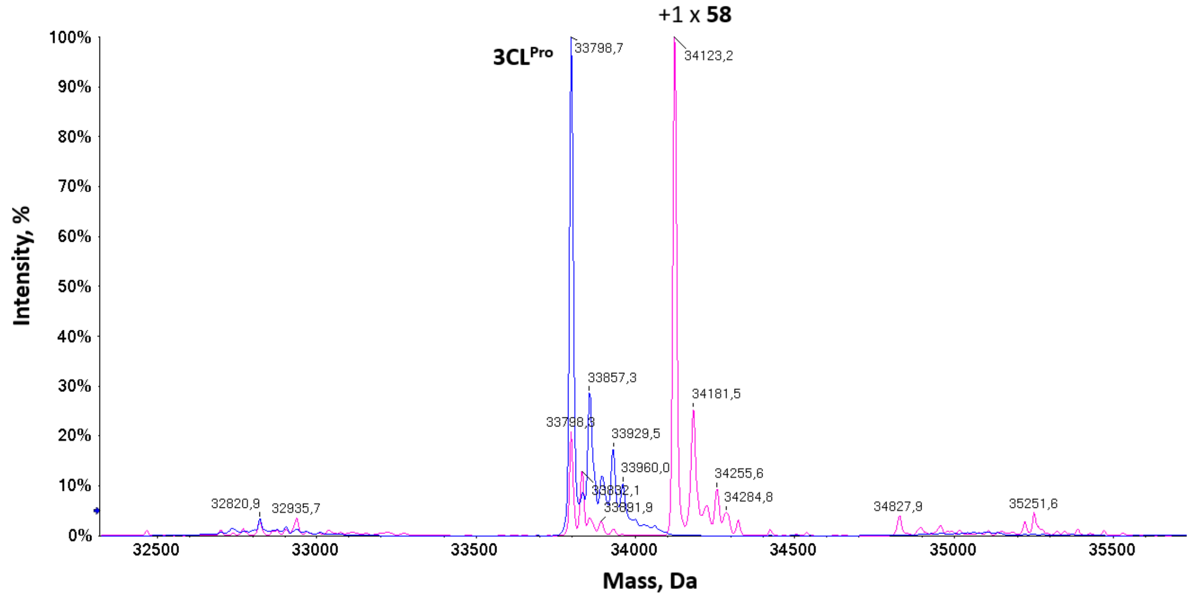

Figure S9. Deconvoluted mass spectrum of the reference 3CL^{pro} (blue) and the **58** modified 3CL^{pro} (pink).

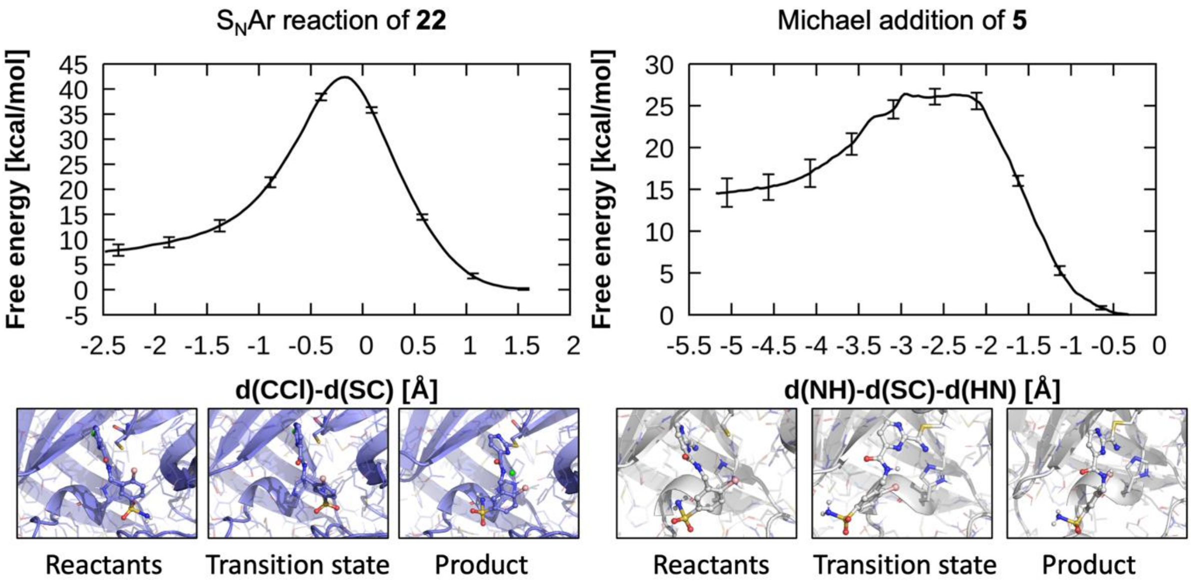

Figure S10. Results of the QM/MM-umbrella sampling calculations for the reaction between Cys145 and **22/5**: applied reaction coordinates, the modelled reactions, highlighted states (reactants, transition state, product) and constructed PMFs.

References

[1] S. Izrailev, S. Stepaniants, B. Isralewitz, D. Kosztin, H. Lu, F. Molnar, W. Wriggers, K. Schulten, "Steered Molecular Dynamics" **2011**, 39–65.

[2] G. M. Torrie, J. P. Valleau, "Nonphysical sampling distributions in Monte Carlo free-energy estimation: Umbrella sampling" *J. Comput. Phys.* **1977**, *23,* 187–199.

[3] W. L. Jorgensen, J. Chandrasekhar, J. D. Madura, R. W. Impey, M. L. Klein, "Comparison of simple potential functions for simulating liquid water" *J. Chem. Phys.* **1983**, *79,* 926–935.

[4] M. Gaus, Q. Cui, M. Elstner, "DFTB3: Extension of the self-consistent-charge density-functional tight-binding method (SCC-DFTB)" *J. Chem. Theory Comput.* **2011**, *7,* 931–948.

[5] J. A. Maier, C. Martinez, K. Kasavajhala, L. Wickstrom, K. E. Hauser, C. Simmerling, "ff14SB: Improving the Accuracy of Protein Side Chain and Backbone Parameters from ff99SB" *J. Chem. Theory Comput.* **2015**, *11,* 3696–3713.

[6] S. Kumar, J. M. Rosenberg, D. Bouzida, R. H. Swendsen, P. A. Kollman, "THE weighted histogram analysis method for free-energy calculations on biomolecules. I. The method" *J. Comput. Chem.* **1992**, *13,* 1011–1021.

[7] A. Grossfield, "WHAM: an implementation of the weighted histogram analysis method," **n.d.**

[8] M. Bonomi, D. Branduardi, G. Bussi, C. Camilloni, D. Provasi, P. Raiteri, D. Donadio, F. Marinelli, F. Pietrucci, R. A. Broglia, M. Parrinello, "PLUMED: A portable plugin for free-energy calculations with molecular dynamics" *Comput. Phys. Commun.* **2009**, *180,* 1961–1972.

[9] D. A. Safin, N. A. Tumanov, A. A. Leitch, J. L. Brusso, Y. Filinchuk, M. Murugesu, "Elucidating the elusive crystal structure of 2,4,6-tris(2-pyrimidyl)-1,3,5-triazine" *CrystEngComm* **2015**, *17*, 2190–2195.

[10] K. Govindan, N. Q. Chen, Y. W. Chuang, W. Y. Lin, "Unlocking Amides through Selective C–N Bond Cleavage: Allyl Bromide-Mediated Divergent Synthesis of Nitrogen-Containing Functional Groups" *Org. Lett.* **2021**, *23,* 9419–9424.

[11] J. Popovici-Muller, F. G. Salituro, J. O. Saunders, J. Travins, Y. Shungi, *Therapeutic Compounds and Compositions,* **2014**, WO2014062511A1.

[12] K. Kyung-Hee, C. Jung-Wook, L. Ji-Hoon, K. Shin-Ae, J. Kyung-Jin, K. Jun-Woo, M. Sang-Hyun, Y. Ji-Hoon, L. Ju-Suk, L. Won-Seok, S. Jae-Young, L. Eung-Seok, J. Tae-

Cheon, K. Jung-Ae, *2-Cyanopyrimidin-4-Yl Carbamate or Urea Derivative or Salt Thereof, and Pharmaceutical Composition Including Same*, **2019**, WO2019190117.
